# Synthesis and Biological Activity of 2,6‐Disubstituted and 2,6,9‐Trisubstituted 7‐Deazapurine Nucleobases

**DOI:** 10.1002/cmdc.70419

**Published:** 2026-08-13

**Authors:** Ugnė Šinkevičiūtė, Kateřina Burgetová Ječmeňová, Soňa Gurská, Lenka Poštová Slavětínská, Vincent Baran, Pavel Polishchuk, Petr Džubák, Michal Hocek, Marián Hajdúch, Michal Tichý

**Affiliations:** ^1^ Institute of Organic Chemistry and Biochemistry, Czech Academy of Sciences Prague 6 Czech Republic; ^2^ Department of Organic Chemistry Faculty of Science Charles University in Prague Prague 2 Czech Republic; ^3^ Institute of Molecular and Translational Medicine Faculty of Medicine and Dentistry Palacký University and University Hospital Olomouc Olomouc Czech Republic

**Keywords:** 7‐deazapurine, adenosine receptor antagonist, Suzuki cross‐coupling

## Abstract

High‐throughput screening of the IMTM compound library for modulatory activity on adenosine receptors (ARs) identified two deazapurine hits based on 2,6‐diaryl‐7‐deazapurines. In this study, a series of 16 new 2,6‐diaryl‐7‐deazapurine nucleobases together with four N‐9 substituted derivatives were synthesized. The synthesis was based on regioselective Suzuki cross‐coupling into position 6 followed by a second Suzuki cross‐coupling at position 2 under harsher conditions. Both cross‐couplings were performed on N‐9 protected nucleobases. Biological activity profiling identified several potent antagonists of all four subtypes of ARs and four compounds as sub‐micromolar and very selective antagonists of A_3_. Several final compounds showed potent in  vitro cytotoxic activity on a small panel of cancer cell lines, but with no selectivity against nonmalignant fibroblasts BJ and MRC‐5.

## Graphical abstract

Four series of 20 (het)aryl substituted 7‐deazapurine nucleobases were synthesized. Several potent antagonists of all four subtypes of adenosine receptors were identified by functional assay.

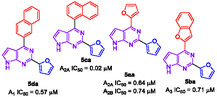

## 1 Introduction

Adenosine is a ubiquitous purine nucleoside with a range of complex biological functions. Besides being one of the canonical nucleosides forming nucleic acids, it is also involved in cellular energy transfer as a constituent of ATP and ADP. In signaling, adenosine contributes to both intracellular and extracellular regulation, acting via cyclic adenosine monophosphate (cAMP) and as an endogenous ligand of adenosine receptors (ARs), thereby controlling adenylyl cyclase activity and downstream cAMP levels.

ARs are G protein‐coupled receptors comprising four subtypes (A_1_, A_2A_, A_2B_, and A_3_) with distinct tissue distribution, signaling pathways, and physiological functions. In general, A_1_ and A_3_ ARs inhibit adenylate cyclase activity and thus reduce intracellular levels of cAMP, which promotes potassium efflux, leading to suppression of neuronal activity and relaxation of smooth muscles. A_2A_ and A_2B,_ on the other hand, stimulate adenylate cyclase and are involved in vasodilation and immune responses [1].

**FIGURE 1:**
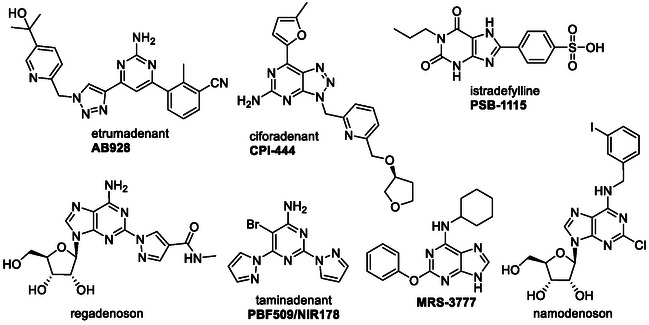
Selected adenosine receptor modulators. Approved istradefylline (A_2A_ antagonist) and regadenoson (A_2A_ agonist). Investigational oncology examples: etrumadenant/AB928 (A_2A_/A_2B_); development paused in 2025 after license return; ciforadenant/CPI‐444 (A_2A_); PBF‐1129 (A_2B_); taminadenant/PBF509/NIR178 (A_2A_); MRS‐3777; namodenoson (A_3_ agonist).

ARs are established therapeutic targets across various disease areas (neurological diseases, such as Parkinson´s disease, type 2 diabetes mellitus, cardiovascular disorders, and inflammation) [2]. In oncology, current strategies mainly focus on A_2A_ and A_2B_ antagonists to relieve adenosine‐mediated immunosuppression (e.g., etrumadenant/**AB928**, A_2A_/A_2B_ antagonist for the treatment of cell lung cancer, colorectal cancer and pancreatic adenocarcinoma [3]; **PBF‐1129** (structure undisclosed) [4], A_2B_ AR antagonist and taminadenant/**PBF‐509**, A_2A_ AR antagonist, both for treatment of nonsmall‐cell lung carcinoma [5]; ciforadenant/CPI‐444 [6], A_2A_/A_2B_ antagonist in advanced cancers; while A_3_ agonism (e.g., namodenoson) [7] has also advanced in specific indications. Despite the number of selective AR modulators in clinical trials, only two have been approved by the FDA: istradefylline (Nourianz), an A_2A_ AR antagonist for the treatment of Parkinson's disease [8], and A_2A_ AR agonist regadenoson (Lexiscan) [9] (Figure 1). From a medicinal chemistry perspective, AR antagonists are predominantly based on modified nucleobases, most notably xanthine derivatives (e.g., istradefylline, **PSB1115**) or 2,6‐disubstituted purine derivatives (e.g., **MRS3777**, an Eisai compound) [10]. While these chemotypes provided clinically relevant agents, their structural similarity to endogenous purines can limit their selectivity and tunability. Replacement of the N7 by carbon alters the electronic distribution and hydrogen‐bonding pattern, providing a rationale for exploring the 7‐deazapurine scaffold as a purine bioisostere in AR ligand design.

**FIGURE 2:**
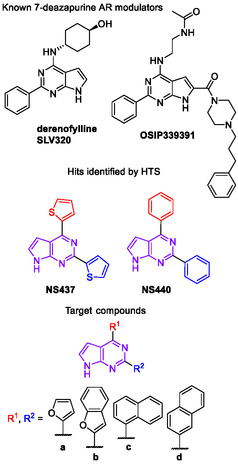
Known 7‐deazapurine AR modulators, 2,6‐disubstituted 7‐deazapurine hits from HTS and the target compound set for this study.

Although 7‐deazapurines are well‐known in medicinal chemistry [11], only a few 7‐deazapurine analogs are shown to act as AR modulators, such as **SLV320** (A_1_ antagonist with in vivo efficacy) [12] and **OSIP339391** (a potent, selective A_2B_ antagonist radioligand, Figure 2) [13]. Whilst there are examples of 2,6‐disubstituted 7‐deazapurines in the literature, their ARs activity has rarely been reported [14, 17]. This chemotype therefore remains largely unexplored, including in oncology, motivating our investigation of modified 7‐deazapurine (systematically 7*H*‐pyrrolo[2,3‐*d*]pyrimidine) nucleobases as AR modulators.

We performed high‐throughput screening (HTS) of an in‐house library (IMTM) containing >1000 modified purine and deazapurine derivatives and tested them first for their modulatory activity on all four ARs (A_1_, A_2A_, A_2B_, and A_3_) using an aequorin functional assay [18]. We identified several hits, all of them belonging to 2,6‐disubstituted 7‐deazapurines. Two most potent compounds were previously published [19] nucleobases bearing two aryl groups in positions 2 and 6; compound **NS437** substituted with two thiophen‐3‐yl groups and **NS440** bearing two phenyl substituents, both derivatives showed antagonistic activity against A_1_, A_2A_ and A_3_ ARs in the sub‐micromolar range (Figure 2). These results motivated us to focus on 7‐deazapurine nucleobases symmetrically and asymmetrically substituted by various (het)aryl groups and to perform structure–activity relationships (SAR) study to enable identification of potent and selective ARs antagonists with potential oncology relevance.

## 2 Results and Discussion

### 2.1 Chemistry

**SCHEME 1:**
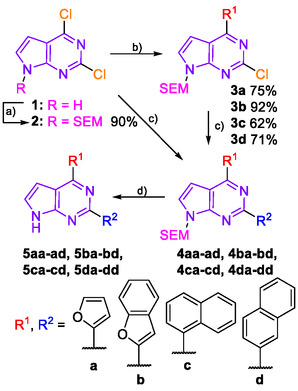
(a) SEM‐Cl (1.3 equiv.), NaH (1.3 equiv.), DMF, 22 °C, 2 h; (b) R‐B(OH)_2_ (1.1 equiv.), Na_2_CO_3_ (1.5 equiv.), Pd(OAc)_2_ (0.025 equiv.), TPPTS (0.06 equiv.), H_2_O/MeCN (1:1), 80 °C, 16 h; (c) R‐B(OH)_2_ (1.8–5 equiv.), Na_2_CO_3_ (3 equiv.), Pd(OAc)_2_ (0.05 equiv.), TPPTS (0.12 equiv.), H_2_O/MeCN (1:2), 100 °C, 16 h or Pd(PPh_3_)_4_ (0.1 equiv.), Na_2_CO_3_ (3 equiv.), H_2_O/DMF (1:3), 110 °C, 16 h; (d) (1) 0.1 m TFA, 22 °C, 1 h; (2) 0.02 m NH_4_OH, 22 °C, overnight.

A series of modified 2,6‐disubstituted 7‐deazapurine nucleobases were synthesized from commercially available 2,6‐dichloro‐7‐deazapurine (2,4‐dichloro‐7*H*‐pyrrolo[2,3‐*d*]pyrimidine) (**1**) by two successive Suzuki coupling reactions. Although there are some examples of successful cross‐coupling reactions on unprotected 6‐chloro‐7‐deazapurine [16, 19] or pyrido‐fused 7‐deazapurine [20], some other studies show that coupling reactions on unprotected chloronucleobases give lower yields or do not work at all [14, 15, 21, 22]. Therefore, we decided to protect the starting 2,6‐dichloro‐7‐deazapurine with the 2‐(trimethylsilyl)ethoxymethyl (SEM) protecting group, which is stable under coupling reaction conditions. The reaction of 2,6‐dichloro‐7‐deazapurine (**1**) with NaH and SEMCl in DMF gave the key intermediate protected nucleobase **2** (Scheme 1) in 90% yield [23]. The first Suzuki cross‐coupling reaction was done using aqueous conditions with water‐soluble ligand TPPTS, Pd(OAc)_2_, and Na_2_CO_3_ in a water and acetonitrile mixture and four different boronic acids [24, 25]. Similarly to our previous study on nucleosides [26], by using only 1.1 equivalent of boronic acid, the coupling was regioselective, and the intermediate nucleobases **3a–d** were synthesized in good 62%–92% yields. Only trace amounts of disubstituted products were observed in some cases and were easily removed during chromatography. The same conditions were then used for the second Suzuki cross‐coupling reaction; only the excess of boronic acid was increased up to 5 equivalents. However, in some cases, the reactivity was poor, so we tested different conditions and changed the catalyst to Pd(PPh_3_)_4_ in a mixture of water and DMF at 110 °C, which significantly improved the conversion and was thus used to synthesize **5bb**, **5cb**, **5db**, and **5dc.** As most of the other derivatives had already been prepared under the original conditions, they were not resynthesized using the optimized protocol. Unfortunately, unlike in the case of 2,6‐diaryl‐7‐deazapurine nucleosides [27], none of the reactions reached full conversion, even if we increased the reaction time and temperature or used a higher excess of boronic acid. This complicates the isolation as both the starting 6‐arylnucleobase **3** and target protected 2,6‐diaryl‐7‐deazapurines **4** are almost inseparable. Thus, the crude products **4aa–ad**, **4ba–bd**, **4ca–cd** and **4da–dd** were directly deprotected using TFA, giving the target 2,6‐disubstituted nucleobases **5aa–ad**, **5ba–bd**, **5ca–cd** and **5da–dd** in a broad range of yields (6%–93% yields over two steps, Scheme 1, Table 1).

**TABLE 1:** 2,6‐Disubstituted 7‐deazapurine nucleobases—yields of 2^nd^ Suzuki coupling and deprotection.

Compound	*R* ^1^	*R* ^2^	Yield^a^, %	Compound	*R* ^1^	*R* ^2^	Yield^a^, %
**5aa**	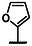	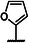	34	**5ca**	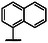	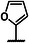	38
**5ab**	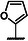	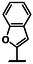	31	**5cb**	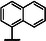	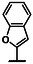	74
**5ac**	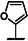	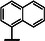	93	**5cc**	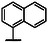	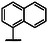	15
**5ad**	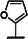	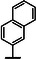	45	**5cd**	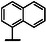	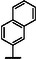	32
**5ba**	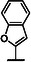	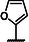	40	**5da**	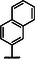	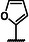	19
**5bb**	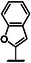	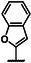	48	**5db**	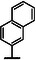	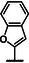	85
**5bc**	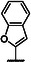	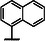	42	**5dc**	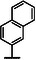	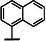	93
**5bd**	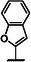	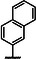	7	**5dd**	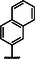	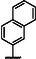	6^b^

a Over two stpng.

b Isolated as a by‐product during the first coupling.

**SCHEME 2:**
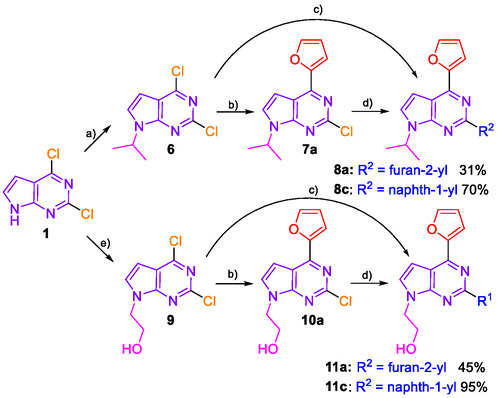
(a) Isopropyl iodide (1.5 equiv.), Cs_2_CO_3_ (1.2 equiv.), DMF, 22 °C, 2 h; (b) R‐B(OH)_2_ (1.1 equiv.), Na_2_CO_3_ (1.5 equiv.), Pd(OAc)_2_ (0.025 equiv.), TPPTS (0.06 equiv.), H_2_O/MeCN (1:1), 80 °C, 1 h; (c) R‐B(OH)_2_ (3 equiv.), Na_2_CO_3_ (1.5 equiv.), Pd(OAc)_2_ (0.025 equiv.), TPPTS (0.06 equiv.), H_2_O/MeCN (1:1), 80 °C, 16 h; (d) R‐B(OH)_2_ (3 equiv.), Na_2_CO_3_ (3 equiv.), Pd(OAc)_2_ (0.05 equiv.), TPPTS (0.12 equiv.), H_2_O/MeCN (1:1), 100 °C, 1 h; (e) 2‐chloroethanol (1.1 equiv.), Cs_2_CO_3_ (1.2 equiv.), DMF, 22 °C, 16 h.

Due to the high lipophilicity and flat structure, we were concerned about the aqueous solubility of these nucleobases, which are indeed poorly soluble in aqueous media (Table 2). To improve the solubility of our compounds and to extend the SAR and see if any modification in position 9 is allowed, we designed and prepared four compounds bearing either an isopropyl or a hydroxyethyl group in position 9 (Scheme 2).

**TABLE 2:** Solubility of the target nucleobases (5, 8, 11).

Solubility (PBS, pH 7.4), µM									
**5aa**	31	**5ba**	<4	**5ca**	8	**5da**	<0.4	**8aa**	27
**5ab**	<1	**5bb**	<1	**5cb**	<0.4	**5db**	<4	**8ac**	34
**5ac**	<0.8	**5bc**	<4	**5cc**	7	**5dc**	<0.1	**11aa**	>100
**5ad**	<1	**5bd**	<1	**5cd**	<0.1	**5dd**	1	**11ac**	18

Synthetic pathway started again from commercially available 2,6‐dichloro‐7‐deazapurine (**1**), which was deprotonated with Cs_2_CO_3_ and alkylated [28] by either isopropyl iodide or 2‐chloroethanol to obtain nucleobases **6** and **9** in 80% and 22% yield, respectively. The target nucleobases were again synthesized by two consecutive Suzuki coupling reactions. In the case of **7a** and **10a**, one Suzuki coupling reaction with an excess amount of boronic acid was performed, while for **13b** and **16b** two consecutive coupling reactions needed to be done (Scheme 2).

### 2.2 Biological Profiling of the Target Compounds

We measured the kinetic solubility of all the target nucleobases (**5, 8, 11**) in PBS buffer at pH 7.4. As we expected, all the N‐9 unsubstituted nucleobases showed very low solubility due to their high lipophilicity and flat nature. Introduction of a lipophilic isopropyl substituent had only a moderate effect on kinetic solubility, whilst substitution with a polar hydroxyethyl group significantly improved the solubility of both compounds **11a** and **11c** (Table 2).

### 2.3 Testing of Agonistic and Antagonistic Activity on ARs

These newly synthesized nucleobases (**5, 8, 11**) were evaluated for their ability to modulate the activity of ARs. Their agonistic and antagonistic effects on the four ARs were tested using a functional assay with an aequorin reporter cell system. A total of 20 compounds were screened in two stages: primary and secondary screening. In the primary screening, coelenterazine‐loaded cells were dispensed, and the agonistic activity of compounds was analyzed at a single concentration (10 μM) using the FLIPR Penta multimodal reader (Molecular Devices). Following a 15 min incubation at room temperature (RT), the antagonistic response, indicated by a reduction in luminescence, was measured immediately after adding the reference agonist at 80% of the maximal effective concentration (EC_80_). In the secondary screening, positive hits from the primary screening were tested concentration‐dependently. Raw data, expressed as the area under the curve (AUC), were normalized as percentages of the reference agonist response. The normalized responses were plotted against concentrations to determine EC_50_ values (for agonists) and IC_50_ values (for antagonists). Data were collected from two independent biological replicates, with each experiment performed in technical triplicate.

### 2.4 SAR and Molecular Interpretation of Receptor Selectivity

None of the compounds exhibited agonistic activity on any AR subtype. On the other hand, the screening for antagonistic activity several antagonists against all subtypes. Five compounds (Table 3) were active on A_1_, with **5da** being the most potent (IC_50_ = 0.57 μM). Interestingly, compound **5cc** showed selective activity only for A_1_ (IC_50_ = 9.44 μM). Six compounds were active on A_2A_, derivative **5ca** was the most active with IC_50_ = 0.02 μM, but only nucleobase **5cd** was a selective antagonist of A_2A_ with moderate potency (IC_50_ = 15.2 μM). Introduction of a 2‐hydroxyethyl group into position 9 led to a decrease in antagonistic activity against A_2A_, but the N‐9 substituted derivative **11aa** was still a very potent antagonist with IC_50_ in the sub‐micromolar range (compounds **5aa** and **11aa** in Table 3). We identified only two antagonists of A_2B_, both compounds bearing a furan‐2‐yl substituent in position 6 and either also a furan‐2‐yl in position 2 (**5aa** with IC_50_ = 0.74 μM) or a more bulky naphthalen‐2‐yl (**5ad** IC_50_ = 9.61 μM). Most of the tested compounds showed antagonistic activity on A_3_ subtype, several of them are not only potent, but also very selective with sub‐micromolar IC_50_s on A_3_ and no activity against the other 3 subtypes (**5ab**, **5ac**, **5ba**, **5bc**).

**TABLE 3:** Antagonistic activity of the target nucleobases.

**IC**_**50**_, **µM**							
Compd	A_1_	A_2A_	A_2B_	A_3_	clogP	TPSA	MW
**5aa**	>50	0.64	0.74	>50	3.1	68	251
**5ab**	>0	>50	>50	1.97	4.1	68	301
**5ac**	>50	>50	>50	0.30	4.9	55	311
**5ad**	>50	15.2	9.6	0.35	4.9	55	311
**5ba**	>50	>50	>50	0.71	4.1	68	301
**5bb**	>50	>50	>50	43	5.0	68	351
**5bc**	>50	>50	>50	0.51	5.9	55	361
**5bd**	>50	>50	>50	8.7	5.9	55	361
**5ca**	5.8	0.02	>50	>50	4.9	55	311
**5cb**	>50	49	>50	>50	5.9	55	361
**5cc**	9.44	>50	>50	>50	6.5	42	371
**5cd**	>50	15.2	>50	>50	6.5	42	371
**5da**	0.57	>50	>50	2.1	4.9	55	311
**5db**	>50	>50	>50	>50	5.9	55	361
**5dc**	>50	>50	>50	3.4	6.5	42	371
**5dd**	>50	>50	>50	15	6.5	42	371
**8aa**	30	>50	>50	39	4.1	57	293
**8ac**	>50	>50	>50	>50	5.9	44	353
**11aa**	34	0.94	>50	>50	2.8	77	295
**11ac**	>50	>50	>50	>50	4.5	64	355

The SAR analysis (Tables 3 and 4) revealed a pronounced receptor subtype‐dependent effect of the (hetero)aryl substituents and was strongly influenced by the size and spatial orientation of both substituents. A_1_ receptor affinity was observed only for a limited number of analogs and appeared to require an asymmetric substitution pattern, combining a bulky hydrophobic group at C6 with a compact 2‐furyl substituent at C2. This was most evident for compound **5da**, bearing a 2‐naphthyl group at C6 and a 2‐furyl group at C2, which was the most potent A_1_ ligand in the series with IC_50_ = 0.57 µM.

**TABLE 4:** Main SAR observations.

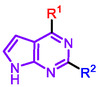	
Structural feature	Observed effect
*R* ^2^ = furan‐2‐yl	Important for A_1_/A_2A_/A_2B_ activity
*R* ^2^ = naphthyl	A_3_ activity, especially with C6 2‐furyl or benzofuran‐2‐yl
*R* ^1^ = naphth‐1‐yl + *R* ^2^ = furan‐2‐yl	Associated with A_1_ affinity
*R* ^1^ = naphthalen‐2‐yl	Favors A_1_
Symmetric bulky substitution	Generally unfavorable
Asymmetric heteroaryl/aryl substitution	Often improves potency and subtype selectivity
*R* ^1^ = 2‐furyl + *R* ^2^ = 2‐furyl	Favors A_2A_/A_2B_ dual activity

High A_2A_ affinity strongly depended on the presence of a small 2‐furyl substituent at C2, as demonstrated by compounds **5aa** (IC_50_ = 0.64 µM) and especially **5ca** (IC_50_ = 0.02 µM). In contrast, replacement of the C2 2‐furyl group with bulkier aromatic substituents such as benzofuran‐2‐yl or naphthyl generally resulted in a marked loss of activity, indicating limited steric tolerance at this position. Substitution at C6 had a modulatory effect on potency. While 2‐furyl substitution was compatible with A_2A_ activity, introduction of a 1‐naphthyl group at C6 significantly enhanced affinity, as observed for compound **5ca**. Conversely, 2‐naphthyl substitution at C6 abolished activity, suggesting that both substituent size and orientation are important for receptor recognition.

In the case of the A_2B_ receptor, significant activity was observed only for two compounds bearing a 2‐furyl substituent at C6, particularly compound **5aa**, which exhibited submicromolar potency (IC_50_ = 0.74 µM).

The A_3_ receptor displayed a markedly different SAR profile compared to A_1_, A_2A_, and A_2B_ receptors. In contrast to A_2A_/A_2B_ receptors, A_3_ affinity was strongly enhanced by larger hydrophobic aromatic substituents at C2, particularly 1‐naphthyl and 2‐naphthyl groups. The most potent compounds in the series were **5ac** (IC_50_ = 0.30 µM) and **5ad** (IC_50_ = 0.35 µM), both containing a 2‐furyl substituent at C6 combined with naphthyl substitution at C2. Similarly, compound **5bc**, bearing benzofuran‐2‐yl at C6 and 1‐naphthyl at C2, also exhibited potent A_3_ affinity (IC_50_ = 0.51 µM). These results indicate that the A_3_ receptor preferentially accommodates extended hydrophobic aromatic systems at C2. Comparison between 1‐naphthyl and 2‐naphthyl substituents further suggests that the spatial orientation of the aromatic system contributes to receptor recognition. Although both substituents supported A_3_ activity, 1‐naphthyl derivatives generally displayed more consistent potency across different C6 substitution patterns (**5bc** vs. **5bd**; **5ac** vs. **5ad**). The nature of the C6 substituent also played a major role. Compounds bearing 2‐furyl and benzofuran‐2‐yl were generally more active, whereas bulky aromatic substitution at C6, particularly 1‐naphthyl, was detrimental. This is clearly demonstrated by the near‐complete loss of A_3_ affinity across the **5c** series. Therefore, the A_3_ receptor appears to favor an asymmetric arrangement combining a heteroaryl substituent at C6 with a larger hydrophobic aromatic group at C2. The N9 substitution in compounds **8aa**, **8ac**, **11aa**, and **11ac** led to significant loss of activity; only **11aa** retained submicromolar A2A activity (but lost A2B activity completely). This is likely caused by masking the hydrogen bond donor and thus changing the binding mode. The most important trends are summarized in Table 4.

**FIGURE 3:**
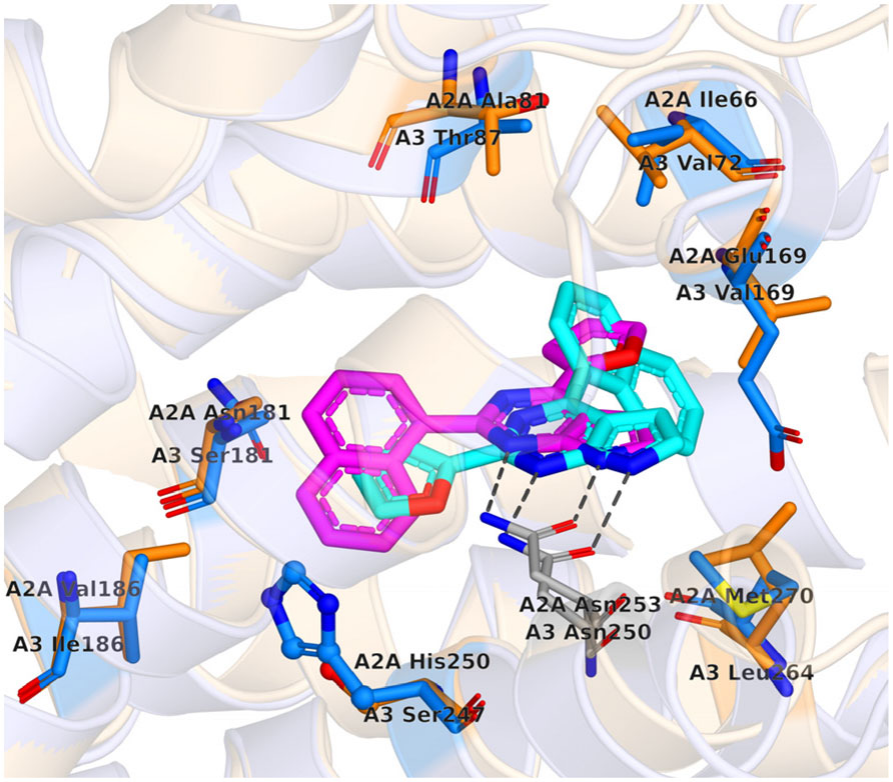
Predicted poses for **5ca** (cyan) and **5ac** (magenta) into A_2A_ (orange) and A_3_ (marine) receptor subtypes, respectively.

To investigate possible structural origins of these SAR trends, receptor–ligand complexes were modeled using Boltz‐2, a biomolecular co‐folding method that predicts ligand‐binding poses and associated affinity estimates [29]. Boltz‐2 was selected because it produced the most internally consistent binding modes across the four receptor subtypes. In the predicted complexes, ligands generally adopted a conserved orientation in which the heterocyclic core formed two hydrogen bonds with the conserved Asn residue at position 6.55 (Figure 3). Similar interactions with Asn^6.55^ have been observed for structurally characterized AR agonists and antagonists and are recognized as a central feature of orthosteric ligand binding [30, 31]. Because this interaction was retained across receptor subtypes and across both active and inactive analogs, it is unlikely to be the principal determinant of selectivity. Instead, selectivity appears to be governed mainly by differences in the shape and volume of adjacent subpockets.

A notable subtype‐dependent difference occurs at position 6.52. A_1_, A_2A_, and A_2B_ contain a relatively bulky His250 at this position, whereas A_3_ contains the smaller Ser247^6.52^. Replacement of histidine by serine creates additional space in the A_3_ orthosteric pocket, allowing accommodation of larger substituents directed toward this region. This difference provides a plausible explanation for the loss of A_1_, A_2A_, and A_2B_ activity, together with retention of A_3_ activity, for compounds bearing a bulky substituent at position 2, including **5ab**, **5ac**, and **5ad** (Figure 3).

The additional space created by Ser^6.52^ does not, however, necessarily favor every ligand. When a substituent does not occupy this region efficiently, the remaining cavity may be hydrated. For ligands presenting a hydrophobic group toward this incompletely occupied subpocket, unfavorable desolvation or poorly compensated water‐mediated interactions may reduce binding affinity. This may contribute to the weak or absent A_3_ activity of compounds such as **5aa**, **5ca**, and **5da** (Table 3). Because explicit water molecules and their thermodynamic contributions were not modeled directly, this explanation should be regarded as a structural hypothesis rather than a definitive mechanism.

A second relevant difference is present on the opposite side of the orthosteric site at position 2.64. The A_3_ receptor contains Val72^2.64^, whereas the other AR subtypes contain a bulkier isoleucine, represented by Ile66^2.64^ in the A_2A_ sequence. The smaller valine side chain further increases the available volume in the A_3_ binding site and may permit somewhat larger substituents at position 6. Together, Ser^6.52^ and Val^2.64^ make the A_3_ orthosteric pocket more tolerant of bulky substituents at either position 2 or position 6 than the corresponding pockets of A_1_, A_2A_, and A_2B_. Nevertheless, simultaneous introduction of bulky substituents at both positions was generally unfavorable for all four receptor subtypes. Compounds such as **5bb**, **5cc**, **5cd**, **5db**, and **5dd** showed only weak or no activity despite forming apparently plausible contacts in the predicted complexes. This suggests that excessive steric bulk can cause overfilling of the binding cavity, suboptimal orientation of the conserved heterocyclic core, incomplete burial of one aromatic substituent, or an increased conformational and desolvation penalty. Thus, the number of predicted receptor‐ligand contacts alone is insufficient to explain potency.

Overall, the experimental SAR and molecular models indicate that the conserved interaction with Asn^6.55^ anchors the common ligand scaffold, whereas subtype selectivity is primarily determined by the orientation of the substituents toward receptor‐specific subpockets. The smaller Ser^6.52^ and Val^2.64^ residues make A_3_ more tolerant of larger substituents, while A_1_, A_2A_, and particularly A_2B_ favor more compact or precisely oriented analogs. The marked activity switches among regioisomeric compounds, especially **5ac/5ca** and **5ad/5da**, further demonstrate that the direction in which a bulky substituent projects into the binding site is more important than its size alone.

### 2.5 Cytotoxicity

The cytotoxic activity of newly synthesized nucleobases was evaluated using the MTS assay on a panel of human cancer cell lines: A549 (lung cancer), CCRF‐CEM (acute T‐lymphoblastic leukemia), HCT116 and HCT116p53^‐^ (colon carcinoma, parental and p53 deficient), K562 (chronic myelogenous leukemia), U2OS (bone osteosarcoma) and two nonmalignant fibroblast cell lines (BJ and MRC‐5). Cytotoxic activities are presented as the IC_50_ values after 72 h treatment, shown in Table 4. Data are expressed as the mean ± SD (μM) of the IC_50_ values from three independent experiments.

Compounds **5ba** and **5db** showed micromolar activity only against leukemia cell line CCRF‐CEM, and no toxicity to all the other tested cell lines, including nonmalignant fibroblasts (BJ and MRC‐5). Nucleobases **5bc** and **5dc** are the most cytotoxic compounds in this study with sub‐micromolar and low single‐digit micromolar IC_50_ values across all tested cell lines, unfortunately including nontumor BJ and MRC‐5. Compound **5cb** was broadly cytotoxic and inactive on BJ; however, with no selectivity against MRC‐5. Compounds **5aa**, **5ac**, **5bb**, **5cc**, and **5da** were completely inactive across all tested cell lines (Table 5). The antagonistic activity at the A_3_ and A_2A_ receptors did not correlate with the cytotoxic activity of these nucleobases, suggesting that a distinct, receptor‐independent mechanism underlies their cytotoxicity. These findings agree with previous reports showing that the anticancer effect of antagonistic AR targeting is mediated largely through the tumor immune microenvironment rather than through direct cytotoxicity [32].

**TABLE 5:** Cytotoxic activities of the title nucleobases 5, 8, 11.

MTS, IC_50_, µM								
Compd	BJ	MRC‐5	A549	CCRF‐CEM	HCT116	HCT116p53‐	K562	U2OS
**5aa**	>50	>50	>50	>50	>50	>50	>50	>50
**5ab**	10	12	>50	5.3	35	>50	>50	33
**5ac**	>50	>50	>50	>50	>50	>50	>50	>50
**5ad**	>50	>50	>50	36	>50	>50	>50	28
**5ba**	>50	>50	>50	5.3	>50	>50	>50	>50
**5bb**	>50	>50	>50	>50	>50	>50	>50	>50
**5bc**	3.1	1.5	0.91	0.51	0.78	0.99	0.52	0.83
**5bd**	>50	>50	>50	44.21	>50	>50	>50	>50
**5ca**	>50	>50	>50	37	>50	>50	>50	>50
**5cb**	>50	2.4	2.4	1.4	8.3	9.0	10	7.2
**5cc**	>50	>50	>50	>50	>50	>50	>50	>50
**5cd**	>50	>50	>50	25	>50	>50	>50	>50
**5da**	>50	>50	>50	>50	>50	>50	>50	>50
**5db**	>50	>50	>50	2.33	>50	>50	>50	>50
**5dc**	28	33	8.0	2.0	2.9	2.5	1.3	2.8
**5dd**	16	9.7	26	35	34	17	37	30
**8aa**	>50	>50	>50	31	>50	>50	33	>50
**8ac**	>50	>50	>50	43	>50	>50	>50	>50
**11aa**	>50	>50	>50	39	>50	>50	>50	44
**11a** **c**	>50	>50	>50	28	>50	>50	37	39

### 2.6 Cell Cycle Analysis

Compounds with activity below 10 µM on the CCRF‐CEM cell line were further analyzed by flow cytometry. We analyzed the cell cycle profile of CCRF‐CEM cells treated with compounds for 24 h. The experiment was performed at 1× and 5× IC_50_ concentrations to observe the effect on the cell cycle.

**FIGURE 4:**
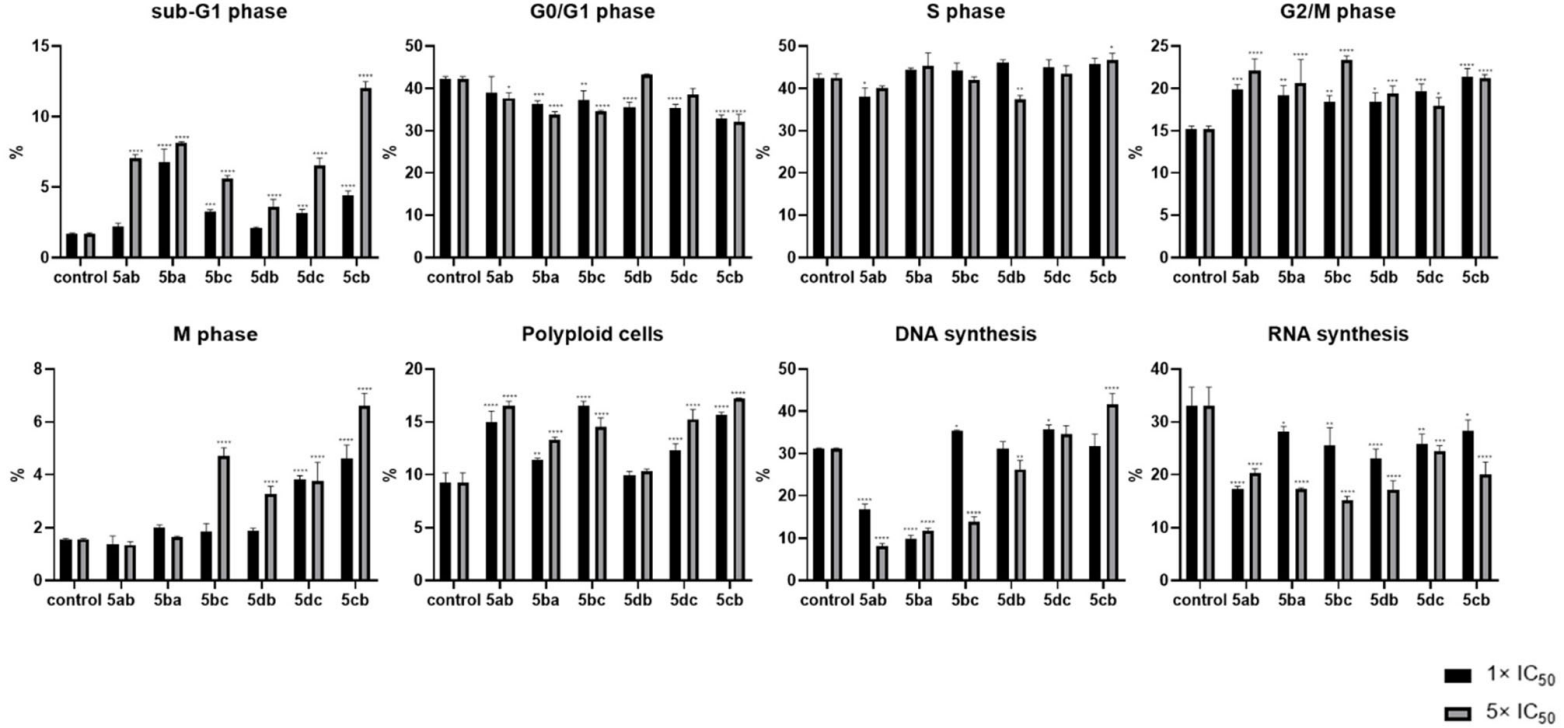
Cell cycle analysis of the most active derivatives.

Flow cytometry analysis (Figure 4) revealed that the tested compounds affected cell cycle distribution and cellular proliferation. The most prominent changes included an increase in the sub‐G1 population, accumulation of cells in the G2/M and M phases, and an increased proportion of polyploid cells. These effects were most pronounced for compound **5cb**, particularly at 5 × IC_50_, suggesting strong induction of DNA damage‐associated cell death and/or mitotic disruption. Compounds **5bc** and **5dc** also showed noticeable effects, especially on mitotic progression and polyploidy. In addition, most compounds reduced RNA synthesis, while DNA synthesis was markedly decreased mainly after treatment with **5ab**, **5ba**, and **5bc**. Overall, the data suggest that the compounds impair cell cycle progression, reduce proliferative activity, and in some cases promote accumulation of damaged or polyploid cells. Statistical analysis was performed using two‐way ANOVA followed by the Dunnett test. The data represent the mean values obtained from three replicates. Differences were considered statistically significant at ^∗^ p<0.05, ^∗∗^ p<0.01, ^∗∗∗^ p<0.001, and ^∗∗∗∗^ p<0.0001 compared to the vehicle control.

### 2.7 Annexin V Binding Assay

**FIGURE 5:**
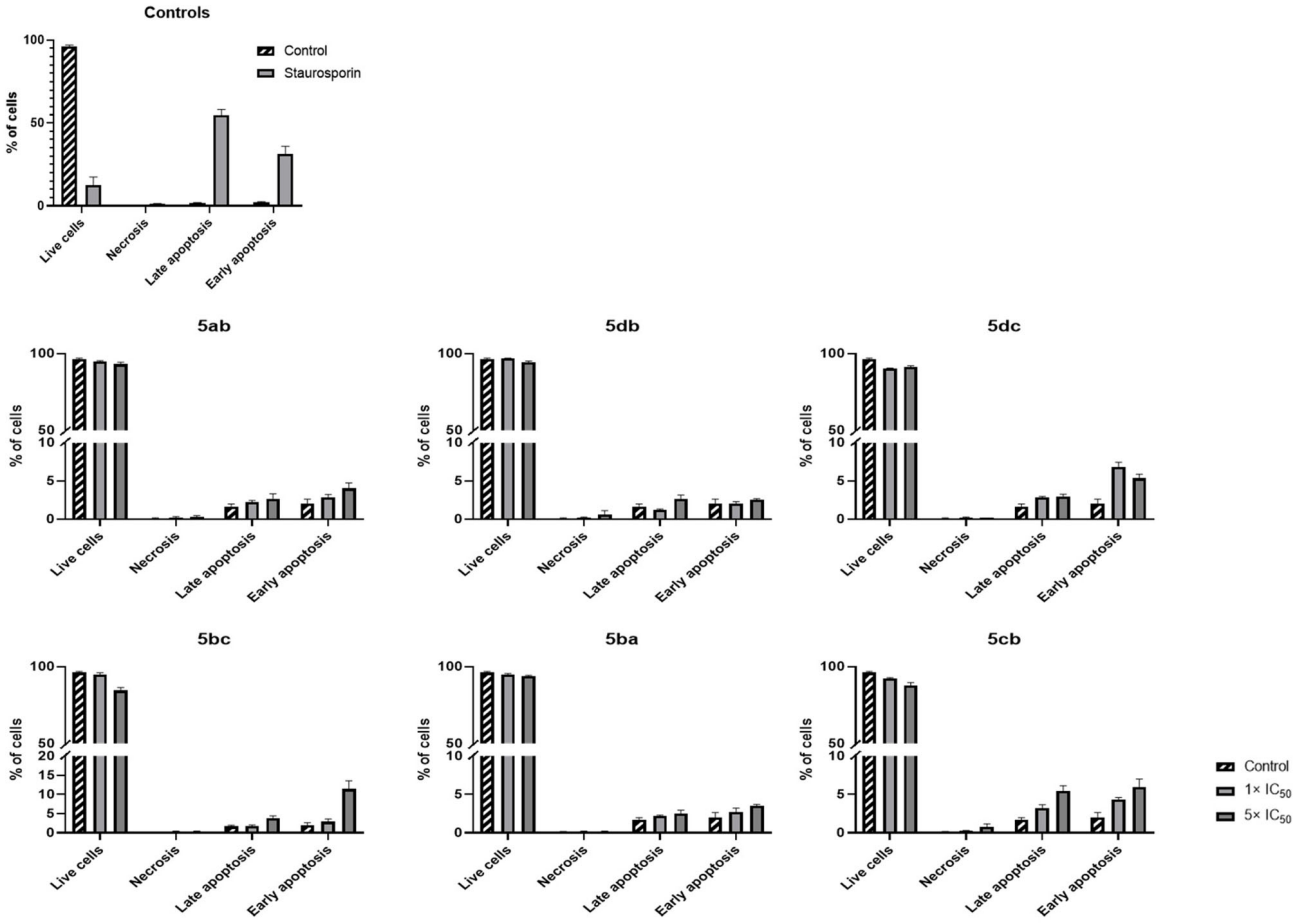
Annexin V binding assay of the most active compounds.

Annexin V/PI staining (Figure 5) revealed that the tested compounds (same as for cell cycle analysis) did not induce pronounced necrotic cell death. In most samples, the proportion of viable cells remained high, and only a moderate increase in apoptotic populations was observed. The most prominent effect was detected for compound **5bc**, particularly at 5 × IC_50_, where reduced viability was accompanied by an increase in early apoptotic cells. A milder pro‐apoptotic effect was also observed for compounds **5cb** and **5dc**. Compared with the positive control staurosporine, however, the apoptotic effect of all tested compounds was markedly weaker.

## 3 Conclusions

This work identifies a novel type of nonxanthine 7‐deazapurine nucleobase bearing (het)aryl groups in position 2 and 6 with antagonistic activity against all four subtypes of ARs. Compound **5da** is a submicromolar antagonist of A_1_, and compound **5ca** is the most potent antagonist of A_2A_ with IC_50_ = 20 nM. Derivative **5aa**, substituted with only small furan‐yl groups, is a sub‐micromolar and selective antagonist of both A_2A_ and A_2B_ subtypes. Several compounds showed potent and very selective sub‐micromolar antagonistic activity on A_3_, notably derivatives **5ac** and **5bc** with IC_50_ 0.30 µM and 0.51 µM, respectively. None of the tested compounds showed any agonistic activity. Interestingly, N9 substitution markedly improved aqueous solubility while retaining activity.

Although the cytotoxic activity screening identified several potent compounds (especially **5bc** and **5dc**), they did not show any selectivity between cancer and nonmalignant cell lines. Despite that, the SAR study brings valuable information about the size and orientation of both (het)aryl groups needed to prepare potent AR antagonists. Whilst only furan‐2‐yl is tolerated in position 2, variation of 6‐(het)aryl groups can change the selectivities between each AR subtype. These findings can be used for further design of AR subtype‐selective antagonists with improved ADME properties.

## 4 Experimental Section

### 4.1 General Remarks

Unless otherwise stated, all cross‐coupling reactions were carried out under an argon atmosphere. 2,4‐Dichloro‐7*H*‐pyrrolo[2,3‐*d*]pyrimidine was obtained from commercial suppliers. Thin‐layer chromatography (TLC) was performed on *Merck silica gel 60 F‐254 aluminum sheets*. Spots were visualized by UV light (λ_max_ = 254 nm or 366 nm). High‐performance flash chromatography (HPFC) was conducted with a *Combi Flash R*_*f*_ instrument from *Teledyne Isco Inc*. using SiO_2_ (particle size 0.040–0.063 mm, 230–400 mesh) from *Fluorochem* in refillable flash columns or *HP C18 Redi Sep R*_*f*_ *gold* flash columns with the solvent gradient indicated in the corresponding procedures. Reactions in the microwave reactor were performed in the Biotage Initiator Microwave Reactor. ^1^H and ^13^C{^1^H} NMR spectra were measured on a *Bruker Avance III 500 MHz* spectrometer at 25 °C in CDCl_3_ referenced to the residual solvent signal (δ_H_ = 7.26 ppm, δ_C_ = 77.16 ppm) or in deuterated dimethyl sulfoxide (DMSO‐*d*_6_) referenced to the residual solvent signal (δ_H_ = 2.50 ppm, δ_C_ = 39.52 ppm). Coupling constants (*J*) are given in Hz and the multiplets are described as s (singlet), d (doublet), t (triplet), q (quartet), m (multiplet), and b (broad). ^13^C{^1^H} NMR experiments are broadband proton‐decoupled and were performed using an APT pulse sequence. Two‐dimensional correlation spectroscopy (COSY), heteronuclear single quantum coherence (HSQC), and heteronuclear multiple bond correlation (HMBC) experiments were used to assign the ^1^H and ^13^C NMR signals where required. High‐resolution mass spectrometry (HR‐MS) was measured by the MS‐Service at IOCB Prague on an *LTQ Orbitrap XL* instrument from Thermo Fisher Scientific using electrospray ionization (ESI). The purity of the final nucleobases (>95%) was confirmed by UPLC‐MS on an *Agilent 1260 Infinity II LC* system with an *Agilent 1260 Photodiode Array Detector* using an *Acquity Premier BEH C8 1.7 µm VanGuard FIT* (2.1 × 100 mm). Cytotoxic activity screening was performed as described previously [33, 35].

### 4.2 General Procedure A: Suzuki Cross‐Coupling Reaction in Position 6

H_2_O/MeCN (1:1) was added through a septum to an argon‐purged vial containing nucleobase intermediate (1 equiv.), corresponding boronic acid (1.1 equiv.), Na_2_CO_3_ (1.5 equiv.), TPPTS (0.06 equiv.) and Pd(OAc)_2_ (0.025 equiv.). The mixture was heated at 80 °C for 16 h. Solvent was evaporated and the crude mixture was purified by HPFC.

### 4.3 General Procedure B: Suzuki Cross‐Coupling Reaction in Position 2

H_2_O/MeCN (1:1) was added through a septum to an argon‐purged vial containing intermediate nucleobase (1 equiv.), corresponding boronic acid (1.8–5 equiv.), Na_2_CO_3_ (3 equiv.), TPPTS (0.12 equiv.) and Pd(OAc)_2_ (0.05 equiv.). The mixture was heated at 100 °C for 16 h. Solvent was evaporated and the crude mixture was purified by HPFC.

### 4.4 General Procedure C: Suzuki Cross‐Coupling Reaction in Position 2

H_2_O/DMF (1:3) was added through a septum to an argon‐purged vial containing protected nucleobase intermediate (1 equiv.), corresponding boronic acid (3 equiv.), Na_2_CO_3_ (3 equiv.), and Pd(PPh_3_)_4_ (0.10 equiv). The mixture was heated to 110 °C for 16 h. Solvent was evaporated and the crude mixture was purified by HPFC.

### 4.5 General Procedure D: Deprotection of SEM Group

Protected nucleobase (1  equiv.) was treated with 0.1 M TFA for 1 h; after that, it was co‐evaporated with MeOH (3 times) and neutralized by aq. NaHCO_3_. The mixture was treated with aqueous ammonia (30% aq.) and stirred for 16 h. Solvent was evaporated and the crude mixture was purified by RP‐HPFC.

### 4.6 2‐Chloro‐4‐(furan‐2‐yl)‐7‐{[2‐(trimethylsilyl)ethoxy]methyl}‐7*H*‐pyrrolo[2,3‐*d*]pyrimidine (3a)


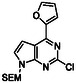


GP A starting from compound **2** (1.13 g, 3.56 mmol) and 2‐furylboronic acid (438.5 mg, 3.92 mmol). HPFC (SiO_2_, cHex/EtOAc 1:0 → 9:1) gave **3a** (929.6 mg, 75%) as a white solid. ^1^H NMR (500 MHz, DMSO‐d_6_): –0.10 (s, 9H, CH_3_Si); 0.83 (m, 2H, OCH_2_C**H**_**2**_Si); 3.53 (m, 2H, OC**H**_**2**_CH_2_Si); 5.58 (NCH_2_O); 6.81 (dd, 1H, *J*_4,3_ = 3.5 Hz, *J*_4,5_ = 1.8 Hz, H‐4‐furyl); 7.07 (d, 1H, *J*_5,6_ = 3.6 Hz, H‐5); 7.53 (dd, 1H, *J*_3,4_ = 3.5 Hz, *J*_3,5_ = 0.7 Hz, H‐3‐furyl); 7.82 (d, 1H, *J*_6,5_ = 3.6 Hz, H‐6); 8.10 (bdd, 1H, *J*_5,4_ = 1.8 Hz, *J*_5,3_ = 0.7 Hz, H‐5‐furyl); ^13^C NMR (125.7 MHz, DMSO‐d_6_): –1.4 (CH_3_Si); 17.1 (OCH_2_ **C**H_2_Si); 65.9 (O**C**H_2_CH_2_Si); 72.6 (OCH_2_N); 101.4 (C‐5); 111.3 (C‐4a); 113.1 (CH‐4‐furyl); 115.1 (CH‐3‐furyl); 131.9 (CH‐6); 147.3 (CH‐5‐furyl); 148.1 (C‐4); 151.1 (C‐2‐furyl); 152.6 (C‐2); 153.5 (C‐7a). HR‐ESI‐MS: *found*: 350.1088 ([M + H]^+^, calcd for C_16_H_21_ClO_2_N_3_Si^+^: 350.1086).

### 4.7 2,4‐Di(furan‐2‐yl)‐7‐{[2‐(trimethylsilyl)ethoxy]methyl}‐7*H*‐pyrrolo[2,3‐*d*]pyrimidine (4aa)


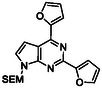


GP B starting from compound **3a** (89.8 mg, 0.26 mmol) and 2‐furylboronic acid (57.4 mg, 0.51 mmol). HPFC (SiO_2_, cHex/EtOAc 1:0 → 9:1) gave crude product **4aa** as a yellow solid (43 mg), which was used directly for the next step.

### 4.8 2,4‐Bis(furan‐2‐yl)‐7*H*‐pyrrolo[2,3‐*d*]pyrimidine (5aa)


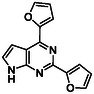


GP D starting from crude **4aa** (97.2 mg, 0.26 mmol). RP‐HPFC (C‐18, H_2_O/MeOH 0 → 100%) gave **5aa** (50.1 mg, 34%, over two steps) as a dark yellow solid. ^1^H NMR (500.0 MHz, DMSO‐d_6_): 6.68 (dd, 1H, *J*_4,3_ = 3.4 Hz, *J*_4,5_ = 1.8 Hz, H‐4‐furyl‐*A*); 6.80 (dd, 1H, *J*_4,3_ = 3.5 Hz, *J*_4,5_ = 1.8 Hz, H‐4‐furyl‐*B*); 6.95 (d, 1H, *J*_5,6_ = 3.5 Hz, H‐5); 7.25 (dd, 1H, *J*_3,4_ = 3.4 Hz, *J*_3,5_ = 0.9 Hz, H‐3‐furyl‐*A*); 7.51 (dd, 1H, *J*_3,4_ = 3.5 Hz, *J*_3,5_ = 0.7 Hz, H‐3‐furyl‐*B*); 7.63 (d, 1H, *J*_6,5_ = 3.5 Hz, H‐6); 7.92 (dd, 1H, *J*_5,4_ = 1.8 Hz, *J*_5,3_ = 0.9 Hz, H‐5‐furyl‐*A*); 8.06 (dd, 1H, *J*_5,4_ = 1.8 Hz, *J*_5,3_ = 0.7 Hz, H‐5‐furyl‐*B*); 12.25 (vbs, 1H, NH); ^13^C NMR (125.7 MHz, DMSO‐d_6_): 100.7 (C‐5); 110.4 (C‐4a); 111.3 (CH‐3‐furyl‐*A*); 112.1 (CH‐4‐furyl‐*A*); 112.6 (CH‐4‐furyl‐*B*); 113.1 (CH‐3‐furyl‐*B*); 128.2 (CH‐6); 144.4 (CH‐5‐furyl‐*A*); 146.1 (CH‐5‐furyl‐*B*); 146.3 (C‐4); 150.3 (C‐2); 152.1 (C‐2‐furyl‐*B*); 152.9 (C‐2‐furyl‐*A*); 153.2 (C‐7a). HR‐ESI‐MS: *found*: 251.0693 ([M]^+^, calcd for C_14_H_9_O_2_N_3_^+^: 251.0689).

### 4.9 2,4‐Dichloro‐7‐*iso*propyl‐7*H*‐pyrrolo[2,3‐*d*]pyrimidine (6)


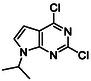


2,4‐Dichloro‐7*H*‐pyrrolo[2,3‐*d*]pyrimidine (**1**) (1 g, 5.32 mmol) was dissolved in DMF (8 mL), and Cs_2_CO_3_ (2.08 mg, 6.38 mmol) was added. The mixture was stirred for 30 min, and then *iso*propyl iodide (0.80 mL, 7.98 mmol) was slowly added dropwise. The mixture was stirred at rt for 2 h, treated with water and extracted with EtOAc. The combined organic layers were dried over anhydrous Na_2_SO_4_. HPFC purification (SiO_2_, cHex/EtOAc 1:0 → 9:1) gave **6** (0.98 g, 80%) as a colorless oil. ^1^H NMR spectrum is in agreement with the literature [36].

### 4.10 2‐Chloro‐4‐(furan‐2‐yl)‐7‐*iso*propyl‐7*H*‐pyrrolo[2,3‐*d*]pyrimidine (7a)


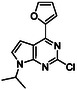


GP A starting from compound **6** (190.8 mg, 0.82 mmol) and 2‐furylboronic acid (101.2 mg, 0.90 mmol). HPFC (SiO_2_, cHex/EtOAc 1:0 → 9:1) and lyophilization from H_2_O/*t*‐BuOH gave **7a** (141.9 mg, 66%) as a pale‐yellow powder. ^1^H NMR (500 MHz, DMSO‐d_6_): 1.47 (d, 6H, *J*_*CH3*,*CH*_ = 6.8 Hz, (C**H**_**3**_)_2_CH); 4.97 (sept, 6H, *J*_*CH*,*CH3*_ = 6.8 Hz, (CH_3_)_2_C**H**); 6.80 (dd, 1H, *J*_4,3_ = 3.5 Hz, *J*_4,5_ = 1.7 Hz, H‐4‐furyl); 7.03 (d, 1H, *J*_5,6_ = 3.7 Hz, H‐5); 7.51 (dd, 1H, *J*_3,4_ = 3.5 Hz, *J*_3,5_ = 0.8 Hz, H‐3‐furyl); 7.87 (d, 1H, *J*_6,5_ = 3.7 Hz, H‐6); 8.08 (dd, 1H, *J*_5,4_ = 1.7 Hz, *J*_5,3_ = 0.8 Hz, H‐5‐furyl); ^13^C NMR (125.7 MHz, DMSO‐d_6_): 22.2 ((**C**H_3_)_2_CH); 46.2 ((CH_3_)_2_ **C**H); 105.7 (CH‐5); 111.3 (C‐4a); 113.0 (CH‐4‐furyl); 114.7 (CH‐3‐furyl); 128.6 (CH‐6); 147.0 (CH‐5‐furyl); 147.7 (C‐2); 151.3 (C‐2‐furyl); 151.01 and 152.91 (C‐4,7a). HR‐ESI‐MS: *found*: 262.0743 ([M + H]^+^, calcd for C_13_H_13_ON_3_Cl^+^: 262.0742); HR‐ESI‐MS: *found*: 284.0562 ([M + Na]^+^, calcd for C_13_H_12_ON_3_ClNa^+^: 284.0561).

### 4.11 2,4‐Di(furan‐2‐yl)‐7‐*iso*propyl‐7*H*‐pyrrolo[2,3‐*d*]pyrimidine (8aa)


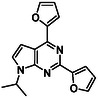


GP B starting from compound **7a** (102.0 mg, 0.44 mmol) and 2‐furylboronic acid (147.5 mg, 1.32 mmol). HPFC (SiO_2_, cHex/EtOAc 1:0 → 9:1) and lyophilization from H_2_O/*t*‐BuOH gave **8aa** (40.1 mg, 31%) as a pale‐yellow powder. ^1^H NMR (500 MHz, DMSO‐d_6_): 1.51 (d, 6H, *J*_*CH3*, *CH*_ = 6.8 Hz, (C**H**_**3**_)_2_CH); 5.12 (sept, 6H, *J*_*CH*, *CH3*_ = 6.8 Hz, (CH_3_)_2_C**H**); 6.68 (dd, 1H, *J*_4,3_ = 3.4 Hz, *J*_4,5_ = 1.8 Hz, H‐4‐furyl‐*A*); 6.80 (dd, 1H, *J*_4,3_ = 3.5 Hz, *J*_4,5_ = 1.8 Hz, H‐4‐furyl‐*B*); 6.99 (d, 1H, *J*_5,6_ = 3.6 Hz, H‐5); 7.30 (dd, 1H, *J*_3,4_ = 3.4 Hz, *J*_3,5_ = 0.9 Hz, H‐3‐furyl‐*A*); 7.52 (dd, 1H, *J*_3,4_ = 3.5 Hz, *J*_3,5_ = 0.8 Hz, H‐3‐furyl‐*B*); 7.83 (d, 1H, *J*_6,5_ = 3.6 Hz, H‐6); 7.88 (dd, 1H, *J*_5,4_ = 1.8 Hz, *J*_5,3_ = 0.9 Hz, H‐5‐furyl‐*A*); 8.06 (dd, 1H, *J*_5,4_ = 1.8 Hz, *J*_5,3_ = 0.7 Hz, H‐5‐furyl‐*B*); ^13^C NMR (125.7 MHz, DMSO‐d_6_): 22.4 ((**C**H_3_)_2_CH); 45.5 ((CH_3_)_2_ **C**H); 100.5 (CH‐5); 110.7 (C‐4a); 111.6 (CH‐3‐furyl‐*A*); 112.2 (CH‐4‐furyl‐*A*); 112.6 (CH‐4‐furyl‐*B*); 113.2 (CH‐3‐furyl‐*B*); 127.9 (CH‐6); 144.5 (CH‐5‐furyl‐*A*); 146.2 (CH‐5‐furyl‐*B*); 146.4 and 150.0 (C‐2,4); 151.2 (C‐7a); 152.5 (C‐2‐furyl‐*B*); 152.8 (C‐2‐furyl‐*A*). HR‐ESI‐MS: *found*: 294.1238 ([M + H]^+^, calcd for C_17_H_16_O_2_N_3_^+^: 294.1237); HR‐ESI‐MS: *found*: 316.1058 ([M + Na]^+^, calcd for C_17_H_15_O_2_N_3_Na^+^: 316.1057).

### 4.12 2‐(2,4‐Dichloro‐7*H*‐pyrrolo[2,3‐*d*]pyrimidin‐7‐yl)ethan‐1‐ol (9)


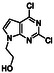


2,4‐Dichloro‐7*H*‐pyrrolo[2,3‐*d*]pyrimidine (**1**) (1 g, 5.32 mmol) was dissolved in DMF (8 mL), and then Cs_2_CO_3_ (2.08 mg, 6.38 mmol) was added. The mixture was stirred for 30 min, and then 2‐chloroethanol (0.40 mL, 5.85 mmol) was slowly added dropwise. The mixture was stirred at rt for 16 h. Volatiles were evaporated. HPFC (SiO_2_, DCM/MeOH 1:0 → 3:7) and lyophilization from H_2_O/*t*BuOH gave **9** (0.28 g, 22%) as a white powder. ^1^H NMR (500 MHz, DMSO‐d_6_): 3.75 (t, 2H, *J*_*CH2*,*CH2*_ = 5.5 Hz, NCH_2_C**H**_**2**_OH); 4.28 (t, 2H, *J*_*CH2*,*CH2*_ = 5.5 Hz, NC**H**_**2**_CH_2_OH); 4.94 (vbs, 1H, NCH_2_CH_2_O**H**); 6.70 (d, 1H, *J*_5,6_ = 3.6 Hz, H‐5); 7.78 (d, 1H, *J*_6,5_ = 3.6 Hz, H‐6); ^13^C NMR (125.7 MHz, DMSO‐d_6_): 47.4 (N**C**H_2_CH_2_OH); 59.5 (NCH_2_ **C**H_2_OH); 98.7 (CH‐5); 116.1 (C‐4a); 133.1 (CH‐6); 150.0 and 150.9 (C‐2,4); 151.8 (C‐7a). HR‐ESI‐MS: *found*: 232.0040 ([M + H]^+^, calcd for C_8_H_8_ON_3_Cl_2_^+^: 232.0039); HR‐ESI‐MS: *found*: 253.9860 ([M + Na]^+^, calcd for C_8_H_7_ON_3_Cl_2_Na^+^: 253.9858).

### 4.13 2‐(2‐Chloro‐4‐(furan‐2‐yl)‐7*H*‐pyrrolo[2,3‐*d*]pyrimidin‐7‐yl)ethan‐1‐ol (10a)


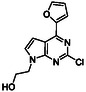


GP A starting from compound **9** (107.2 mg, 0.46 mmol) and 2‐furylboronic acid (56.9 mg, 0.51 mmol), reaction performed in a microwave reactor for 1 h at 100 °C. HPFC (SiO_2_, cHex/EtOAc 8:2 → 5:5) gave **10a** (54.2 mg, 44%) as a white solid. ^1^H NMR (500 MHz, DMSO‐d_6_): 3.76 (q, 2H, *J*_*CH2*,*CH2*_ = *J*_*CH2*, *OH*_ = 5.5 Hz, NCH_2_C**H**_**2**_OH); 4.27 (t, 2H, *J*_*CH2*,*CH2*_ = 5.6 Hz, NC**H**_**2**_CH_2_OH); 4.97 (t, 1H, *J*_*OH*,*CH2*_ = 5.4 Hz, NCH_2_CH_2_O**H**); 6.81 (dd, 1H, *J*_4,3_ = 3.6 Hz, *J*_4,5_ = 1.7 Hz, H‐4‐furyl); 7.00 (d, 1H, *J*_5,6_ = 3.6 Hz, H‐5); 7.51 (dd, 1H, *J*_3,4_ = 3.6 Hz, *J*_3,5_ = 0.7 Hz, H‐3‐furyl); 7.72 (d, 1H, *J*_6,5_ = 3.6 Hz, H‐6); 8.09 (dd, 1H, *J*_5,4_ = 1.8 Hz, *J*_5,3_ = 0.7 Hz, H‐5‐furyl); ^13^C NMR (125.7 MHz, DMSO‐d_6_): 46.8 (N**C**H_2_CH_2_OH); 59.6 (NCH_2_ **C**H_2_OH); 100.1 (CH‐5); 111.2 (C‐4a); 112.9 (CH‐4‐furyl); 114.6 (CH‐3‐furyl); 132.3 (CH‐6); 147.0 (CH‐5‐furyl); 147.6 (C‐4); 151.3 (C‐2‐furyl); 152.0 (C‐2); 152.9 (C‐7a). HR‐ESI‐MS: *found*: 264.0535 ([M + H]^+^, calcd for C_12_H_11_O_2_N_3_Cl^+^: 264.0534); HR‐ESI‐MS: *found*: 286.0355 ([M + Na]^+^, calcd for C_12_H_10_O_2_N_3_ClNa^+^: 286.0354).

### 4.14 2‐(2,4‐Di(furan‐2‐yl)‐7*H*‐pyrrolo[2,3‐*d*]pyrimidin‐7‐yl)ethan‐1‐ol (11aa)


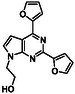


GP B starting from compound **10a** (68.8 mg, 0.30 mmol) and 2‐furylboronic acid (132.7 mg, 1.19 mmol). HPFC (SiO_2_, cHex/EtOAc 8:2 → 5:5) and lyophilization from H_2_O/*t*BuOH gave **11aa** (39.8 mg, 45%) as a pale‐yellow powder. ^1^H NMR (500 MHz, DMSO‐d_6_): 3.81 (t, 2H, *J*_*CH2*,*CH2*_ = 5.7 Hz, NCH_2_C**H**_**2**_OH); 4.35 (t, 2H, *J*_*CH2*,*CH2*_ = 5.7 Hz, NC**H**_**2**_CH_2_OH); 5.00 (bs, 1H, NCH_2_CH_2_O**H**); 6.69 (dd, 1H, *J*_4,3_ = 3.3 Hz, *J*_4,5_ = 1.8 Hz, H‐4‐furyl‐*A*); 6.80 (dd, 1H, *J*_4,3_ = 3.5 Hz, *J*_4,5_ = 1.8 Hz, H‐4‐furyl‐*B*); 6.96 (d, 1H, *J*_5,6_ = 3.5 Hz, H‐5); 7.30 (dd, 1H, *J*_3,4_ = 3.3 Hz, *J*_3,5_ = 0.9 Hz, H‐3‐furyl‐*A*); 7.52 (dd, 1H, *J*_3,4_ = 3.5 Hz, *J*_3,5_ = 0.8 Hz, H‐3‐furyl‐*B*); 7.69 (d, 1H, *J*_6,5_ = 3.5 Hz, H‐6); 7.88 (dd, 1H, *J*_5,4_ = 1.8 Hz, *J*_5,3_ = 0.9 Hz, H‐5‐furyl‐*A*); 8.07 (dd, 1H, *J*_5,4_ = 1.8 Hz, *J*_5,3_ = 0.8 Hz, H‐5‐furyl‐*B*); ^13^C NMR (125.7 MHz, DMSO‐d_6_): 46.5 (N**C**H_2_CH_2_OH); 59.8 (NCH_2_ **C**H_2_OH); 99.9 (CH‐5); 110.7 (C‐4a); 111.7 (CH‐3‐furyl‐*A*); 112.2 (CH‐4‐furyl‐*A*); 112.6 (CH‐4‐furyl‐*B*); 113.2 (CH‐3‐furyl‐*B*); 131.7 (CH‐6); 144.5 (CH‐5‐furyl‐*A*); 146.2 (CH‐5‐furyl‐*B*); 146.3 (C‐4); 150.1 (C‐2); 151.9 (C‐7a); 152.5 (C‐2‐furyl‐*B*); 152.8 (C‐2‐furyl‐*A*). HR‐ESI‐MS: *found*: 296.1032 ([M + H]^+^, calcd for C_16_H_14_O_3_N_3_^+^: 296.1030); HR‐ESI‐MS: *found*: 318.0851 ([M + Na]^+^, calcd for C_16_H_13_O_3_N_3_Na^+^: 318.0849).

### 4.15 Cell Lines

For the cytotoxicity assay, all cell lines were purchased from the American Type Culture Collection (ATCC), except for HCT116p53‐/‐ cells, which were obtained from Horizon Discovery. The cells were maintained in TPP 75 cm^2^ plastic tissue culture flasks and subcultured in cell culture medium according to ATCC or Horizon recommendations (DMEM/McCoy's 5A modified medium, IMDM/EMEM/RPMI 1640, supplemented with 10% fetal bovine serum (FBS) and antibiotics, including 100 U/mL Penicillin‐Streptomycin). Cells were incubated at 37 °C in a humidified incubator with a 5% CO_2_ atmosphere. The aequorin reporter cell lines ES‐010‐A (human A_1_), ES‐011‐A (human A_2A_), ES‐012‐A (human A_3_), and ES‐013‐A (human A_2B_), which express the human AR of interest, photoprotein aequorin, and the promiscuous Gα16 protein subunit, were purchased from Revvity. ES‐010‐A and ES‐012‐A were cultivated in Ham's F‐12 medium with 10% FBS, 0.4 mg/mL geneticin, and 0.25 mg/mL zeocin. ES‐011‐A and ES‐013‐A were cultured in EMEM medium supplemented with 10% FBS, 100 μg/mL geneticin, and 10 μg/mL zeocin.

### 4.16 Cytotoxic MTS Assay

The in vitro cytotoxicity of compounds was assessed using the standard 3‐(4,5 4,5‐dimethyl‐thiazol‐2yl)‐5‐(3‐carboxymethoxyphenyl)‐2‐(sulfophenyl)‐2H‐tetrazolium (MTS). Cell suspensions were prepared and diluted according to the specific cell type and the expected target cell density (25,000–35,000 cells/mL, based on cell growth characteristics). An automatic pipettor (30 µL) was used to add cells into 384‐well plates. All tested compounds were dissolved in DMSO, and fourfold dilutions of the intended test concentrations were added to the microtiter plate wells at time zero using the Echo acoustic noncontact liquid handler Echo550 (Labcyte, San Jose, CA, USA); the corresponding DMSO concentration was used in the controls. The assays were accomplished in technical duplicates and at least three biological replicates, carried out using cells from independent cultures at different passage numbers. The cells were incubated with the tested compound for 72 h at 37 °C in a 5% CO_2_ atmosphere with 100% humidity. At the end of the incubation period, cells were assayed using the MTS test. Aliquots (5 µL) of the MTS stock solution were pipetted into each well and incubated for 1–4 h. After this incubation period, the absorbance was measured at 490 nm using an Envision reader (PerkinElmer, Waltham, MA, USA). The IC_50_ value, representing the concentration of the compound lethal to 50% of the tumor cells, was calculated from the corresponding dose–response curves using Dotmatics software, using nonlinear regression with a standard four‐parameter logistic model.

### 4.17 FLIPR Analysis

Cell lines for analysis of ARs were maintained in the presence of the selection antibiotics to ensure high expression of both aequorin protein and the AR of interest. 24 h prior to the harvest, the medium was changed to one without selection antibiotics. On the following day, cells were harvested using PBS containing 0.5 mM EDTA and then incubated in DMEM/F12 medium supplemented with 0.1% BSA and 5 μM coelenterazine for 4 h at RT in the dark.

Coelenterazine‐loaded cells (5 × 10^3^ cells per well) were injected using the reader's automatic injection system, and relative light emission was immediately recorded (FLIPR Penta multimodal reader, Molecular Devices). During this first read (compound addition), the baseline signaling or any potential agonistic response of the test compounds was measured. Following the initial measurement, the cells were incubated within the instrument for an additional 15 min at RT.

To evaluate the antagonistic response (second read), a reference agonist was dispensed at its EC_80_ concentration. An inactive compound yields a full luminescent response equivalent to the EC_80_ agonist control alone. Conversely, an active antagonist blocks the receptor, resulting in a concentration‐dependent reduction of the luminescent signal, potentially reducing it back to the baseline level.

After the initial measurement, the cells were incubated within the instrument for an additional 15 min at RT. The antagonistic response, indicated by a reduction in luminescence, was measured immediately after adding a reference agonist at 80% of its maximal effective concentration (EC80).

Raw data AUC were normalized as a percentage of the response to the reference agonist (NECA) or antagonists (DPCPX for A_1_, ZM 241 385 for A_2A_, MRS1706 for A_2B_), MRS1220 for A_3_. The normalized responses were plotted against the concentrations of the test compounds, and EC_50_ values (for agonists) and IC_50_ (for antagonists) were determined. The presented data represent the mean values obtained from two independent biological replicates (performed using different cell passages), with each experiment performed in technical triplicate and analyzed using Dotmatics software.

### 4.18 Annexin V Binding Assay

We employed an apoptosis detection assay based on Annexin V‐FITC and propidium iodide staining, utilizing a commercial kit from Exbio. The procedure followed the manufacturer's protocol with minor modifications. Briefly, cells were adjusted to the appropriate concentration, washed with Annexin V binding buffer, and subsequently stained with Annexin V‐FITC and propidium iodide. To reduce cytotoxic effects observed in CCRF‐CEM cells, we used only half of the recommended volume of propidium iodide, as the standard concentration caused acute toxicity. Stained cells were incubated for 15 min at RT in the dark, centrifuged, and resuspended in 100 μL of Annexin V binding buffer. Samples were immediately subjected to flow cytometric analysis using a FACSAria II cytometer (Becton Dickinson), with a minimum acquisition of 10,000 cells per sample.

## Funding

This work was supported by the Ministerstvo Školství, Mládeže a Tělovýchovy (LX22NPO5102, LM2023052, and LM2023053).

## Conflicts of Interest

The authors declare no conflicts of interest.

## Supporting information

The authors have cited additional references within the Supporting Information [37, 38]. The data (raw NMR and UPLC data) that support the findings of this study are openly available in Data Catch‐all Repository at https://doi.org/10.48700/datst.nqzqa‐4nz46.

## Data Availability

The data that support the findings of this study are openly available in Data Catch‐all Repository at https://doi.org/10.48700/datst.nqzqa‐4nz46, reference number 10.48700/220x4‐5wa12.
