# Supplementary material for: Synthesis and Biological Activity of 2,6‐Disubstituted and 2,6,9‐Trisubstituted 7‐Deazapurine Nucleobases
Source: ChemMedChem. 2026 Aug 13;21(16):e70419. doi: 10.1002/cmdc.70419 (PMC13473637; doi:10.1002/cmdc.70419)
Supplement: Supplementary file 1 [file CMDC-21-e70419-s001.pdf]

Synthesis and Biological Activity of 2,6-Disubstituted and 2,6,9-Trisubstituted 7-deazapurine Nucleobases

Ugnė Šinkevičiūtė,#[a,b] Kateřina Burgetová Ječmeňová,#[c] Soňa Gurská,[c] Lenka Poštová Slavětínská,[a] Vincent Baran, [a] Pavel Polishchuk,[c] Petr Džubák,[c] Michal Hocek [a,b], Marián Hajdúch, [c]* Michal Tichý[a]*

[a] *Dr. U. Šinkevičiūtė, Dr. L. Poštová Slavětínská, Dr. V. Baran, Prof. M. Hocek, Dr. M. Tichý*

Bioorganic and Medicinal Chemistry of Nucleic Acids

Institute of Organic Chemistry and Biochemistry, Czech Academy of Sciences, Flemingovo nam. 2, CZ-16610 Prague 6, Czech Republic.

E-mail: michal.tichy@uochb.cas.cz

[b] *Prof. M. Hocek*

Department of Organic Chemistry, Faculty of Science, Charles University in Prague, Hlavova 8, CZ-12843 Prague 2, Czech Republic.

[c] *K. Burgetová Ječmeňová, Dr. S. Gurská, Dr. P. Polishchuk, Dr. P. Džubák, Prof. M. Hajdúch*

Institute of Molecular and Translational Medicine, Faculty of Medicine and Dentistry, Palacký University and University Hospital Olomouc, Hněvotínská 5, CZ-77515 Olomouc, Czech Republic.

E-mail: marian.hajduch@upol.cz

# these authors contributed equally

* authors for correspondence: e-mail: michal.tichy@uochb.cas.cz; marian.hajduch@upol.cz

Table of Contents

S1. Experimental part 3

S2. Adenosine Receptor Activity Screening 21

S3. Annexin V binding assay 22

S4. Cytotoxicity Data – IC_{50} Curves 25

S5. Copies of NMR spectra 68

S4. HPLC Purity of Final Compounds 95

S5. References 95

S1. Experimental part

2,4-Dichloro-7-{[2-(trimethylsilyl)ethoxy]methyl}-7*H*-pyrrolo[2,3-*d*]pyrimidine (2)

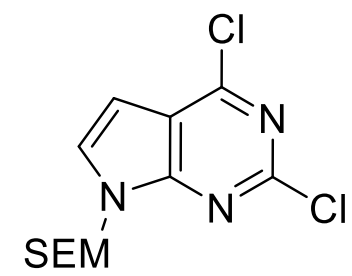

An ice-cold solution of 2,4-dichloro-7*H*-pyrrolo[2,3-*d*]pyrimidine (**1**) (1.0 g, 5.32 mmol) in DMF (8 mL) was portionwise treated by NaH (60 % in mineral oil, 276.6 mg, 6.92 mmol). The mixture was stirred at 0 °C for 30 min and then dropwise treated with SEM-Cl (1.15 g, 6.92 mmol). The mixture was stirred at rt for 2 h, treated with water and extracted with EtOAc. Combined organic layers were dried over anhydrous Na_2SO_4. HPFC (SiO_2, cHex/EtOAc 1:0 → 9:1) gave **2** (1.52 g, 90 %) as a yellow oil. ^{1}H NMR spectrum is in agreement with the literature.[1]

4-(Benzofuran-2-yl)-2-chloro-7-{[2-(trimethylsilyl)ethoxy]methyl}-7*H*-pyrrolo[2,3-*d*]pyrimidine (3b)

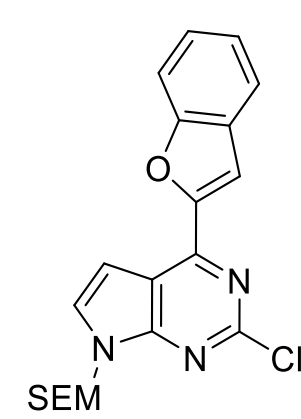

GP A starting from compound **2** (1.18 g, 3.70 mmol) and 2-benzofurylboronic acid (658.4 mg, 4.07 mmol. HPFC (SiO_2, cHex/EtOAc 1:0 → 9:1) gave **3b** (1.35 g, 92 %) as a yellow solid. ^{1}H NMR (500 MHz, DMSO-d_6): –0.09 (s, 9H, CH_3Si); 0.86 (m, 2H, $OCH_2C\mathbf{H_2}Si$); 3.56 (m, 2H, $OC\mathbf{H_2}CH_2Si$); 5.62 (NCH_2O); 7.29 (d, 1H, $J_{5,6}$ = 3.7 Hz, H-5); 7.38 (m, 1H, H-5-benzofuryl); 7.51 (ddd, 1H, $J_{6,7}$ = 8.2 Hz, $J_{6,5}$ = 7.2 Hz, $J_{6,4}$ = 1.4 Hz, H-6-benzofuryl); 7.81–7.84 (m, 2H, H-4,7-benzofuryl); 7.94 (d, 1H, $J_{6,5}$ = 3.7 Hz, H-6); 7.99 (d, 1H, $J_{3,7}$ = 0.9 Hz, H-3-benzofuryl); ^{13}C NMR (125.7 MHz, DMSO-d_6): –1.4 (CH_3Si); 17.1 ($OCH_2\mathbf{C}H_2Si$); 65.9 ($O\mathbf{C}H_2CH_2Si$); 72.7 (OCH_2N); 101.6 (C-5); 110.6 (CH-3-benzofuryl); 112.0 (CH-7-benzofuryl); 112.7 (C-4a); 122.7 (CH-4-benzofuryl); 124.0 (CH-5-benzofuryl); 127.1 (CH-6-benzofuryl); 127.6 (C-3a-benzofuryl); 132.5 (CH-6); 148.0 (C-4); 152.51 and 152.5 (C-2, C-2-benzofuryl); 153.78 (C-7a); 155.4 (C-7a-benzofuryl). HR-ESI-MS: *found*: 400.1247 ($[M + H]^+$, calcd for $C_{20}H_{23}ClO_2N_3Si^+$: 400.1243).

2-Chloro-4-(naphth-1-yl)-7-{[2-(trimethylsilyl)ethoxy]methyl}-7*H*-pyrrolo[2,3-*d*]pyrimidine (3c)

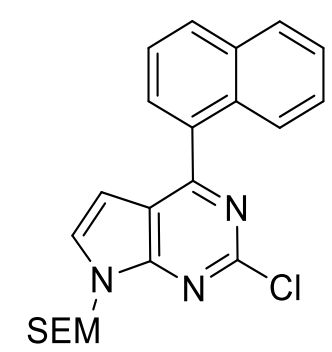

GP A starting from compound **2** (593.1 mg, 1.86 mmol) and naphthalene-1-boronic acid (352.6 mg, 2.05 mmol). HPFC (SiO_2, cHex/EtOAc 1:0 → 9:1) gave **3c** (470.4 g, 62 %) as a yellow amorphous solid. ^{1}H NMR (500 MHz, DMSO-d$_6$): –0.07 (s, 9H, CH_3Si); 0.87 (m, 2H, $OCH_2C\mathbf{H_2}Si$); 3.60 (m, 2H, $OC\mathbf{H_2}CH_2Si$); 5.66 (s, 2H, NCH_2O); 6.45 (d, 1H, $J_{5,6}$ = 3.6 Hz, H-5); 7.55 (ddd, 1H, $J_{7,8}$ = 8.5 Hz, $J_{7,6}$ = 6.8 Hz, $J_{7,5}$ = 1.4 Hz, H-7-naphthyl); 7.61 (ddd, 1H, $J_{6,5}$ = 8.2 Hz, $J_{6,7}$ = 6.8 Hz, $J_{6,8}$ = 1.3 Hz, H-6-naphthyl); 7.70 (dd, 1H, $J_{3,4}$ = 8.2 Hz, $J_{3,2}$ = 7.1 Hz, H-3-naphthyl); 7.79 (dd, 1H, $J_{2,3}$ = 7.1 Hz, $J_{2,4}$ = 1.3 Hz, H-2-naphthyl); 7.82 (d, 1H, $J_{6,5}$ = 3.6 Hz, H-6); 8.00 (bdq, 1H, $J_{8,7}$ = 8.5 Hz, $J_{8,6}$ = $J_{8,5}$ = $J_{8,4}$ = 1.0 Hz, H-8-naphthyl); 8.08 (bdm, 1H, $J_{5,6}$ = 8.2 Hz, H-5-naphthyl); 8.15 (dt, 1H, $J_{4,3}$ = 8.2 Hz, $J_{4,2}$ = $J_{4,8}$ = 1.1 Hz, H-4-naphthyl); ^{13}C NMR (125.7 MHz, DMSO-d$_6$): –1.4 (CH_3Si); 17.1 ($OCH_2\mathbf{C}H_2Si$); 66.0 ($O\mathbf{C}H_2CH_2Si$); 72.8 (NCH_2O); 101.0 (CH-5); 117.1 (C-4a); 125.1 (CH-8-naphthyl); 125.4 (CH-3-naphthyl); 126.5 (CH-6-naphthyl); 127.0 (CH-7-naphthyl); 128.47 (CH-2-naphthyl); 128.52 (CH-5-naphthyl); 130.1 (C-8a-naphthyl); 130.4 (CH-4-naphthyl); 131.9 (CH-6); 133.3 and 133.5 (C-1,4a-naphthyl); 152.4 (C-2); 152.9 (C-7a); 160.1 (C-4). HR-ESI-MS: *found*: 410.1451 ([M + H]$^+$, calcd for $C_{22}H_{25}ON_3ClSi^+$: 410.1450); HR-ESI-MS: *found*: 432.1271 ([M + Na]$^+$, calcd for $C_{22}H_{24}ON_3ClNaSi^+$: 432.1269).

2-Chloro-4-(naphthalen-2-yl)-7-{[2-(trimethylsilyl)ethoxy]methyl}-7*H*-pyrrolo[2,3-*d*]pyrimidine (3d) and **2,4-bis(naphthalen-2-yl)-7-{[2-(trimethylsilyl)ethoxy]methyl}-7*H*-pyrrolo[2,3-*d*]pyrimidine (4dd)**

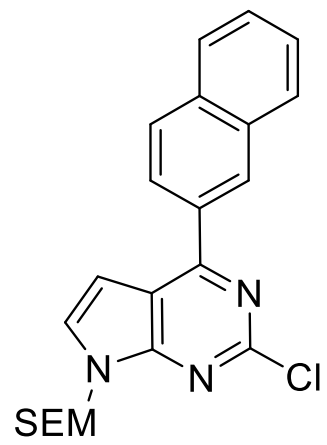

3d

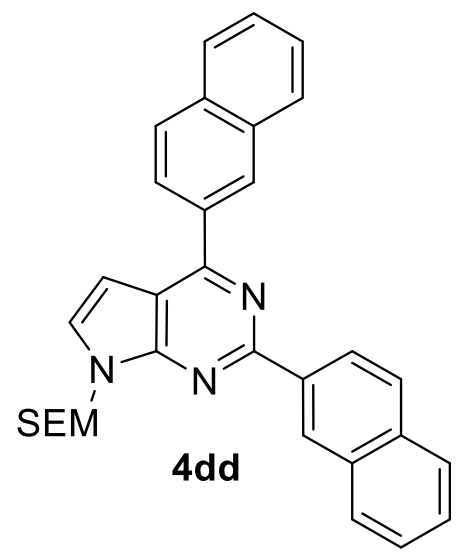

4dd

GP A starting from compound **2** (536.7 mg, 1.69 mmol) and naphthalene-2-boronic acid (319.0 mg, 1.86 mmol), reaction temperature 100 °C. HPFC (SiO_2, cHex/EtOAc 1:0 → 9:1) gave **3d** (491.7 mg, 71 %) as a pale-yellow solid and 52.9 mg of disubstituted compound **4dd** (53 mg, 6 %) as a yellow oil, which was used directly for the next step.

Characterization of compound **3d**: ^{1}H NMR (500 MHz, DMSO-d$_6$): –0.07 (s, 9H, CH_3Si); 0.87 (m, 2H, $OCH_2C\mathbf{H_2}Si$); 3.58 (m, 2H, $OC\mathbf{H_2}CH_2Si$); 5.65 (s, 2H, NCH_2O); 7.24 (d, 1H, $J_{5,6}$ = 3.7 Hz, H-5); 7.60–7.68 (m, 2H, H-6,7-naphthyl); 7.93 (d, 1H, $J_{6,5}$ = 3.7 Hz, H-6); 8.03 (m, 1H, H-5-naphthyl); 8.13 (d, 1H, $J_{4,3}$ = 8.7 Hz, H-4-naphthyl); 8.22 (m, 1H, H-8-naphthyl); 8.28 (dd, 1H, $J_{3,4}$ = 8.6 Hz, $J_{3,1}$ = 1.8 Hz, H-3-naphthyl); 8.78 (bd, 1H, $J_{1,3}$ = 1.8 Hz, H-1-naphthyl); ^{13}C NMR (125.7 MHz, DMSO-d$_6$): –1.4 (CH_3Si); 17.1 ($OCH_2\mathbf{C}H_2Si$); 65.9 ($O\mathbf{C}H_2CH_2Si$); 72.74(NCH_2O); 101.5 (CH-5); 114.4 (C-4a); 125.4 (CH-3-naphthyl); 126.8 (CH-6/7-naphthyl); 127.7 (CH-5-naphthyl); 127.9 (CH-6/7-naphthyl); 128.7 (CH-4-naphthyl); 129.26 and 129.27 (CH-1,8-naphthyl); 132.1 (CH-6); 132.8 (C-8a-naphthyl); 133.6 and 134.0 (C-2,4a-naphthyl); 152.67 (C-2); 153.5 (C-7a); 158.1 (C-4); HR-ESI-MS: *found*: 410.1452 ([M + H]$^+$, calcd for $C_{22}H_{25}ON_3ClSi^+$: 410.1450); HR-ESI-MS: *found*: 432.1271 ([M + Na]$^+$, calcd for $C_{22}H_{24}ON_3ClNaSi^+$: 432.1269).

2-(Benzofuran-2-yl)-4-(furan-2-yl)-7-{[2-(trimethylsilyl)ethoxy]methyl}-7*H*-pyrrolo[2,3-*d*]pyrimidine (4ab)

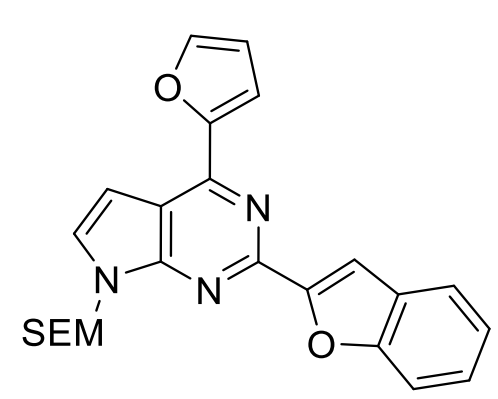

GP B starting from compound **3a** (151.9 mg, 0.44 mmol) and 2-benzofurylboronic acid (126.8 mg, 0.78 mmol). HPFC (SiO_2, cHex/EtOAc 1:0 → 9:1) gave crude product **4ab** as a yellow solid (161.7 mg), which was used directly for the next step.

4-(Furan-2-yl)-2-(naphth-1-yl)-7-{[2-(trimethylsilyl)ethoxy]methyl}-7*H*-pyrrolo[2,3-*d*]pyrimidine (4ac)

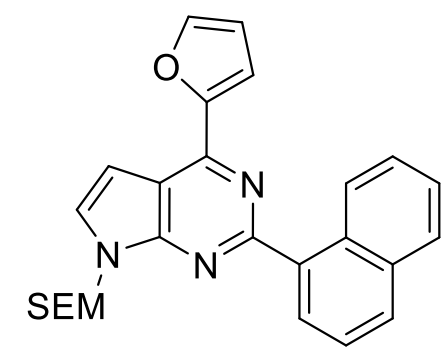

GP B starting from compound **3a** (105.1 mg, 0.30 mmol) and naphthalene-1-boronic acid (103.3 mg, 0.60 mmol). HPFC (SiO_2, cHex/EtOAc 1:0 → 9:1) gave crude product **4ac** as a yellow solid (131.8 mg), which was used directly for the next step.

4-(Furan-2-yl)-2-(naphthalen-2-yl)-7-{[2-(trimethylsilyl)ethoxy]methyl}-7*H*-pyrrolo[2,3-*d*]pyrimidine (4ad)

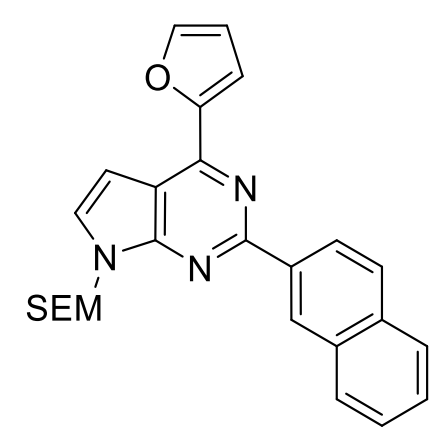

GP B starting from compound **3a** (139.3 mg, 0.40 mmol) and naphthalene-2-boronic acid (171.2 mg, 1.00 mmol). HPFC (SiO_2, cHex/EtOAc 1:0 → 9:1) gave crude product **4ad** as a yellow oil (82.1 mg), which was used directly for the next step.

4-(Benzofuran-2-yl)-2-(furan-2-yl)-7-{[2-(trimethylsilyl)ethoxy]methyl}-7*H*-pyrrolo[2,3-*d*]pyrimidine (4be)

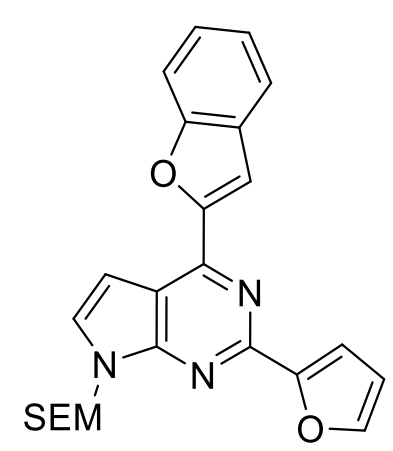

GP A starting from compound **3b** (153.0 mg, 0.38 mmol) was reacted with 2-furylboronic acid (107.0 mg, 0.96 mmol). HPFC (SiO_2, cHex/EtOAc 1:0 → 9:1) gave crude product **4be** as a yellow solid (106.4 mg), which was used directly for the next step.

2,4-Bis(benzofuran-2-yl)-7-{[2-(trimethylsilyl)ethoxy]methyl}-7*H*-pyrrolo[2,3-*d*]pyrimidine (4bb)

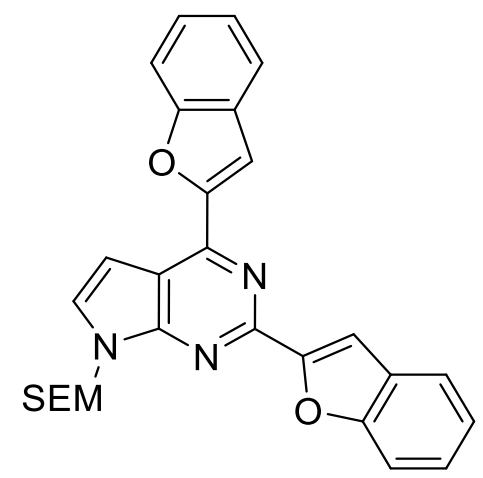

GP C starting from compound **3b** (104.7 mg, 0.26 mmol) and 2-benzofurylboronic acid (127.2 mg, 0.79 mmol). HPFC (SiO_2, cHex/EtOAc 1:0 → 9:1) gave crude product **4bb** (124.4 mg) as a colorless oil, which was used directly for the next step.

4-(Benzofuran-2-yl)-2-(naphth-1-yl)-7-{[2-(trimethylsilyl)ethoxy]methyl}-7*H*-pyrrolo[2,3-*d*]pyrimidine (4bc)

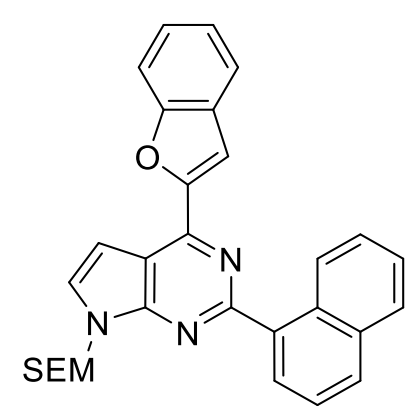

GP B starting from compound **3b** (112.5 mg, 0.28 mmol) and naphthalene-1-boronic acid (120.9 mg, 0.70 mmol). HPFC (SiO_2, cHex/EtOAc 1:0 → 9:1) gave crude product **4bc** as a yellow oil (82.1 mg), which was used directly for the next step.

4-(Benzofuran-2-yl)-2-(naphthalen-2-yl)-7-{[2-(trimethylsilyl)ethoxy]methyl}-7*H*-pyrrolo[2,3-*d*]pyrimidine (4bd)

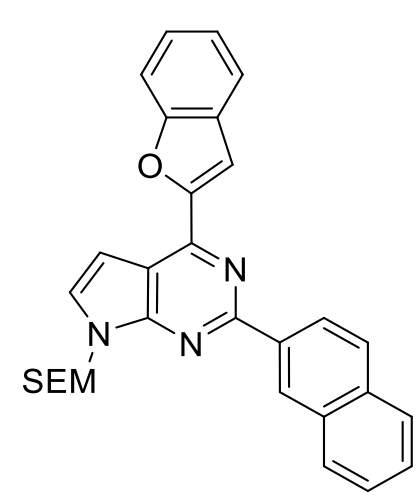

GP A starting from compound **3b** (134.3 mg, 0.34 mmol) and naphthalene-2-boronic acid (173.3 mg, 1.01 mmol). HPFC (SiO_2, cHex/EtOAc 1:0 → 9:1) gave crude product **4bd** as a yellow oil (19.4 mg), which was used directly for the next step.

2-(Furan-2-yl)-4-(naphth-1-yl)-7-{[2-(trimethylsilyl)ethoxy]methyl}-7*H*-pyrrolo[2,3-*d*]pyrimidine (4ca)

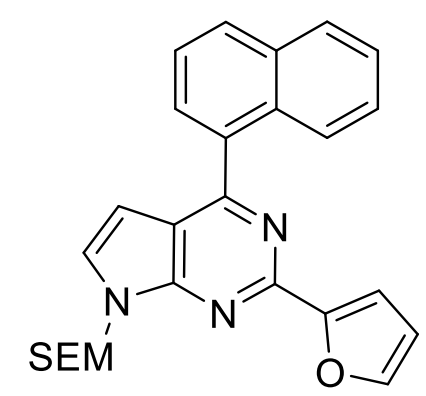

GP B starting from compound **3c** (120.5 mg, 0.29 mmol) and 2-furanylboronic acid (98.7 mg, 0.88 mmol). HPFC (SiO_2, cHex/EtOAc 1:0 → 9:1) gave crude product **4ca** as a yellow oil (51.3 mg), which was used directly for the next step.

2-(Benzofuran-2-yl)-4-(naphth-1-yl)-7-{[2-(trimethylsilyl)ethoxy]methyl}-7*H*-pyrrolo[2,3-*d*]pyrimidine (4cb)

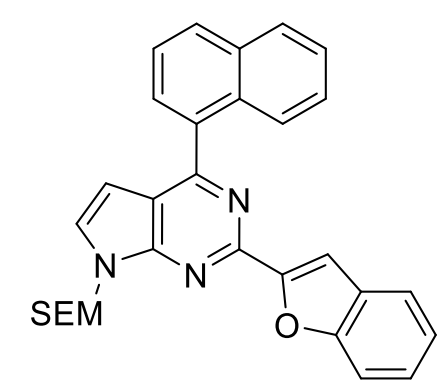

GP C starting from compound **3c** (103.3 mg, 0.25 mmol) and 2-benzofurylboronic acid (122.4 mg, 0.76 mmol). HPFC (SiO_2, cHex/EtOAc 1:0 → 9:1) gave crude product **4cb** as a colorless oil (121.6 mg), which was used directly for the next step.

2,4-Di(naphth-1-yl)-7-{[2-(trimethylsilyl)ethoxy]methyl}-7*H*-pyrrolo[2,3-*d*]pyrimidine (4cc)

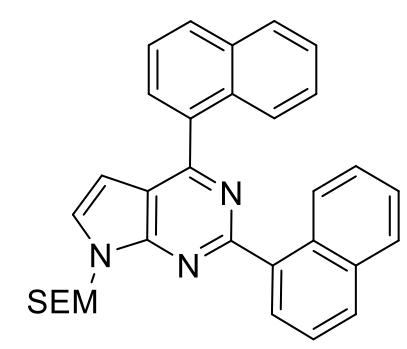

GP B starting from compound **2** (162.2 mg, 0.51 mmol) and naphthalene-1-boronic acid (438.3 mg, 2.55 mmol). The reaction was performed in microwave reactor at 100 °C for 2 h. HPFC (SiO_2, cHex/EtOAc 1:0 → 9:1) gave crude product **4cc** (54.3 mg) as a yellow oil, which was used directly for the next step.

4-(Naphth-1-yl)-2-(naphthalen-2-yl)-7-{[2-(trimethylsilyl)ethoxy]methyl}-7*F*-pyrrolo[2,3-*d*]pyrimidine (4cd)

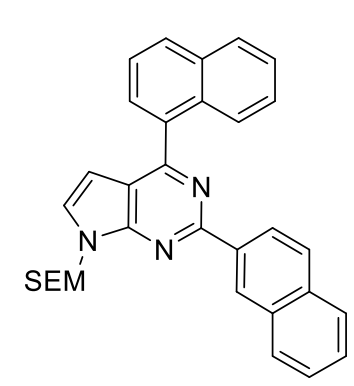

GP B starting from compound **3c** (109.4 mg, 0.27 mmol) and naphthalene-2-boronic acid (137.7 mg, 0.80 mmol). The reaction was performed in microwave reactor at 140 °C for 1 h. HPFC

(SiO_2, cHex/EtOAc 1:0 → 9:1) gave crude product **4cd** (46.5 mg) as a yellow oil, which was used directly for the next step.

2-(Furan-2-yl)-4-(naphthalen-2-yl)-7-{[2-(trimethylsilyl)ethoxy]methyl}-7*H*-pyrrolo[2,3-*d*]pyrimidine (4da)

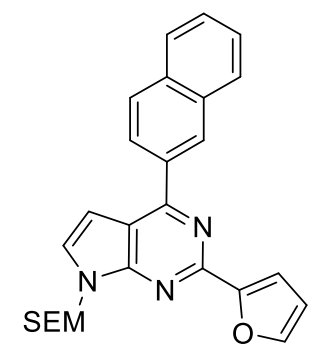

GP B starting from compound **3d** (115.9 mg, 0.28 mmol) and 2-furanylboronic acid (101.9 mg, 0.91 mmol). The reaction was performed in microwave reactor at 100 °C for 2 h. HPFC (SiO_2, cHex/EtOAc 1:0 → 9:1) gave crude product **4da** (29.7 mg) as a yellow oil, which was used directly for the next step.

2-(Benzofuran-2-yl)-4-(naphthalen-2-yl)-7-{[2-(trimethylsilyl)ethoxy]methyl}-7*H*-pyrrolo[2,3-*d*]pyrimidine (4db)

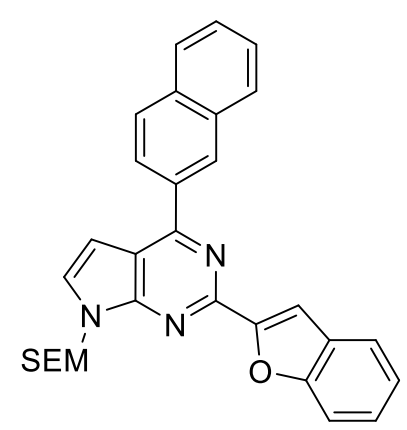

GP B starting from compound **3d** (105.5 mg, 0.26 mmol) and 2-benzofurylboronic acid (125.0 mg, 0.77 mmol). HPFC (SiO_2, cHex/EtOAc 1:0 → 9:1) gave crude product **4db** (124.2 mg) as a colorless oil, which was used directly for the next step.

2-(Naphth-1-yl)-4-(naphthalen-2-yl)-7-{[2-(trimethylsilyl)ethoxy]methyl}-7*H*-pyrrolo[2,3-*d*]pyrimidine (4dc)

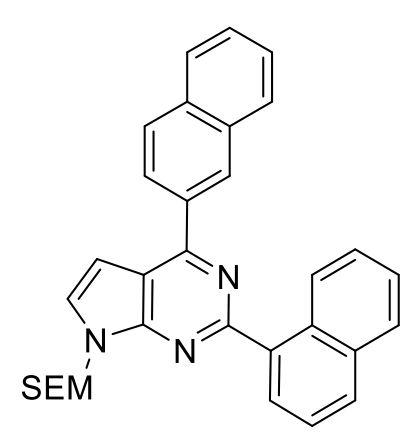

Gp A starting from compound **3d** (101.4 mg, 0.25 mmol) and naphthalene-1-boronic acid (127.6 mg, 0.74 mmol). HPFC (SiO_2, cHex/EtOAc 1:0 → 9:1) gave crude product **4dc** (120.3 mg) as a yellow oil, which was used directly for the next step.

2-(Benzofuran-2-yl)-4-(furan-2-yl)-7*H*-pyrrolo[2,3-*d*]pyrimidine (5ab)

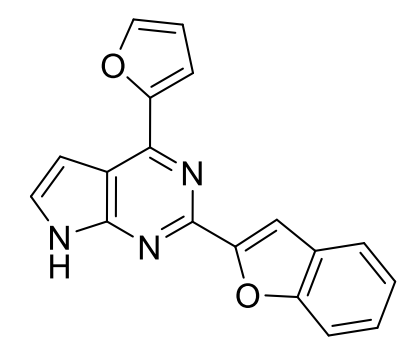

GP D using crude compound **4ab** (156.7 mg, 0.36 mmol). RP-HPFC (C-18, H_2O/MeOH 0 → 100 %) gave **5ab** (39.9 mg, 31 %, over two steps) as a yellow solid. ^{1}H NMR (500 MHz, DMSO-d$_6$): 6.83 (dd, 1H, $J_{4,3}$ = 3.5 Hz, $J_{4,5}$ = 1.8 Hz, H-4-furyl); 7.01 (d, 1H, $J_{5,6}$ = 3.5 Hz, H-5); 7.32 (bt, 1H, $J_{5,6}$ = $J_{5,4}$ = 7.5 Hz, H-5-benzofuryl); 7.41 (ddd, 1H, $J_{6,7}$ = 8.0 Hz, $J_{6,5}$ = 7.4 Hz, $J_{6,4}$ = 1.3 Hz, H-6-benzofuryl); 7.60 (bd, 1H, $J_{3,4}$ = 3.4 Hz, H-3-furyl); 7.10–7.50 (m, 3H, H-3,7-benzofuryl, H-6); 7.77 (bd, 1H, $J_{4,5}$ = 7.7 Hz, H-4-benzofuryl); 8.10 (bd, 1H, $J_{5,4}$ = 1.8 Hz, H-5-furyl); 12.39 (bs, 1H, NH); ^{13}C NMR (125.7 MHz, DMSO-d$_6$): 100.9 (C-5); 107.2 (CH-3-benzofuryl); 110.9 (C-4a); 111.6 (CH-7-benzofuryl); 112.7 (CH-4-furyl); 113.4 (CH-3-furyl); 121.9 (CH-4-benzofuryl); 123.4 (CH-5-benzofuryl); 125.6 (CH-6-benzofuryl); 128.3 (C-3a-benzofuryl); 128.9 (CH-6); 146.2 (CH-5-furyl); 146.4 (C-4); 150.0 (C-2); 152.4 (C-2-furyl); 153.1 (C-7a); 154.7 (C-2-benzofuryl); 154.9 (C-7a-benzofuryl). HR-ESI-MS: *found*: 302.0922 ([M + H]$^+$, calcd for $C_{18}H_{12}O_2N_3^+$: 302.0924); HR-ESI-MS: *found*: 324.0741 ([M + Na]$^+$, calcd for $C_{18}H_{11}O_2N_3Na^+$: 324.0744).

4-(Furan-2-yl)-2-(naphth-1-yl)-7*H*-pyrrolo[2,3-*d*]pyrimidine (5ac)

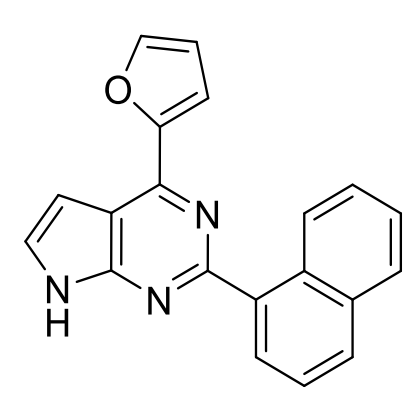

GP D using crude compound **4ac** (131.8 mg, 0.30 mmol). RP-HPFC (C-18, H_2O/MeOH 0 → 100 %) gave **5ac** (87.4 mg, 93 %, over two steps) as a pale-yellow solid. ^{1}H NMR (500 MHz, DMSO-d$_6$): 6.80 (dd, 1H, $J_{4,3}$ = 3.5 Hz, $J_{4,5}$ = 1.7 Hz, H-4-furyl); 7.04 (d, 1H, $J_{5,6}$ = 3.5 Hz, H-5); 7.47 (dd, 1H, $J_{3,4}$ = 3.5 Hz, $J_{3,5}$ = 0.8 Hz, H-3-furyl); 7.52–7.59 (m, 2H, H-6,7-naphthyl); 7.65 (dd, 1H, $J_{3,2}$ and $J_{3,4}$ = 8.2 and 7.1 Hz, H-3-naphthyl); 7.71 (d, 1H, $J_{6,5}$ = 3.5 Hz, H-6); 8.00–8.10 (m, 4H, H-2,4,5-naphthyl, H-5-furyl); 8.73 (m, 1H, H-8-napthtyl); 12.35 (bs, 1H, NH); ^{13}C NMR (125.7 MHz, DMSO-d$_6$): 100.3 (C-5); 110.1 (C-4a); 112.6 (CH-4-furyl); 112.9 (CH-3-furyl); 125.3 (CH-3-naphthyl); 125.8 (CH-6-naphthyl); 126.2 (CH-8-naphthyl); 126.4 (CH-7-naphthyl); 128.3 (CH-5-naphthyl, CH-6); 128.7 and 129.2 (CH-2,4-naphthyl); 130.8 (C-8a-naphthyl); 133.6 (C-4a-

naphthyl); 136.9 (C-1-naphthyl); 146.1 (CH-5-furyl); 146.2 (C-4); 153.0 (C-2-furyl); 153.5 (C-7a); 158.6 (C-2). HR-ESI-MS: *found*: 311.1047 ([M]$^+$, calcd for $C_{20}H_{13}ON_3^+$: 311.1053).

4-(Furan-2-yl)-2-(naphthalen-2-yl)-7*H*-pyrrolo[2,3-*d*]pyrimidine (5ad)

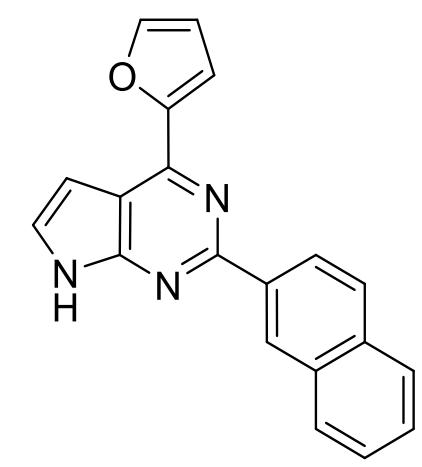

GP D using crude compound **4ad** (82.1 mg, 0.19 mmol). RP-HPFC (C-18, H_2O/MeOH 0 → 100 %) gave **5ad** (55.0 mg, 45 %, over two steps) as a yellowish solid. ^{1}H NMR (500 MHz, DMSO-d$_6$): 6.84 (dd, 1H, $J_{4,3}$ = 3.4 Hz, $J_{4,5}$ = 1.7 Hz, H-4-furyl); 6.99 (d, 1H, $J_{5,6}$ = 3.4 Hz, H-5); 7.54–7.61 (m, 2H, H-6,7-naphthyl); 7.677 (d, 1H, $J_{6,5}$ = 3.4 Hz, H-6); 7.682 (bd, 1H, $J_{3,4}$ = 3.4 Hz, H-3-furyl); 7.98 (m, 1H, H-5-naphthyl); 8.05 (d, 1H, $J_{4,3}$ = 8.6 Hz, H-4-naphthyl); 8.10 (bd, 1H, $J_{5,4}$ = 1.7 Hz, H-5-furyl); 8.12 (m, 1H, H-8-naphthyl); 8.67 (dd, 1H, $J_{3,4}$ = 8.6 Hz, $J_{3,1}$ = 1.7 Hz, H-3-naphthyl); 9.07 (bd, 1H, $J_{1,3}$ = 1.7 Hz, H-1-naphthyl); ^{13}C NMR (125.7 MHz, DMSO-d$_6$): 100.5 (CH-5); 110.8 (C-4a); 112.6 (CH-4-furyl); 112.2 (CH-3-furyl); 125.1 (CH-3-naphthyl); 126.4 and 126.8 (CH-6,7-naphthyl); 127.0 (CH-1-naphthyl); 127.6 (CH-5-naphthyl); 127.9 (CH-4-naphthyl); 128.6 (CH-6); 128.9 (CH-8-naphthyl); 133.0 (C-8a-naphthyl); 133.7 (C-4a-naphthyl); 135.9 (C-2-naphthyl); 146.0 (CH-5-furyl); 146.3 (C-4); 153.0 (C-2-furyl); 153.9 (C-7a); 156.1 (C-2). HR-ESI-MS: *found*: 311.1050 ([M + H]$^+$, calcd for $C_{20}H_{14}ON_3^+$: 311.1053).

4-(Benzofuran-2-yl)-2-(furan-2-yl)-7*H*-pyrrolo[2,3-*d*]pyrimidine (5ba)

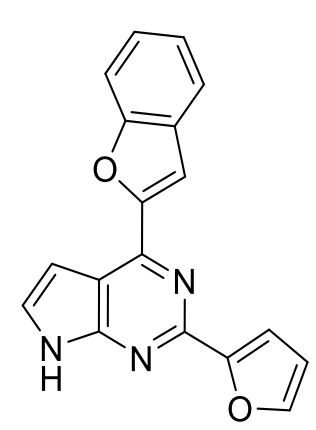

GP D using crude compound **4ba** (106.4 mg, 0.25 mmol). RP-HPFC (C-18, H_2O/MeOH 0 → 100 %) gave **5ba** (46.9 mg, 40 %, over two steps) as a yellow solid. ^{1}H NMR (500 MHz, DMSO-d$_6$: 6.71 (dd, 1H, $J_{4,3}$ = 3.4 Hz, $J_{4,5}$ = 1.8 Hz, H-4-furyl); 7.16 (d, 1H, $J_{5,6}$ = 3.5 Hz, H-5); 7.32 (dd, 1H, $J_{3,4}$ = 3.4 Hz, $J_{3,5}$ = 0.9 Hz, H-3-furyl); 7.37 (td, 1H, $J_{5,4}$ = $J_{5,6}$ = 7.5 Hz, $J_{5,7}$ = 0.9 Hz, H-5-benzofuryl); 7.48 (ddd, 1H, $J_{6,7}$ = 8.4 Hz, $J_{6,5}$ = 7.2 Hz, $J_{6,4}$ = 1.0 Hz, H-6-benzofuryl); 7.73 (d, 1H, $J_{6,5}$ = 3.5 Hz, H-6); 7.827 (dq, 1H, $J_{7,6}$ = 8.4 Hz, $J_{7,5}$ = $J_{7,4}$ = $J_{7,3}$ = 0.9 Hz, H-7-benzofuryl); 7.834 (dm, 1H, $J_{4,5}$ = 7.8 Hz, H-4-benzofuryl); 7.90 (dd, 1H, $J_{5,4}$ = 1.8 Hz, $J_{5,3}$ = 0.9 Hz, H-5-

furyl); 7.98 (d, 1H, $J_{3,7}$ = 1.0 Hz, H-3-benzofuryl); 12.37 (vbs, 1H, NH); ^{13}C NMR (125.7 MHz, DMSO-d$_6$): 101.0 (C-5); 108.8 (CH-3-benzofuryl); 111.5 (CH-3-furyl); 111.6 (C-4a); 111.9 (CH-7-benzofuryl); 112.2 (CH-4-furyl); 122.4 (CH-4-benzofuryl); 123.7 (CH-5-benzofuryl); 126.4 (CH-6-benzofuryl); 127.9 (C-3a-benzofuryl); 128.9 (CH-6); 144.6 (CH-5-furyl); 146.1 (C-4); 150.3 (C-2); 152.8 (C-2-furyl); 153.6 (C-7a); 154.3 (C-2-benzofuryl); 155.3 (C-7a-benzofuryl). HR-ESI-MS: *found*: 301.0841 ([M]$^+$, calcd for $C_{18}H_{11}O_2N_3^+$: 301.0846).

2,4-Di(benzofuran-2-yl)-7*H*-pyrrolo[2,3-*d*]pyrimidine (5bb)

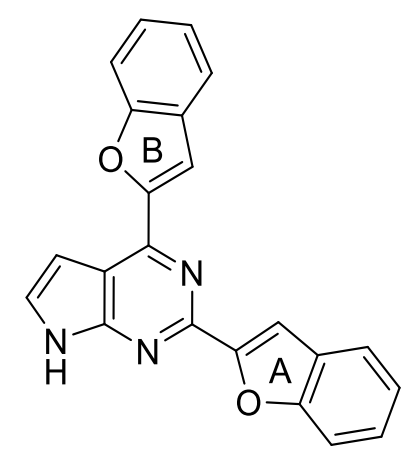

GP D using crude compound **4bb** (124.4 mg, 0.26 mmol). HPFC (SiO_2, DCM/MeOH 0 → 5 %) gave **5bb** (43.5 mg, 48 %, over two steps) as a pale-yellow solid. ^{1}H NMR (500 MHz, DMSO-d$_6$): 7.22 (bd, 1H, $J_{5,6}$ = 3.5 Hz, H-5); 7.34 (bbq, 1H, $J_{5,4}$ = $J_{5,6}$ = 7.5 Hz, $J_{5,7}$ = 1.0 Hz, H-5-benzofuryl-*A*); 7.39 (btd, 1H, $J_{5,4}$ = $J_{5,6}$ = 7.5 Hz, $J_{5,7}$ = 1.0 Hz, H-5-benzofuryl-*B*); 7.43 (ddd, 1H, $J_{6,7}$ = 8.3 Hz, $J_{6,5}$ = 7.2 Hz, $J_{6,4}$ = 1.4 Hz, H-6-benzofuryl-*A*); 7.50 (ddd, 1H, $J_{6,7}$ = 8.3 Hz, $J_{6,5}$ = 7.2 Hz, $J_{6,4}$ = 1.3 Hz, H-6-benzofuryl-*B*); 7.75 (dm, 1H, $J_{7,6}$ = 8.2 Hz, H-7-benzofuryl-*A*); 7.80 (m, 1H, H-4-benzofuryl-*A*); 7.81 (d, 1H, $J_{3,LR}$ = 1.0 Hz, H-3-benzofuryl-*A*); 7.83 (dd, 1H, $J_{6,5}$ = 3.5 Hz, $J_{6,NH}$ = 1.5 Hz, H-6); 7.85 (m, 1H, H-7-benzofuryl-*B*); 7.86 (m, 1H, H-4-benzofuryl-*B*); 8.07 (d, 1H, $J_{3,LR}$ = 0.9 Hz, H-3-benzofuryl-*B*); 12.51 (bs, 1H, NH); ^{13}C NMR (125.7 MHz, DMSO-d$_6$): 101.2 (CH-5); 107.5 (CH-3-benzofuryl-*A*); 109.1 (CH-3-benzofuryl-*B*); 111.6 (CH-7-benzofuryl-*A*); 111.9 (CH-7-benzofuryl-*B*); 112.2 (C-4a); 122.0 (CH-4-benzofuryl-*A*); 122.5 (CH-4-benzofuryl-*B*); 123.5 (CH-5-benzofuryl-*A*); 123.8 (CH-5-benzofuryl-*B*); 125.7 (CH-6-benzofuryl-*A*); 126.5 (CH-6-benzofuryl-*B*); 127.9 (C-3a-benzofuryl-*B*); 128.3 (C-3a-benzofuryl-*A*); 129.6 (CH-6); 146.3 (C-4); 150.0 (C-2); 153.4 (C-7a); 154.1 (C-2-benzofuryl-*B*); 154.6 (C-2-benzofuryl-*A*); 154.9 (C-7a-benzofuryl-*A*); 155.3 (C-7a-benzofuryl-*B*). HR-ESI-MS: *found*: 352.1080 ([M + H]$^+$, calcd for $C_{22}H_{14}O_2N_3^+$: 352.1081); HR-ESI-MS: *found*: 374.0899 ([M + Na]$^+$, calcd for $C_{22}H_{13}O_2N_3Na^+$: 374.0900).

4-(Benzofuran-2-yl)-2-(naphth-1-yl)-7*H*-pyrrolo[2,3-*d*]pyrimidine (5bc)

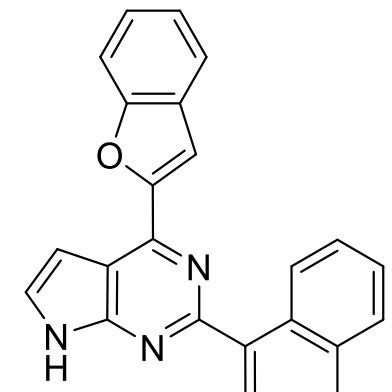

GP D using crude compound **4bc** (45.6 mg, 0.09 mmol). RP-HPFC (C-18, H_2O/MeOH 0 → 100 %) gave **5bc** (30.2 mg, 42 %, over two steps) as a white solid. ^{1}H NMR (500 MHz, DMSO-d$_6$): 7.23 (d, 1H, $J_{5,6}$ = 3.5 Hz, H-5); 7.36 (btd, 1H, $J_{5,4}$ = $J_{5,6}$ = 7.5 Hz, $J_{5,7}$ = 0.8 Hz, H-5-benzofuryl); 7.47 (ddd, 1H, $J_{6,7}$ = 8.3 Hz, $J_{6,5}$ = 7.2 Hz, $J_{6,4}$ = 1.3 Hz, H-6-benzofuryl); 7.55–7.60 (m, 2H, H-6,7-naphthyl); 7.68 (dd, 1H, $J_{3,4}$ = 8.2 Hz, $J_{3,1}$ = 7.2 Hz, H-3-naphthyl); 7.81 (d, 1H, $J_{6,5}$ = 3.5 Hz, H-6); 7.81 – 7.85 (m, 2H, H-4,7-benzofuryl); 7.93 (d, 1H, $J_{3,LR}$ = 0.9 Hz, H-3-benzofuryl); 8.04 (m, 1H, H-5-naphthyl); 8.07 (d, 1H, $J_{4,3}$ = 8.2 Hz, H-4-naphthyl); 8.13 (dd, 1H, $J_{2,3}$ = 7.2 Hz, $J_{2,4}$ = 1.3 Hz, H-2-naphthyl); 8.78 (m, 1H, H-8-naphthyl); ^{13}C NMR (125.7 MHz, DMSO-d$_6$): 100.5 (CH-5); 108.5 (CH-3-benzofuryl); 111.5 (C-4a); 111.9 (CH-7-benzofuryl); 122.4 (CH-4-benzofuryl); 123.7 (CH-5-benzofuryl); 125.3 (CH-3-naphthyl); 125.8, 126.26, 126.27 and 126.5 (CH-6-benzofuryl, CH-6,7,8-naphthyl); 128.0 (C-3a-benzofuryl); 128.3 (CH-5-naphthyl); 128.8 (CH-2-naphthyl); 129.3 (CH-4-naphthyl); 129.7 (CH-6); 130.9 (C-8a-naphthyl); 133.7 (C-4a-naphthyl); 136.9 (C-1-naphthyl); 145.8 (C-4); 154.2 (C-7a); 154.8 (C-2-benzofuryl); 155.3 (C-7a-benzofuryl); 158.4 (C-2). HR-ESI-MS: *found*: 362.1290 ([M + H]$^+$, calcd for $C_{24}H_{16}ON_3^+$: 362.1288); HR-ESI-MS: *found*: 384.1110 ([M + Na]$^+$, calcd for $C_{24}H_{15}ON_3Na^+$: 384.1107).

4-(Benzofuran-2-yl)-2-(naphthalen-2-yl)-7*H*-pyrrolo[2,3-*d*]pyrimidine (5bd)

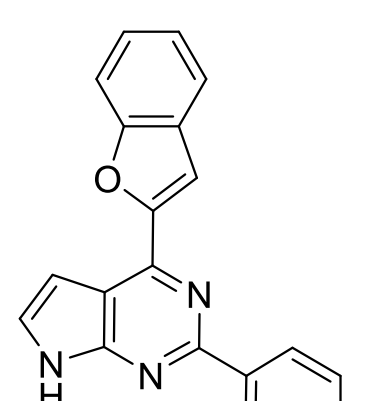

GP D using crude compound **4bd** (33.0 mg, 0.07 mmol). RP-HPFC (C-18, H_2O/MeOH 0 → 100 %) gave **5bd** (15.1 mg, 7 %, over two steps) as a pale-yellow solid. ^{1}H NMR (500 MHz, DMSO-d$_6$): 7.20 (d, 1H, $J_{5,6}$ = 3.5 Hz, H-5); 7.39 (m, 1H, H-5-benzofuryl); 7.50 (ddd, 1H, $J_{6,7}$ = 8.4 Hz, $J_{6,5}$ = 7.2 Hz, $J_{6,4}$ = 1.3 Hz, H-6-benzofuryl); 7.57–7.62 (m, 2H, H-6,7-naphthyl); 7.78 (d, 1H, $J_{6,5}$ = 3.5 Hz, H-6); 7.84–7.89 (m, 2H, H-4,7-benzofuryl); 8.01 (m, 1H, H-5-naphthyl); 8.08 (d, 1H, $J_{4,3}$ = 8.7 Hz, H-4-naphthyl); 8.15 (m, 1H, H-8-naphthyl); 8.16 (d, 1H, $J_{3,LR}$ = 1.0 Hz, H-

3-benzofuryl); 8.72 (dd, 1H, $J_{3,4}$ = 8.6 Hz, $J_{3,1}$ = 1.7 Hz, H-3-naphthyl); 9.13 (d, 1H, $J_{1,3}$ = 1.7 Hz, H-1-naphthyl); ^{13}C NMR (125.7 MHz, DMSO-d_6): 100.8 (CH-5); 108.7 (CH-3-benzofuryl); 111.9 (CH-7-benzofuryl); 112.0 (C-4a); 122.4 (CH-4-benzofuryl); 123.8 (CH-5-benzofuryl); 125.1 (CH-3-naphthyl); 126.3 (CH-6-benzofuryl); 126.5 and 126.9 (CH-6,7-naphthyl); 127.1 (CH-1-naphthyl); 127.7 (CH-5-naphthyl); 127.95 (C-3a-benzofuryl); 128.00 (CH-4-naphthyl); 128.9 (CH-8-naphthyl); 129.4 (CH-6); 133.02 (C-8a-naphthyl); 133.8 (C-4a-naphthyl); 135.8 (C-2-naphthyl); 146.0 (C-4); 154.3 (C-2-benzofuryl); 154.8 (C-7a); 155.4 (C-7a-benzofuryl); 156.2 (C-2). HR-ESI-MS: *found*: 362.1289 ([M + H]$^+$, calcd for $C_{24}H_{16}ON_3^+$: 362.1288); HR-ESI-MS: *found*: 384.1108 ([M + Na]$^+$, calcd for $C_{24}H_{15}ON_3Na^+$: 384.1107).

2-(Furan-2-yl)-4-(naphth-1-yl)-7*H*-pyrrolo[2,3-*d*]pyrimidine (5ca)

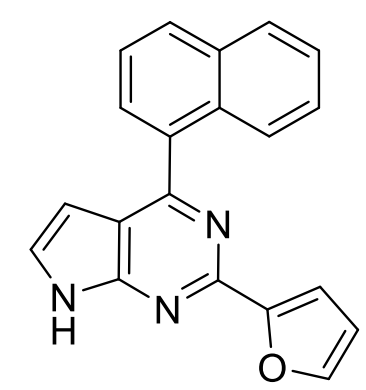

GP D using crude compound **4ca** (51.3 mg, 0.12 mmol). RP-HPFC (C-18, H_2O/MeOH 0 → 100 %) gave **5ca** (34.4 mg, 38 %, over two steps) as a white solid. ^{1}H NMR (500 MHz, DMSO-d_6): 6.30 (d, 1H, $J_{5,6}$ = 3.5 Hz, H-5); 6.67 (dd, 1H, $J_{4,3}$ = 3.4 Hz, $J_{4,5}$ = 1.8 Hz, H-4-furyl); 7.19 (dd, 1H, $J_{3,4}$ = 3.4 Hz, $J_{3,5}$ = 0.9 Hz, H-3-furyl); 7.51 (ddd, 1H, $J_{7,8}$ = 8.5 Hz, $J_{7,6}$ = 6.8 Hz, $J_{7,5}$ = 1.4 Hz, H-7-naphthyl); 7.59 (ddd, 1H, $J_{6,5}$ = 8.2 Hz, $J_{6,7}$ = 6.8 Hz, $J_{6,8}$ = 1.3 Hz, H-6-naphthyl); 7.60 (d, 1H, $J_{6,5}$ = 3.5 Hz, H-6); 7.70 (dd, 1H, $J_{3,4}$ = 8.2 Hz, $J_{3,2}$ = 7.0 Hz, H-3-naphthyl); 7.81 (dd, 1H, $J_{2,3}$ = 7.0 Hz, $J_{2,4}$ = 1.2 Hz, H-2-naphthyl); 7.86 (dd, 1H, $J_{5,4}$ = 1.8 Hz, $J_{5,3}$ = 0.9 Hz, H-5-furyl); 8.07 (bd, 1H, $J_{5,6}$ = 8.2 Hz, H-5-naphthyl); 8.09 (bd, 1H, $J_{8,7}$ = 8.5 Hz, H-8-naphthyl); 8.12 (bd, 1H, $J_{4,3}$ = 8.2 Hz, H-4-naphthyl); ^{13}C NMR (125.7 MHz, DMSO-d_6): 100.2 (CH-5); 111.2 (CH-3-furyl); 112.2 (CH-4-furyl); 115.8 (C-4a); 125.4 (CH-3-naphthyl); 125.5 (CH-8-naphthyl); 126.2 (CH-6-naphthyl); 126.6 (CH-7-naphthyl); 127.9 (CH-2-naphthyl); 128.2 (CH-6); 128.39 (CH-5-naphthyl); 129.6 (CH-4-naphthyl); 130.5 (C-8a-naphthyl); 133.6 (C-4a-naphthyl); 135.0 (C-1-naphthyl); 144.5 (CH-5-furyl); 150.2 (C-2); 152.7 (C-7a); 153.2 (C-2-furyl); 157.7 (C-4). HR-ESI-MS: *found*: 312.1132 ([M + H]$^+$, calcd for $C_{20}H_{14}ON_3^+$: 312.1131); HR-ESI-MS: *found*: 334.0951 ([M + Na]$^+$, calcd for $C_{20}H_{13}ON_3Na^+$: 334.0951).

2-(Benzofuran-2-yl)-4-(naphth-1-yl)-7*H*-pyrrolo[2,3-*d*]pyrimidine (5cb)

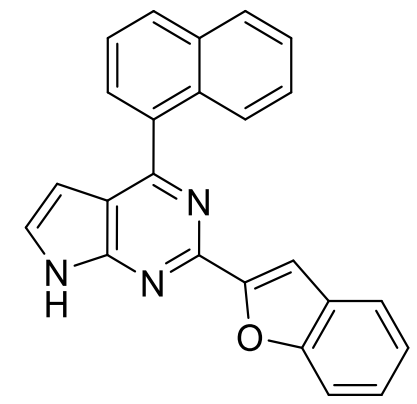

GP D using crude compound **4cb** (121.6 mg, 0.25 mmol). RP-HPFC (C-18, H_2O/MeOH 0 → 100 %) gave **5cb** (67.8 mg, 74 %, over two steps) as a white solid. ^{1}H NMR (500 MHz, DMSO-d$_6$): 6.36 (d, 1H, $J_{5,6}$ = 3.4 Hz, H-5); 7.30 (btd, 1H, $J_{5,4}$ = $J_{5,6}$ = 7.5 Hz, $J_{5,7}$ = 0.9 Hz, H-5-benzofuryl); 7.39 (ddd, 1H, $J_{6,7}$ = 8.3 Hz, $J_{6,5}$ = 7.2 Hz, $J_{6,4}$ = 1.4 Hz, H-6-benzofuryl); 7.53 (ddd, 1H, $J_{7,8}$ = 8.5 Hz, $J_{7,6}$ = 6.8 Hz, $J_{7,5}$ = 1.4 Hz, H-7-naphthyl); 7.60 (ddd, 1H, $J_{6,5}$ = 8.2 Hz, $J_{6,7}$ = 6.8 Hz, $J_{6,8}$ = 1.2 Hz, H-6-naphthyl); 7.65 (d, 1H, $J_{3,LR}$ = 1.0 Hz, H-3-benzofuryl); 7.69 (d, 1H, $J_{6,5}$ = 3.4 Hz, H-6); 7.69–7.76 (m, 3H, H-4,7-benzofuryl, H-3-naphthyl); 7.86 (dd, 1H, $J_{2,3}$ = 7.1 Hz, $J_{2,4}$ = 1.3 Hz, H-2-naphthyl); 8.09 (bd, 1H, $J_{5,6}$ = 8.2 Hz, H-5-naphthyl); 8.13 (bd, 1H, $J_{8,7}$ = 8.5 Hz, H-8-naphthyl); 8.14 (bd, 1H, $J_{4,3}$ = 8.2 Hz, H-4-naphthyl); ^{13}C NMR (125.7 MHz, DMSO-d$_6$): 100.3 (CH-5); 107.0 (CH-3-benzofuryl); 111.6 (CH-7-benzofuryl); 116.5 (C-4a); 121.90 (CH-4-benzofuryl); 123.4 (CH-5-benzofuryl); 125.4, 125.5 and 125.6 (CH-3,8-naphthyl, CH-6-benzofuryl); 126.2 (CH-6-naphthyl); 126.7 (CH-7-naphthyl); 128.0 (CH-2-naphthyl); 128.4 (C-3a-benzofuryl); 128.4 (CH-5-naphthyl); 129.4 (CH-6); 129.6 (CH-4-naphthyl); 130.5 (C-8a-naphthyl); 133.6 (C-4a-naphthyl); 135.0 (C-1-naphthyl); 149.7 (C-2); 152.8 (C-7a); 154.9 and 155.1 (C-2,7a-benzofuryl); 157.7 (C-4). HR-ESI-MS: *found*: 362.1287 ([M + H]$^+$, calcd for $C_{24}H_{16}ON_3^+$: 362.1288); HR-ESI-MS: *found*: 384.1107 ([M + Na]$^+$, calcd for $C_{24}H_{15}ON_3Na^+$: 384.1107).

2,4-Bis(naphth-1-yl)-7*H*-pyrrolo[2,3-*d*]pyrimidine (5cc)

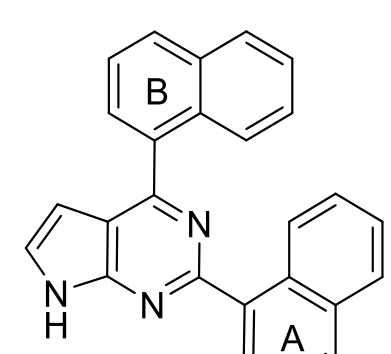

GP D using crude compound **4cc** (54.3 mg, 0.11 mmol). RP-HPFC (C-18, H_2O/MeOH 0 → 100 %) gave **5cc** (28.2 mg, 15 %, over two steps) as a grey solid. ^{1}H NMR (500 MHz, DMSO-d$_6$): 6.39 (d, 1H, $J_{5,6}$ = 3.5 Hz, H-5); 7.52 (ddd, 1H, $J_{7,8}$ = 8.5 Hz, $J_{7,6}$ = 6.8 Hz, $J_{7,5}$ = 1.4 Hz, H-7-naphthyl-*A*); 7.52–7.57 (m, 2H, H-6-naphthyl-*A*, H-7-naphthyl-*B*); 7.58 (ddd, 1H, $J_{6,5}$ = 8.2 Hz, $J_{6,7}$ = 6.8 Hz, $J_{6,8}$ = 1.3 Hz, H-6-naphthyl-*B*); 7.64 (dd, 1H, $J_{3,4}$ = 8.2 Hz, $J_{3,2}$ = 7.2 Hz, H-3-naphthyl-*A*);

7.68 (d, 1H, $J_{6,5}$ = 3.5 Hz, H-6); 7.71 (dd, 1H, $J_{3,4}$ = 8.2 Hz, $J_{3,2}$ = 7.1 Hz, H-3-naphthyl-*B*); 7.89 (dd, 1H, $J_{2,3}$ = 7.1 Hz, $J_{2,4}$ = 1.3 Hz, H-2-naphthyl-*B*); 8.01 (m, 1H, H-5-naphthyl-*A*); 8.03 (bd, 1H, $J_{4,3}$ = 8.2 Hz, H-4-naphthyl-*A*); 8.07 (bd, 1H, $J_{5,6}$ = 8.2 Hz, H-5-naphthyl-*B*); 8.116 (dd, 1H, $J_{2,3}$ = 7.2 Hz, $J_{2,4}$ = 1.3 Hz, H-2-naphthyl-*A*); 8.123 (bd, 1H, $J_{4,3}$ = 8.2 Hz, H-4-naphthyl-*B*); 8.17 (bd, 1H, $J_{8,7}$ = 8.5 Hz, H-8-naphthyl-*B*); 8.81 (m, 1H, H-8-naphthyl-*A*); ^{13}C NMR (125.7 MHz, DMSO-d$_6$): 99.8 (CH-5); 115.6 (C-4a); 125.3 (CH-3-naphthyl-*A*); 125.5 (CH-3-naphthyl-*B*); 125.7 (CH-8-naphthyl-*B*); 125.8 (CH-7-naphthyl-*A*); 126.2 (CH-6-naphthyl-*B*); 126.3 and 126.4 (CH-6,8-naphthyl-*A*); 126.6 (CH-7-naphthyl-*B*); 128.1 (CH-2-naphthyl-*B*); 128.3 (CH-5-naphthyl-*A*); 128.4 (CH-5-naphthyl-*B*); 128.4 (CH-6); 128.9 (CH-2-naphthyl-*A*); 129.2 (CH-4-naphthyl-*A*); 129.5 (CH-4-naphthyl-*B*); 130.5 (C-8a-naphthyl-*B*); 130.8 (C-8a-naphthyl-*A*); 133.58 and 133.63 (C-4a-naphthyl-*A*,*B*); 135.4 (C-1-naphthyl-*B*); 137.2 (C-1-naphthyl-*A*); 153.0 (C-7a); 157.54 (C-4); 158.4 (C-2). HR-ESI-MS: *found*: 372.1496 ($[M + H]^+$, calcd for $C_{26}H_{18}N_3^+$: 372.1495); HR-ESI-MS: *found*: 394.1316 ($[M + Na]^+$, calcd for $C_{26}H_{17}N_3Na^+$: 394.1315).

4-(Naphth-1-yl)-2-(naphthalen-2-yl)-7*H*-pyrrolo[2,3-*d*]pyrimidine (5cd)

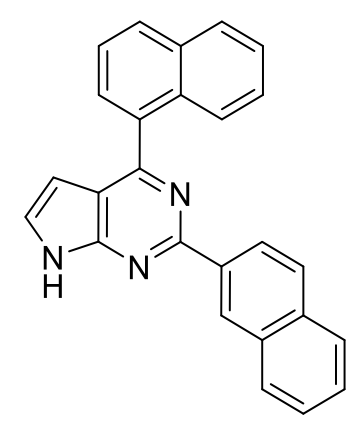

GP D using crude compound **4cd** (46.5 mg, 0.09 mmol). RP-HPFC (C-18, H_2O/MeOH 0 → 100 %) gave **5cd** (31.6 mg, 32 %, over two steps) as a white solid. ^{1}H NMR (500 MHz, DMSO-d$_6$): 6.36 (d, 1H, $J_{5,6}$ = 3.5 Hz, H-5); 7.52–7.59 (m, 3H, H-6,7-naphthyl-*A*, H-7-naphthyl-*B*); 7.61 (ddd, 1H, $J_{6,5}$ = 8.2 Hz, $J_{6,7}$ = 6.8 Hz, $J_{6,8}$ = 1.3 Hz, H-6-naphthyl-*B*); 7.65 (d, 1H, $J_{6,5}$ = 3.5 Hz, H-6); 7.74 (dd, 1H, $J_{3,4}$ = 8.2 Hz, $J_{3,2}$ = 7.1 Hz, H-3-naphthyl-*B*); 7.90 (dd, 1H, $J_{2,3}$ = 7.1 Hz, $J_{2,4}$ = 1.3 Hz, H-2-naphthyl-*B*); 7.97 (m, 1H, H-5-naphthyl-*A*); 8.04 (d, 1H, $J_{4,3}$ = 8.8 Hz, H-4-naphthyl-*A*); 8.07 (m, 1H, H-8-naphthyl-*A*); 8.10 (bd, 1H, $J_{5,6}$ = 8.3 Hz, H-5-naphthyl-*B*); 8.15 (bd, 1H, $J_{4,3}$ = 8.3 Hz, H-4-naphthyl-*B*); 8.20 (bd, 1H, $J_{8,7}$ = 8.5 Hz, H-8-naphthyl-*B*); 8.63 (dd, 1H, $J_{3,4}$ = 8.7 Hz, $J_{3,1}$ = 1.7 Hz, H-3-naphthyl-*A*); 9.02 (bd, 1H, $J_{1,3}$ = 1.5 Hz, H-1-naphthyl-*A*); ^{13}C NMR (125.7 MHz, DMSO-d$_6$): 100.1 (CH-5); 116.2 (C-4a); 125.1 (CH-3-naphthyl-*A*); 125.5 (CH-3-naphthyl-*B*); 125.6 (CH-8-naphthyl-*B*); 126.2 (CH-6-naphthyl-*B*); 126.4 (CH-7-naphthyl-*A*); 126.7 and 126.8 (CH-7-naphthyl-*B*; CH-6-naphthyl-*A*); 126.9 (CH-1-naphthyl-*A*); 127.6 (CH-5-naphthyl-*A*); 128.00 and 128.02 (CH-2-naphthyl-*B*, CH-naphthyl-4-*A*); 128.4 (CH-5-naphthyl-*B*); 128.5 (CH-

6); 128.8 (CH-8-naphthyl-*A*); 129.6 (CH-4-naphthyl-*B*); 130.6 (C-8a-naphthyl-*B*); 133.0 (C-8a-naphthyl-*A*); 133.65 and 133.68 (C-4a-naphthyl-*A*,*B*); 135.4 (C-1-naphthyl-*B*); 136.3 (C-2-naphthyl-*A*); 153.4 (C-7a); 156.1 (C-2); 157.6 (C-4). HR-ESI-MS: *found*: 372.1492 ($[M + H]^+$, calcd for $C_{26}H_{18}N_3^+$: 372.1495); HR-ESI-MS: *found*: 394.1312 ($[M + Na]^+$, calcd for $C_{26}H_{17}N_3Na^+$: 394.1315).

2-(Furan-2-yl)-4-(naphthalen-2-yl)-7*H*-pyrrolo[2,3-*d*]pyrimidine (5da)

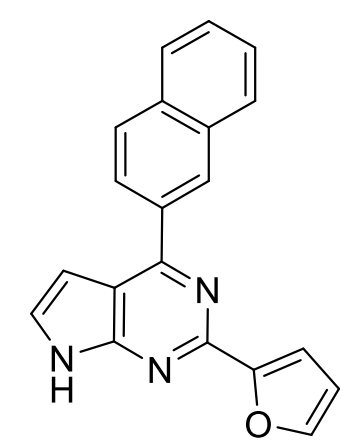

GP D using crude compound **4da** (55.0 mg, 0.13 mmol). RP-HPFC (C-18, H_2O/MeOH 0 → 100 %) gave **5da** (31.3 mg, 19 %, over two steps) as a pale-yellow solid. ^{1}H NMR (500 MHz, DMSO-d$_6$): 6.71 (dd, 1H, $J_{4,3}$ = 3.3 Hz, $J_{4,5}$ = 1.7 Hz, H-4-furyl); 7.07 (d, 1H, $J_{5,6}$ = 3.5 Hz, H-5); 7.33 (dd, 1H, $J_{3,4}$ = 3.3 Hz, $J_{3,5}$ = 0.9 Hz, H-3-furyl); 7.58–7.66 (m, 2H, H-6,7-naphthyl); 7.71 (d, 1H, $J_{6,5}$ = 3.5 Hz, H-6); 7.90 (dd, 1H, $J_{5,4}$ = 1.7 Hz, $J_{5,3}$ = 0.9 Hz, H-5-furyl); 8.03 (m, 1H, H-5-naphthyl); 8.13 (d, 1H, $J_{4,3}$ = 8.6 Hz, H-4-naphthyl); 8.21 (m, 1H, H-8-naphthyl); 8.42 (dd, 1H, $J_{3,4}$ = 8.6 Hz, $J_{3,1}$ = 1.8 Hz, H-3-naphthyl); 8.82 (d, 1H, $J_{1,3}$ = 1.7 Hz, H-1-naphthyl); ^{13}C NMR (125.7 MHz, DMSO-d$_6$): 100.6 (CH-5); 111.2 (CH-3-furyl); 112.2 (CH-4-furyl); 113.3 (C-4a); 125.7 (CH-3-naphthyl); 126.6, 127.33 and 127.6 (CH-5,6,7-naphthyl); 128.3 (CH-4-naphthyl); 128.6 (CH-1-naphthyl); 128.8 (CH-6); 129.1 (CH-8-naphthyl); 132.9 (C-8a-naphthyl); 133.7 (C-4a-naphthyl); 135.3 (C-2-naphthyl); 144.4 (CH-5-furyl); 150.2 (C-2); 153.2 (C-2-furyl); 153.5 (C-7a); 155.4 (C-4). HR-ESI-MS: *found*: 312.1133 ($[M + H]^+$, calcd for $C_{20}H_{14}ON_3^+$: 312.1131); HR-ESI-MS: *found*: 334.0952 ($[M + Na]^+$, calcd for $C_{20}H_{13}ON_3Na^+$: 334.0951).

2-(Benzofuran-2-yl)-4-(naphthalen-2-yl)-7*H*-pyrrolo[2,3-*d*]pyrimidine (5db)

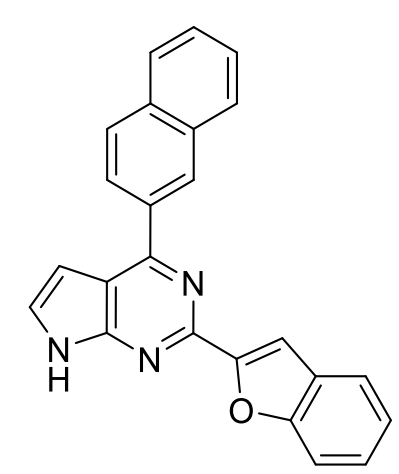

GP D using crude compound **4db** (124.2 mg, 0.25 mmol). RP-HPFC (C-18, H_2O/MeOH 0 → 100 %) gave **5db** (79.5 mg, 85 %, over two steps) as a pale-yellow solid. ^{1}H NMR (500 MHz, DMSO-

d6): 7.13 (d, 1H, $J_{5,6}$ = 3.5 Hz, H-5); 7.33 (btd, 1H, $J_{5,4}$ = $J_{5,6}$ = 7.5 Hz, $J_{5,7}$ = 1.0 Hz, H-5-benzofuryl); 7.42 (ddd, 1H, $J_{6,7}$ = 8.2 Hz, $J_{6,5}$ = 7.2 Hz, $J_{6,4}$ = 1.3 Hz, H-6-benzofuryl); 7.61–7.67 (m, 2H, H-6,7-naphthyl); 7.75 (dm, 1H, $J_{7,6}$ = 8.2 Hz, H-7-benzofuryl); 7.79 (m, 1H, H-4-benzofuryl); 7.81 (d, 1H, $J_{6,5}$ = 3.5 Hz, H-6); 7.83 (d, 1H, $J_{3,LR}$ = 1.0 Hz, H-3-benzofuryl); 8.05 (m, 1H, H-5-naphthyl); 8.16 (d, 1H, $J_{4,3}$ = 8.6 Hz, H-4-naphthyl); 8.24 (m, 1H, H-8-naphthyl); 8.48 (dd, 1H, $J_{3,4}$ = 8.6 Hz, $J_{3,1}$ = 1.8 Hz, H-3-naphthyl); 8.88 (d, 1H, $J_{1,3}$ = 1.7 Hz, H-1-naphthyl); ^{13}C NMR (125.7 MHz, DMSO-d6): 100.8 (CH-5); 107.1 (CH-3-benzofuryl); 111.9 (CH-7-benzofuryl); 113.1 (C-4a); 121.9 (CH-4-benzofuryl); 123.5 (CH-5-benzofuryl); 125.46 (CH-5-benzofuryl); 125.46 (CH-6-benzofuryl); 125.8 (CH-3-naphthyl); 126.6 (CH-7-naphthyl); 127.4 (CH-6-naphthyl); 127.7 (CH-5-naphthyl); 128.38 (CH-4-naphthyl); 128.44 (C-3a-benzofuryl); 128.7 (CH-1-naphthyl); 129.1 (CH-8-naphthyl); 129.8 (CH-6); 132.9 (C-8a-naphthyl); 133.7 (C-4a-naphthyl); 135.2 (C-2-naphthyl); 149.8 (C-2); 153.5 (C-7a); 154.9 (C-7a-benzofuryl); 155.1 (C-2-benzofuryl); 155.4 (C-4). HR-ESI-MS: *found*: 362.1287 ([M + H]$^+$, calcd for $C_{24}H_{16}ON_3^+$: 362.1288); HR-ESI-MS: *found*: 384.1106 ([M + Na]$^+$, calcd for $C_{24}H_{15}ON_3Na^+$: 384.1107).

2-(Naphth-1-yl)-4-(naphthalen-2-yl)-7*H*-pyrrolo[2,3-*d*]pyrimidine (5dc)

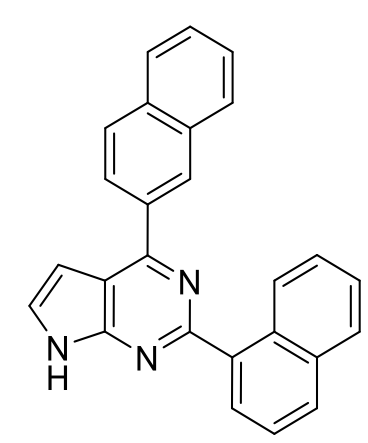

GP D using crude compound **4dc** (120.3 mg, 0.24 mmol). RP-HPFC (C-18, H_2O/MeOH 0 → 100 %) gave **5dc** (85.9 mg, 93 %, over two steps) as a grey solid. ^{1}H NMR (500 MHz, DMSO-d6): 7.15 (d, 1H, $J_{5,6}$ = 3.5 Hz, H-5); 7.55–7.65 (m, 4H, H-6,7-naphthyl-*A*,*B*); 7.69 (dd, 1H, $J_{3,4}$ = 8.2 Hz, $J_{3,2}$ = 7.1 Hz, H-3-naphthyl-*A*); 7.79 (d, 1H, $J_{6,5}$ = 3.5 Hz, H-6); 8.02 and 8.04 (2×m, 2×1H, H-5-naphthyl-*A*,*B*); 8.06 (bd, 1H, $J_{4,3}$ = 8.2 Hz, H-4-naphthyl-*A*); 8.13 (d, 1H, $J_{4,3}$ = 8.6 Hz, H-4-naphthyl-*B*); 8.16 (dd, 1H, $J_{2,3}$ = 7.2 Hz, $J_{2,4}$ = 1.3 Hz, H-2-naphthyl-*A*); 8.20 (m, 1H, H-8-naphthyl-*B*); 8.44 (dd, 1H, $J_{3,4}$ = 8.6 Hz, $J_{3,1}$ = 1.8 Hz, H-3-naphthyl-*B*); 8.81 (m, 1H, H-8-naphthyl-*A*); 8.86 (d, 1H, $J_{1,3}$ = 1.7 Hz, H-1-naphthyl-*B*); ^{13}C NMR (125.7 MHz, DMSO-d6): 100.18 (CH-5); 113.1 (C-4a); 125.4 (CH-3-naphthyl-*A*); 125.8 (CH-3,6-naphthyl-*B*); 126.3 and 126.4 (CH-6,8-naphthyl-*A*); 126.6 and 127.3 (CH-7-naphthyl-*A,B*); 127.6 (CH-5-naphthyl-*A*/*B*), 128.3 and 128.4 (CH-5-naphthyl-*A*/*B*, H-4-naphthyl-*B*); 128.6 (CH-1-naphthyl-*B*); 128.8 (CH-2-naphthyl-*A*); 129.0 (CH-8-naphthyl-*B*); 129.1 (CH-6, CH-4-naphthyl-*A*); 130.9 (C-8a-naphthyl-

A); 132.9 (C-8a-naphthyl-*B*); 133.6 and 133.7 (C-4a-naphthyl-*A*,*B*); 135.8 (C-2-naphthyl-*B*); 137.3 (C-1-naphthyl-*A*); 153.9 (C-7a); 155.2 (C-4); 158.4 (C-2). HR-ESI-MS: *found*: 372.1493 ($[M + H]^+$, calcd for $C_{26}H_{18}N_3^+$: 372.1495); HR-ESI-MS: *found*: 394.1312 ($[M + Na]^+$, calcd for $C_{26}H_{17}N_3Na^+$: 394.1315).

2,4-Di(naphthalen-2-yl)-7*H*-pyrrolo[2,3-*d*]pyrimidine (5dd)

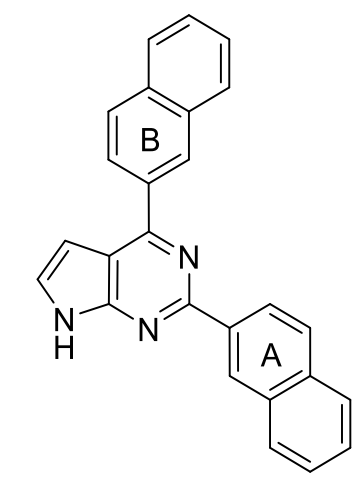

GP D using crude compound **4dd** (52.9 mg, 0.11 mmol). RP-HPFC (C-18, H_2O/MeOH 0 → 100 %) gave **5dd** (37.0 mg, 6 %, over two steps) as a brownish solid. ^{1}H NMR (500 MHz, DMSO-d$_6$): 7.13 (d, 1H, $J_{5,6}$ = 3.5 Hz, H-5); 7.56–7.62 (m, 2H, H-7-naphthyl-*A*, H-6-naphthyl-*B*); 7.61–7.68 (m, 2H, H-7-naphthyl-*B*; H-6-naphthyl-*A*); 7.76 (d, 1H, $J_{6,5}$ = 3.5 Hz, H-6); 8.00 (m, 1H, H-5-naphthyl-*A*); 8.06 (m, 1H, H-5-naphthyl-*B*); 8.09 (d, 1H, $J_{4,3}$ = 8.6 Hz, H-4-naphthyl-*A*); 8.16 (m, 1H, H-8-naphthyl-*A*); 8.19 (d, 1H, $J_{4,3}$ = 8.6 Hz, H-4-naphthyl-*B*); 8.26 (m, 1H, H-8-naphthyl-*B*); 8.56 (dd, 1H, $J_{3,4}$ = 8.6 Hz, $J_{3,1}$ = 1.7 Hz, H-3-naphthyl-*B*); 8.76 (dd, 1H, $J_{3,4}$ = 8.6 Hz, $J_{3,1}$ = 1.7 Hz, H-3-naphthyl-*A*); 8.93 (d, 1H, $J_{1,3}$ = 1.75 Hz, H-1-naphthyl-*B*); 9.15 (d, 1H, $J_{1,3}$ = 1.7 Hz, H-1-naphthyl-*A*); ^{13}C NMR (125.7 MHz, DMSO-d$_6$): 100.6 (CH-5); 113.6 (C-4a); 125.2 (CH-3-naphthyl-*A*); 125.9 (CH-3-naphthyl-*B*); 126.4, 126.6, 126.8, 127.0 and 127.3 (CH-6,7-naphthyl-*A*,*B*); 127.7 (CH-5-naphthyl-*A*,*B*); 128.0 (CH-4-naphthyl-*A*); 128.4 (CH-4-naphthyl-*B*); 128.7 (CH-naphthyl-1-*B*); 128.9 (CH-8-naphthyl-*A*); 128.9 (CH-6); 128.1 (CH-8-naphthyl-*B*); 132.97 (C-8a-naphthyl-*B*); 133.04 (C-8a-naphthyl-*A*); 133.7 (C-4a-naphthyl-*A*,*B*); 135.7 (C-2-naphthyl-*B*); 136.3 (C-2-naphthyl-*A*); 154.1 (C-7a); 155.4 (C-2); 156.2 (C-4). HR-ESI-MS: *found*: 371.1408 ($[M + H]^+$, calcd for $C_{26}H_{18}N_3^+$: 371.1417).

4-(Furan-2-yl)-7-isopropyl-2-(naphth-1-yl)-7*H*-pyrrolo[2,3-*d*]pyrimidine (8ac)

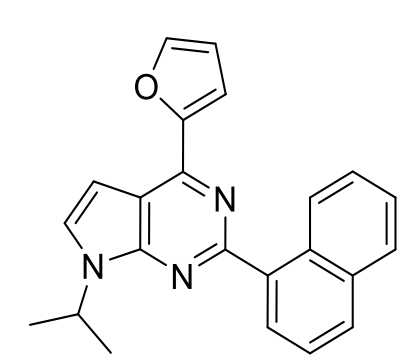

GP B starting from compound **7a** (92.1 mg, 0.35 mmol) and naphthalene-1-boronic acid (181.6 mg, 1.06 mmol). The reaction was performed in microwave reactor at 100 °C for 1 h. HPFC (SiO_2,

cHex/EtOAc 1:0 → 9:1) gave **8ac** (86.6 mg, 70 %) as a yellow sticky solid. ^{1}H NMR (500 MHz, DMSO-d_6): 1.54 (d, 6H, $J_{CH3,CH}$ = 6.8 Hz, (C**H**$_3$)$_2$CH); 5.13 (sept, 6H, $J_{CH,CH3}$ = 6.8 Hz, (CH$_3$)$_2$C**H**); 6.80 (dd, 1H, $J_{4,3}$ = 3.5 Hz, $J_{4,5}$ = 1.8 Hz, H-4-furyl); 7.07 (d, 1H, $J_{5,6}$ = 3.6 Hz, H-5); 7.48 (dd, 1H, $J_{3,4}$ = 3.5 Hz, $J_{3,5}$ = 0.8 Hz, H-3-furyl); 7.53–7.58 (m, 2H, H-6,7-naphthyl); 7.66 (dd, 1H, $J_{3,4}$ = 8.2 Hz, $J_{3,2}$ = 7.2 Hz, H-3-naphthyl); 7.90 (d, 1H, $J_{6,5}$ = 3.6 Hz, H-6); 8.03 (m, 1H, CH-5-naphthyl), 8.05 (dm, 1H, $J_{4,3}$ = 8.3 Hz, H-4-naphthyl); 8.08 (dd, 1H, $J_{5,4}$ = 1.8 Hz, $J_{5,3}$ = 0.8 Hz, H-5-furyl); 8.11 (dd, 1H, $J_{2,3}$ = 7.2 Hz, $J_{2,4}$ = 1.4 Hz, H-2-naphthyl); 8.74 (m, 1H, H-8-naphthyl); ^{13}C NMR (125.7 MHz, DMSO-d_6): 22.3 ((**C**H$_3$)$_2$CH); 46.0 ((CH$_3$)$_2$**C**H); 100.04 (CH-5); 110.7 (C-4a); 112.7 (CH-4-furyl); 113.1 (CH-3-furyl); 125.3 (CH-3-naphthyl); 125.8 (CH-6/7-naphthyl); 126.1 (CH-8-naphthyl); 126.5 (CH-6/7-naphthyl); 128.3 (CH-6); 128.4 (CH-5-naphthyl); 128.9 (CH-2-naphthyl); 129.4 (CH-4-naphthyl); 130.9 (C-8a-naphthyl); 133.7 (C-4a-naphthyl); 136.8 (C-1-naphthyl); 146.2 (CH-5-furyl); 146.3 (C-4); 151.6 (C-7a); 152.9 (C-2-furyl); 158.2 (C-2). HR-ESI-MS: *found*: 354.1602 ([M + H]$^+$, calcd for $C_{23}H_{20}ON_3^+$: 354.1601); HR-ESI-MS: *found*: 376.1420 ([M + Na]$^+$, calcd for $C_{23}H_{19}ON_3Na^+$: 376.1420).

2-(4-(Furan-2-yl)-2-(naphth-1-yl)-7*H*-pyrrolo[2,3-*d*]pyrimidin-7-yl)ethan-1-ol (11ac)

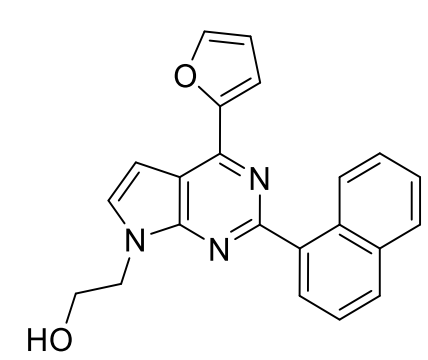

GP B starting from compound **10a** (45.5 mg, 0.17 mmol) and naphthalene-1-boronic acid (89.0 mg, 0.52 mmol). The reaction was performed in microwave reactor at 100 °C for 1 h. HPFC (SiO_2, cHex/EtOAc 8:2 → 5:5) and lyophilization from H_2O/*t*BuOH gave **11ac** (58.0 mg, 95 %) as a sand color solid. ^{1}H NMR (500 MHz, DMSO-d_6): 3.83 (q, 2H, $J_{CH2,CH2}$ = $J_{CH2,OH}$ = 5.6 Hz, NCH$_2$C**H$_2$**OH); 4.39 (t, 2H, $J_{CH2,CH2}$ = 5.7 Hz, NC**H$_2$**CH$_2$OH); 4.99 (t, 1H, $J_{OH,CH2}$ = 5.4 Hz, NCH$_2$CH$_2$O**H**); 6.80 (dd, 1H, $J_{4,3}$ = 3.5 Hz, $J_{4,5}$ = 1.7 Hz, H-4-furyl); 7.05 (d, 1H, $J_{5,6}$ = 3.5 Hz, H-5); 7.48 (dd, 1H, $J_{3,4}$ = 3.5 Hz, $J_{3,5}$ = 0.8 Hz, H-3-furyl); 7.53 – 7.59 (m, 2H, H-6,7-naphthyl); 7.66 (dd, 1H, $J_{3,4}$ = 8.2 Hz, $J_{3,2}$ = 7.2 Hz, H-3-naphthyl); 7.77 (d, 1H, $J_{6,5}$ = 3.5 Hz, H-6); 8.03 (m, 1H, CH-5-naphthyl); 8.06 (dm, 1H, $J_{4,3}$ = 8.2 Hz, H-4-naphthyl); 8.09 (dd, 1H, $J_{5,4}$ = 1.7 Hz, $J_{5,3}$ = 0.8 Hz, H-5-furyl); 8.10 (dd, 1H, $J_{2,3}$ = 7.2 Hz, $J_{2,4}$ = 1.3 Hz, H-2-naphthyl); 8.74 (m, 1H, H-8-naphthyl); ^{13}C NMR (125.7 MHz, DMSO-d_6): 46.8 (N**C**H$_2$CH$_2$OH); 59.9 (NCH$_2$**C**HOH); 99.5 (CH-5); 110.5 (C-4a); 112.6 (CH-4-furyl); 113.0 (CH-3-furyl); 125.3 (CH-3-naphthyl); 125.8 (CH-6/7-naphthyl); 126.1 (CH-8-naphthyl); 126.5 (CH-6/7-naphthyl); 128.3 (CH-5-naphthyl);

128.8 (CH-2-naphthyl); 129.3 (CH-4-naphthyl); 130.9 (C-8a-naphthyl); 132.0 (CH-6); 133.6 (C-4a-naphthyl); 136.8 (C-1-naphthyl); 146.16 (CH-5-furyl); 146.17 (C-4); 152.2 (C-7a); 152.9 (C-2-furyl); 158.3 (C-2). HR-ESI-MS: *found*: 356.1393 ($[M + H]^+$, calcd for $C_{22}H_{18}O_2N_3^+$: 356.1394); HR-ESI-MS: *found*: 378.1211 ($[M + Na]^+$, calcd for $C_{22}H_{17}O_2N_3Na^+$: 378.1213).

S2. Adenosine Receptor Activity Screening

All the target nucleosides were tested for their agonistic and antagonistic activities on all four adenosine receptor subtypes (A_1AR, $A_{2A}AR$, $A_{2B}AR$, and A_3AR) using an aequorin functional assay.[2] In this assay, coelenterazine-loaded cells expressing the adenosine receptors were dispensed, and the agonistic activity of compounds was analyzed using a FLIPR Penta multimodal reader (Molecular Devices). After the initial measurement, the cells were incubated within the instrument for an additional 15 minutes at room temperature. The antagonistic response, indicated by a reduction in luminiscence, was then measured immediately after adding a reference agonist at 80% of its maximal effective concentration (EC_{80}). Raw data, expressed as the area under the curve (AUC), were normalized as a percentage of the response to the reference agonist. The normalized responses were plotted against the concentrations of the test compounds, and EC_{50} values (for agonists) and IC_{50} (for antagonists) were determined. The data used for analysis were obtained from three independent experiments.

S3. Annexin V binding assay

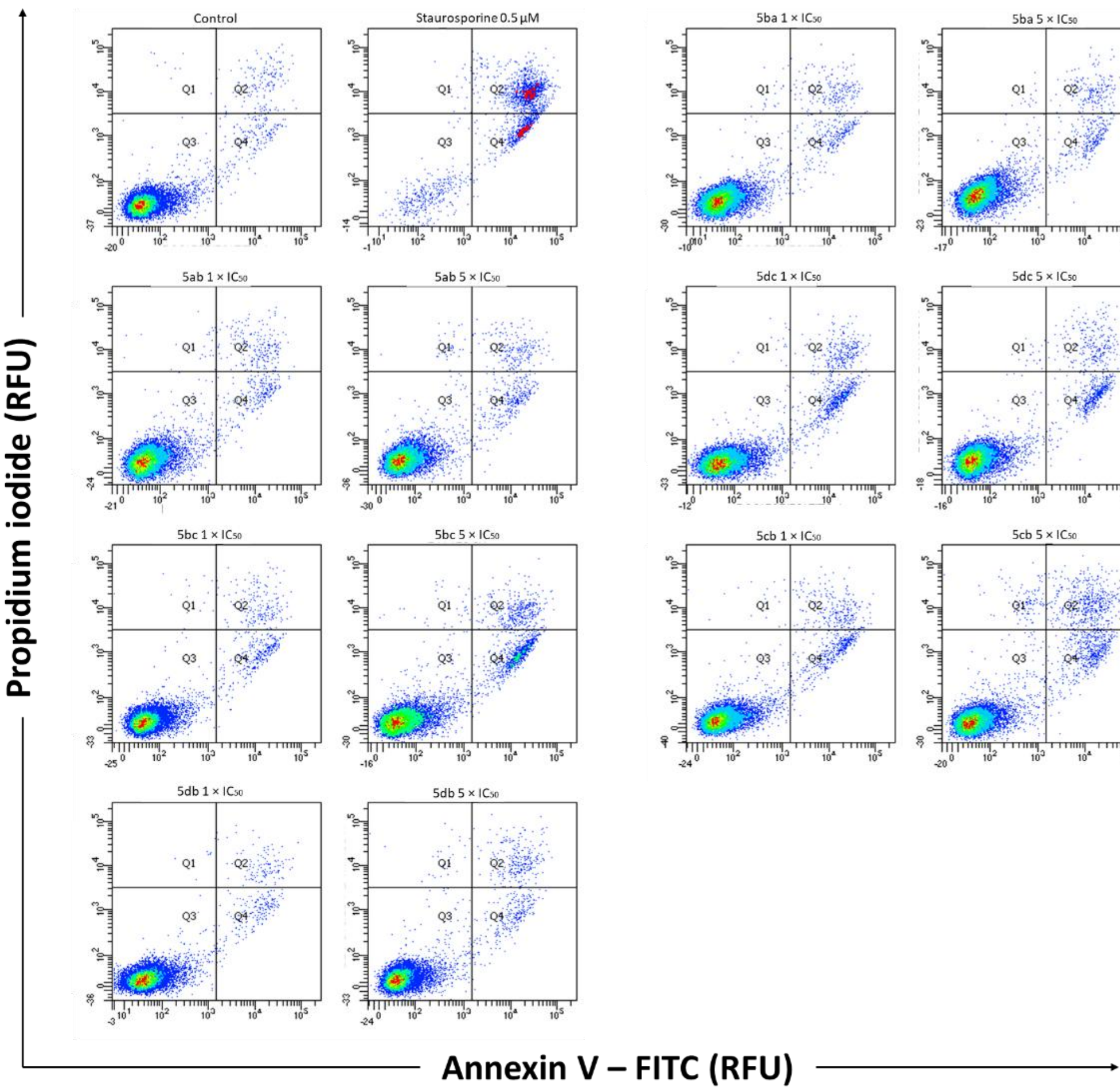

Supplementary Figure 1: Flow cytometric analysis of apoptosis in CCRF-CEM cells treated with tested compounds. Cells were incubated with the tested compounds at 1 × IC_{50} and 5 × IC_{50} concentrations for 24 h and subsequently stained with annexin V-FITC and propidium iodide (PI). Dot plots represent the distribution of viable (Annexin V− /PI−), early apoptotic (Annexin V+/PI−), and late apoptotic or necrotic (Annexin V+/PI+) cells. Representative results from one of three independent experiments are shown.

Expression of adenosine receptors levels

Pellets of individual cell lines (5 × 10^6 cells) were resuspended in TRI Reagent (Sigma-Aldrich). Subsequently, mRNA was isolated using the miRNeasy Mini Kit (QIAGEN) according to the manufacturer's protocol. Reverse transcription was performed using 3 μg of total RNA in a total reaction volume of 30 μL, with random hexamers priming the cDNA synthesis.

The resulting cDNA was used to determine the relative expression of individual adenosine receptors via qPCR. Amplification was carried out using the GoTaq® qPCR Master Mix (Promega) on a LightCycler® 480 Real-Time PCR System (Roche) using the thermal profile outlined in Table 2.

No expression of A_1 and A_{2B} receptors was detected in the tested cell lines (with C_T values remaining undetermined after 40 cycles). The relative expression levels of A_{2A} and A3 receptors for the individual cell lines are presented in Figure 2. Target gene expression levels were normalized to the housekeeping gene beta-actin (β-actin).

Supplementary Table 1: Primers used for PCR experiments

Gene	Primer	Sequence	Product length (bp)
hADORA1	**FP**	5'-TCCCTCTCCGGTACAAGATG-3'	300
hADORA1	**RP**	5'-GCTGCTTGCGGATTAGGTAG-3'	300
hADORA2A	**FP**	5'-CTACCGTATCCGCGAGTTCC-3'	397
hADORA2A	**RP**	5'-GGAAGGGGCAAACTCCATGA-3'	397
hADORA2B	**FP**	5'-GTGTCCCGCTCAGGTATAAAAG-3'	414
hADORA2B	**RP**	5'-ACAGGGCAAAAATCCCCACA-3'	414
hADORA3	**FP**	5'-TGTTTGGCTGGAACATGAAA-3'	155
hADORA3	**RP**	5'-ATAGATGGCGCACATGACAA-3'	155
hACTB	**FP**	5'-GGACTTCGAGCAAGAGATGG-3'	234
hACTB	**RP**	5'-AGCACTGTGTTGGCGTACAG-3'	234

Supplementary Table 2: Thermal profile of qPCR

Step	Process	Temperature (°C)	Time (s)	Cycles
**1**	Initial Denaturation / Activation	95	120	1
**2**	Denaturation	95	15	40
**3**	Annealing / Extension	60	60	
**4**	Melting Curve Analysis	95	Continuous	1
**5**	Cooling	37	60	1

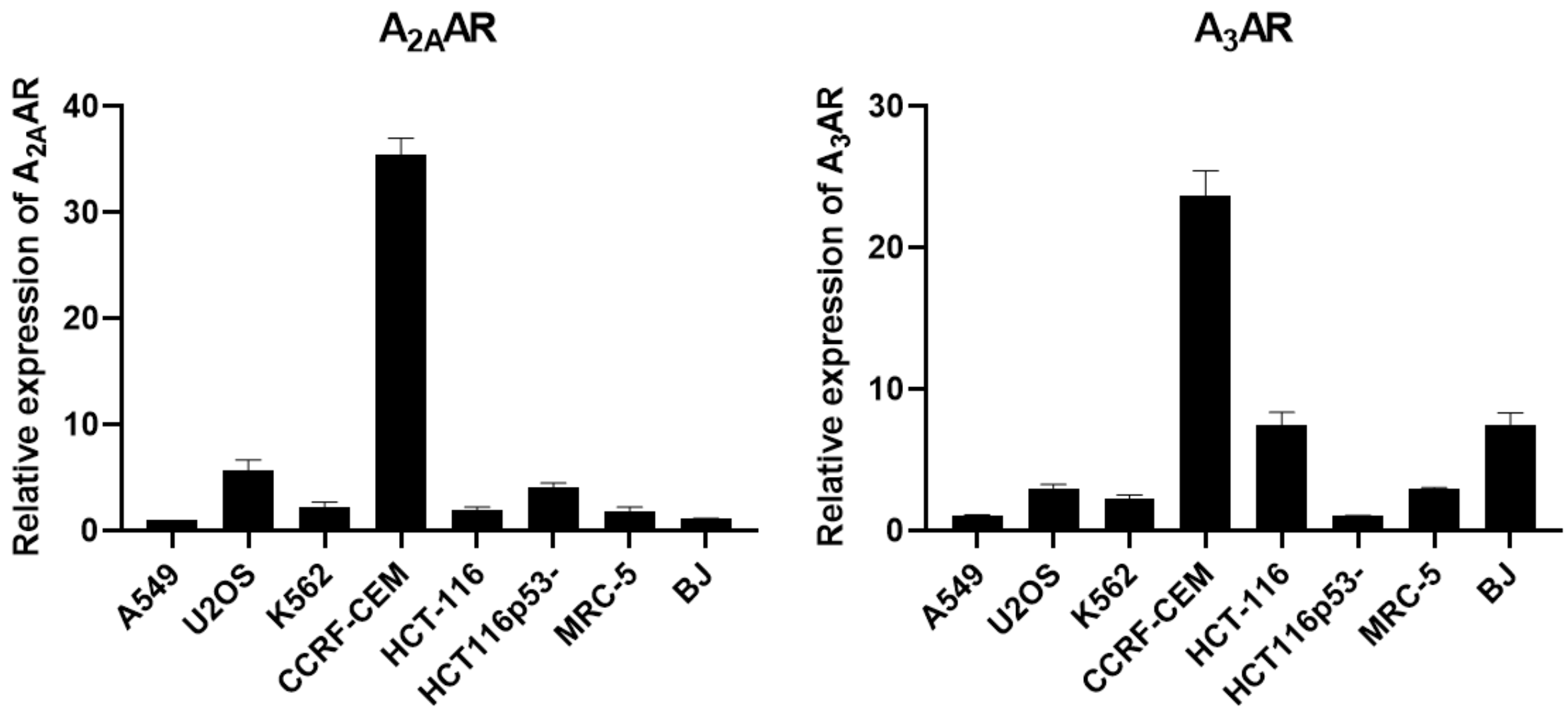

Supplementary Figure 2: Relative expression of A_{2A} and A_3 adenosine receptors.

Supplementary Table 3: Relative expression of A_{2A} and A_3 adenosine receptors.

**Cell line**	**A_{2A}AR**		**A_3AR**	
	**Mean**	**SD**	**Mean**	**SD**
**A549**	1.01	0.01	1.07	0.07
**U2OS**	5.67	1.01	3.00	0.29
**K562**	2.20	0.52	2.26	0.27
**CCRF-CEM**	35.42	1.59	23.66	1.80
**HCT-116**	1.99	0.24	7.43	0.97
**HCT116p53-**	4.08	0.42	1.05	0.04
**MRC-5**	1.80	0.46	2.99	0.08
**BJ**	1.17	0.00	7.46	0.88

S4. Cytotoxicity Data – IC_{50} Curves

LEM 230901 (5aa)

BJ (IC50 > 50 µM)

Primary screen (50 µM)

plate	well	sample id	PI	pass	conc	inc		
1 (Plate 1)	J3	LEM00230901	9.708		50.0uM	+		
1 (Plate 1)	J4	LEM00230901	20.495		50.0uM	+		
1 (Plate 1)	J5	LEM00230901	14.778		50.0uM	+		

plate	well	sample id	PI	pass	conc	inc		
1 (Plate 1)	J3	LEM00230901	58.522		50.0uM	+		
1 (Plate 1)	J4	LEM00230901	44.746		50.0uM	+		
1 (Plate 1)	J5	LEM00230901	13.072		50.0uM	+		

MRC5 (IC50 > 50 µM)

Primary screen (50 µM)

plate	well	sample id	PI	pass	conc	inc		
1 (Plate 1)	J3	LEM00230901	23.401		50.0uM	+		
1 (Plate 1)	J4	LEM00230901	21.994		50.0uM	+		
1 (Plate 1)	J5	LEM00230901	18.092		50.0uM	+		

plate	well	sample id	PI	pass	conc	inc		
1 (Plate 1)	J3	LEM00230901	8.3679		50.0uM	+		
1 (Plate 1)	J4	LEM00230901	8.3679		50.0uM	+		
1 (Plate 1)	J5	LEM00230901	6.3935		50.0uM	+		

A549 (IC50 > 50 µM)

Primary screen (50 µM)

plate	well	sample id	PI	pass	conc	inc		
1 (Plate 1)	J3	LEM00230901	11.302		50.0uM	+		
1 (Plate 1)	J4	LEM00230901	12.268		50.0uM	+		
1 (Plate 1)	J5	LEM00230901	2.1294		50.0uM	+		

plate	well	sample id	PI	pass	conc	inc		
1 (Plate 1)	J3	LEM00230901	12.604		50.0uM	+		
1 (Plate 1)	J4	LEM00230901	11.559		50.0uM	+		
1 (Plate 1)	J5	LEM00230901	0.8034		50.0uM	+		

CCRF-CEM (IC50 > 50 µM)

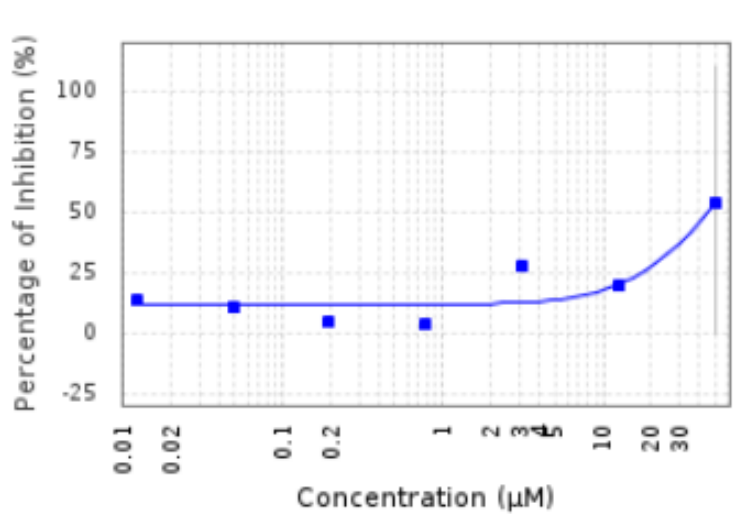

IC50: >50 pass
slope=1.500 min=11.378 max=110.000 err=342.611 R^2=0.8125

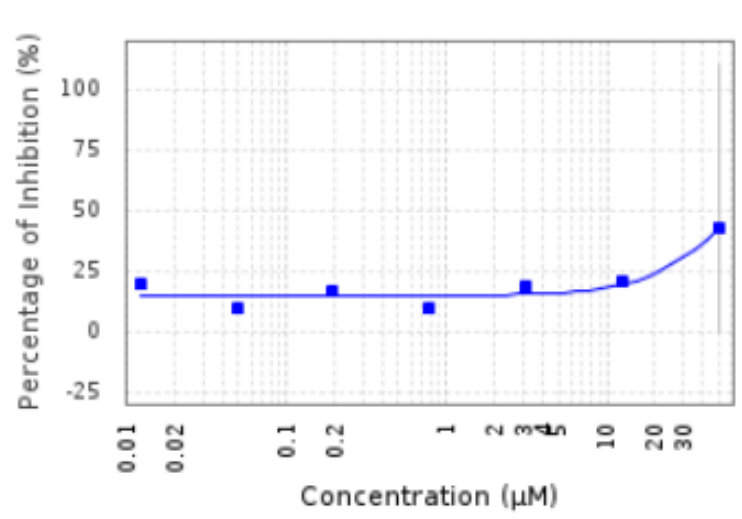

IC50: >50 pass
slope=1.500 min=14.522 max=109.936 err=96.759 R^2=0.8741

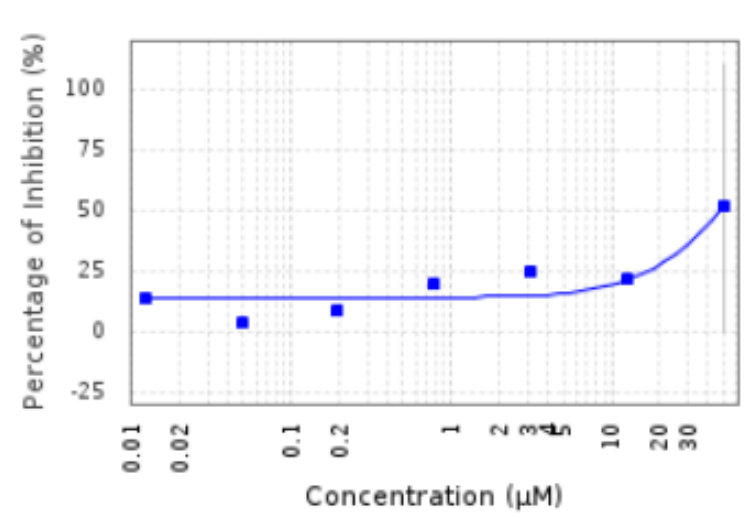

IC50: >50 pass
slope=1.500 min=13.633 max=110.000 err=269.884 R^2=0.8177

HCT116 (IC50 > 50 μM)

Primary screen (50 μM)

plate	well	sample id	PI	pass	conc	inc	✓	⊗
1 (Plate 1)	J3	LEM00230901	-1.0521	●	50.0uM	+	✓	⊗
1 (Plate 1)	J4	LEM00230901	-1.4946	●	50.0uM	+		
1 (Plate 1)	J5	LEM00230901	-0.1671	●	50.0uM	+		

plate	well	sample id	PI	pass	conc	inc	✓	⊗
1 (Plate 1)	J3	LEM00230901	4.6395	●	50.0uM	+	✓	⊗
1 (Plate 1)	J4	LEM00230901	5.0282	●	50.0uM	+		
1 (Plate 1)	J5	LEM00230901	6.083	●	50.0uM	+		

HCT116p53-/- (IC50 > 50 μM)

Primary screen (50 μM)

plate	well	sample id	PI	pass	conc	inc	✓	⊗
1 (Plate 1)	J3	LEM00230901	4.5457	●	50.0uM	+	✓	⊗
1 (Plate 1)	J4	LEM00230901	4.6261	●	50.0uM	+		
1 (Plate 1)	J5	LEM00230901	5.9117	●	50.0uM	+		

plate	well	sample id	PI	pass	conc	inc	✓	⊗
1 (Plate 1)	J3	LEM00230901	11.732	●	50.0uM	+	✓	⊗
1 (Plate 1)	J4	LEM00230901	14.272	●	50.0uM	+		
1 (Plate 1)	J5	LEM00230901	12.29	●	50.0uM	+		

K562 (IC50 > 50 μM)

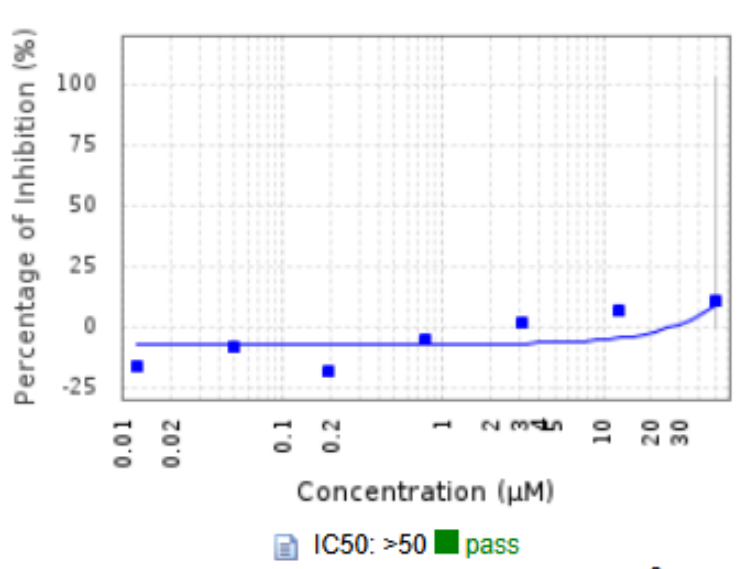

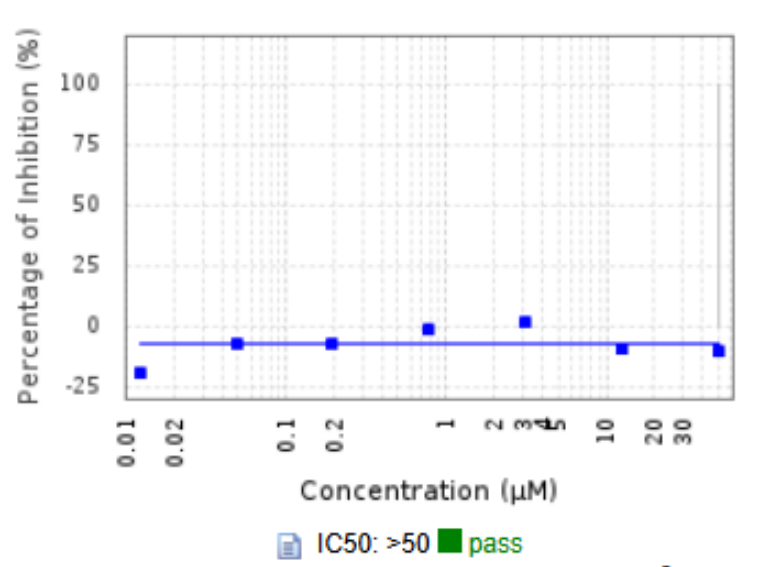

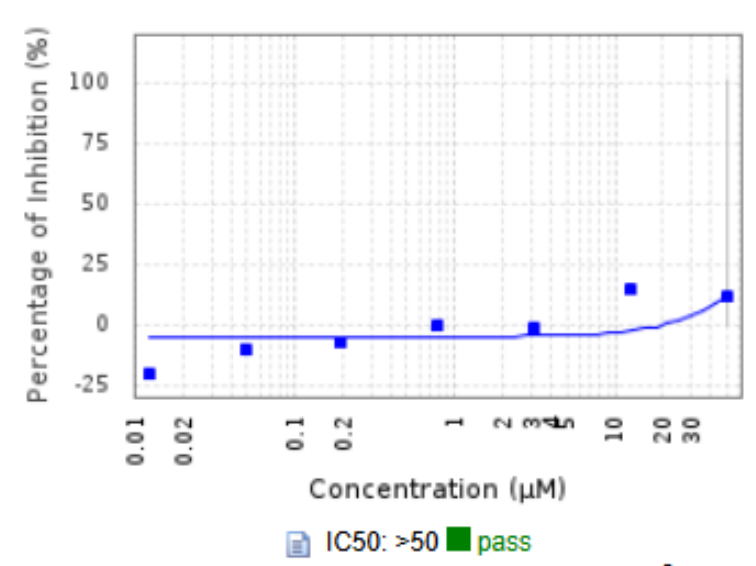

U2OS (IC50 > 50 μM)

Primary screen (50 μM)

plate	well	sample id	PI	pass	conc	inc	✓	⊗
1 (Plate 1)	J3	LEM00230901	22.851	●	50.0uM	+	✓	⊗
1 (Plate 1)	J4	LEM00230901	25.111	●	50.0uM	+		
1 (Plate 1)	J5	LEM00230901	23.72	●	50.0uM	+		

plate	well	sample id	PI	pass	conc	inc	✓	⊗
1 (Plate 1)	J3	LEM00230901	26.422	●	50.0uM	+	✓	⊗
1 (Plate 1)	J4	LEM00230901	13.848	●	50.0uM	+		
1 (Plate 1)	J5	LEM00230901	15.944	●	50.0uM	+		

LEM 230900 (5ab)

BJ (IC50 10 µM)

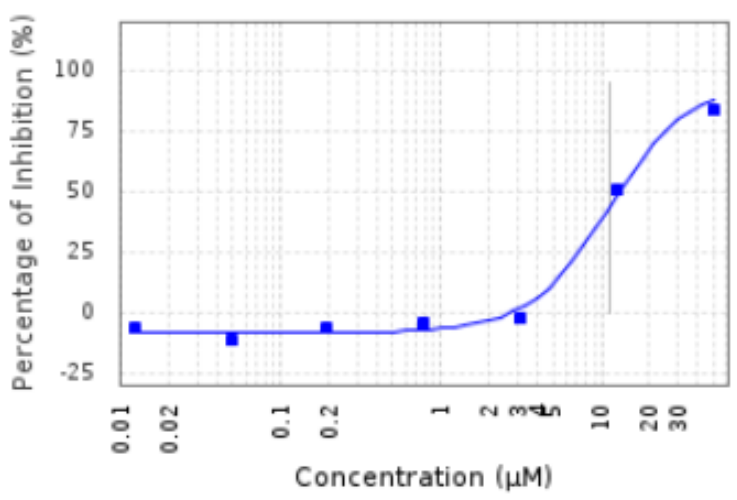

IC50: 11.16 *(9.8638 - 12.627)* pass
slope=1.740 min=-8.491 max=95.000 err=69.786 R^2=0.9916

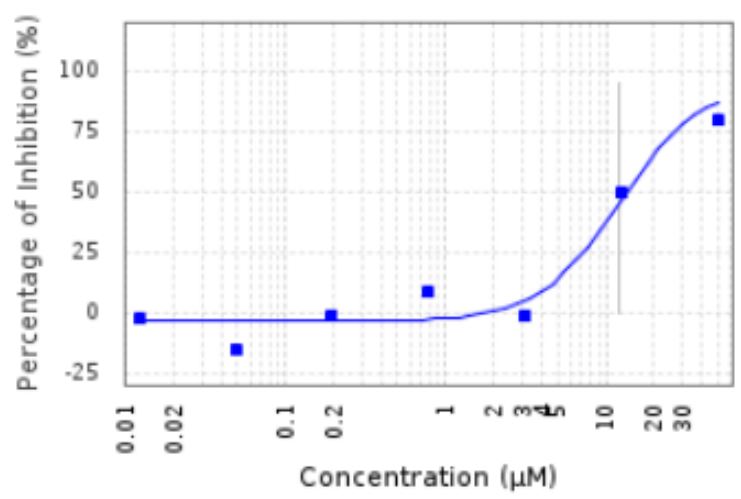

IC50: 12.279 *(9.3252 - 16.169)* pass
slope=1.700 min=-3.986 max=95.000 err=358.086 R^2=0.9499

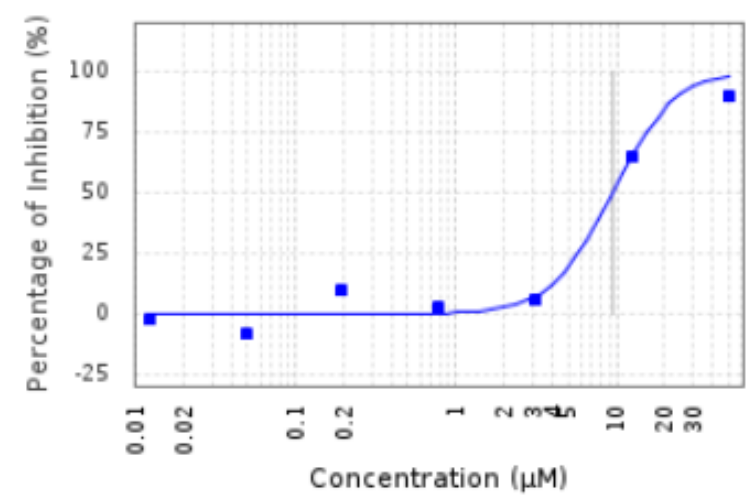

IC50: 9.5068 *(7.262 - 12.445)* pass
slope=2.260 min=-0.468 max=100.000 err=239.564 R^2=0.9726

MRC5 (IC50 12 µM)

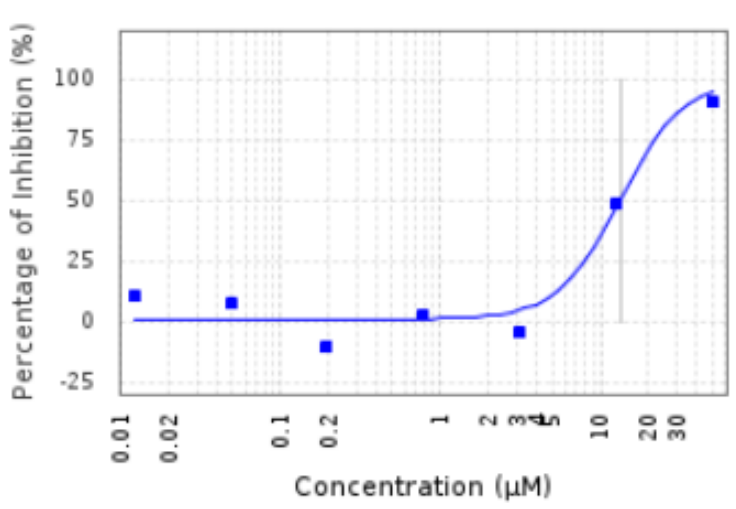

IC50: 13.402 *(10.004 - 17.953)* pass
slope=2.200 min=0.675 max=100.000 err=358.205 R^2=0.9545

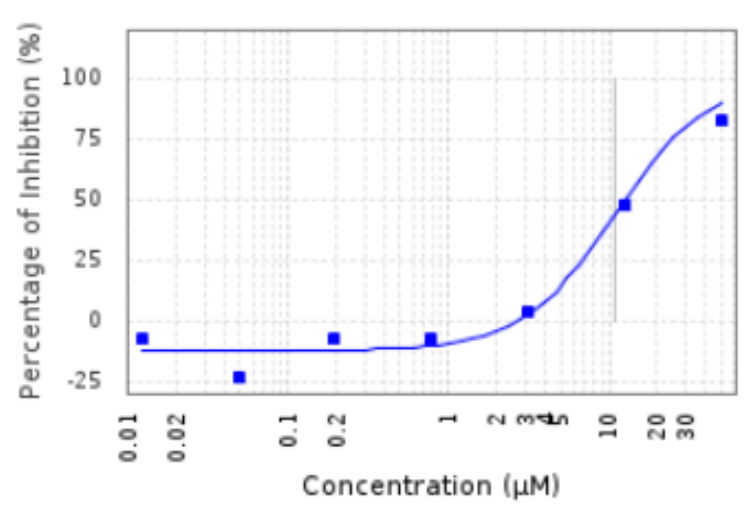

IC50: 10.912 *(7.0886 - 16.799)* pass
slope=1.500 min=-12.610 max=100.000 err=224.203 R^2=0.9742

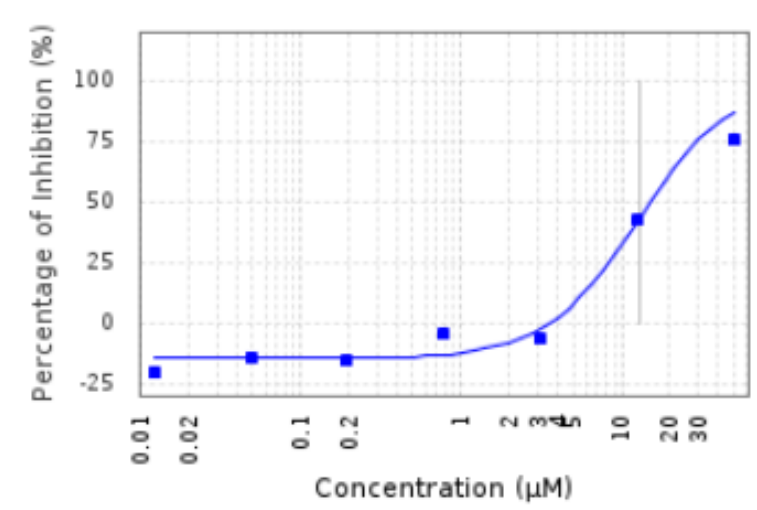

IC50: 12.745 *(7.5355 - 21.556)* pass
slope=1.500 min=-15.000 max=100.000 err=246.588 R^2=0.9690

A549 (IC50 > 50 µM)

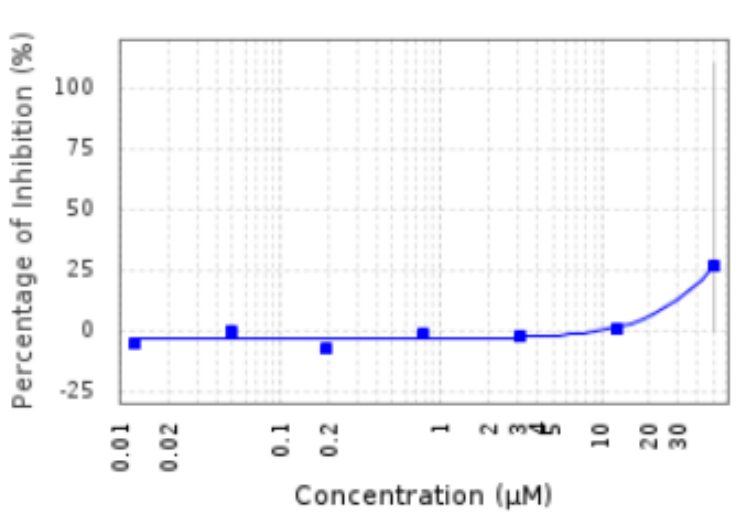

IC50: >50 pass
slope=1.502 min=-3.605 max=110.000 err=35.323 R^2=0.9540

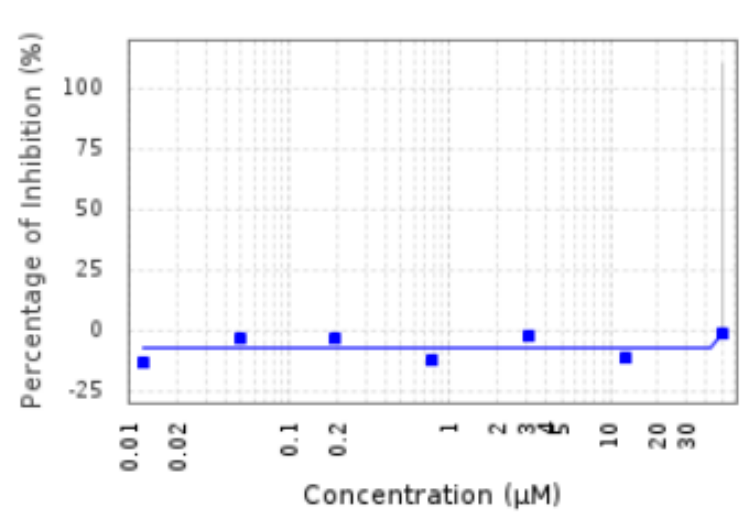

IC50: >50 pass
slope=13.389 min=-7.754 max=110.000 err=130.824 R^2=0.2110

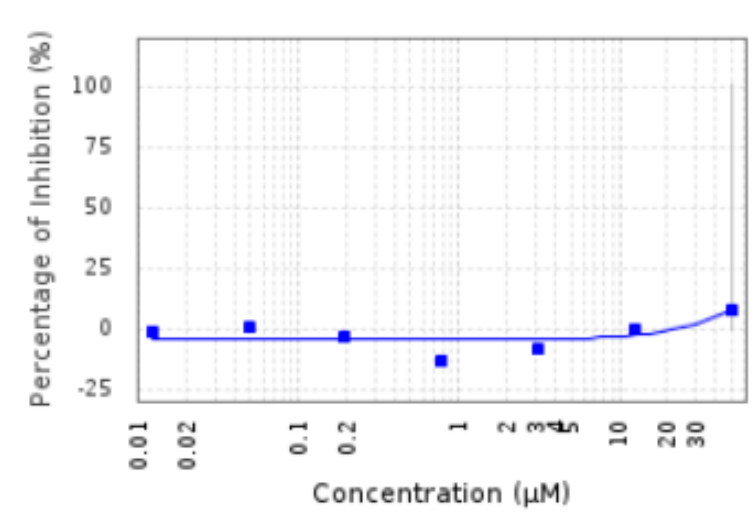

IC50: >50 pass
slope=1.500 min=-4.731 max=100.054 err=135.652 R^2=0.4920

CCRF-CEM (IC50 5,3 µM)

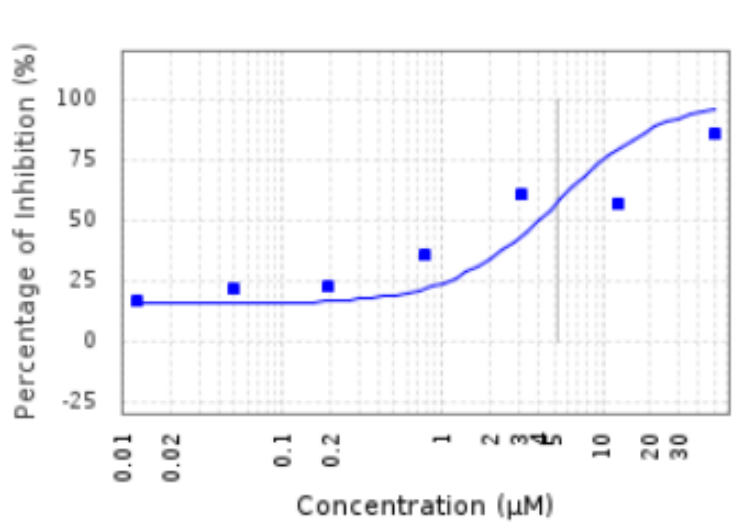

IC50: 5.2796 *(1.8812 - 14.817)* pass
slope=1.300 min=15.000 max=100.000 err=1141.064 R^2=0.7109

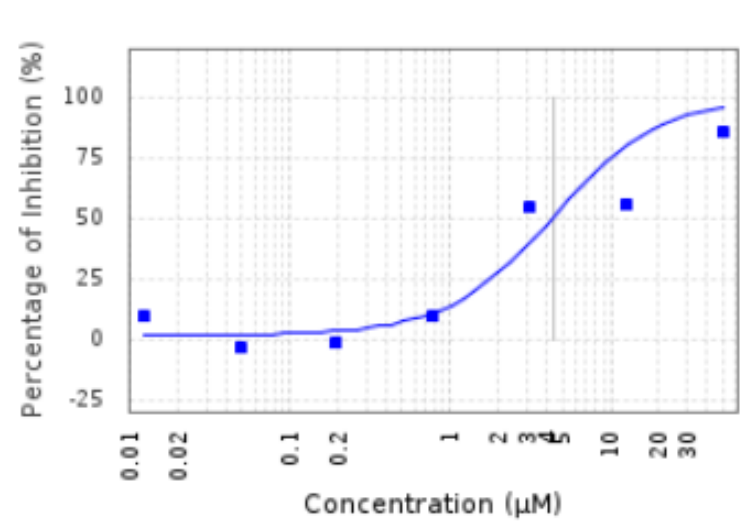

IC50: 4.4453 *(1.9788 - 9.9863)* pass
slope=1.300 min=1.466 max=100.000 err=1002.079 R^2=0.8617

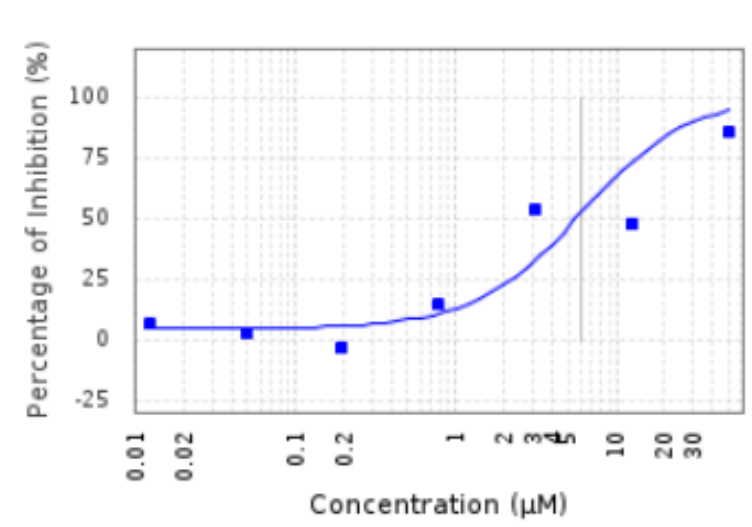

IC50: 6.0649 *(2.2683 - 16.216)* pass
slope=1.300 min=4.336 max=100.000 err=1247.492 R^2=0.8114

HCT116 (IC50 35 μM)

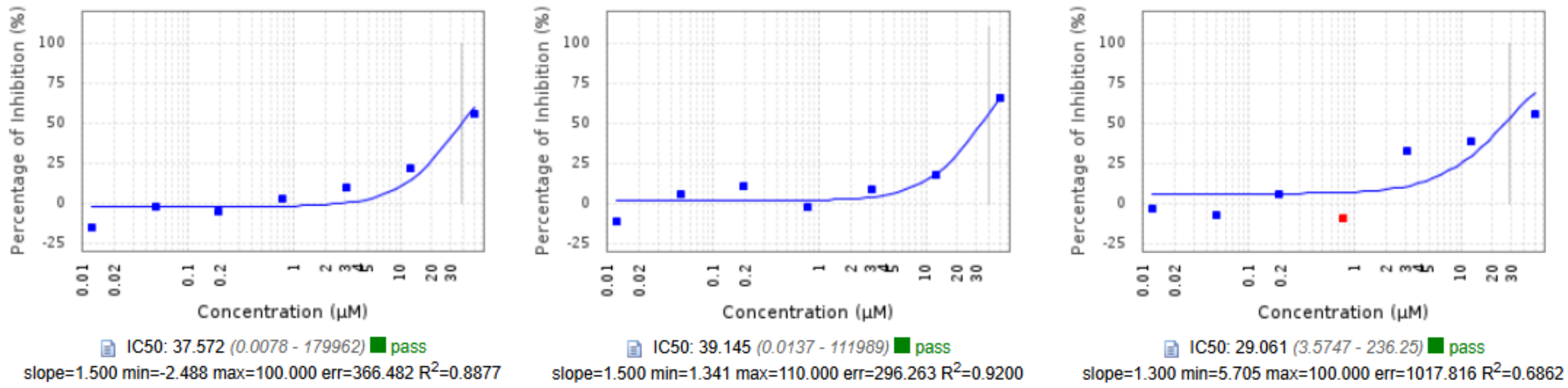

HCT116p53-/- (IC50 > 50 μM)

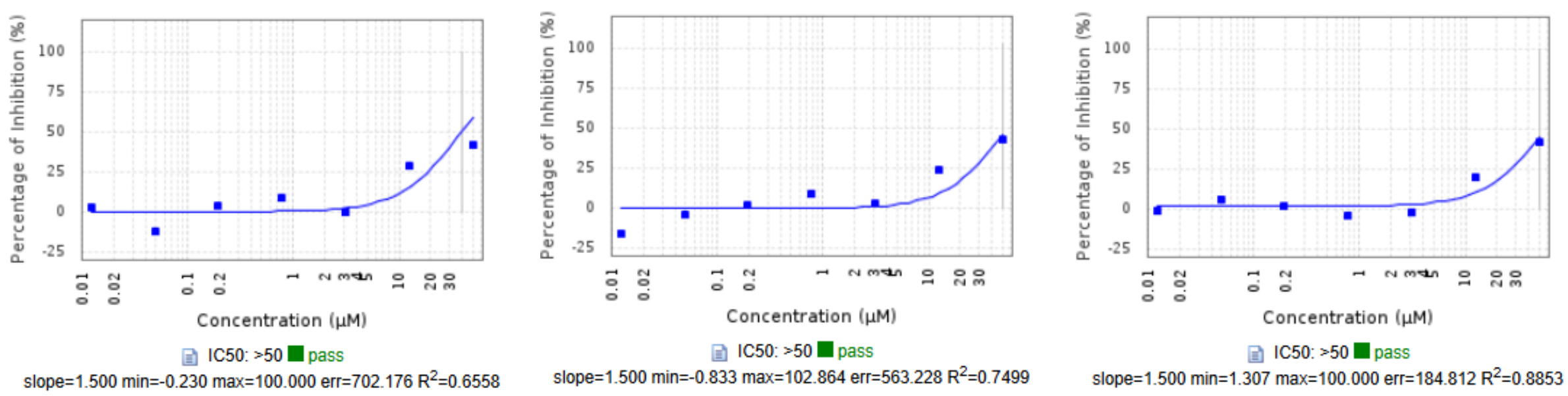

K562 (IC50 > 50 μM)

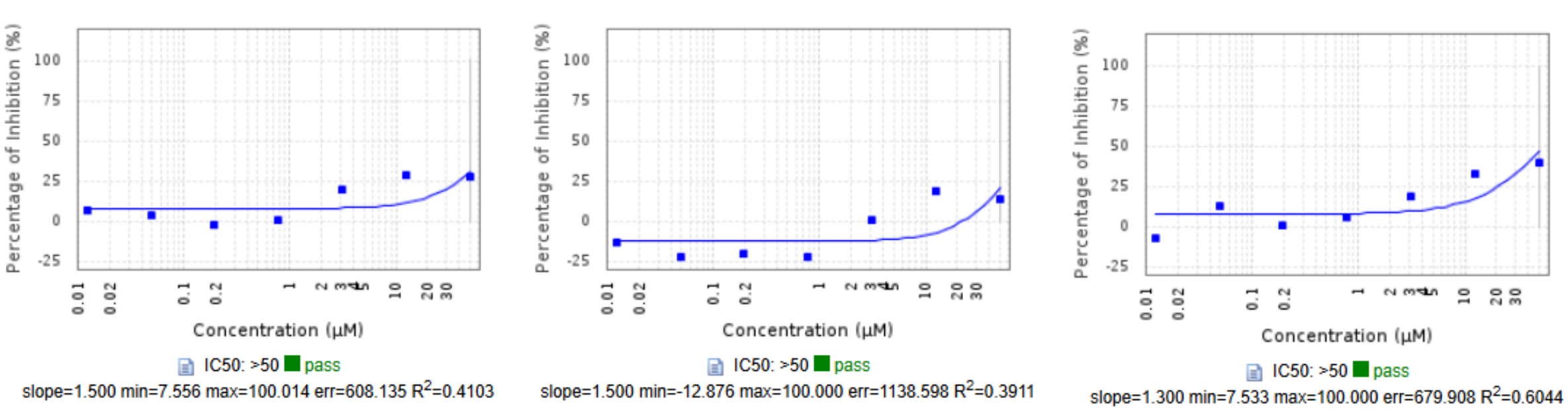

U2OS (IC50 33 µM)

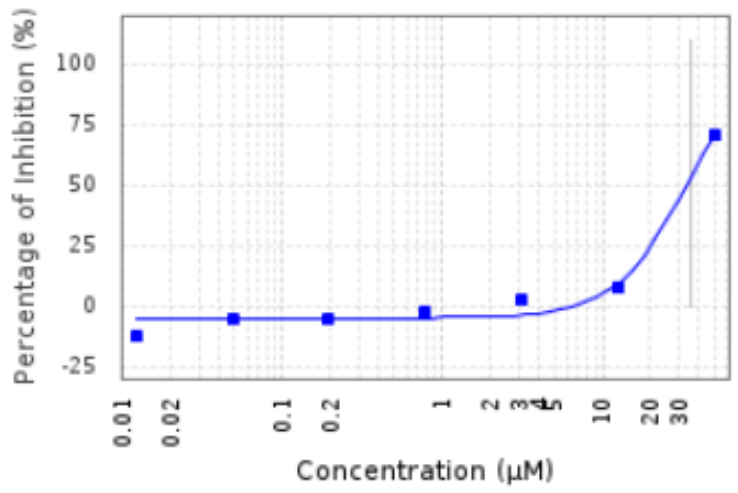

IC50: 35.289 (0.1549 - 8041) pass
slope=1.878 min=-5.119 max=110.000 err=102.905 R^2=0.9789

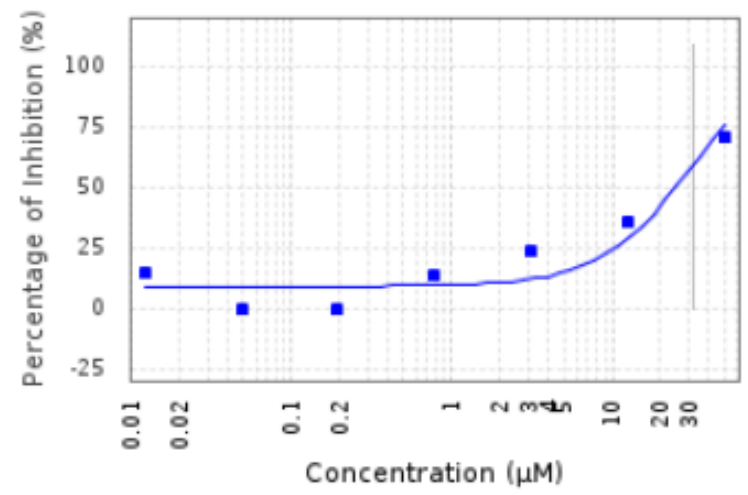

IC50: 31.705 (0.0639 - 15735) pass
slope=1.500 min=8.844 max=108.489 err=432.184 R^2=0.8848

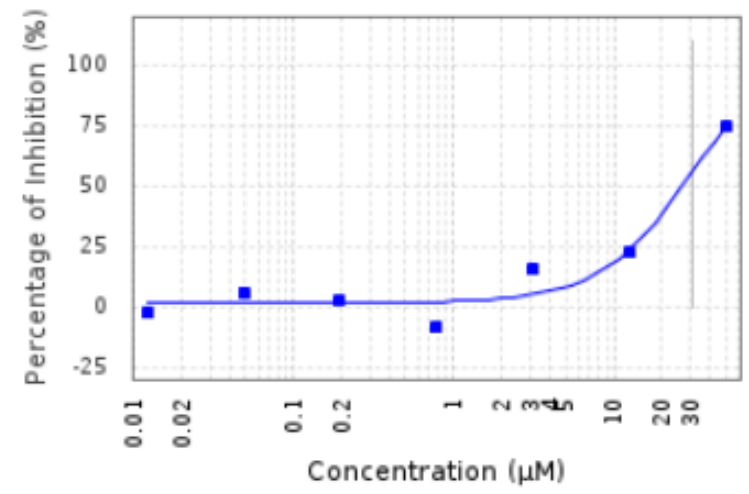

IC50: 31.195 (0.4730 - 2057.2) pass
slope=1.500 min=1.419 max=109.965 err=253.119 R^2=0.9452

LEM 230902 (5ac)

BJ (IC50 > 50 µM)

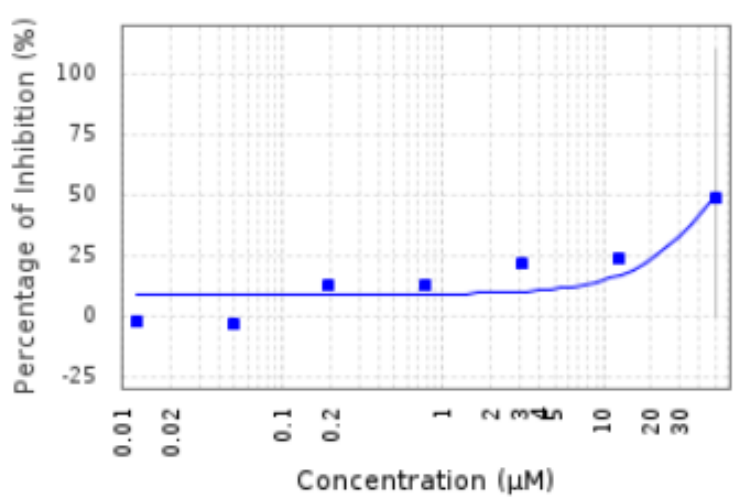

IC50: >50 pass
slope=1.500 min=8.621 max=109.536 err=483.816 R^2=0.7411

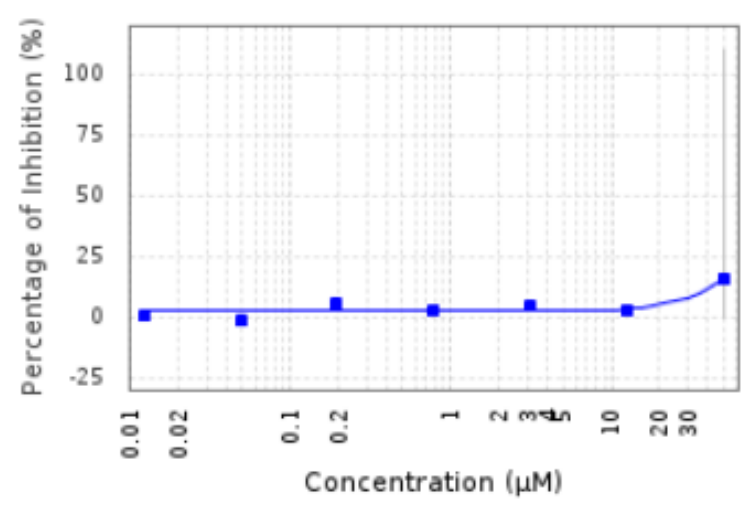

IC50: >50 pass
slope=1.868 min=2.016 max=110.000 err=34.690 R^2=0.8181

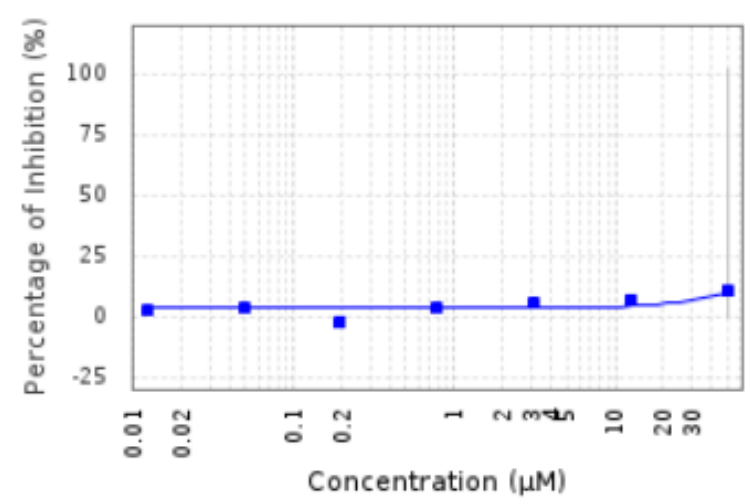

IC50: >50 pass
slope=1.500 min=3.051 max=101.729 err=40.252 R^2=0.5892

MRC5 (IC50 > 50 µM)

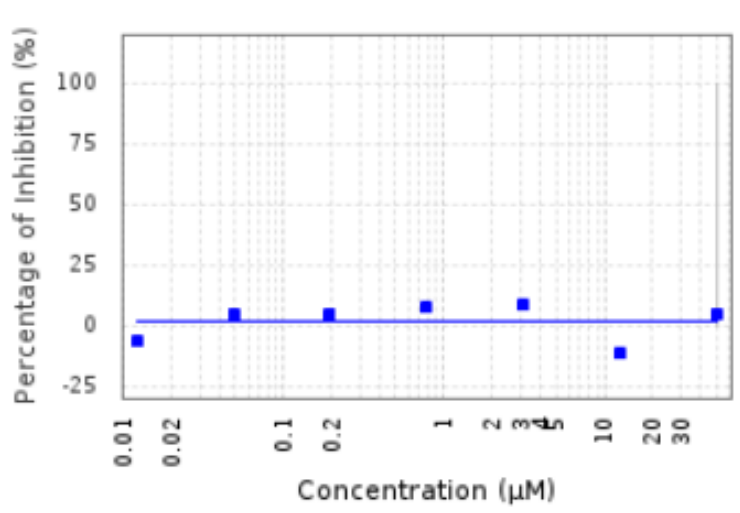

IC50: >50 pass
slope=6.187 min=1.665 max=100.000 err=351.355 R^2=0.0000

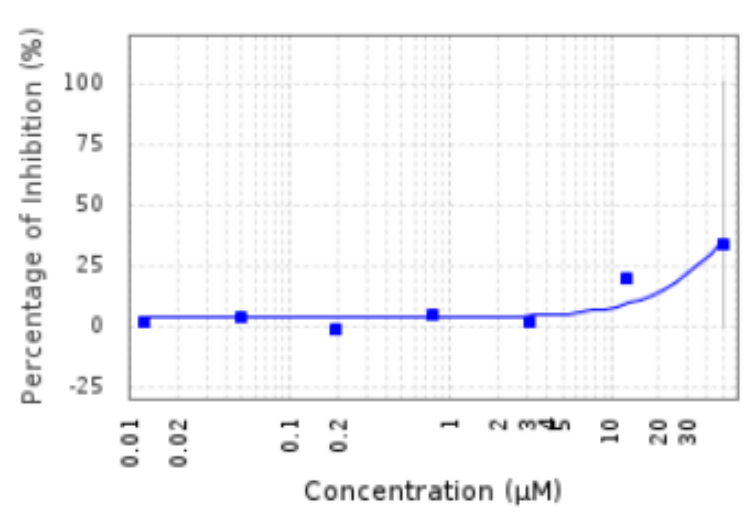

IC50: >50 pass
slope=1.500 min=3.279 max=100.498 err=154.079 R^2=0.8478

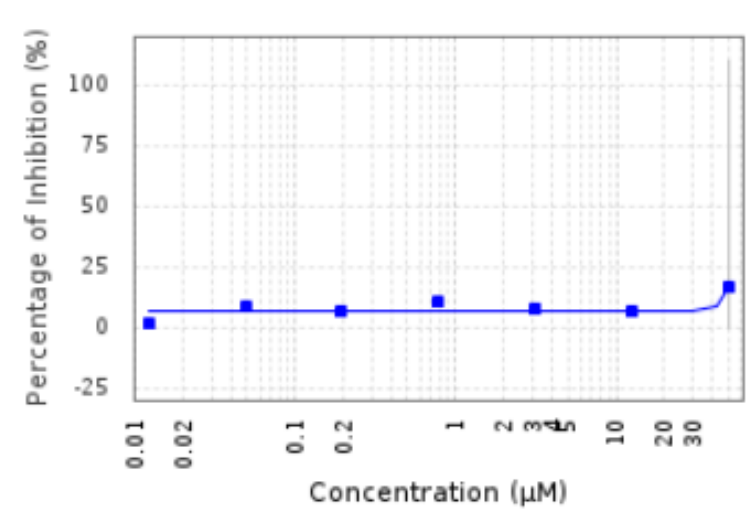

IC50: >50 pass
slope=9.836 min=6.741 max=110.000 err=41.366 R^2=0.6585

A549 (IC50 > 50 µM)

Primary screen (50 µM)

plate	well	sample id	PI	pass	conc	inc	✓	⊗
1 (Plate 1)	K3	LEM00230902	10.759		50.0uM	+	✓	⊗
1 (Plate 1)	K4	LEM00230902	8.6469		50.0uM	+		
1 (Plate 1)	K5	LEM00230902	3.6381		50.0uM	+		

plate	well	sample id	PI	pass	conc	inc	✓	⊗
1 (Plate 1)	K3	LEM00230902	11.805		50.0uM	+	✓	⊗
1 (Plate 1)	K4	LEM00230902	9.1623		50.0uM	+		
1 (Plate 1)	K5	LEM00230902	11.437		50.0uM	+		

CCRF-CEM (IC50 > 50 µM)

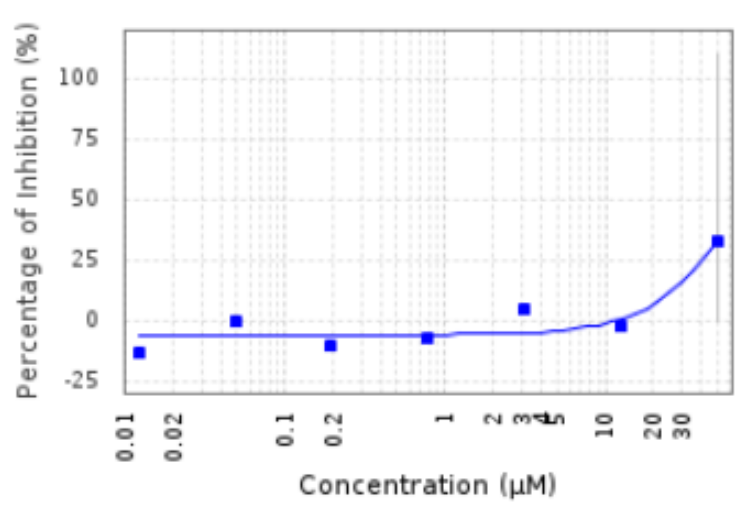

IC50: >50 pass

slope=1.532 min=-6.166 max=110.000 err=222.115 R^2=0.8484

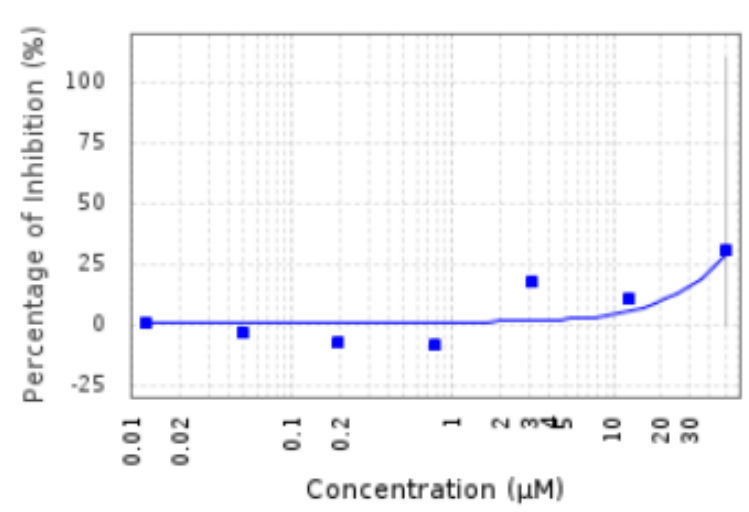

IC50: >50 pass

slope=1.500 min=0.729 max=110.000 err=467.145 R^2=0.6288

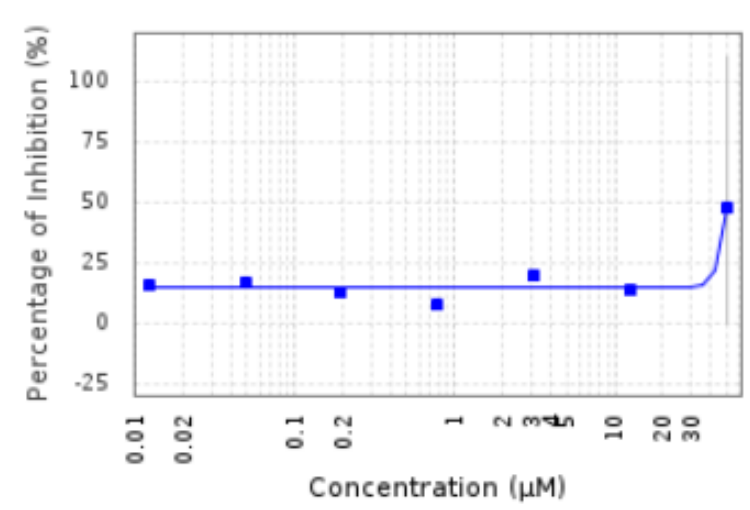

IC50: >50 pass

slope=10.600 min=14.287 max=110.000 err=90.609 R^2=0.9116

HCT116 (IC50 > 50 µM)

Primary screen (50 µM)

plate	well	sample id	PI	pass	conc	inc	✓	⊗
1 (Plate 1)	K3	LEM00230902	40.037		50.0uM	+	✓	⊗
1 (Plate 1)	K4	LEM00230902	20.82		50.0uM	+		
1 (Plate 1)	K5	LEM00230902	16.521		50.0uM	+		

plate	well	sample id	PI	pass	conc	inc	✓	⊗
1 (Plate 1)	K4	LEM00230902	7.915		50.0uM	+		
1 (Plate 1)	K5	LEM00230902	7.6929		50.0uM	+		
1 (Plate 1)	L3	LEM00230903	14.41		50.0uM	+	✓	⊗

HCT116p53-/- (IC50 > 50 µM)

Primary screen (50 µM)

plate	well	sample id	PI	pass	conc	inc	✓	⊗
1 (Plate 1)	K3	LEM00230902	24.473		50.0uM	+	✓	⊗
1 (Plate 1)	K4	LEM00230902	24.152		50.0uM	+		
1 (Plate 1)	K5	LEM00230902	17.402		50.0uM	+		

plate	well	sample id	PI	pass	conc	inc	✓	⊗
1 (Plate 1)	K3	LEM00230902	20.282		50.0uM	+	✓	⊗
1 (Plate 1)	K4	LEM00230902	10.245		50.0uM	+		
1 (Plate 1)	K5	LEM00230902	14.768		50.0uM	+		

K562 (IC50 > 50 µM)

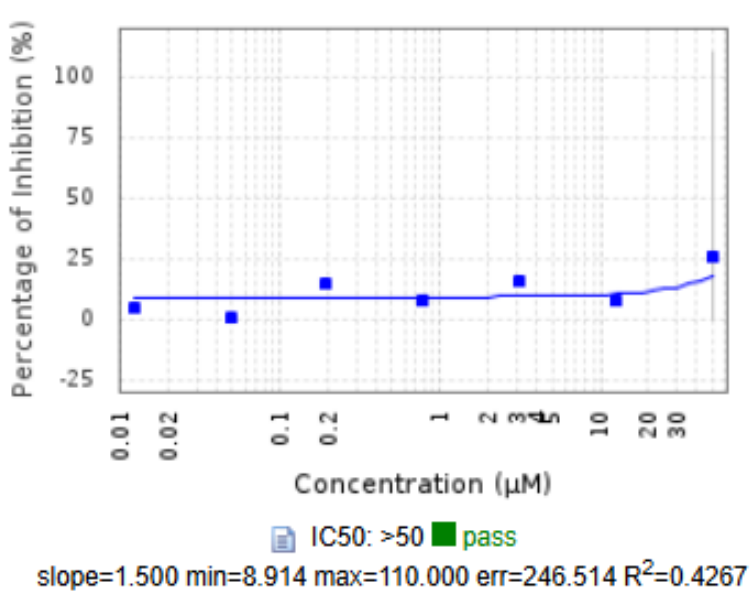

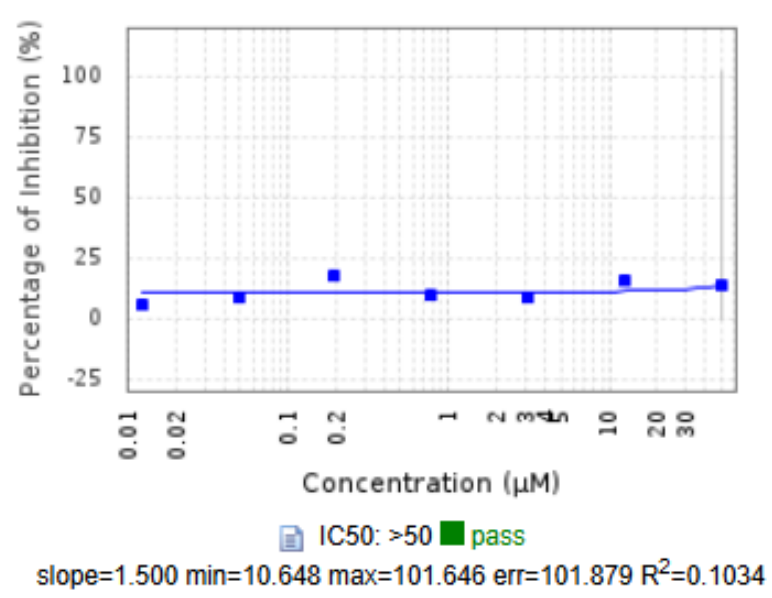

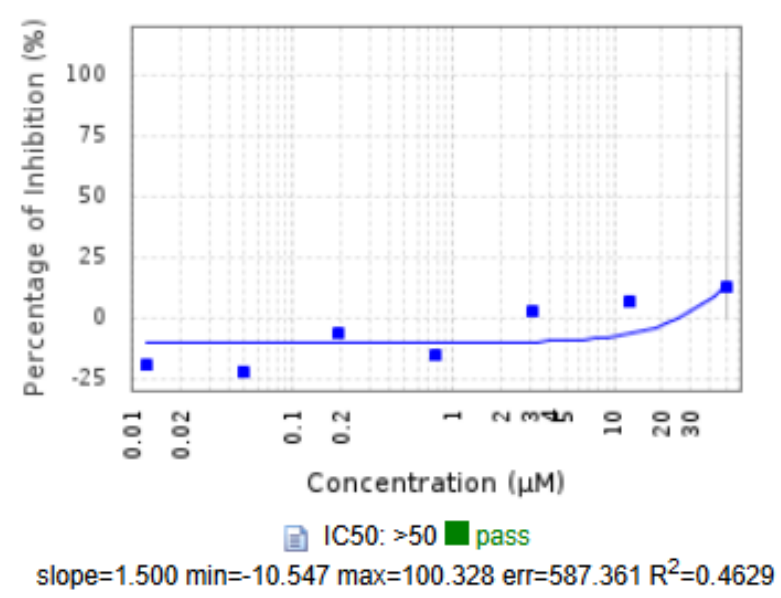

U2OS (IC50 > 50 µM)

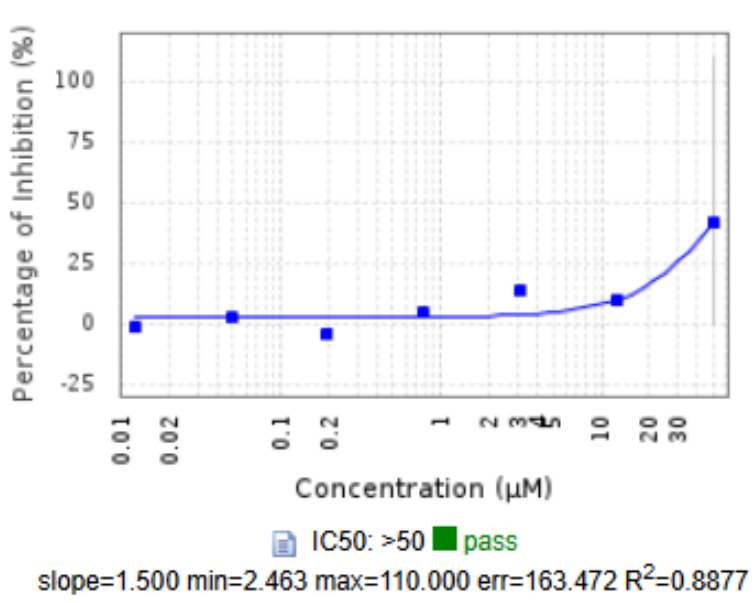

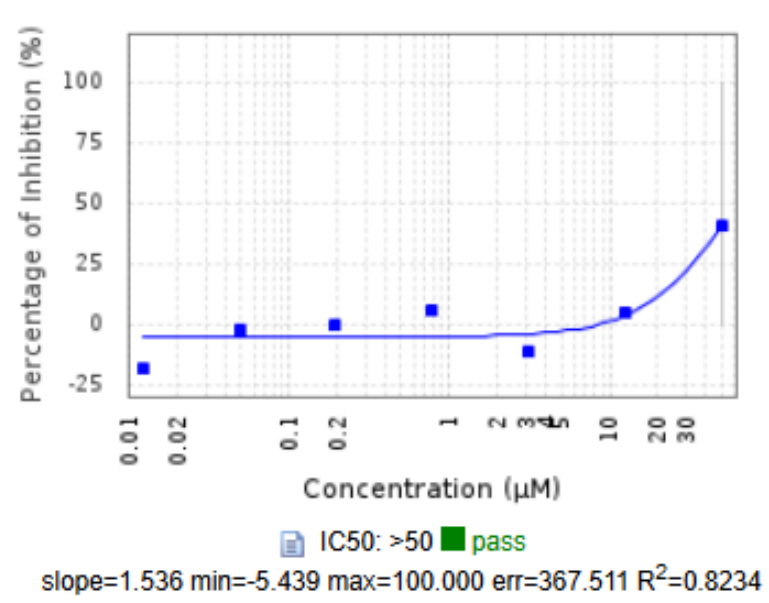

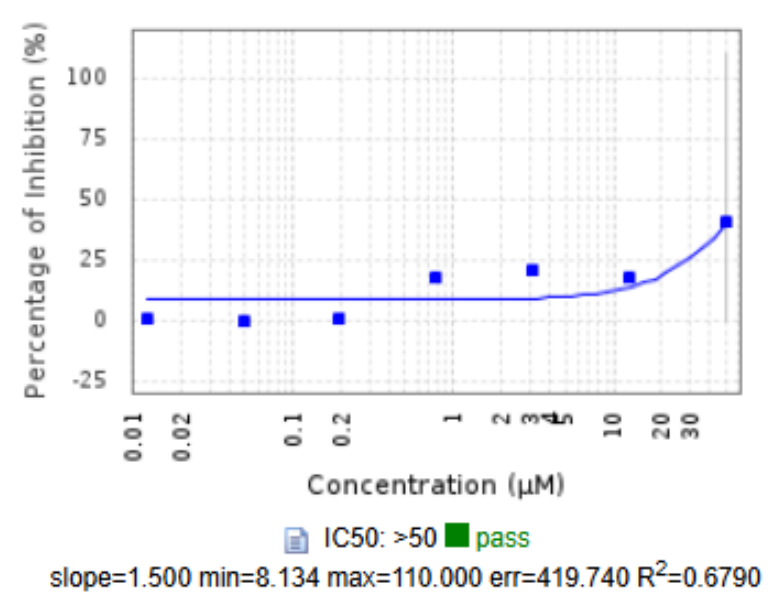

LEM 241422 (5ad)

BJ (IC50 > 50 µM)

Primary screen (50 µM)

plate	well	sample id	PI	pass	conc	inc		
1 (Plate 1)	H9	LEM00241422	9.8532		50.0uM	+		
1 (Plate 1)	H10	LEM00241422	4.0772		50.0uM	+		
1 (Plate 1)	H11	LEM00241422	2.1235		50.0uM	+		

plate	well	sample id	PI	pass	conc	inc		
1 (Plate 1)	H9	LEM00241422	7.83		50.0uM	+		
1 (Plate 1)	H10	LEM00241422	20.217		50.0uM	+		
1 (Plate 1)	H11	LEM00241422	2.9938		50.0uM	+		

MRC5 (IC50 > 50 µM)

Primary screen (50 µM)

plate	well	sample id	PI	pass	conc	inc		
1 (Plate 1)	H9	LEM00241422	21.75		50.0uM	+		
1 (Plate 1)	H10	LEM00241422	21.059		50.0uM	+		
1 (Plate 1)	H11	LEM00241422	22.51		50.0uM	+		

plate	well	sample id	PI	pass	conc	inc		
1 (Plate 1)	H9	LEM00241422	22.312		50.0uM	+		
1 (Plate 1)	H10	LEM00241422	17.686		50.0uM	+		
1 (Plate 1)	H11	LEM00241422	17.045		50.0uM	+		

A549 (IC50 > 50 μM)

Primary screen (50 μM)

plate	well	sample id	PI	pass	conc	inc	✓	⊗
1 (Plate 1)	H9	LEM00241422	15.414		50.0uM	+	✓	⊗
1 (Plate 1)	H10	LEM00241422	23.343		50.0uM	+		
1 (Plate 1)	H11	LEM00241422	13.622		50.0uM	+		

plate	well	sample id	PI	pass	conc	inc	✓	⊗
1 (Plate 1)	H9	LEM00241422	21.637		50.0uM	+	✓	⊗
1 (Plate 1)	H10	LEM00241422	25.303		50.0uM	+		
1 (Plate 1)	H11	LEM00241422	19.542		50.0uM	+		

CCRF-CEM (IC50 36 μM)

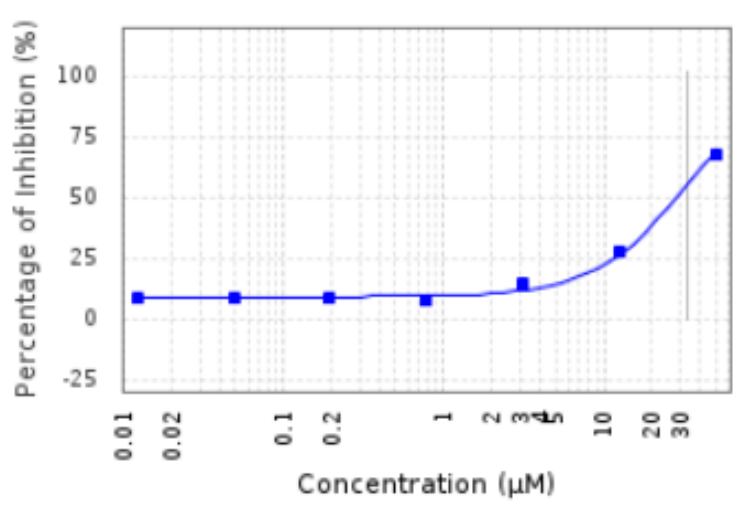

IC50: 33.605 (31.277 - 36.107) pass
slope=1.500 min=8.916 max=102.000 err=15.044 R^2=0.9948

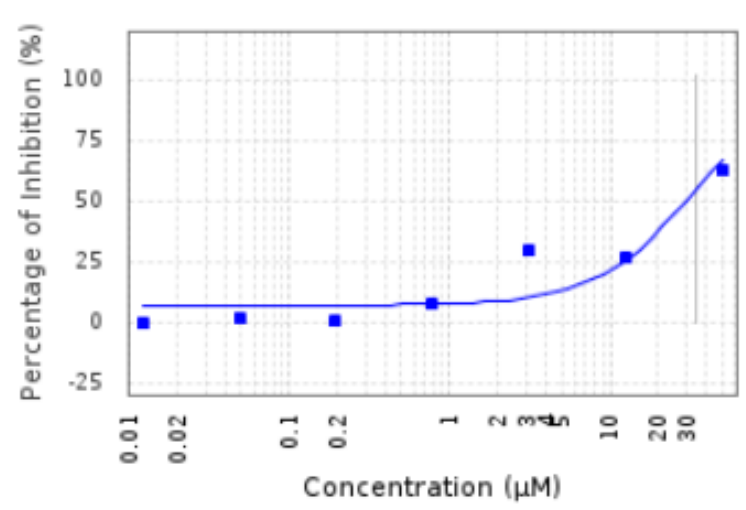

IC50: 34.509 (23.029 - 51.71) pass
slope=1.400 min=6.785 max=102.000 err=509.303 R^2=0.8432

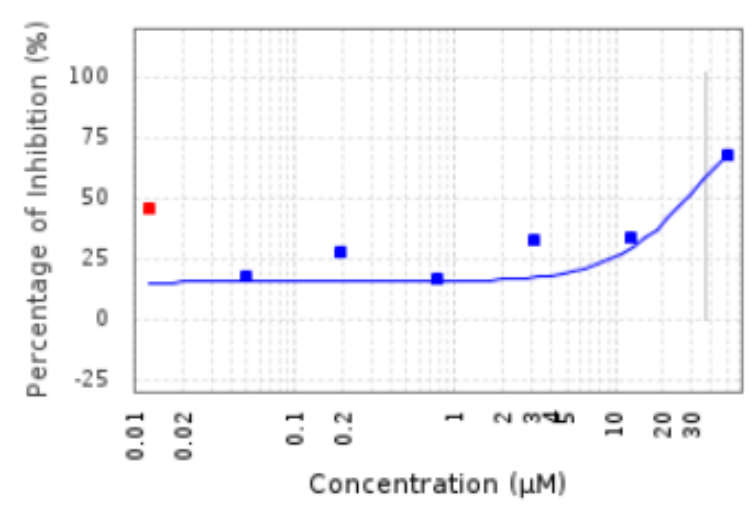

IC50: 37.217 (23.524 - 58.881) pass
slope=1.500 min=15.000 max=102.000 err=403.981 R^2=0.7685

HCT116 (IC50 > 50 μM)

Primary screen (50 μM)

plate	well	sample id	PI	pass	conc	inc	✓	⊗
1 (Plate 1)	H9	LEM00241422	21.718		50.0uM	+	✓	⊗
1 (Plate 1)	H10	LEM00241422	20.751		50.0uM	+		
1 (Plate 1)	H11	LEM00241422	14.147		50.0uM	+		

plate	well	sample id	PI	pass	conc	inc	✓	⊗
1 (Plate 1)	H9	LEM00241422	12.226		50.0uM	+	✓	⊗
1 (Plate 1)	H10	LEM00241422	19.599		50.0uM	+		
1 (Plate 1)	H11	LEM00241422	17.115		50.0uM	+		

HCT116p53-/- (IC50 > 50 μM)

Primary screen (50 μM)

plate	well	sample id	PI	pass	conc	inc	✓	⊗
1 (Plate 1)	H9	LEM00241422	32.384		50.0uM	+	✓	⊗
1 (Plate 1)	H10	LEM00241422	17.293		50.0uM	+		
1 (Plate 1)	H11	LEM00241422	28.719		50.0uM	+		

plate	well	sample id	PI	pass	conc	inc	✓	⊗
1 (Plate 1)	H9	LEM00241422	13.226		50.0uM	+	✓	⊗
1 (Plate 1)	H10	LEM00241422	18.44		50.0uM	+		
1 (Plate 1)	H11	LEM00241422	20.894		50.0uM	+		

K562 (IC50 > 50 μM)

Primary screen (50 µM)

plate	well	sample id	PI	pass	conc	inc		
1 (Plate 1)	H9	LEM00241422	24.213		50.0uM	+	✓	⊗
1 (Plate 1)	H10	LEM00241422	21.569		50.0uM	+		
1 (Plate 1)	H11	LEM00241422	19.746		50.0uM	+		

plate	well	sample id	PI	pass	conc	inc		
1 (Plate 1)	H9	LEM00241422	7.6165		50.0uM	+	✓	⊗
1 (Plate 1)	H10	LEM00241422	8.1698		50.0uM	+		
1 (Plate 1)	H11	LEM00241422	12.928		50.0uM	+		

U2OS (IC50 28 µM)

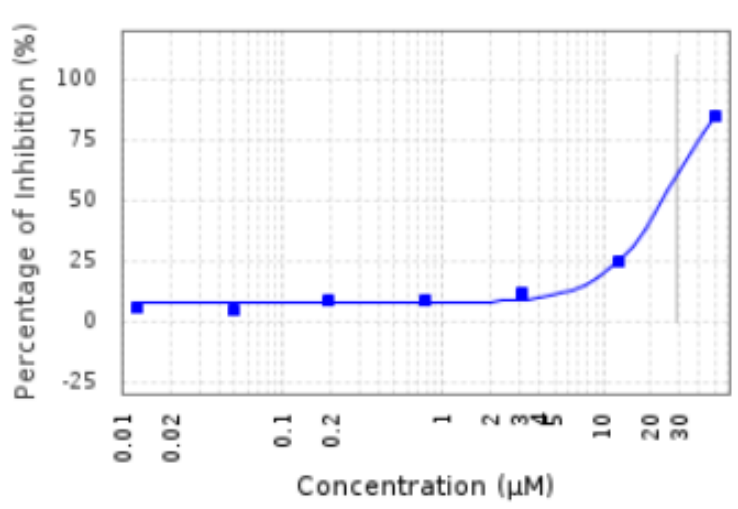

IC50: 28.378 *(5.6809 - 141.76)* pass
slope=1.946 min=7.391 max=110.000 err=24.118 R^2=0.9952

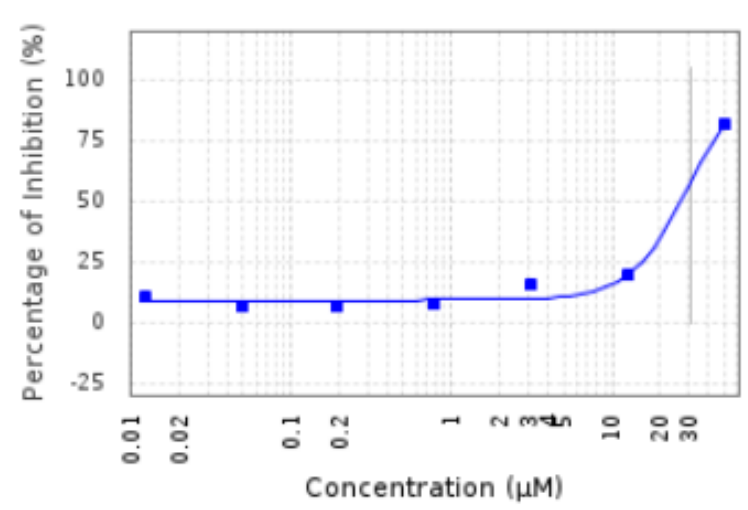

IC50: 30.78 *(27.382 - 34.601)* pass
slope=2.314 min=8.987 max=105.000 err=53.410 R^2=0.9879

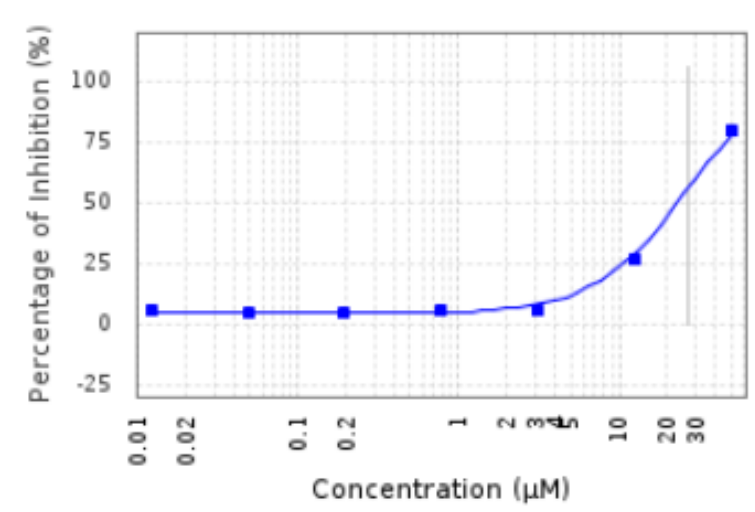

IC50: 26.589 *(24.735 - 28.582)* pass
slope=1.500 min=4.029 max=106.000 err=20.339 R^2=0.9957

LEM 230903 (5ba)

BJ (IC50 > 50 µM)

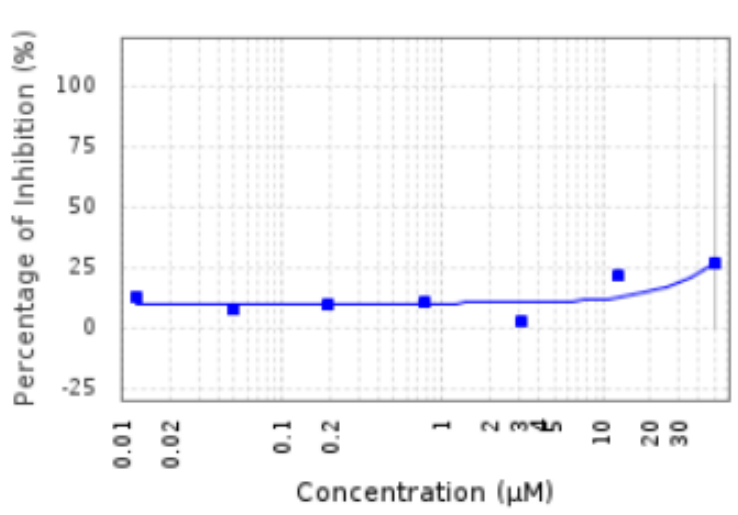

IC50: >50 pass
slope=1.500 min=9.913 max=100.830 err=152.982 R^2=0.6293

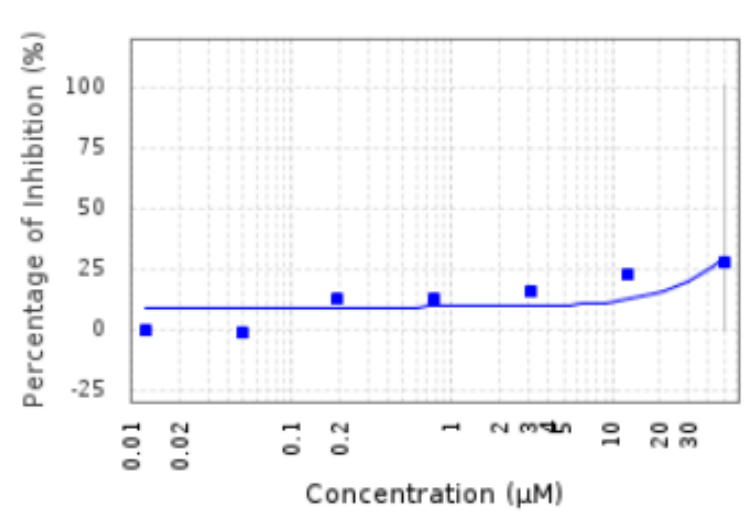

IC50: >50 pass
slope=1.500 min=8.958 max=100.004 err=381.556 R^2=0.4692

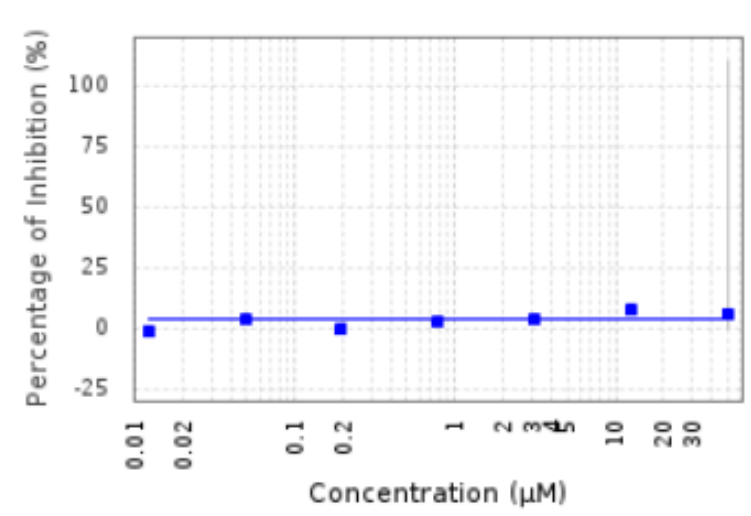

IC50: >50 pass
slope=1.500 min=3.052 max=110.000 err=52.965 R^2=0.0288

MRC5 (IC50 > 50 µM)

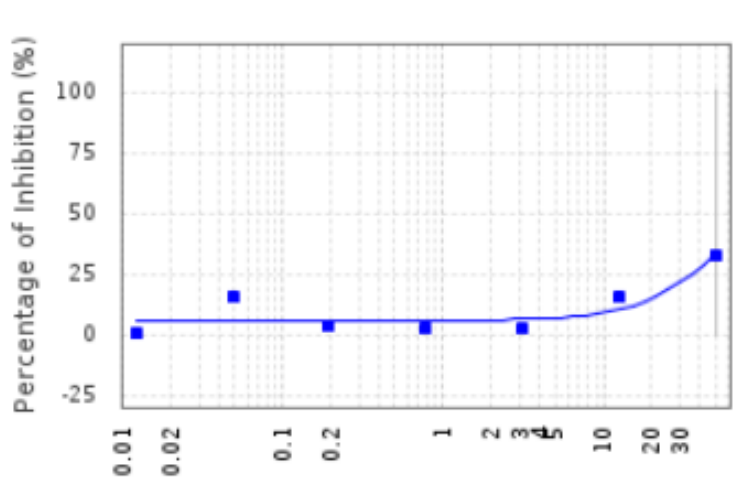

IC50: >50 pass
slope=1.500 min=5.523 max=100.004 err=175.985 R^2=0.7850

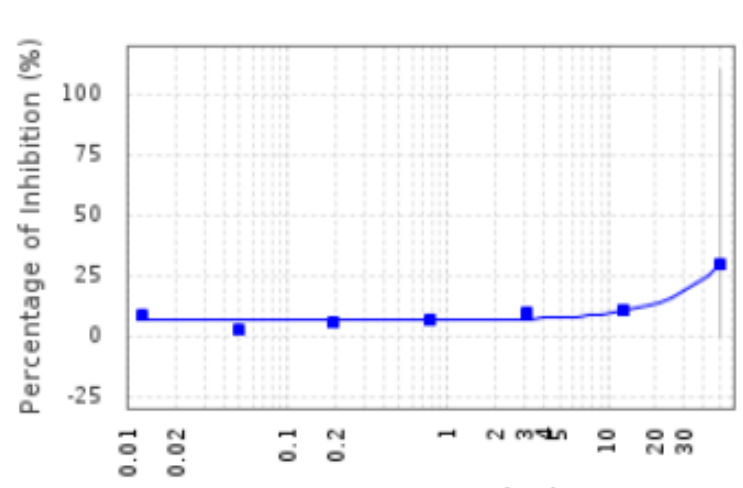

IC50: >50 pass
slope=1.500 min=6.445 max=110.000 err=28.059 R^2=0.9416

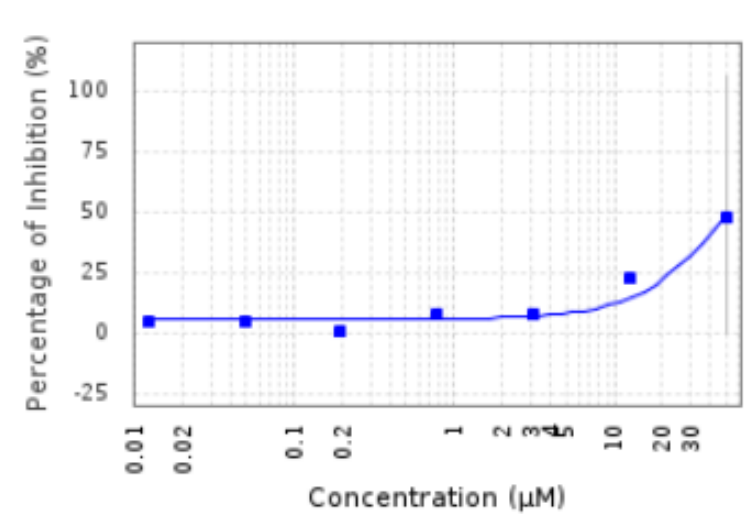

IC50: >50 pass
slope=1.500 min=5.476 max=105.871 err=98.596 R^2=0.9392

A549 (IC50 > 50 µM)

Primary screen (50 μM)

plate	well	sample id	PI	pass	conc	inc	✓	✗
1 (Plate 1)	L3	LEM00230903	1.2845		50.0uM	+	✓	✗
1 (Plate 1)	L4	LEM00230903	5.9313		50.0uM	+		
1 (Plate 1)	L5	LEM00230903	9.2504		50.0uM	+		

plate	well	sample id	PI	pass	conc	inc	✓	✗
1 (Plate 1)	L3	LEM00230903	10.146		50.0uM	+	✓	✗
1 (Plate 1)	L4	LEM00230903	11.867		50.0uM	+		
1 (Plate 1)	L5	LEM00230903	12.85		50.0uM	+		

CCRF-CEM (IC50 5.3 μM)

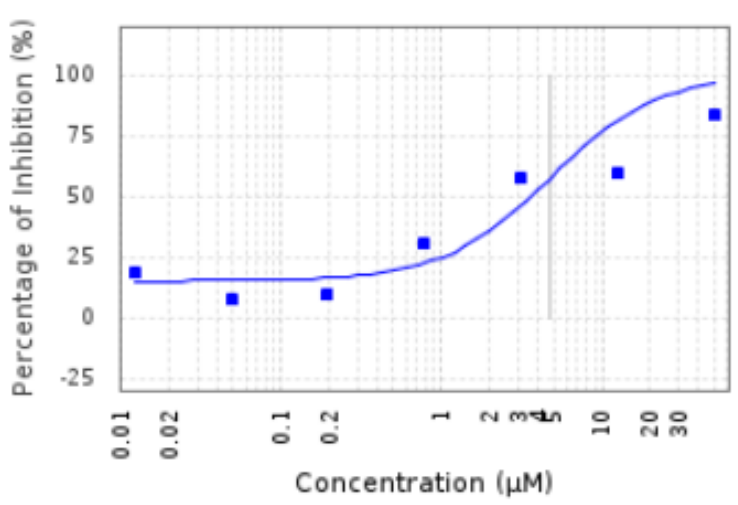

IC50: 4.7703 (1.9183 - 11.863) pass
slope=1.300 min=14.912 max=100.000 err=924.218 R^2=0.8182

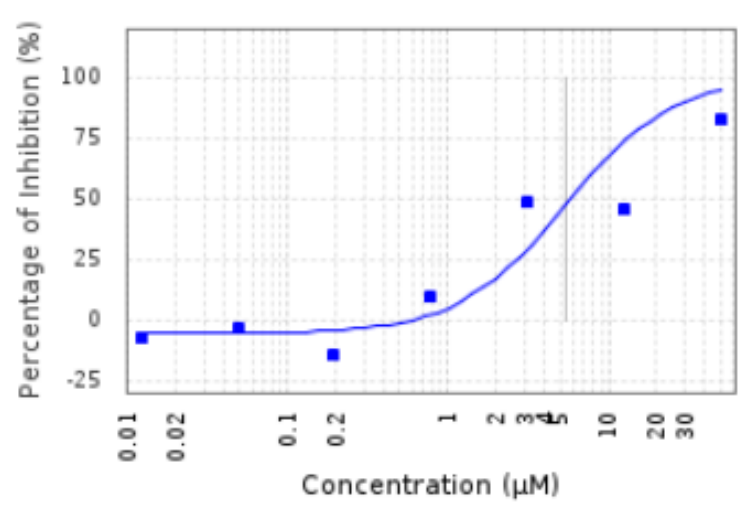

IC50: 5.3728 (2.11 - 13.681) pass
slope=1.300 min=-5.858 max=100.000 err=1442.629 R^2=0.8182

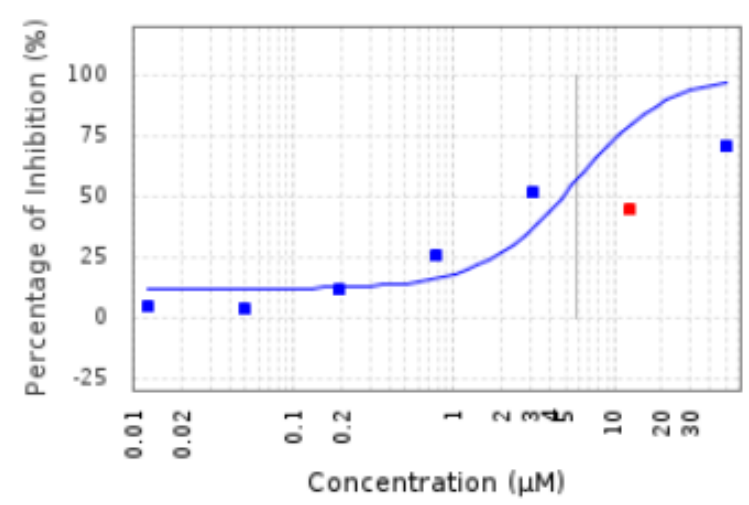

IC50: 5.7757 (0.2309 - 144.45) pass
slope=1.500 min=11.648 max=100.000 err=1081.725 R^2=0.7166

HCT116 (IC50 > 50 μM)

Primary screen (50 μM)

plate	well	sample id	PI	pass	conc	inc	✓	✗
1 (Plate 1)	L3	LEM00230903	17.723		50.0uM	+	✓	✗
1 (Plate 1)	L4	LEM00230903	20.251		50.0uM	+		
1 (Plate 1)	L5	LEM00230903	17.975		50.0uM	+		

plate	well	sample id	PI	pass	conc	inc	✓	✗
1 (Plate 1)	L3	LEM00230903	14.41		50.0uM	+	✓	✗
1 (Plate 1)	L4	LEM00230903	12.023		50.0uM	+		
1 (Plate 1)	L5	LEM00230903	16.298		50.0uM	+		

HCT116p53-/- (IC50 > 50 μM)

Primary screen (50 μM)

plate	well	sample id	PI	pass	conc	inc	✓	✗
1 (Plate 1)	L3	LEM00230903	16.358		50.0uM	+	✓	✗
1 (Plate 1)	L4	LEM00230903	21.34		50.0uM	+		
1 (Plate 1)	L5	LEM00230903	17		50.0uM	+		

plate	well	sample id	PI	pass	conc	inc	✓	✗
1 (Plate 1)	L3	LEM00230903	19.663		50.0uM	+	✓	✗
1 (Plate 1)	L4	LEM00230903	15.945		50.0uM	+		
1 (Plate 1)	L5	LEM00230903	19.043		50.0uM	+		

K562 (IC50 > 50 μM)

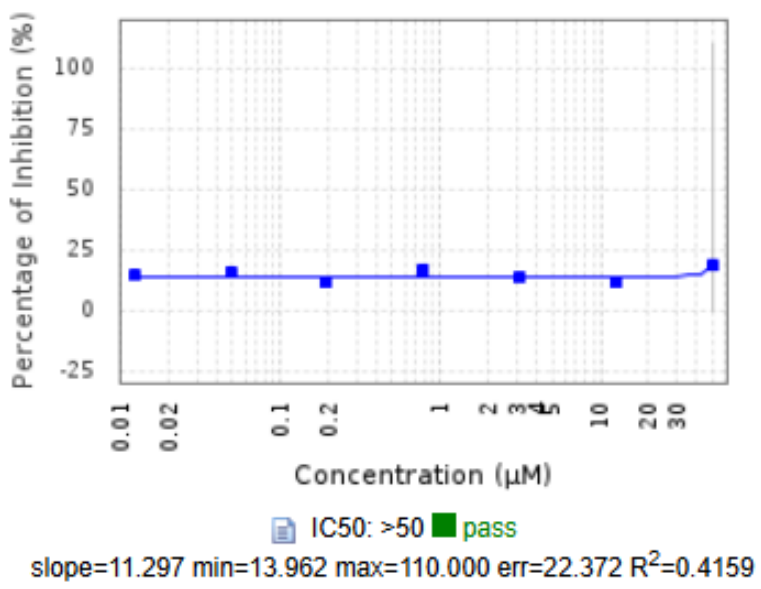

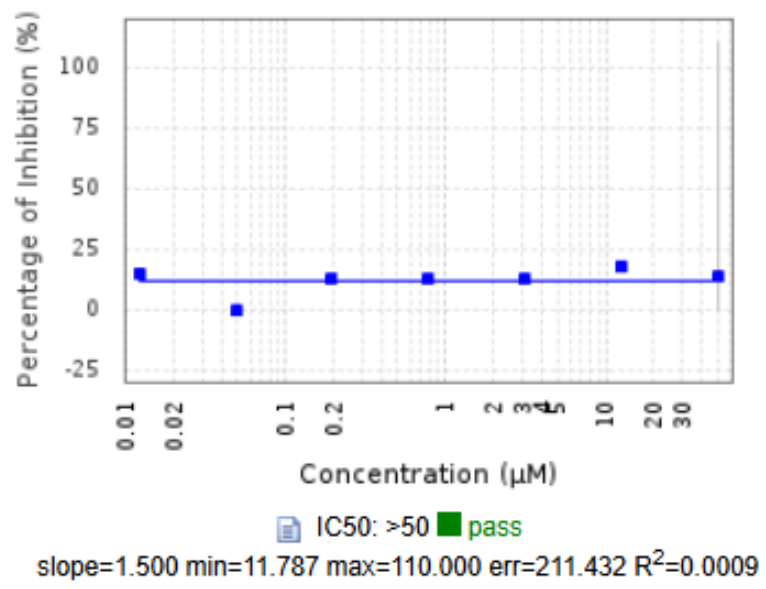

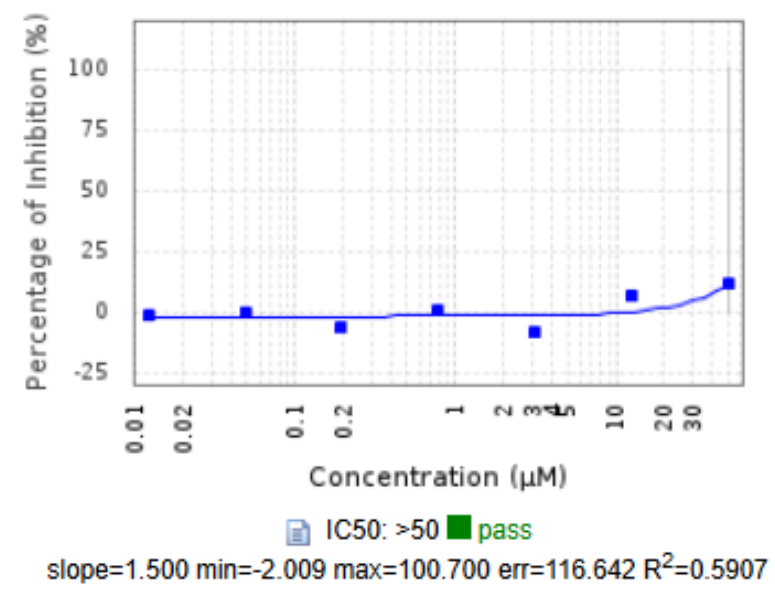

U2OS (IC50 > 50 μM)

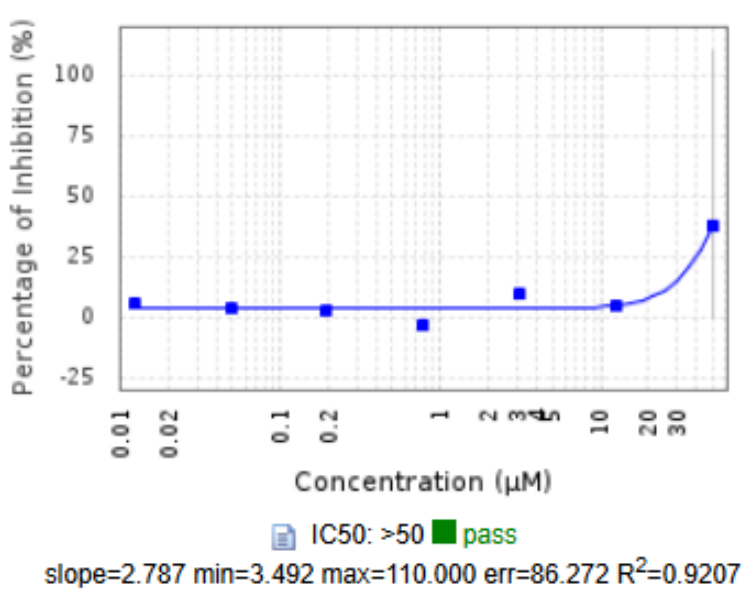

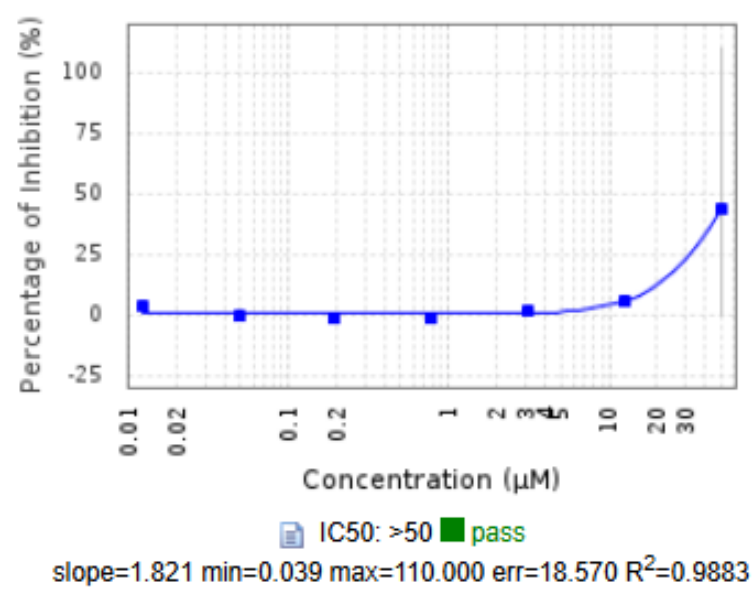

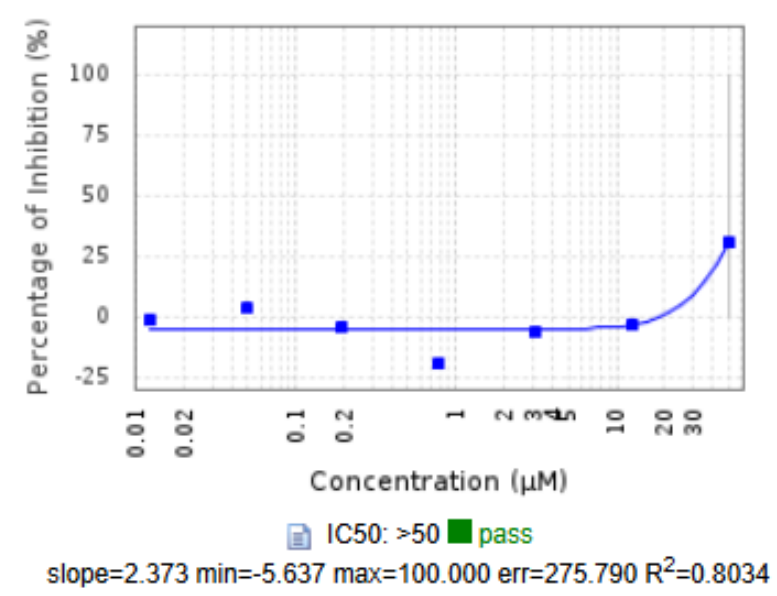

LEM 241452 (5bb)

BJ (IC50 > 50 μM)

Primary screen (50 μM)

plate	well	sample id	PI	pass	conc	inc	✓	⊗
1 (Plate 1)	G12	LEM00241452	6.7104	●	50.0uM	+	✓	⊗
1 (Plate 1)	G13	LEM00241452	10.193	●	50.0uM	+		
1 (Plate 1)	G14	LEM00241452	7.8995	●	50.0uM	+		

plate	well	sample id	PI	pass	conc	inc	✓	⊗
1 (Plate 1)	G12	LEM00241452	8.5087	●	50.0uM	+	✓	⊗
1 (Plate 1)	G13	LEM00241452	4.7756	●	50.0uM	+		
1 (Plate 1)	G14	LEM00241452	4.6059	●	50.0uM	+		

MRC5 (IC50 > 50 μM)

Primary screen (50 μM)

plate	well	sample id	PI	pass	conc	inc	✓	⊗
1 (Plate 1)	G12	LEM00241452	22.648	●	50.0uM	+	✓	⊗
1 (Plate 1)	G13	LEM00241452	21.542	●	50.0uM	+		
1 (Plate 1)	G14	LEM00241452	21.404	●	50.0uM	+		

plate	well	sample id	PI	pass	conc	inc	✓	⊗
1 (Plate 1)	G12	LEM00241452	19.75	●	50.0uM	+	✓	⊗
1 (Plate 1)	G13	LEM00241452	19.465	●	50.0uM	+		
1 (Plate 1)	G14	LEM00241452	19.038	●	50.0uM	+		

A549 (IC50 > 50 μM)

Primary screen (50 µM)

plate	well	sample id	PI	pass	conc	inc	✓	⊗
1 (Plate 1)	G12	LEM00241452	5.8308	🔴	50.0uM	+	✓	⊗
1 (Plate 1)	G13	LEM00241452	5.9687	🔴	50.0uM	+		
1 (Plate 1)	G14	LEM00241452	6.9339	🔴	50.0uM	+		

plate	well	sample id	PI	pass	conc	inc	✓	⊗
1 (Plate 1)	G12	LEM00241452	10.442	🔴	50.0uM	+	✓	⊗
1 (Plate 1)	G13	LEM00241452	9.5254	🔴	50.0uM	+		
1 (Plate 1)	G14	LEM00241452	9.2635	🔴	50.0uM	+		

CCRF-CEM (IC50 > 50 µM)

Primary screen (50 µM)

plate	well	sample id	PI	pass	conc	inc	✓	⊗
1 (Plate 1)	G12	LEM00241452	48.069	🔴	50.0uM	+	✓	⊗
1 (Plate 1)	G13	LEM00241452	42.499	🔴	50.0uM	+		
1 (Plate 1)	G14	LEM00241452	36.161	🔴	50.0uM	+		

plate	well	sample id	PI	pass	conc	inc	✓	⊗
1 (Plate 1)	G12	LEM00241452	45.106	🔴	50.0uM	+	✓	⊗
1 (Plate 1)	G13	LEM00241452	49.481	🔴	50.0uM	+		
1 (Plate 1)	G14	LEM00241452	53.26	🟢	50.0uM	+		

HCT116 (IC50 > 50 µM)

Primary screen (50 µM)

plate	well	sample id	PI	pass	conc	inc	✓	⊗
1 (Plate 1)	G12	LEM00241452	1.3405	🔴	50.0uM	+	✓	⊗
1 (Plate 1)	G13	LEM00241452	1.7432	🔴	50.0uM	+		
1 (Plate 1)	G14	LEM00241452	4.9649	🔴	50.0uM	+		

plate	well	sample id	PI	pass	conc	inc	✓	⊗
1 (Plate 1)	G12	LEM00241452	6.6384	🔴	50.0uM	+	✓	⊗
1 (Plate 1)	G13	LEM00241452	6.2504	🔴	50.0uM	+		
1 (Plate 1)	G14	LEM00241452	1.5163	🔴	50.0uM	+		

HCT116p53-/- (IC50 > 50 µM)

Primary screen (50 µM)

plate	well	sample id	PI	pass	conc	inc	✓	⊗
1 (Plate 1)	G12	LEM00241452	-0.3850	🔴	50.0uM	+	✓	⊗
1 (Plate 1)	G13	LEM00241452	0.6929	🔴	50.0uM	+		
1 (Plate 1)	G14	LEM00241452	-0.7083	🔴	50.0uM	+		

plate	well	sample id	PI	pass	conc	inc	✓	⊗
1 (Plate 1)	G12	LEM00241452	4.024	🔴	50.0uM	+	✓	⊗
1 (Plate 1)	G13	LEM00241452	8.3181	🔴	50.0uM	+		
1 (Plate 1)	G14	LEM00241452	4.9441	🔴	50.0uM	+		

K562 (IC50 > 50 µM)

Primary screen (50 µM)

plate	well	sample id	PI	pass	conc	inc	✓	⊗
1 (Plate 1)	G12	LEM00241452	11.266		50.0uM	+	✓	⊗
1 (Plate 1)	G13	LEM00241452	6.9813		50.0uM	+		
1 (Plate 1)	G14	LEM00241452	8.6224		50.0uM	+		

plate	well	sample id	PI	pass	conc	inc	✓	⊗
1 (Plate 1)	G12	LEM00241452	-7.1001		50.0uM	+	✓	⊗
1 (Plate 1)	G13	LEM00241452	2.3052		50.0uM	+		
1 (Plate 1)	G14	LEM00241452	0.4242		50.0uM	+		

U2OS (IC50 > 50 µM)

Primary screen (50 µM)

plate	well	sample id	PI	pass	conc	inc	✓	⊗
1 (Plate 1)	G12	LEM00241452	15.213		50.0uM	+	✓	⊗
1 (Plate 1)	G13	LEM00241452	15.847		50.0uM	+		
1 (Plate 1)	G14	LEM00241452	15.937		50.0uM	+		

plate	well	sample id	PI	pass	conc	inc	✓	⊗
1 (Plate 1)	G12	LEM00241452	18.276		50.0uM	+	✓	⊗
1 (Plate 1)	G13	LEM00241452	17.175		50.0uM	+		
1 (Plate 1)	G14	LEM00241452	14.467		50.0uM	+		

LEM 241423 (5bc)

BJ (IC50 3.1 µM)

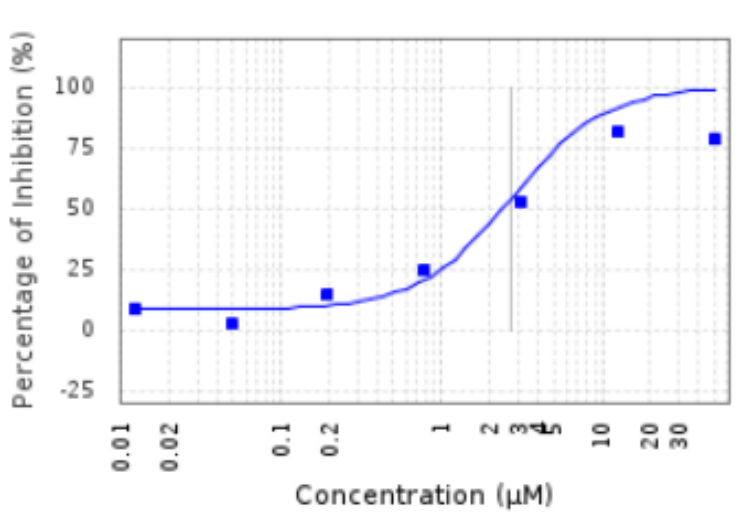

IC50: 2.7255 *(1.4843 - 5.0046)* pass
slope=1.500 min=8.243 max=100.000 err=623.549 R^2=0.9048

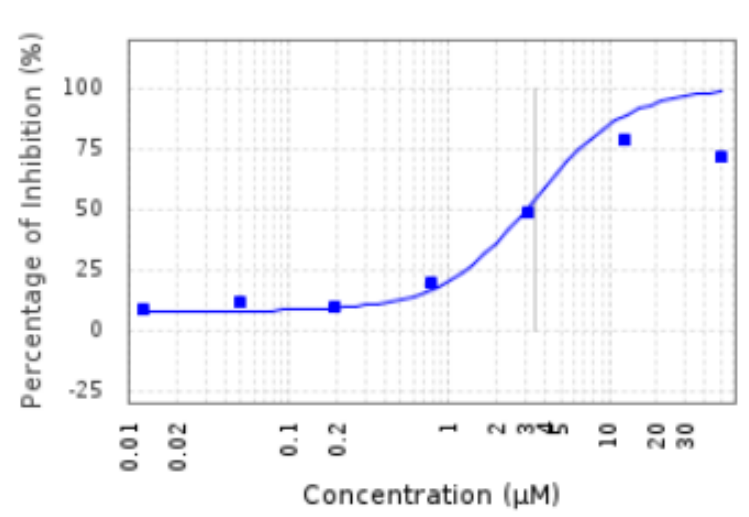

IC50: 3.4363 *(1.6486 - 7.1625)* pass
slope=1.500 min=7.623 max=100.000 err=833.121 R^2=0.8499

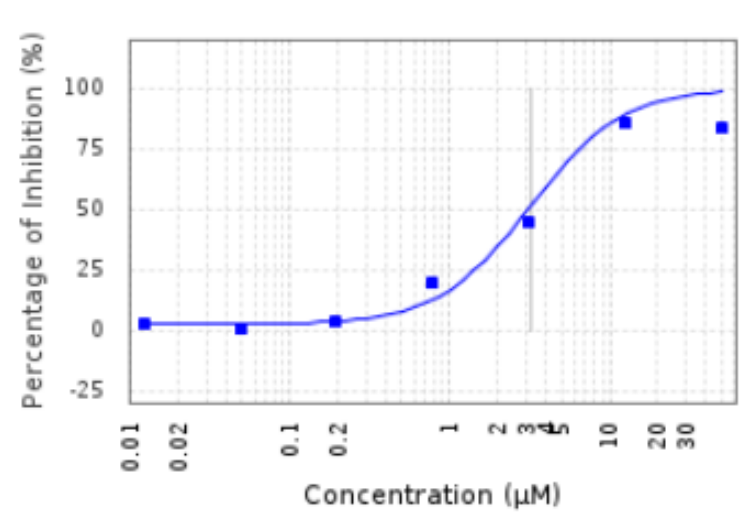

IC50: 3.1985 *(2.0794 - 4.9197)* pass
slope=1.500 min=2.043 max=100.000 err=336.201 R^2=0.9601

MRC5 (IC50 1.5 µM)

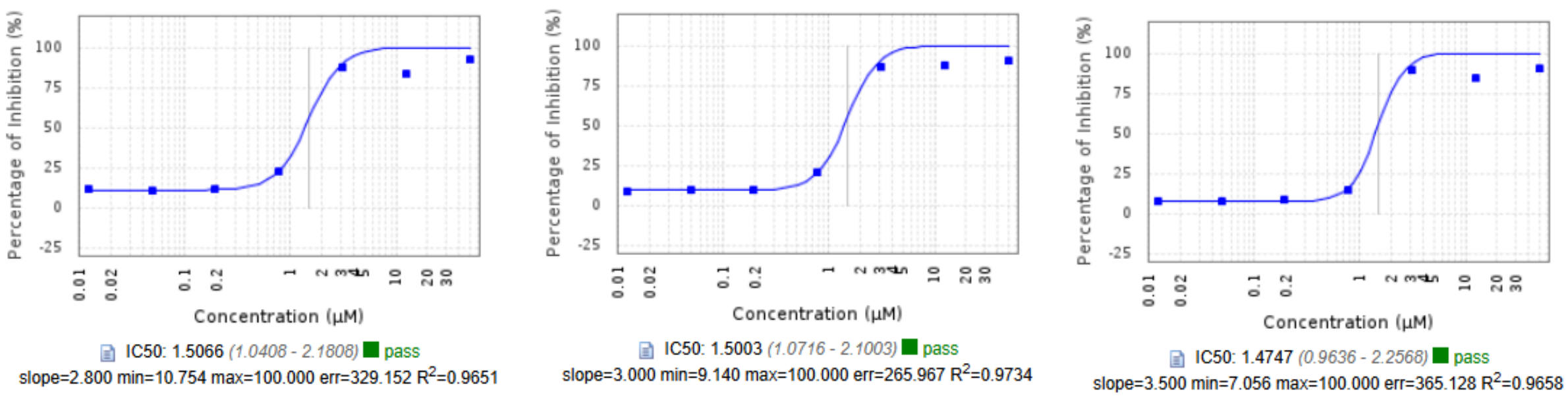

A549 (IC50 0.91 μM)

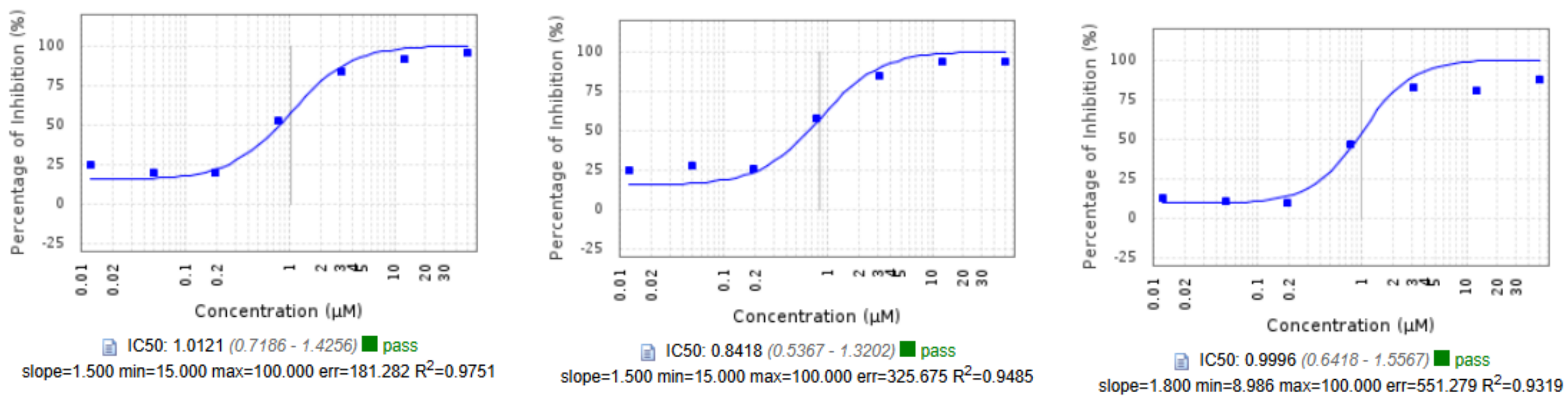

CCRF-CEM (IC50 0.51 μM)

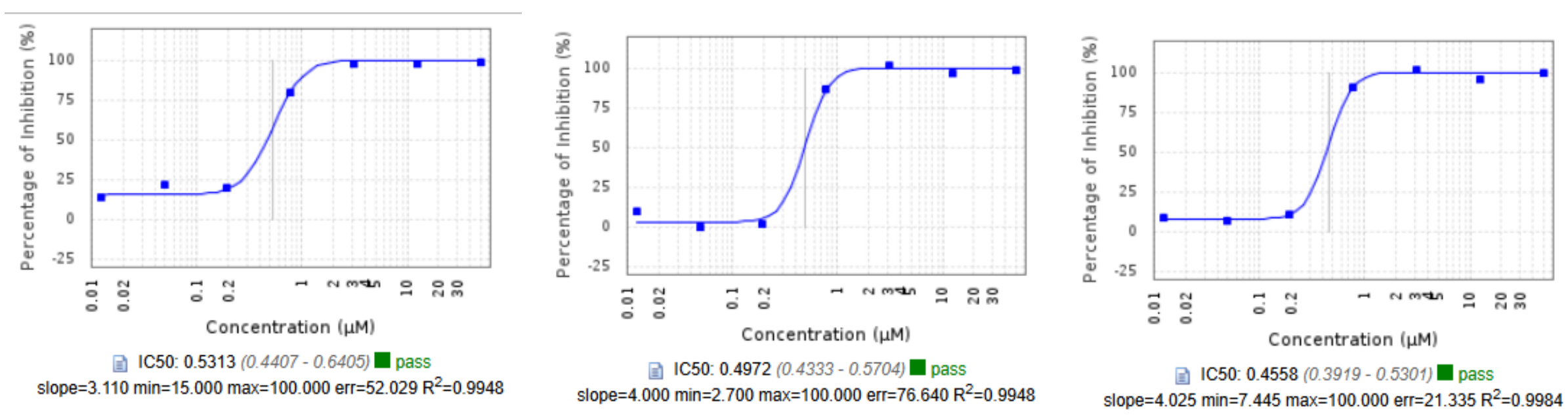

HCT116 (IC50 0.78 μM)

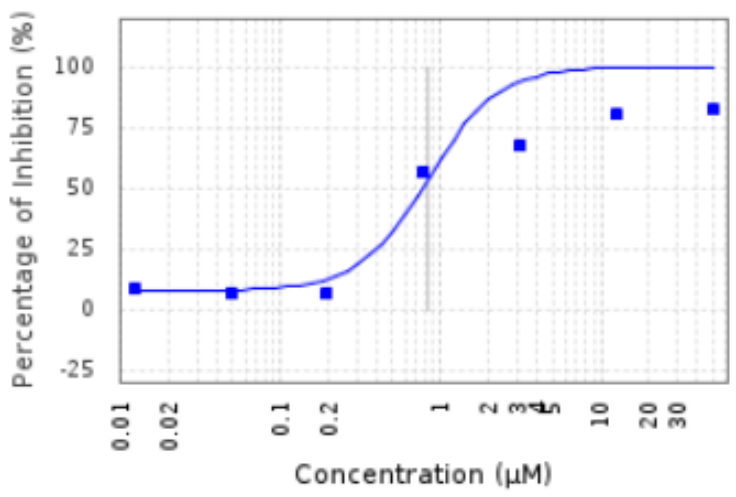

IC50: 0.8351 *(0.4513 - 1.5452)* pass
slope=2.000 min=7.471 max=100.000 err=1438.865 R^2=0.8091

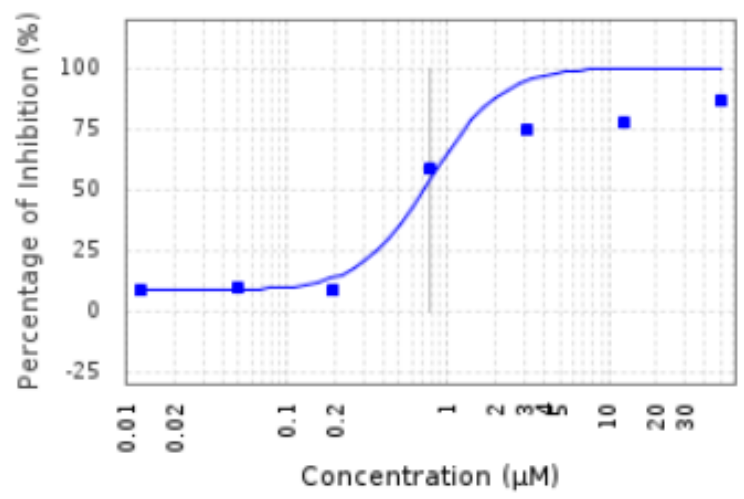

IC50: 0.7797 *(0.4534 - 1.3408)* pass
slope=2.000 min=8.174 max=100.000 err=1107.736 R^2=0.8563

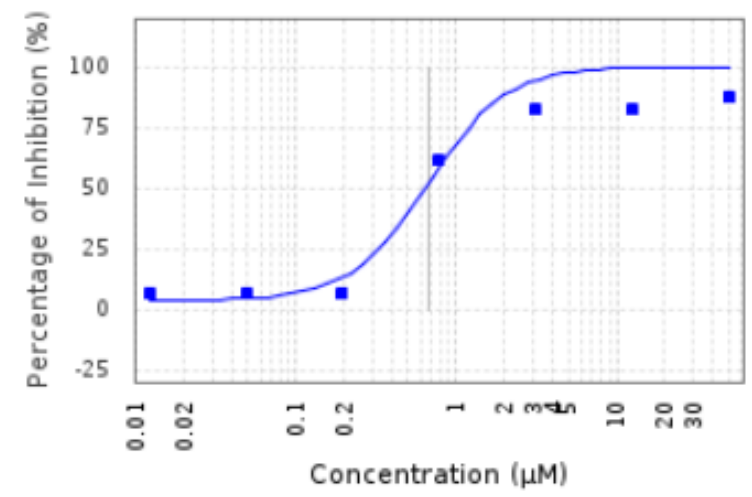

IC50: 0.6671 *(0.4260 - 1.0444)* pass
slope=1.800 min=3.467 max=100.000 err=657.955 R^2=0.9286

HCT116p53-/- (IC50 0.99 µM)

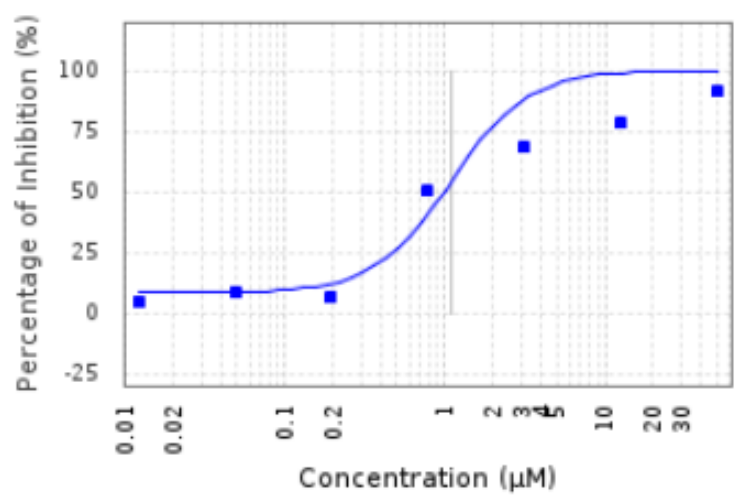

IC50: 1.0844 *(0.5895 - 1.9947)* pass
slope=1.800 min=8.053 max=100.000 err=1022.033 R^2=0.8756

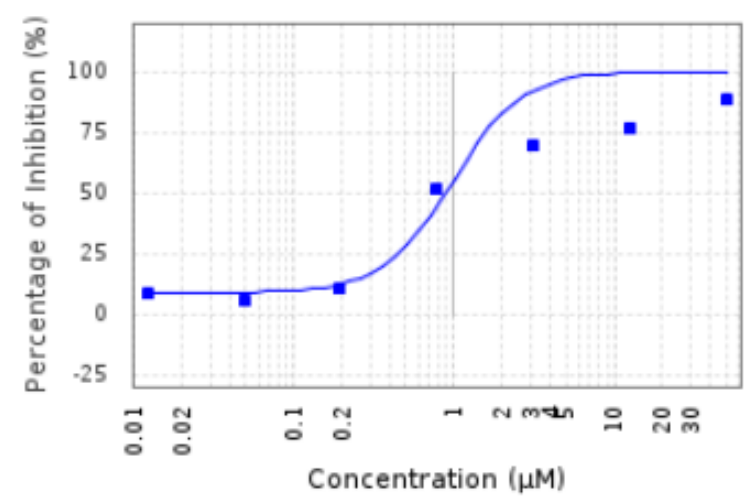

IC50: 0.9712 *(0.5361 - 1.7594)* pass
slope=2.000 min=8.603 max=100.000 err=1224.886 R^2=0.8397

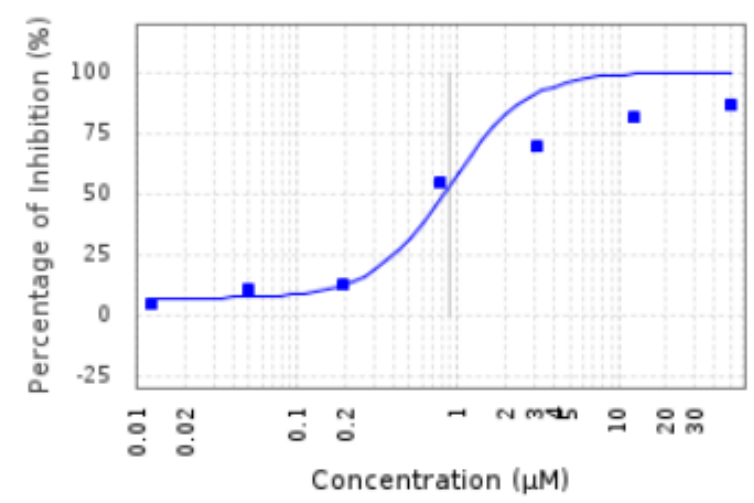

IC50: 0.8864 *(0.4961 - 1.5837)* pass
slope=1.800 min=6.709 max=100.000 err=1039.472 R^2=0.8642

K562 (IC50 0.52 µM)

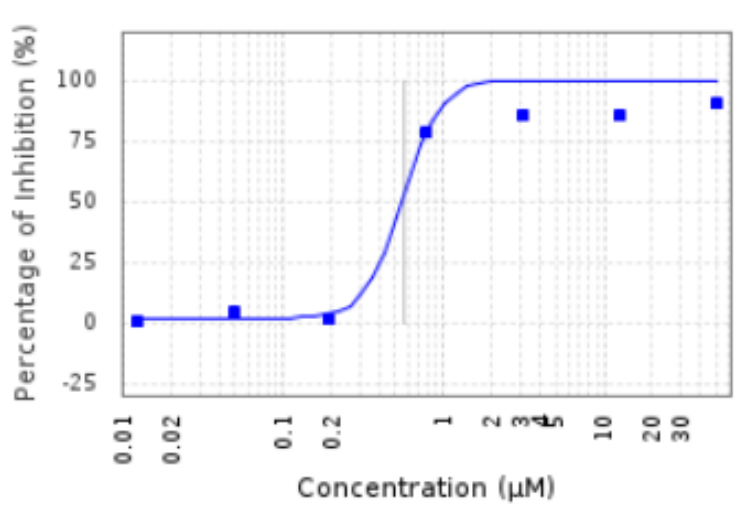

IC50: 0.5555 *(0.4268 - 0.7230)* pass
slope=3.800 min=1.659 max=100.000 err=507.766 R^2=0.9573

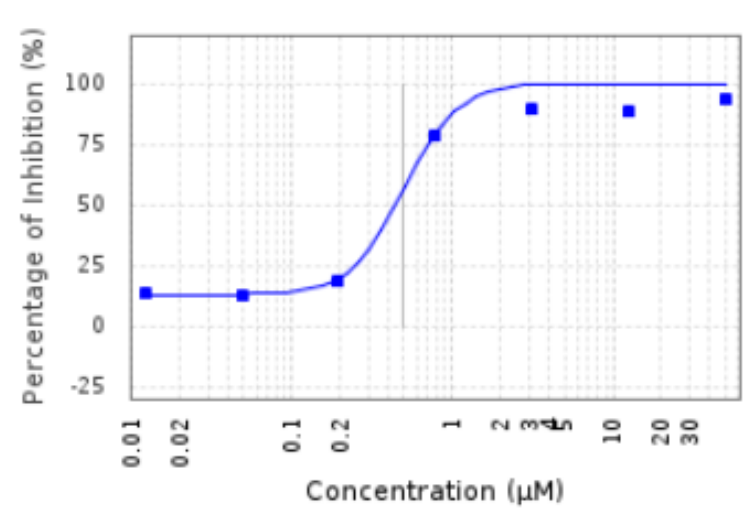

IC50: 0.5000 *(0.3456 - 0.7232)* pass
slope=2.621 min=12.790 max=100.000 err=282.128 R^2=0.9690

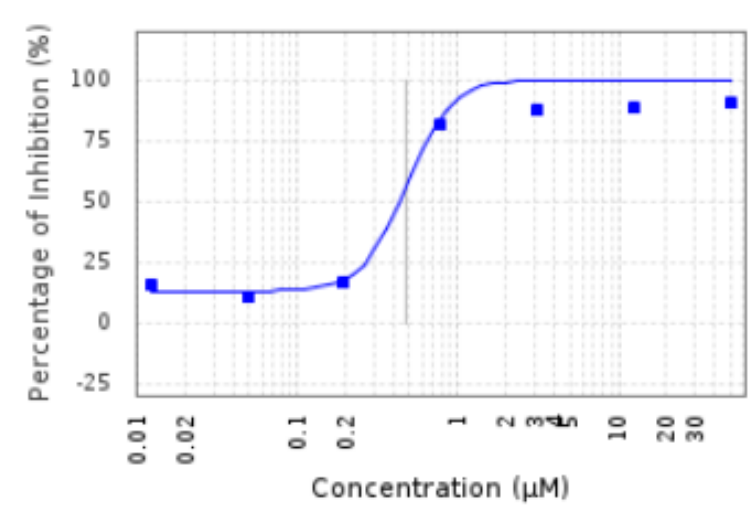

IC50: 0.4815 *(0.2925 - 0.7929)* pass
slope=3.114 min=12.771 max=100.000 err=401.856 R^2=0.9557

U2OS (IC50 0.83 µM)

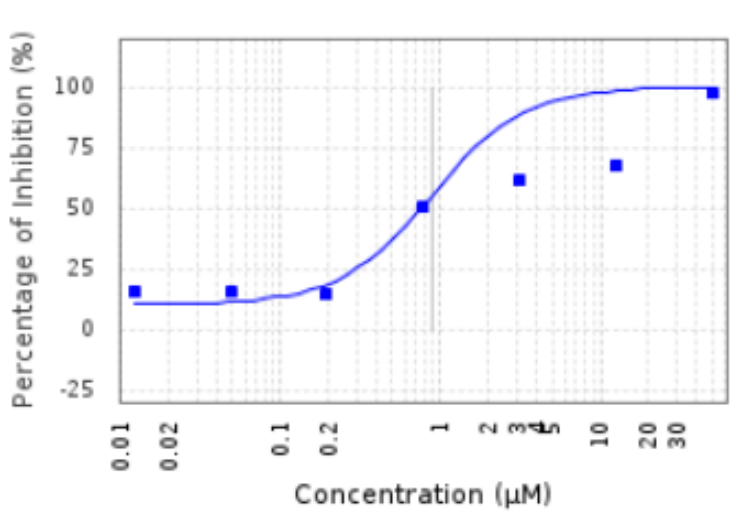

IC50: 0.8840 *(0.3331 - 2.346)* pass
slope=1.500 min=10.112 max=100.000 err=1700.537 R^2=0.7259

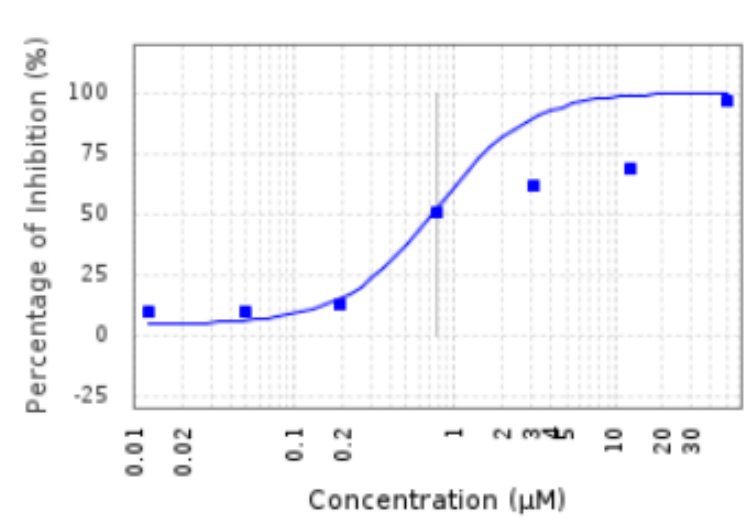

IC50: 0.7730 *(0.3098 - 1.929)* pass
slope=1.500 min=4.295 max=100.000 err=1712.112 R^2=0.7582

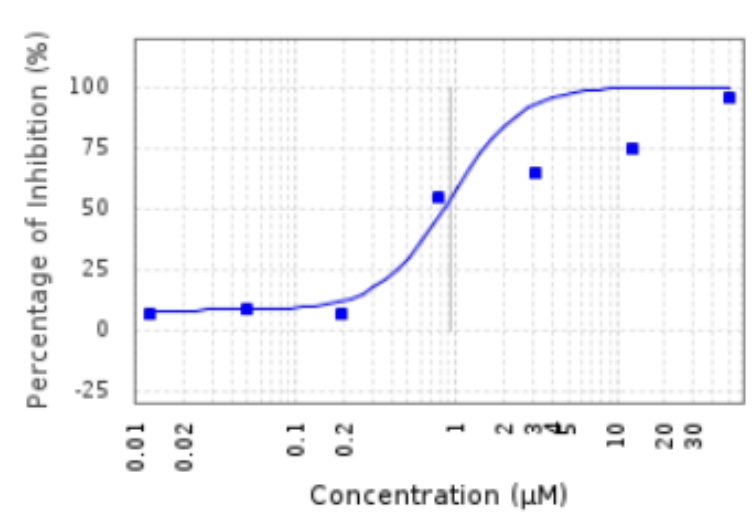

IC50: 0.9129 *(0.4812 - 1.7319)* pass
slope=2.000 min=7.929 max=100.000 err=1495.178 R^2=0.8189

LEM 241424 (5bd)

BJ (IC50 > 50 µM)

Primary screen (50 µM)

plate	well	sample id	PI	pass	conc	inc		
1 (Plate 1)	J9	LEM00241424	20.811		50.0uM	+		
1 (Plate 1)	J10	LEM00241424	20.726		50.0uM	+		
1 (Plate 1)	J11	LEM00241424	19.706		50.0uM	+		

plate	well	sample id	PI	pass	conc	inc		
1 (Plate 1)	J9	LEM00241424	17.078		50.0uM	+		
1 (Plate 1)	J10	LEM00241424	12.412		50.0uM	+		
1 (Plate 1)	J11	LEM00241424	18.096		50.0uM	+		

MRC5 (IC50 > 50 µM)

Primary screen (50 µM)

plate	well	sample id	PI	pass	conc	inc		
1 (Plate 1)	J9	LEM00241424	22.441		50.0uM	+		
1 (Plate 1)	J10	LEM00241424	22.026		50.0uM	+		
1 (Plate 1)	J11	LEM00241424	21.197		50.0uM	+		

plate	well	sample id	PI	pass	conc	inc		
1 (Plate 1)	J9	LEM00241424	23.379		50.0uM	+		
1 (Plate 1)	J10	LEM00241424	22.098		50.0uM	+		
1 (Plate 1)	J11	LEM00241424	18.54		50.0uM	+		

A549 (IC50 > 50 µM)

Primary screen (50 µM)

plate	well	sample id	PI	pass	conc	inc		
1 (Plate 1)	J9	LEM00241424	10.243		50.0uM	+		
1 (Plate 1)	J10	LEM00241424	11.553		50.0uM	+		
1 (Plate 1)	J11	LEM00241424	15.759		50.0uM	+		

plate	well	sample id	PI	pass	conc	inc		
1 (Plate 1)	J9	LEM00241424	16.923		50.0uM	+		
1 (Plate 1)	J10	LEM00241424	17.316		50.0uM	+		
1 (Plate 1)	J11	LEM00241424	18.822		50.0uM	+		

CCRF-CEM (IC50 44.21 µM)

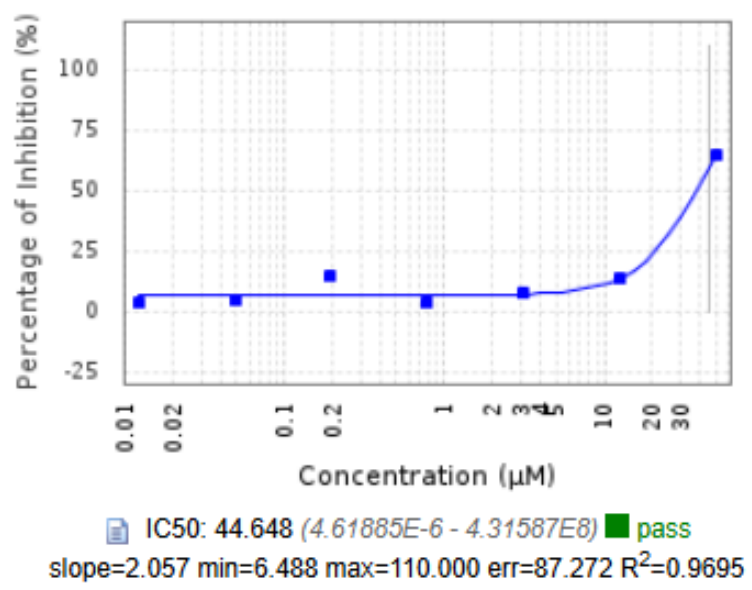

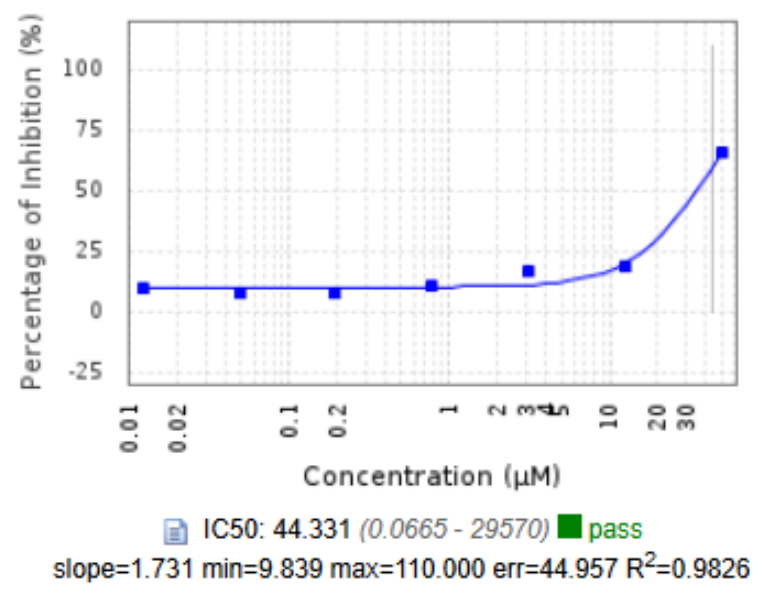

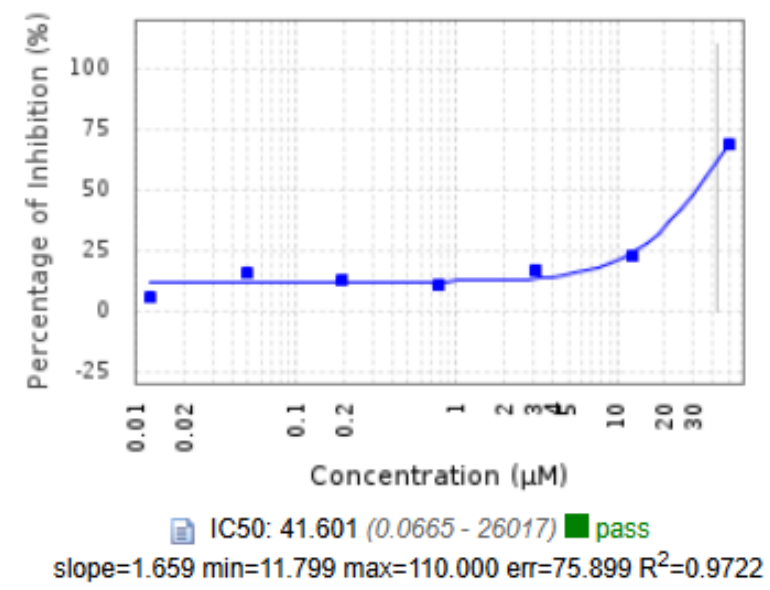

HCT116 (IC50 > 50 µM)

Primary screen (50 µM)

plate	well	sample id	PI	pass	conc	inc	✓	⊗
1 (Plate 1)	J9	LEM00241424	17.61	●	50.0uM	+	✓	⊗
1 (Plate 1)	J10	LEM00241424	15.919	●	50.0uM	+		
1 (Plate 1)	J11	LEM00241424	19.06	●	50.0uM	+		

plate	well	sample id	PI	pass	conc	inc	✓	⊗
1 (Plate 1)	J9	LEM00241424	28.369	●	50.0uM	+	✓	⊗
1 (Plate 1)	J10	LEM00241424	24.721	●	50.0uM	+		
1 (Plate 1)	J11	LEM00241424	17.659	●	50.0uM	+		

HCT116p53-/- (IC50 > 50 µM)

Primary screen (50 µM)

plate	well	sample id	PI	pass	conc	inc	✓	⊗
1 (Plate 1)	J9	LEM00241424	27.857	●	50.0uM	+	✓	⊗
1 (Plate 1)	J10	LEM00241424	38.312	●	50.0uM	+		
1 (Plate 1)	J11	LEM00241424	23.437	●	50.0uM	+		

plate	well	sample id	PI	pass	conc	inc	✓	⊗
1 (Plate 1)	J9	LEM00241424	34.901	●	50.0uM	+	✓	⊗
1 (Plate 1)	J10	LEM00241424	19.565	●	50.0uM	+		
1 (Plate 1)	J11	LEM00241424	19.974	●	50.0uM	+		

K562 (IC50 > 50 µM)

Primary screen (50 µM)

plate	well	sample id	PI	pass	conc	inc	✓	⊗
1 (Plate 1)	J9	LEM00241424	23.94	●	50.0uM	+	✓	⊗
1 (Plate 1)	J10	LEM00241424	32.601	●	50.0uM	+		
1 (Plate 1)	J11	LEM00241424	31.233	●	50.0uM	+		

plate	well	sample id	PI	pass	conc	inc	✓	⊗
1 (Plate 1)	J9	LEM00241424	19.788	●	50.0uM	+	✓	⊗
1 (Plate 1)	J10	LEM00241424	16.469	●	50.0uM	+		
1 (Plate 1)	J11	LEM00241424	16.137	●	50.0uM	+		

U2OS (IC50 > 50 µM)

Primary screen (50 µM)

plate	well	sample id	PI	pass	conc	inc	✓	✗
1 (Plate 1)	J9	LEM00241424	25.256		50.0uM	+	✓	✗
1 (Plate 1)	J10	LEM00241424	27.518		50.0uM	+		
1 (Plate 1)	J11	LEM00241424	27.971		50.0uM	+		

plate	well	sample id	PI	pass	conc	inc	✓	✗
1 (Plate 1)	J9	LEM00241424	26.655		50.0uM	+	✓	✗
1 (Plate 1)	J10	LEM00241424	20.476		50.0uM	+		
1 (Plate 1)	J11	LEM00241424	20.9		50.0uM	+		

LEM 241425 (5ca)

BJ (IC50 > 50 µM)

Primary screen (50 µM)

plate	well	sample id	PI	pass	conc	inc	✓	✗
1 (Plate 1)	K9	LEM00241425	0.0849		50.0uM	+	✓	✗
1 (Plate 1)	K10	LEM00241425	3.4826		50.0uM	+		
1 (Plate 1)	K11	LEM00241425	2.2934		50.0uM	+		

plate	well	sample id	PI	pass	conc	inc	✓	✗
1 (Plate 1)	K9	LEM00241425	2.8241		50.0uM	+	✓	✗
1 (Plate 1)	K10	LEM00241425	-0.9091		50.0uM	+		
1 (Plate 1)	K11	LEM00241425	-4.3029		50.0uM	+		

MRC5 (IC50 > 50 µM)

Primary screen (50 µM)

plate	well	sample id	PI	pass	conc	inc	✓	✗
1 (Plate 1)	K9	LEM00241425	20.506		50.0uM	+	✓	✗
1 (Plate 1)	K10	LEM00241425	21.611		50.0uM	+		
1 (Plate 1)	K11	LEM00241425	22.372		50.0uM	+		

plate	well	sample id	PI	pass	conc	inc	✓	✗
1 (Plate 1)	K9	LEM00241425	18.54		50.0uM	+	✓	✗
1 (Plate 1)	K10	LEM00241425	20.604		50.0uM	+		
1 (Plate 1)	K11	LEM00241425	20.959		50.0uM	+		

A549 (IC50 > 50 µM)

Primary screen (50 µM)

plate	well	sample id	PI	pass	conc	inc	✓	✗
1 (Plate 1)	K9	LEM00241425	6.796		50.0uM	+	✓	✗
1 (Plate 1)	K10	LEM00241425	6.4513		50.0uM	+		
1 (Plate 1)	K11	LEM00241425	8.8644		50.0uM	+		

plate	well	sample id	PI	pass	conc	inc	✓	✗
1 (Plate 1)	K9	LEM00241425	12.668		50.0uM	+	✓	✗
1 (Plate 1)	K10	LEM00241425	14.37		50.0uM	+		
1 (Plate 1)	K11	LEM00241425	14.239		50.0uM	+		

CCRF-CEM (IC50 37 µM)

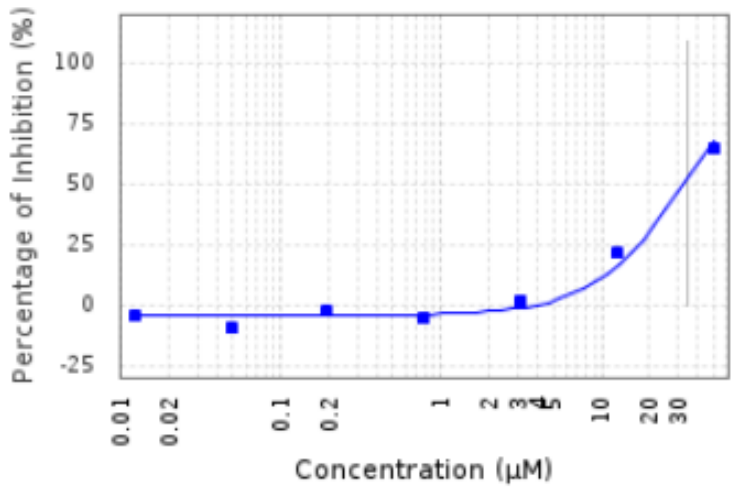

IC50: 34.343 *(2.0252 - 582.37)* pass
slope=1.500 min=-4.525 max=108.096 err=77.187 R^2=0.9813

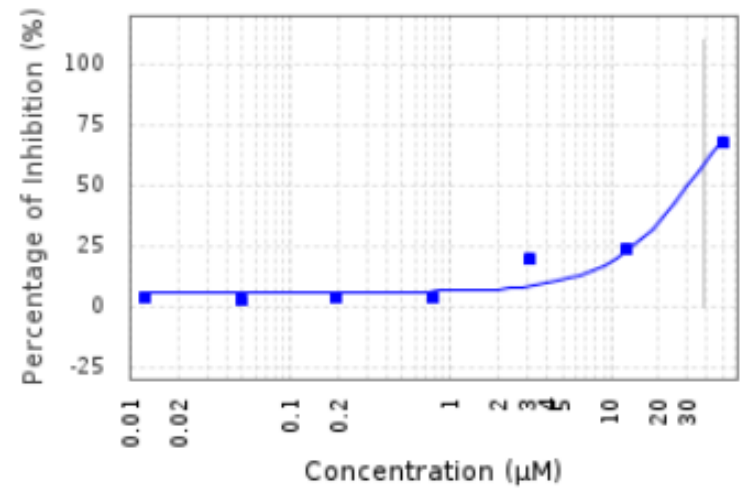

IC50: 37.595 *(0.1844 - 7665.9)* pass
slope=1.500 min=5.720 max=109.737 err=148.187 R^2=0.9557

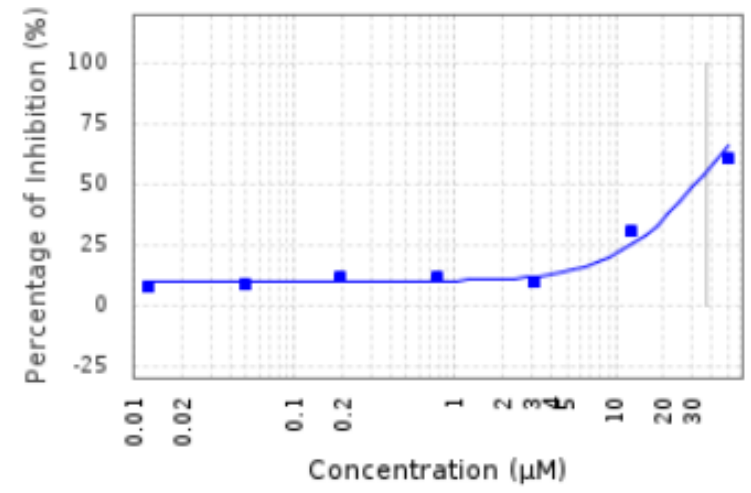

IC50: 36.627 *(0.7980 - 1681.1)* pass
slope=1.500 min=9.515 max=100.000 err=66.104 R^2=0.9713

HCT116 (IC50 > 50 µM)

Primary screen (50 µM)

plate	well	sample id	PI	pass	conc	inc	✓	⊗
1 (Plate 1)	K9	LEM00241425	25.262		50.0uM	+	✓	⊗
1 (Plate 1)	K10	LEM00241425	22.523		50.0uM	+		
1 (Plate 1)	K11	LEM00241425	26.228		50.0uM	+		

plate	well	sample id	PI	pass	conc	inc	✓	⊗
1 (Plate 1)	K9	LEM00241425	24.022		50.0uM	+	✓	⊗
1 (Plate 1)	K10	LEM00241425	20.685		50.0uM	+		
1 (Plate 1)	K11	LEM00241425	26.195		50.0uM	+		

HCT116p53-/- (IC50 > 50 µM)

Primary screen (50 µM)

plate	well	sample id	PI	pass	conc	inc	✓	⊗
1 (Plate 1)	K9	LEM00241425	10.502		50.0uM	+	✓	⊗
1 (Plate 1)	K10	LEM00241425	0.8007		50.0uM	+		
1 (Plate 1)	K11	LEM00241425	16.538		50.0uM	+		

plate	well	sample id	PI	pass	conc	inc	✓	⊗
1 (Plate 1)	K9	LEM00241425	10.976		50.0uM	+	✓	⊗
1 (Plate 1)	K10	LEM00241425	17.111		50.0uM	+		
1 (Plate 1)	K11	LEM00241425	22.223		50.0uM	+		

K562 (IC50 > 50 µM)

Primary screen (50 µM)

plate	well	sample id	PI	pass	conc	inc	✓	⊗
1 (Plate 1)	K9	LEM00241425	21.934		50.0uM	+	✓	⊗
1 (Plate 1)	K10	LEM00241425	18.925		50.0uM	+		
1 (Plate 1)	K11	LEM00241425	18.105		50.0uM	+		

plate	well	sample id	PI	pass	conc	inc	✓	⊗
1 (Plate 1)	K9	LEM00241425	11.821		50.0uM	+	✓	⊗
1 (Plate 1)	K10	LEM00241425	10.051		50.0uM	+		
1 (Plate 1)	K11	LEM00241425	16.911		50.0uM	+		

U2OS (IC50 > 50 μM)

Primary screen (50 μM)

plate	well	sample id	PI	pass	conc	inc	✓	⊗
1 (Plate 1)	K9	LEM00241425	35.028		50.0uM	+	✓	⊗
1 (Plate 1)	K10	LEM00241425	29.418		50.0uM	+		
1 (Plate 1)	K11	LEM00241425	36.114		50.0uM	+		

plate	well	sample id	PI	pass	conc	inc	✓	⊗
1 (Plate 1)	K9	LEM00241425	29.11		50.0uM	+	✓	⊗
1 (Plate 1)	K10	LEM00241425	26.232		50.0uM	+		
1 (Plate 1)	K11	LEM00241425	30.802		50.0uM	+		

LEM 241450 (5cb)

BJ (IC50 > 50 μM)

Primary screen (50 μM)

plate	well	sample id	PI	pass	conc	inc	✓	⊗
1 (Plate 1)	E12	LEM00241450	16.394		50.0uM	+	✓	⊗
1 (Plate 1)	E13	LEM00241450	19.961		50.0uM	+		
1 (Plate 1)	E14	LEM00241450	14.78		50.0uM	+		

plate	well	sample id	PI	pass	conc	inc	✓	⊗
1 (Plate 1)	E12	LEM00241450	15.381		50.0uM	+	✓	⊗
1 (Plate 1)	E13	LEM00241450	11.563		50.0uM	+		
1 (Plate 1)	E14	LEM00241450	12.751		50.0uM	+		

MRC5 (IC50 2.4 μM)

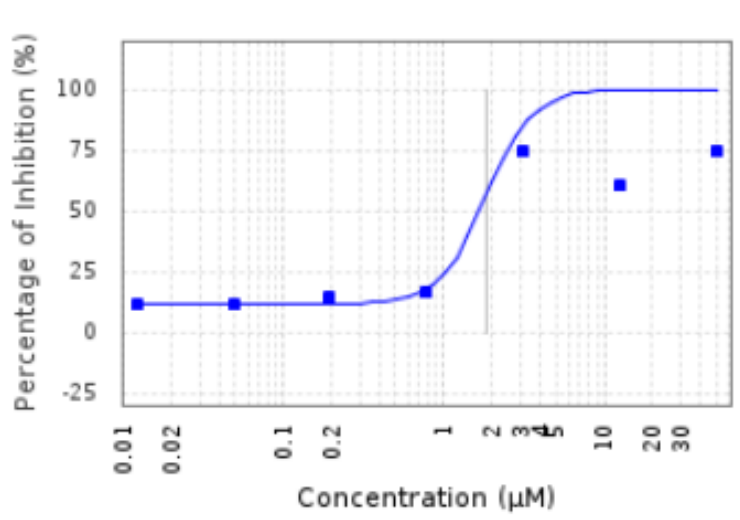

IC50: 1.8221 (0.7129 - 4.6569) pass
slope=3.000 min=11.463 max=100.000 err=2324.289 R^2=0.5843

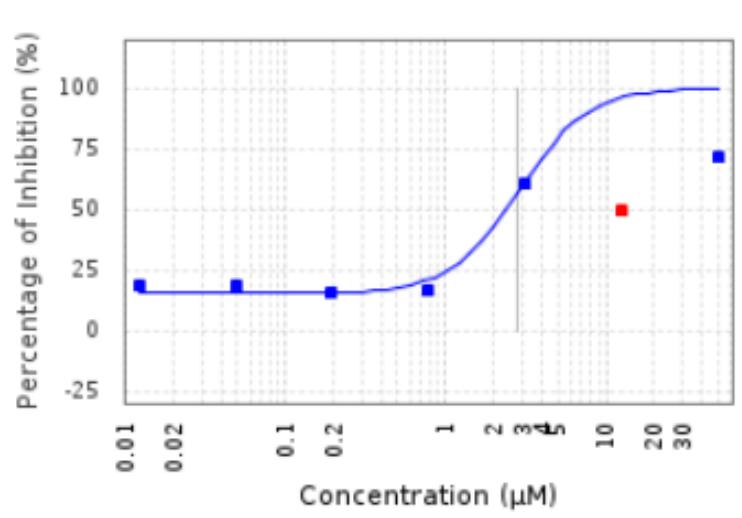

IC50: 2.8499 (1.5472 - 5.2495) pass
slope=2.000 min=15.000 max=100.000 err=849.734 R^2=0.7360

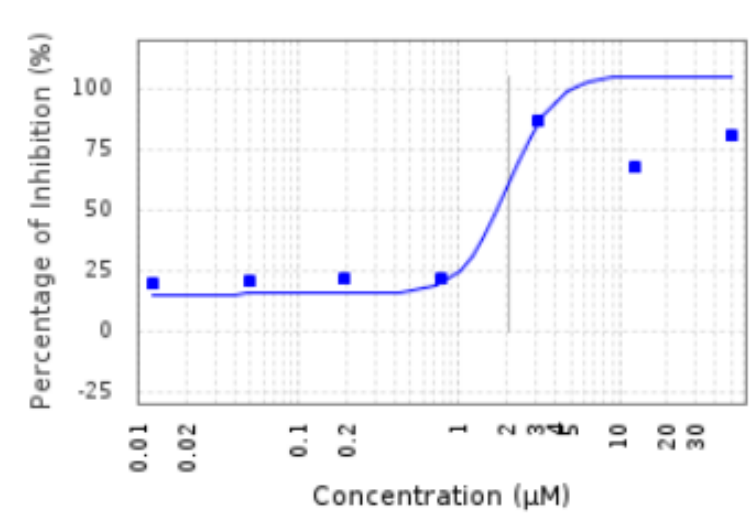

IC50: 2.032 (1.0929 - 3.7779) pass
slope=3.000 min=15.000 max=105.000 err=2080.739 R^2=0.6466

A549 (IC50 2.4 μM)

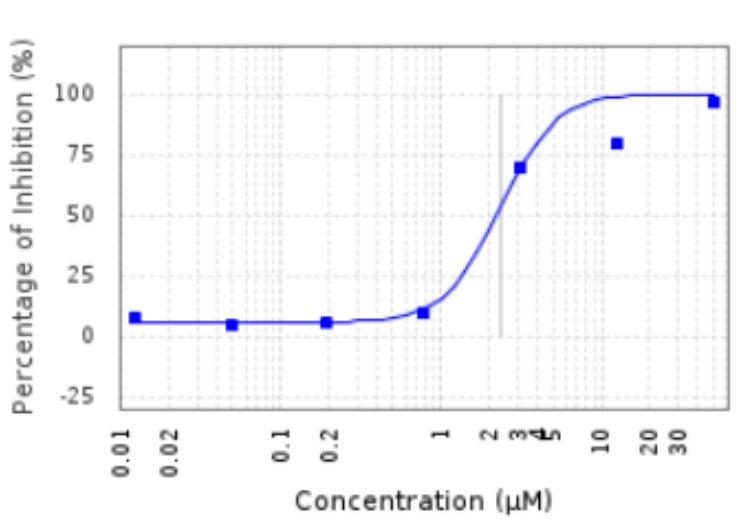

IC50: 2.3242 (1.7461 - 3.0938) pass
slope=2.500 min=5.409 max=100.000 err=385.162 R^2=0.9619

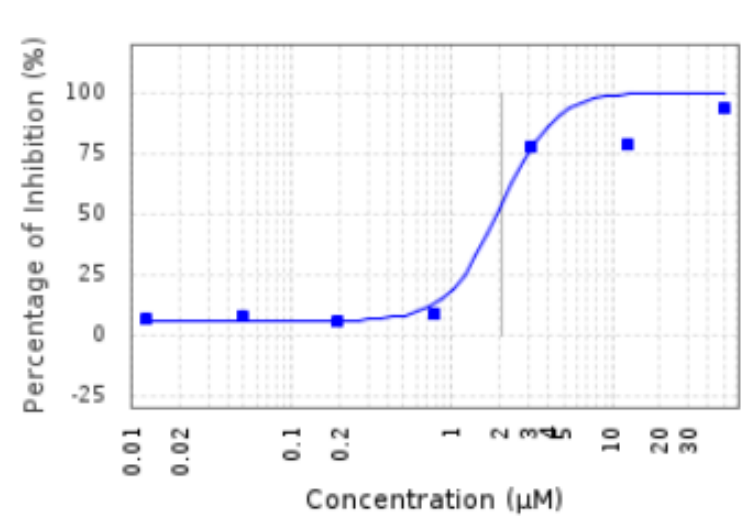

IC50: 2.0184 (1.3174 - 3.0923) pass
slope=2.608 min=5.401 max=100.000 err=477.035 R^2=0.9531

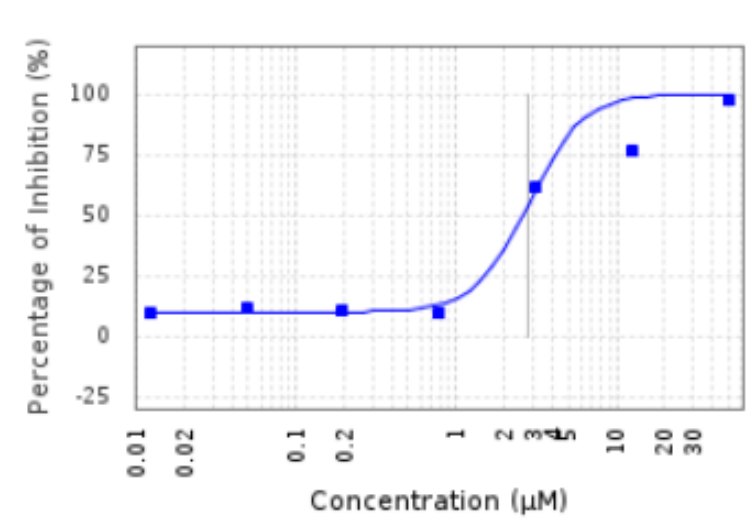

IC50: 2.8409 (2.1314 - 3.7864) pass
slope=2.600 min=9.777 max=100.000 err=488.324 R^2=0.9430

CCRF-CEM (IC50 1.4 µM)

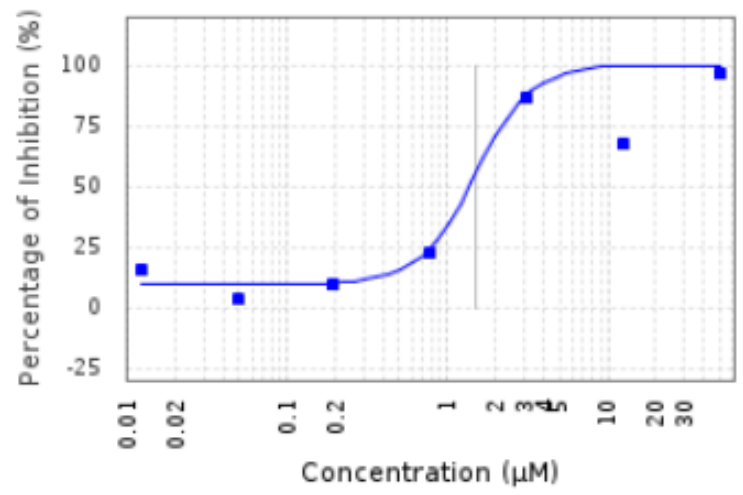

IC50: 1.4954 *(0.7799 - 2.8675)* pass
slope=2.500 min=9.500 max=100.000 err=1114.053 R^2=0.8800

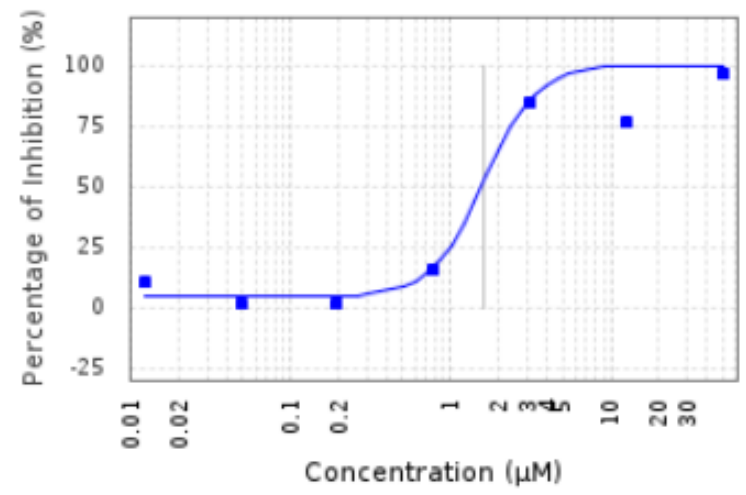

IC50: 1.6109 *(0.9877 - 2.6271)* pass
slope=2.630 min=4.132 max=100.000 err=604.245 R^2=0.9448

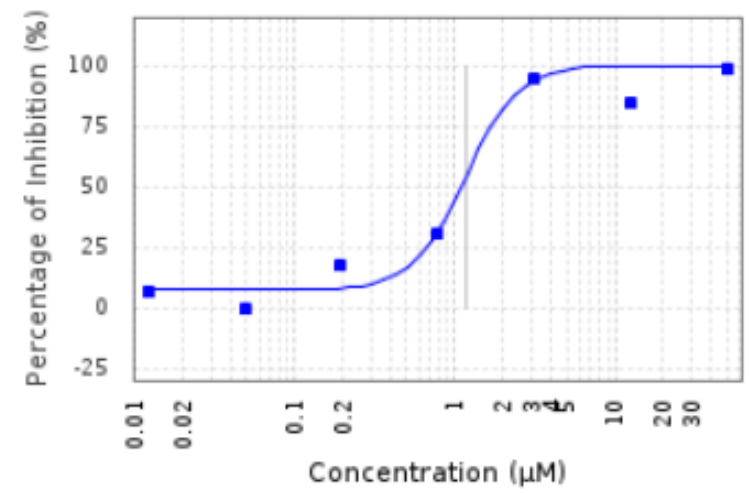

IC50: 1.1572 *(0.7801 - 1.7166)* pass
slope=2.633 min=7.197 max=100.000 err=370.797 R^2=0.9674

HCT116 (IC50 8.3 µM)

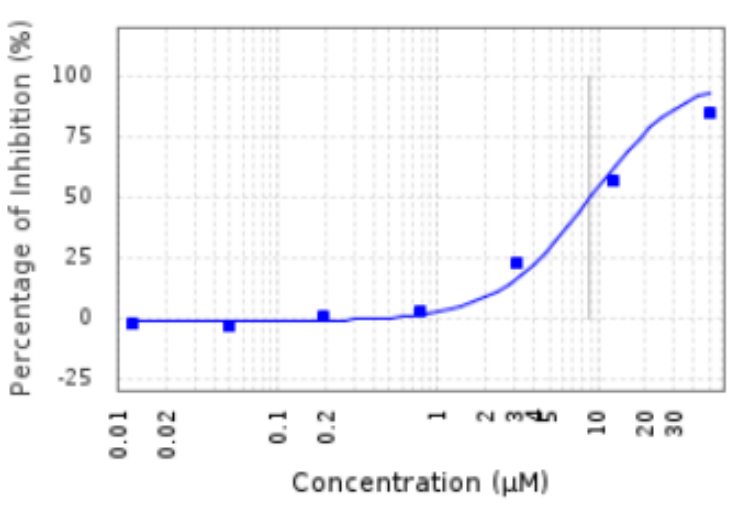

IC50: 8.8786 *(6.1769 - 12.762)* pass
slope=1.500 min=-1.601 max=100.000 err=160.367 R^2=0.9777

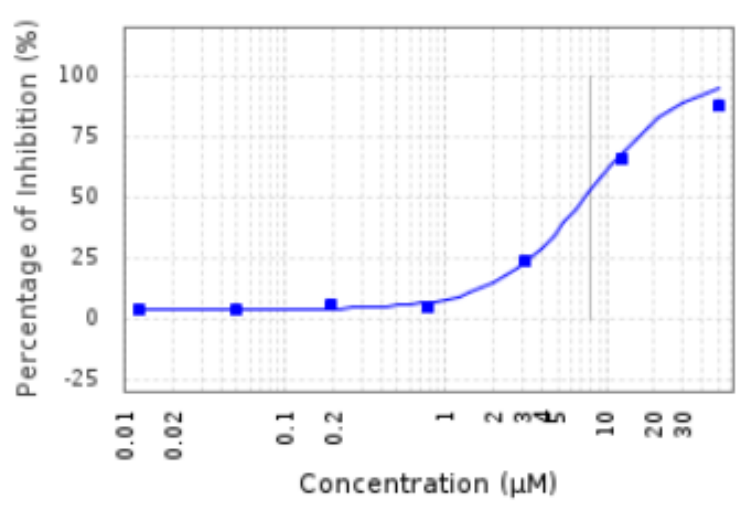

IC50: 7.8835 *(6.2424 - 9.9559)* pass
slope=1.500 min=3.487 max=100.000 err=63.125 R^2=0.9912

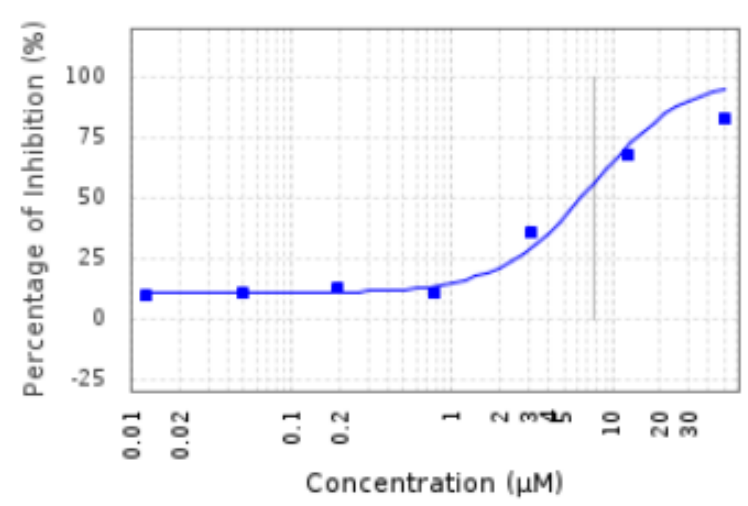

IC50: 7.5927 *(4.8295 - 11.937)* pass
slope=1.500 min=10.387 max=100.000 err=206.826 R^2=0.9637

HCT116p53-/- (IC50 9.0 µM)

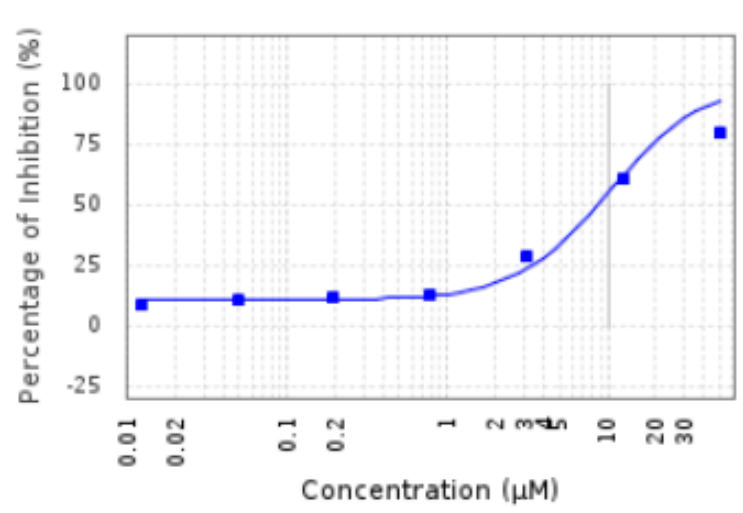

IC50: 10.209 *(6.3198 - 16.491)* pass
slope=1.500 min=10.262 max=100.000 err=193.377 R^2=0.9606

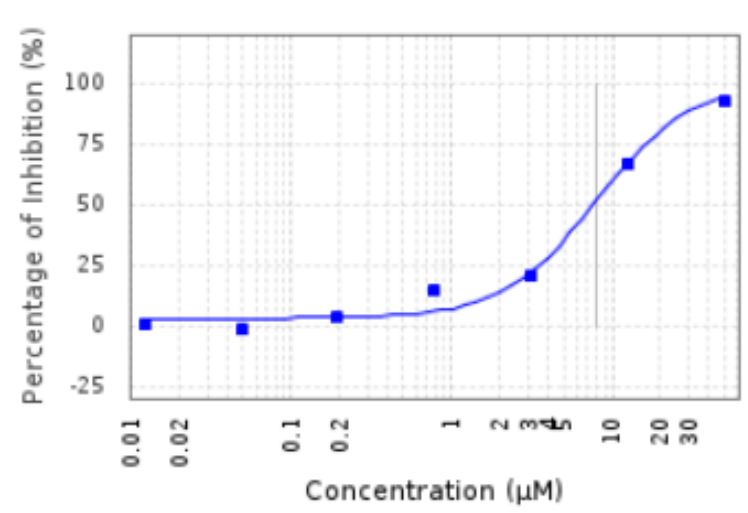

IC50: 8.0296 *(5.9095 - 10.91)* pass
slope=1.500 min=2.845 max=100.000 err=109.677 R^2=0.9866

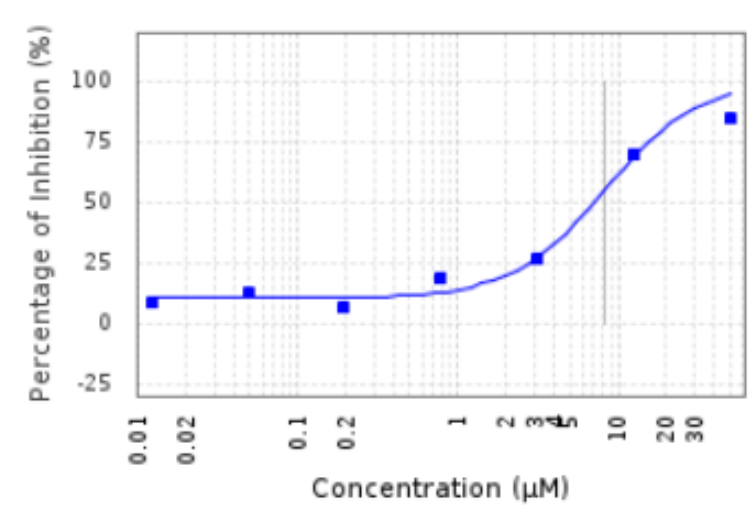

IC50: 8.3097 *(5.5032 - 12.547)* pass
slope=1.500 min=10.116 max=100.000 err=167.383 R^2=0.9722

K562 (IC50 10 µM)

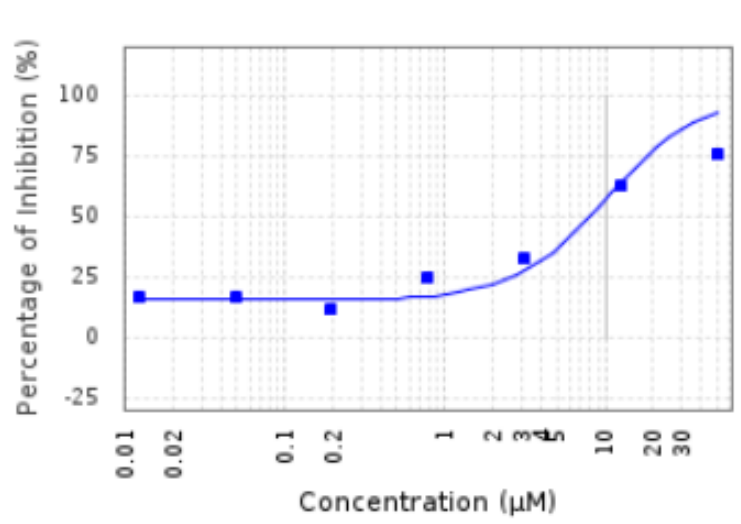

IC50: 10.292 *(4.9507 - 21.394)* pass
slope=1.500 min=15.000 max=100.000 err=399.987 R^2=0.8933

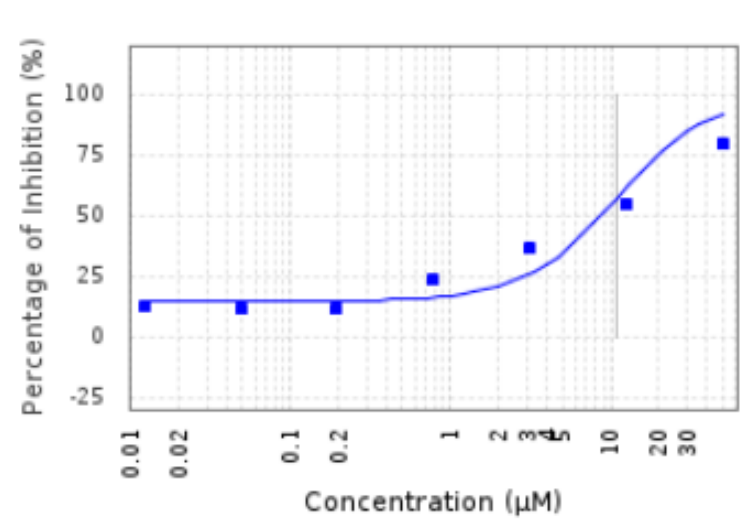

IC50: 11.029 *(5.2878 - 23.003)* pass
slope=1.500 min=14.477 max=100.000 err=368.885 R^2=0.9105

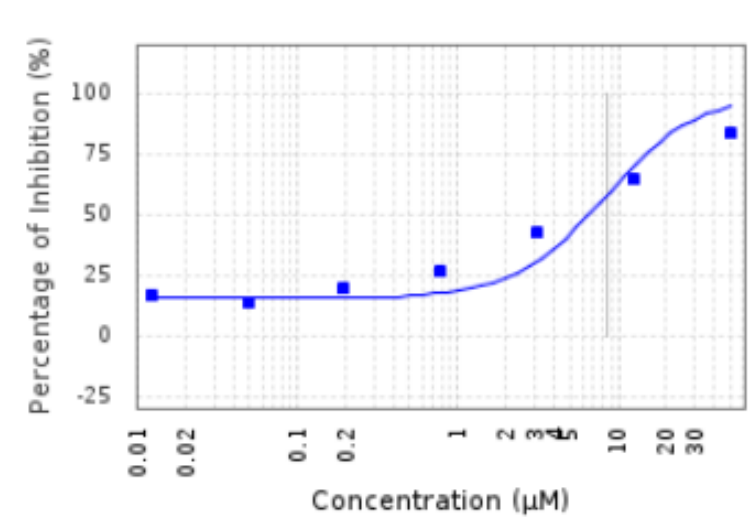

IC50: 8.4882 *(4.3622 - 16.517)* pass
slope=1.500 min=15.000 max=100.000 err=386.972 R^2=0.9097

U2OS (IC50 7.2 μM)

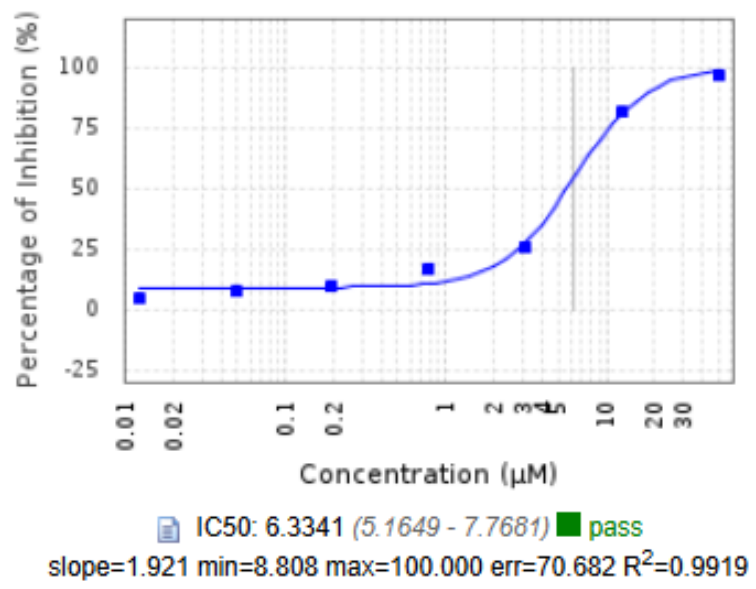

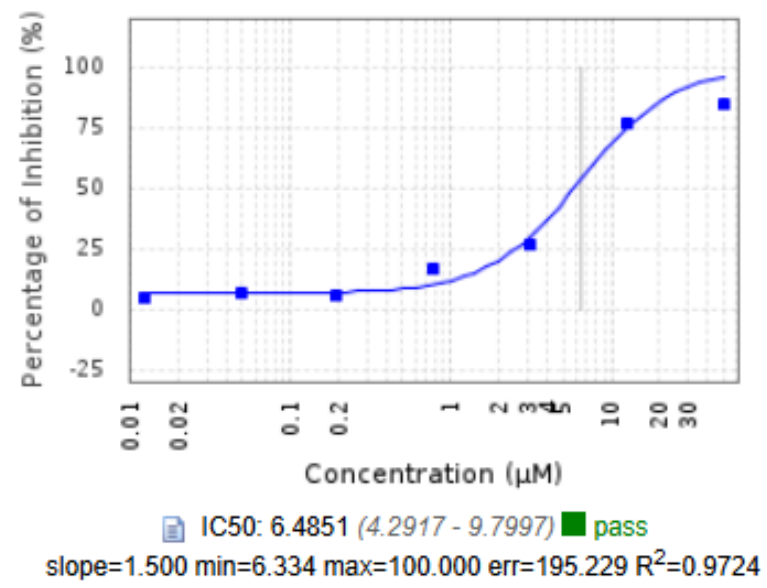

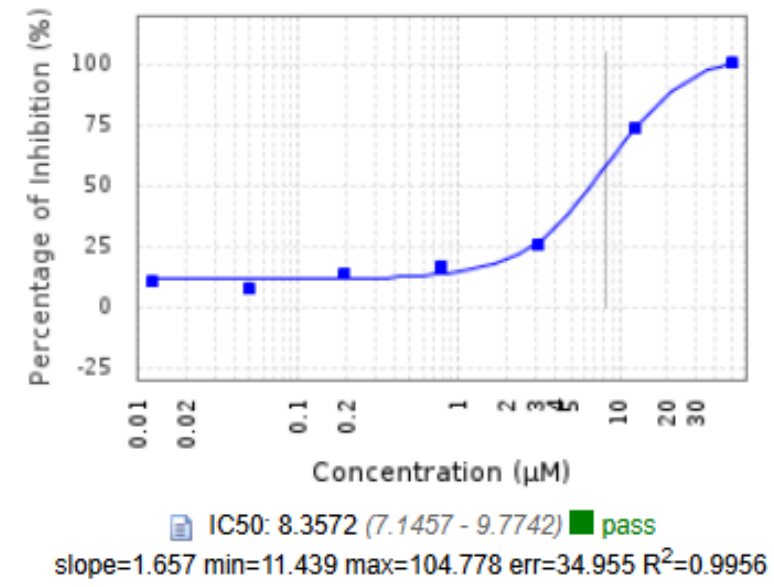

LEM 241427 (5cc)

BJ (IC50 > 50 μM)

Primary screen (50 μM)

plate	well	sample id	PI	pass	conc	inc	✓	⊗
1 (Plate 1)	M9	LEM00241427	-1.2741	●	50.0uM	+	✓	⊗
1 (Plate 1)	M10	LEM00241427	-6.4555	●	50.0uM	+		
1 (Plate 1)	M11	LEM00241427	-4.9266	●	50.0uM	+		

plate	well	sample id	PI	pass	conc	inc	✓	⊗
1 (Plate 1)	M9	LEM00241427	-5.5755	●	50.0uM	+	✓	⊗
1 (Plate 1)	M10	LEM00241427	-2.012	●	50.0uM	+		
1 (Plate 1)	M11	LEM00241427	-4.8119	●	50.0uM	+		

MRC5 (IC50 > 50 μM)

Primary screen (50 μM)

plate	well	sample id	PI	pass	conc	inc	✓	⊗
1 (Plate 1)	M9	LEM00241427	14.078	●	50.0uM	+	✓	⊗
1 (Plate 1)	M10	LEM00241427	16.358	●	50.0uM	+		
1 (Plate 1)	M11	LEM00241427	14.838	●	50.0uM	+		

plate	well	sample id	PI	pass	conc	inc	✓	⊗
1 (Plate 1)	M9	LEM00241427	7.2237	●	50.0uM	+	✓	⊗
1 (Plate 1)	M10	LEM00241427	8.8606	●	50.0uM	+		
1 (Plate 1)	M11	LEM00241427	10.853	●	50.0uM	+		

A549 (IC50 > 50 μM)

Primary screen (50 µM)

plate	well	sample id	PI	pass	conc	inc	✓	⊗
1 (Plate 1)	M9	LEM00241427	7.8991	●	50.0uM	+	✓	⊗
1 (Plate 1)	M10	LEM00241427	5.3482	●	50.0uM	+		
1 (Plate 1)	M11	LEM00241427	4.7277	●	50.0uM	+		

plate	well	sample id	PI	pass	conc	inc	✓	⊗
1 (Plate 1)	M9	LEM00241427	10.115	●	50.0uM	+	✓	⊗
1 (Plate 1)	M10	LEM00241427	10.442	●	50.0uM	+		
1 (Plate 1)	M11	LEM00241427	9.198	●	50.0uM	+		

CCRF-CEM (IC50 > 50 µM)

Primary screen (50 µM)

plate	well	sample id	PI	pass	conc	inc	✓	⊗
1 (Plate 1)	M9	LEM00241427	23.87	●	50.0uM	+	✓	⊗
1 (Plate 1)	M10	LEM00241427	30.591	●	50.0uM	+		
1 (Plate 1)	M11	LEM00241427	21.565	●	50.0uM	+		

plate	well	sample id	PI	pass	conc	inc	✓	⊗
1 (Plate 1)	M9	LEM00241427	33.769	●	50.0uM	+	✓	⊗
1 (Plate 1)	M10	LEM00241427	30.786	●	50.0uM	+		
1 (Plate 1)	M11	LEM00241427	11.891	●	50.0uM	+		

HCT116 (IC50 > 50 µM)

Primary screen (50 µM)

plate	well	sample id	PI	pass	conc	inc	✓	⊗
1 (Plate 1)	M9	LEM00241427	0.5350	●	50.0uM	+	✓	⊗
1 (Plate 1)	M10	LEM00241427	1.8237	●	50.0uM	+		
1 (Plate 1)	M11	LEM00241427	-3.7337	●	50.0uM	+		

plate	well	sample id	PI	pass	conc	inc	✓	⊗
1 (Plate 1)	M9	LEM00241427	0.5074	●	50.0uM	+	✓	⊗
1 (Plate 1)	M10	LEM00241427	-0.1134	●	50.0uM	+		
1 (Plate 1)	M11	LEM00241427	2.1372	●	50.0uM	+		

HCT116p53-/- (IC50 > 50 µM)

Primary screen (50 µM)

plate	well	sample id	PI	pass	conc	inc	✓	⊗
1 (Plate 1)	M9	LEM00241427	-7.2836	●	50.0uM	+	✓	⊗
1 (Plate 1)	M10	LEM00241427	-8.5771	●	50.0uM	+		
1 (Plate 1)	M11	LEM00241427	-13.751	●	50.0uM	+		

plate	well	sample id	PI	pass	conc	inc	✓	⊗
1 (Plate 1)	M9	LEM00241427	-3.4397	●	50.0uM	+	✓	⊗
1 (Plate 1)	M10	LEM00241427	-10.188	●	50.0uM	+		
1 (Plate 1)	M11	LEM00241427	-0.8837	●	50.0uM	+		

K562 (IC50 > 50 µM)

Primary screen (50 µM)

plate	well	sample id	PI	pass	conc	inc	✓	⊗
1 (Plate 1)	M9	LEM00241427	2.9697	●	50.0uM	+	✓	⊗
1 (Plate 1)	M10	LEM00241427	3.2432	●	50.0uM	+		
1 (Plate 1)	M11	LEM00241427	7.7107	●	50.0uM	+		

plate	well	sample id	PI	pass	conc	inc	✓	⊗
1 (Plate 1)	M9	LEM00241427	-9.9771	●	50.0uM	+	✓	⊗
1 (Plate 1)	M10	LEM00241427	-10.641	●	50.0uM	+		
1 (Plate 1)	M11	LEM00241427	-7.764	●	50.0uM	+		

U2OS (IC50 > 50 µM)

Primary screen (50 µM)

plate	well	sample id	PI	pass	conc	inc	✓	⊗
1 (Plate 1)	M9	LEM00241427	17.747	●	50.0uM	+	✓	⊗
1 (Plate 1)	M10	LEM00241427	20.009	●	50.0uM	+		
1 (Plate 1)	M11	LEM00241427	17.204	●	50.0uM	+		

plate	well	sample id	PI	pass	conc	inc	✓	⊗
1 (Plate 1)	M9	LEM00241427	21.577	●	50.0uM	+	✓	⊗
1 (Plate 1)	M10	LEM00241427	15.567	●	50.0uM	+		
1 (Plate 1)	M11	LEM00241427	14.636	●	50.0uM	+		

LEM 241428 (5cd)

BJ (IC50 > 50 µM)

Primary screen (50 µM)

plate	well	sample id	PI	pass	conc	inc	✓	⊗
1 (Plate 1)	N9	LEM00241428	-5.5212	●	50.0uM	+	✓	⊗
1 (Plate 1)	N10	LEM00241428	-3.3976	●	50.0uM	+		
1 (Plate 1)	N11	LEM00241428	-5.6911	●	50.0uM	+		

plate	well	sample id	PI	pass	conc	inc	✓	⊗
1 (Plate 1)	N9	LEM00241428	-3.9635	●	50.0uM	+	✓	⊗
1 (Plate 1)	N10	LEM00241428	-4.7271	●	50.0uM	+		
1 (Plate 1)	N11	LEM00241428	-5.5755	●	50.0uM	+		

MRC5 (IC50 > 50 µM)

Primary screen (50 µM)

plate	well	sample id	PI	pass	conc	inc	✓	⊗
1 (Plate 1)	N9	LEM00241428	15.736	●	50.0uM	+	✓	⊗
1 (Plate 1)	N10	LEM00241428	15.875	●	50.0uM	+		
1 (Plate 1)	N11	LEM00241428	14.561	●	50.0uM	+		

plate	well	sample id	PI	pass	conc	inc	✓	⊗
1 (Plate 1)	N9	LEM00241428	6.939	●	50.0uM	+	✓	⊗
1 (Plate 1)	N10	LEM00241428	6.7967	●	50.0uM	+		
1 (Plate 1)	N11	LEM00241428	7.1525	●	50.0uM	+		

A549 (IC50 > 50 µM)

Primary screen (50 µM)

plate	well	sample id	PI	pass	conc	inc	✓	⊗
1 (Plate 1)	N9	LEM00241428	10.726	●	50.0uM	+	✓	⊗
1 (Plate 1)	N10	LEM00241428	8.9333	●	50.0uM	+		
1 (Plate 1)	N11	LEM00241428	6.9339	●	50.0uM	+		

plate	well	sample id	PI	pass	conc	inc	✓	⊗
1 (Plate 1)	N9	LEM00241428	11.948	●	50.0uM	+	✓	⊗
1 (Plate 1)	N10	LEM00241428	11.555	●	50.0uM	+		
1 (Plate 1)	N11	LEM00241428	10.049	●	50.0uM	+		

CCRF-CEM (IC50 25 µM)

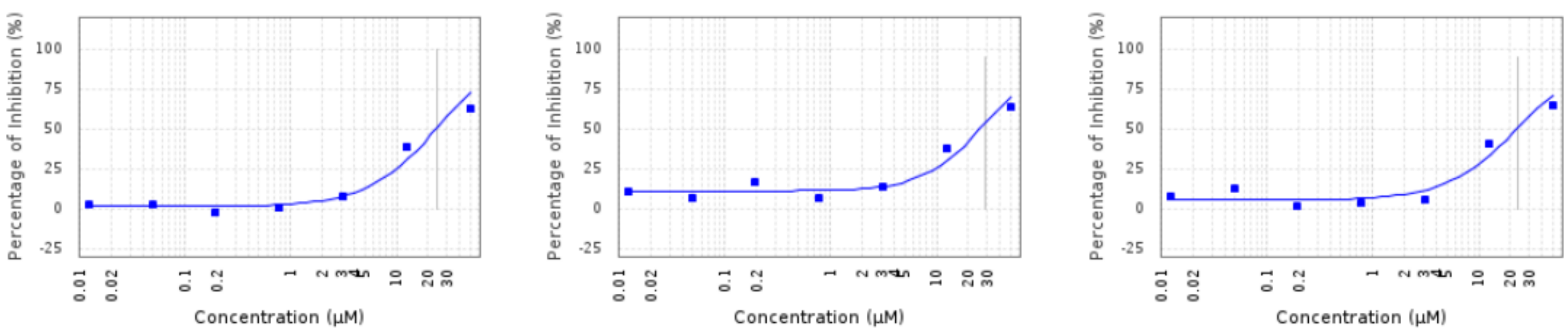

IC50: 24.481 *(13.27 - 45.165)* pass
slope=1.300 min=1.185 max=100.000 err=176.220 R^2=0.9526

IC50: 28.656 *(22.206 - 36.978)* pass
slope=1.500 min=10.812 max=95.000 err=172.715 R^2=0.9351

IC50: 23.261 *(17.465 - 30.98)* pass
slope=1.300 min=5.179 max=95.000 err=211.153 R^2=0.9396

HCT116 (IC50 > 50 µM)

Primary screen (50 µM)

plate	well	sample id	PI	pass	conc	inc	✓	⊗
1 (Plate 1)	N9	LEM00241428	17.852	●	50.0uM	+	✓	⊗
1 (Plate 1)	N10	LEM00241428	20.912	●	50.0uM	+		
1 (Plate 1)	N11	LEM00241428	18.335	●	50.0uM	+		

plate	well	sample id	PI	pass	conc	inc	✓	⊗
1 (Plate 1)	N9	LEM00241428	14.244	●	50.0uM	+	✓	⊗
1 (Plate 1)	N10	LEM00241428	13.546	●	50.0uM	+		
1 (Plate 1)	N11	LEM00241428	13.546	●	50.0uM	+		

HCT116p53-/- (IC50 > 50 µM)

Primary screen (50 µM)

plate	well	sample id	PI	pass	conc	inc	✓	⊗
1 (Plate 1)	N9	LEM00241428	25.808	●	50.0uM	+	✓	⊗
1 (Plate 1)	N10	LEM00241428	19.125	●	50.0uM	+		
1 (Plate 1)	N11	LEM00241428	9.3163	●	50.0uM	+		

plate	well	sample id	PI	pass	conc	inc	✓	⊗
1 (Plate 1)	N9	LEM00241428	18.031	●	50.0uM	+	✓	⊗
1 (Plate 1)	N10	LEM00241428	14.248	●	50.0uM	+		
1 (Plate 1)	N11	LEM00241428	17.827	●	50.0uM	+		

K562 (IC50 > 50 µM)

Primary screen (50 µM)

plate	well	sample id	PI	pass	conc	inc	✓	⊗
1 (Plate 1)	N9	LEM00241428	-0.1302	●	50.0uM	+	✓	⊗
1 (Plate 1)	N10	LEM00241428	-1.1332	●	50.0uM	+		
1 (Plate 1)	N11	LEM00241428	-4.1419	●	50.0uM	+		

plate	well	sample id	PI	pass	conc	inc	✓	⊗
1 (Plate 1)	N9	LEM00241428	-16.616	●	50.0uM	+	✓	⊗
1 (Plate 1)	N10	LEM00241428	-14.293	●	50.0uM	+		
1 (Plate 1)	N11	LEM00241428	-15.399	●	50.0uM	+		

U2OS (IC50 > 50 μM)

Primary screen (50 μM)

plate	well	sample id	PI	pass	conc	inc	✓	⊗
1 (Plate 1)	N9	LEM00241428	28.785	●	50.0uM	+	✓	⊗
1 (Plate 1)	N10	LEM00241428	22.904	●	50.0uM	+		
1 (Plate 1)	N11	LEM00241428	26.433	●	50.0uM	+		

plate	well	sample id	PI	pass	conc	inc	✓	⊗
1 (Plate 1)	N9	LEM00241428	25.555	●	50.0uM	+	✓	⊗
1 (Plate 1)	N10	LEM00241428	25.131	●	50.0uM	+		
1 (Plate 1)	N11	LEM00241428	27.247	●	50.0uM	+		

LEM 241426 (5da)

BJ (IC50 > 50 μM)

Primary screen (50 μM)

plate	well	sample id	PI	pass	conc	inc	✓	⊗
1 (Plate 1)	L9	LEM00241426	5.0965	●	50.0uM	+	✓	⊗
1 (Plate 1)	L10	LEM00241426	3.9922	●	50.0uM	+		
1 (Plate 1)	L11	LEM00241426	6.7104	●	50.0uM	+		

plate	well	sample id	PI	pass	conc	inc	✓	⊗
1 (Plate 1)	L9	LEM00241426	2.7393	●	50.0uM	+	✓	⊗
1 (Plate 1)	L10	LEM00241426	3.5877	●	50.0uM	+		
1 (Plate 1)	L11	LEM00241426	6.3028	●	50.0uM	+		

MRC5 (IC50 > 50 μM)

Primary screen (50 μM)

plate	well	sample id	PI	pass	conc	inc	✓	⊗
1 (Plate 1)	L9	LEM00241426	22.648	●	50.0uM	+	✓	⊗
1 (Plate 1)	L10	LEM00241426	23.823	●	50.0uM	+		
1 (Plate 1)	L11	LEM00241426	23.063	●	50.0uM	+		

plate	well	sample id	PI	pass	conc	inc	✓	⊗
1 (Plate 1)	L9	LEM00241426	17.614	●	50.0uM	+	✓	⊗
1 (Plate 1)	L10	LEM00241426	20.39	●	50.0uM	+		
1 (Plate 1)	L11	LEM00241426	18.682	●	50.0uM	+		

A549 (IC50 > 50 μM)

Primary screen (50 μM)

plate	well	sample id	PI	pass	conc	inc	✓	⊗
1 (Plate 1)	L9	LEM00241426	13.553	●	50.0uM	+	✓	⊗
1 (Plate 1)	L10	LEM00241426	10.588	●	50.0uM	+		
1 (Plate 1)	L11	LEM00241426	9.6917	●	50.0uM	+		

plate	well	sample id	PI	pass	conc	inc	✓	⊗
1 (Plate 1)	L9	LEM00241426	11.358	●	50.0uM	+	✓	⊗
1 (Plate 1)	L10	LEM00241426	12.406	●	50.0uM	+		
1 (Plate 1)	L11	LEM00241426	12.93	●	50.0uM	+		

CCRF-CEM (IC50 > 50 μM)

Primary screen (50 μM)

plate	well	sample id	PI	pass	conc	inc	✓	⊗
1 (Plate 1)	L10	LEM00241426	43.843	●	50.0uM	+		
1 (Plate 1)	L11	LEM00241426	25.598	●	50.0uM	+		
1 (Plate 1)	M9	LEM00241427	23.87	●	50.0uM	+	✓	⊗

plate	well	sample id	PI	pass	conc	inc	✓	⊗
1 (Plate 1)	L9	LEM00241426	44.907	●	50.0uM	+	✓	⊗
1 (Plate 1)	L10	LEM00241426	35.559	●	50.0uM	+		
1 (Plate 1)	L11	LEM00241426	30.189	●	50.0uM	+		

HCT116 (IC50 > 50 μM)

Primary screen (50 μM)

plate	well	sample id	PI	pass	conc	inc	✓	⊗
1 (Plate 1)	L9	LEM00241426	22.684	●	50.0uM	+	✓	⊗
1 (Plate 1)	L10	LEM00241426	20.671	●	50.0uM	+		
1 (Plate 1)	L11	LEM00241426	19.382	●	50.0uM	+		

plate	well	sample id	PI	pass	conc	inc	✓	⊗
1 (Plate 1)	L9	LEM00241426	17.581	●	50.0uM	+	✓	⊗
1 (Plate 1)	L10	LEM00241426	24.721	●	50.0uM	+		
1 (Plate 1)	L11	LEM00241426	19.133	●	50.0uM	+		

HCT116p53-/- (IC50 > 50 μM)

Primary screen (50 μM)

plate	well	sample id	PI	pass	conc	inc	✓	⊗
1 (Plate 1)	L9	LEM00241426	31.737	●	50.0uM	+	✓	⊗
1 (Plate 1)	L10	LEM00241426	28.072	●	50.0uM	+		
1 (Plate 1)	L11	LEM00241426	22.898	●	50.0uM	+		

plate	well	sample id	PI	pass	conc	inc	✓	⊗
1 (Plate 1)	L9	LEM00241426	21.814	●	50.0uM	+	✓	⊗
1 (Plate 1)	L10	LEM00241426	20.587	●	50.0uM	+		
1 (Plate 1)	L11	LEM00241426	8.8293	●	50.0uM	+		

K562 (IC50 > 50 μM)

Primary screen (50 μM)

plate	well	sample id	PI	pass	conc	inc	✓	⊗
1 (Plate 1)	L9	LEM00241426	20.019	●	50.0uM	+	✓	⊗
1 (Plate 1)	L10	LEM00241426	20.384	●	50.0uM	+		
1 (Plate 1)	L11	LEM00241426	19.198	●	50.0uM	+		

plate	well	sample id	PI	pass	conc	inc	✓	⊗
1 (Plate 1)	L9	LEM00241426	8.5017	●	50.0uM	+	✓	⊗
1 (Plate 1)	L10	LEM00241426	7.3952	●	50.0uM	+		
1 (Plate 1)	L11	LEM00241426	7.8378	●	50.0uM	+		

U2OS (IC50 > 50 μM)

Primary screen (50 μM)

plate	well	sample id	PI	pass	conc	inc	✓	⊗
1 (Plate 1)	L9	LEM00241426	35.299	●	50.0uM	+	✓	⊗
1 (Plate 1)	L10	LEM00241426	33.309	●	50.0uM	+		
1 (Plate 1)	L11	LEM00241426	40.276	●	50.0uM	+		

plate	well	sample id	PI	pass	conc	inc	✓	⊗
1 (Plate 1)	M9	LEM00241427	21.577	●	50.0uM	+	✓	⊗
1 (Plate 1)	M10	LEM00241427	15.567	●	50.0uM	+		
1 (Plate 1)	M11	LEM00241427	14.636	●	50.0uM	+		

LEM 241451 (5db)

BJ (IC50 > 50 μM)

Primary screen (50 μM)

plate	well	sample id	PI	pass	conc	inc	✓	⊗
1 (Plate 1)	F12	LEM00241451	8.8339	●	50.0uM	+	✓	⊗
1 (Plate 1)	F13	LEM00241451	9.0038	●	50.0uM	+		
1 (Plate 1)	F14	LEM00241451	11.127	●	50.0uM	+		

plate	well	sample id	PI	pass	conc	inc	✓	⊗
1 (Plate 1)	F12	LEM00241451	10.46	●	50.0uM	+	✓	⊗
1 (Plate 1)	F13	LEM00241451	8.3391	●	50.0uM	+		
1 (Plate 1)	F14	LEM00241451	4.012	●	50.0uM	+		

MRC5 (IC50 > 50 μM)

Primary screen (50 µM)

plate	well	sample id	PI	pass	conc	inc		
1 (Plate 1)	F12	LEM00241451	26.657		50.0uM	+		
1 (Plate 1)	F13	LEM00241451	22.234		50.0uM	+		
1 (Plate 1)	F14	LEM00241451	28.385		50.0uM	+		

plate	well	sample id	PI	pass	conc	inc		
1 (Plate 1)	F12	LEM00241451	23.735		50.0uM	+		
1 (Plate 1)	F13	LEM00241451	24.375		50.0uM	+		
1 (Plate 1)	F14	LEM00241451	22.667		50.0uM	+		

A549 (IC50 > 50 µM)

Primary screen (50 µM)

plate	well	sample id	PI	pass	conc	inc		
1 (Plate 1)	F12	LEM00241451	26.79		50.0uM	+		
1 (Plate 1)	F13	LEM00241451	19.482		50.0uM	+		
1 (Plate 1)	F14	LEM00241451	19.344		50.0uM	+		

plate	well	sample id	PI	pass	conc	inc		
1 (Plate 1)	F12	LEM00241451	16.923		50.0uM	+		
1 (Plate 1)	F13	LEM00241451	19.934		50.0uM	+		
1 (Plate 1)	F14	LEM00241451	17.578		50.0uM	+		

CCRF-CEM (IC50 2.33 µM)

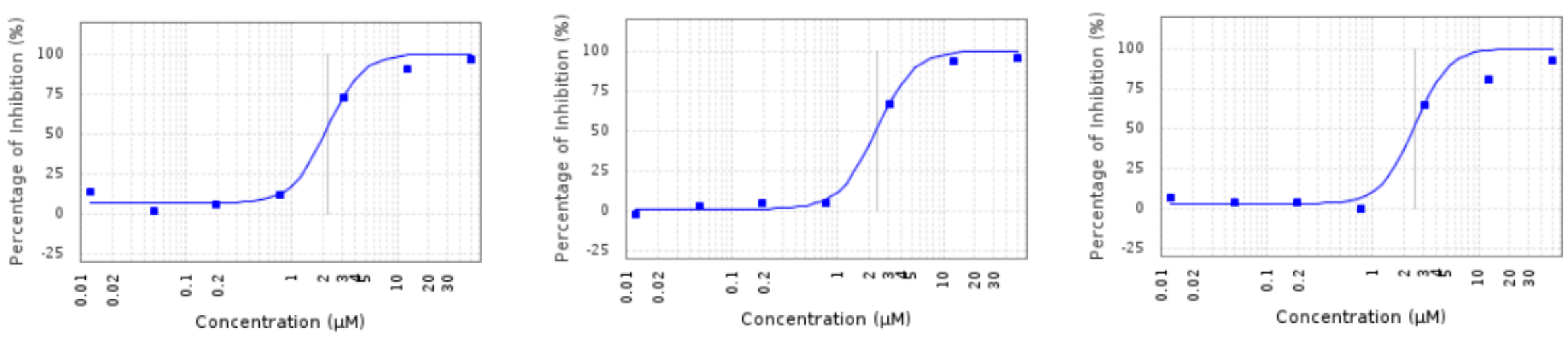

IC50: 2.1653 (1.6941 - 2.7677) pass
slope=2.580 min=6.225 max=100.000 err=164.697 R^2=0.9850

IC50: 2.3242 (1.9924 - 2.7112) pass
slope=2.463 min=0.590 max=100.000 err=81.873 R^2=0.9933

IC50: 2.484 (1.7258 - 3.5754) pass
slope=2.717 min=2.494 max=100.000 err=463.431 R^2=0.9549

HCT116 (IC50 > 50 µM)

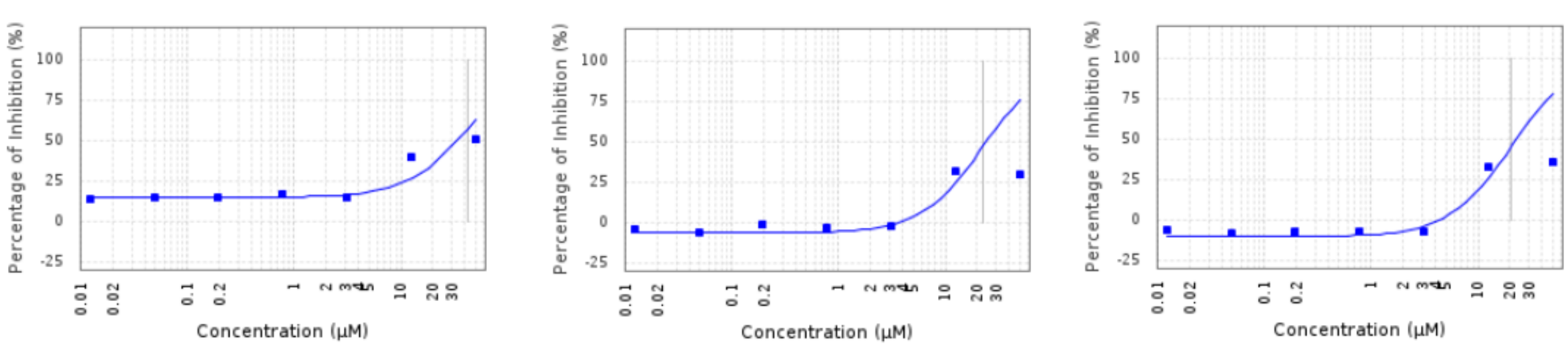

IC50: >50 pass
slope=1.500 min=14.554 max=100.000 err=318.994 R^2=0.7710

IC50: >50 pass
slope=1.500 min=-6.946 max=100.000 err=2213.811 R^2=-0.3430

IC50: >50 pass
slope=1.500 min=-10.582 max=100.000 err=1809.267 R^2=0.2639

HCT116p53-/- (IC50 > 50 µM)

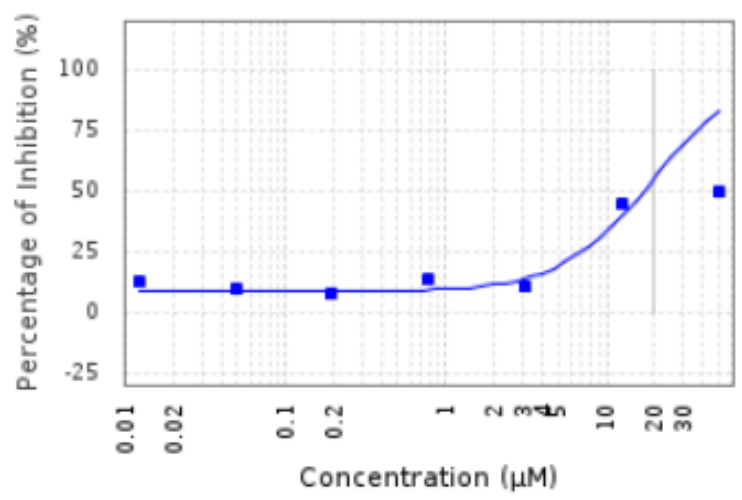

IC50: >50 pass
slope=1.500 min=8.182 max=100.000 err=1143.563 R^2=0.3924

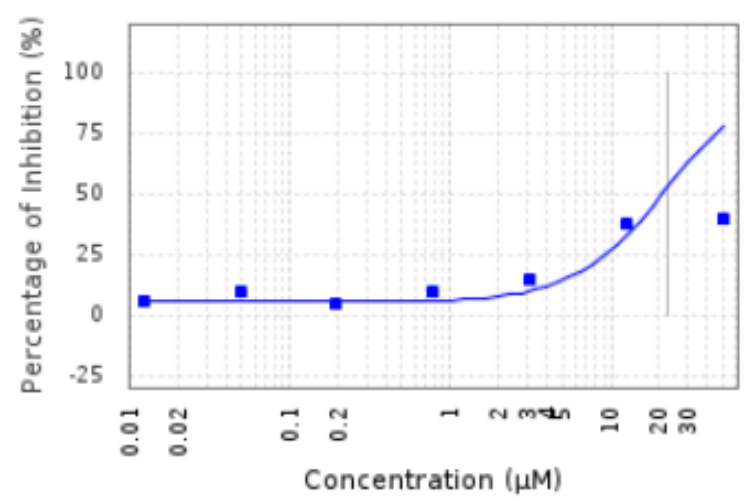

IC50: >50 pass
slope=1.500 min=5.059 max=100.000 err=1497.166 R^2=-0.1188

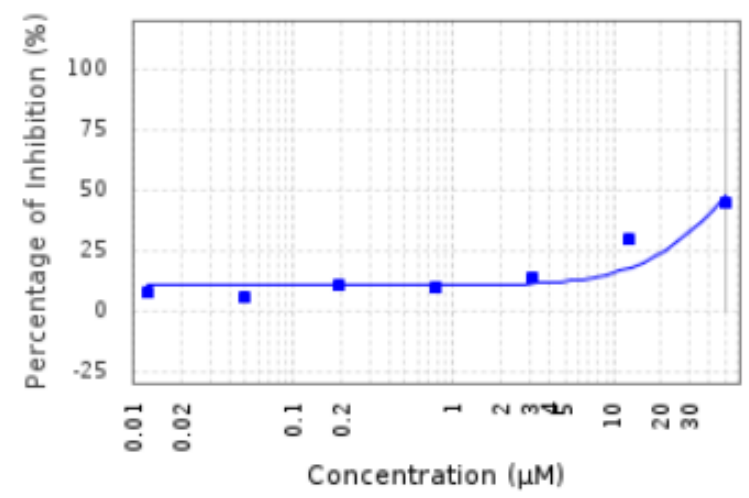

IC50: >50 pass
slope=1.500 min=10.036 max=100.000 err=185.647 R^2=0.8479

K562 (IC50 > 50 µM)

Primary screen (50 µM)

plate	well	sample id	PI	pass	conc	inc		
1 (Plate 1)	F12	LEM00241451	29.775		50.0uM	+	✓	⊗
1 (Plate 1)	F13	LEM00241451	35.154		50.0uM	+		
1 (Plate 1)	F14	LEM00241451	27.04		50.0uM	+		

plate	well	sample id	PI	pass	conc	inc		
1 (Plate 1)	F12	LEM00241451	11.268		50.0uM	+	✓	⊗
1 (Plate 1)	F13	LEM00241451	18.903		50.0uM	+		
1 (Plate 1)	F14	LEM00241451	20.231		50.0uM	+		

U2OS (IC50 > 50 µM)

Primary screen (50 µM)

plate	well	sample id	PI	pass	conc	inc		
1 (Plate 1)	F12	LEM00241451	47.514		50.0uM	+	✓	⊗
1 (Plate 1)	F13	LEM00241451	47.876		50.0uM	+		
1 (Plate 1)	F14	LEM00241451	46.338		50.0uM	+		

plate	well	sample id	PI	pass	conc	inc		
1 (Plate 1)	F12	LEM00241451	44.344		50.0uM	+	✓	⊗
1 (Plate 1)	F13	LEM00241451	49.761		50.0uM	+		
1 (Plate 1)	F14	LEM00241451	47.476		50.0uM	+		

LEM 241429 (5dc)

BJ (IC50 28 µM)

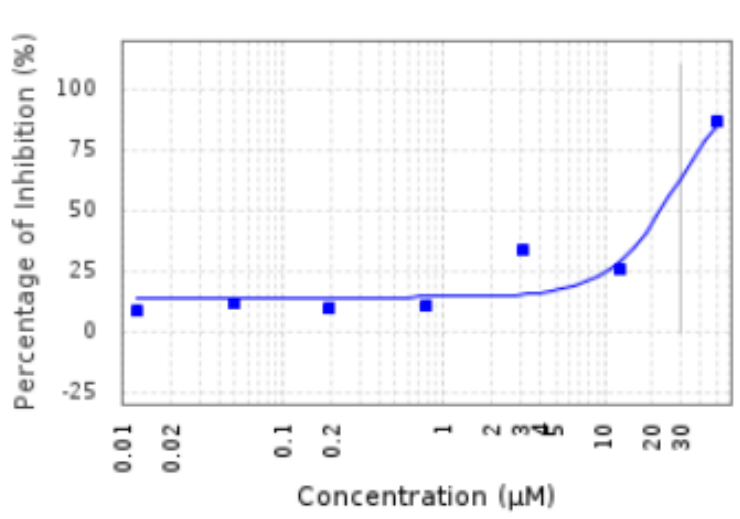

IC50: 29.411 *(0.0080 - 108296)* pass
slope=1.966 min=13.946 max=110.000 err=415.695 R^2=0.9119

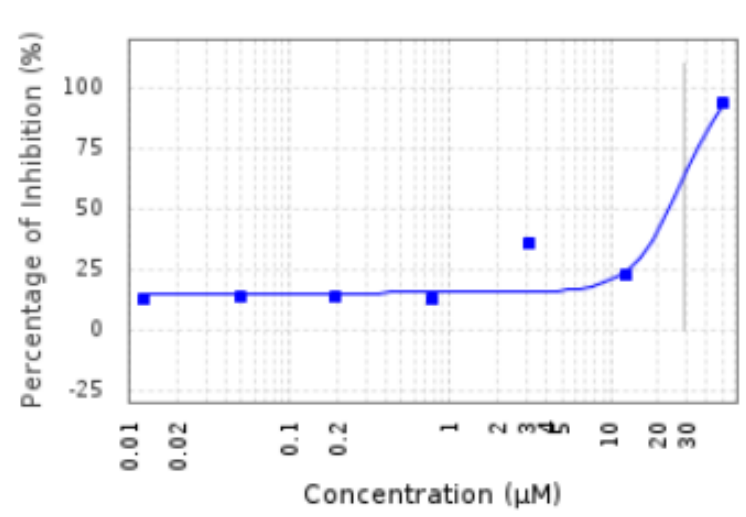

IC50: 28.853 *(4.77086E-9 - 1.74494E11)* pass
slope=2.682 min=15.000 max=110.000 err=446.006 R^2=0.9149

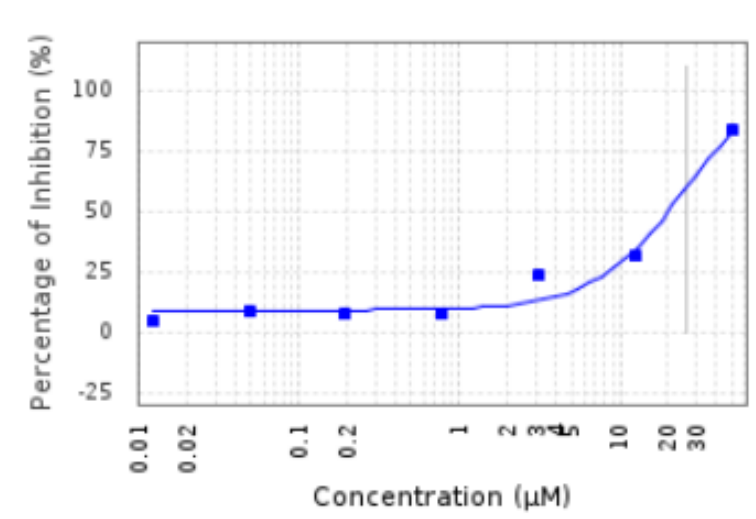

IC50: 26.052 *(3.1649 - 214.44)* pass
slope=1.500 min=8.876 max=110.000 err=135.997 R^2=0.9714

MRC5 (IC50 33 µM)

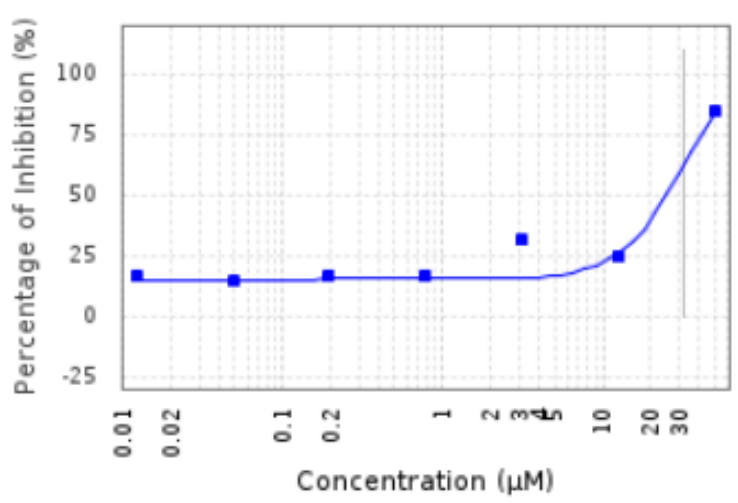

IC50: 32.403 *(0.0002 - 5.85693E6)* pass
slope=2.170 min=15.000 max=110.000 err=259.903 R^2=0.9311

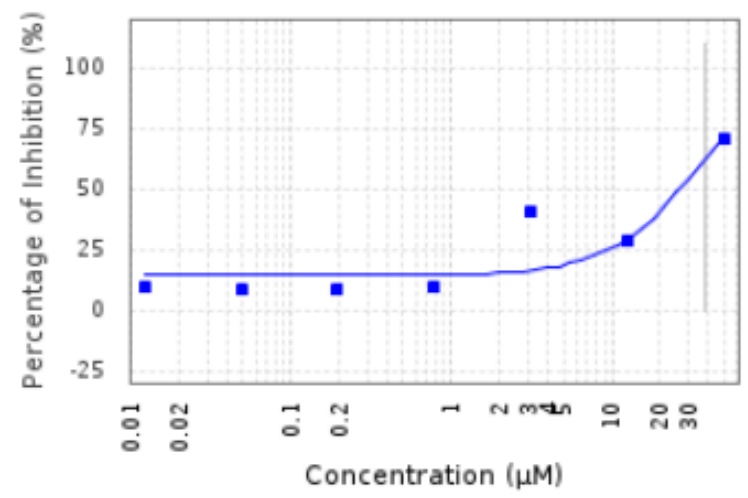

IC50: 37.812 *(0.0001 - 1.35671E7)* pass
slope=1.500 min=14.027 max=109.917 err=708.044 R^2=0.7900

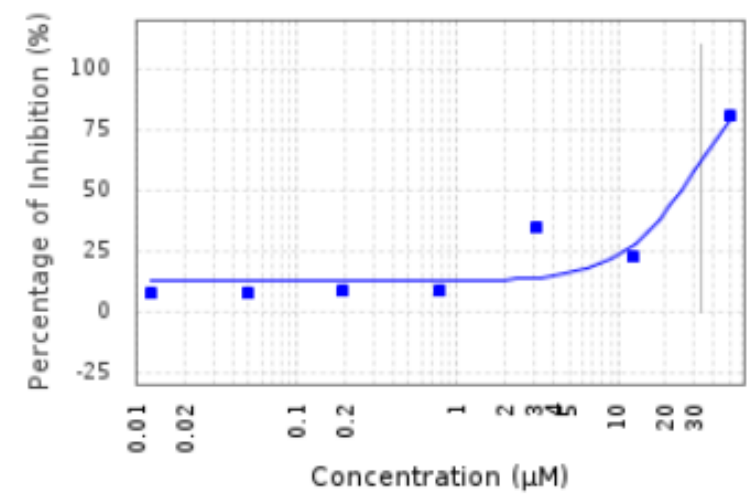

IC50: 32.892 *(0.0010 - 1.05529E6)* pass
slope=1.783 min=12.234 max=110.000 err=535.889 R^2=0.8748

A549 (IC50 8.0 µM)

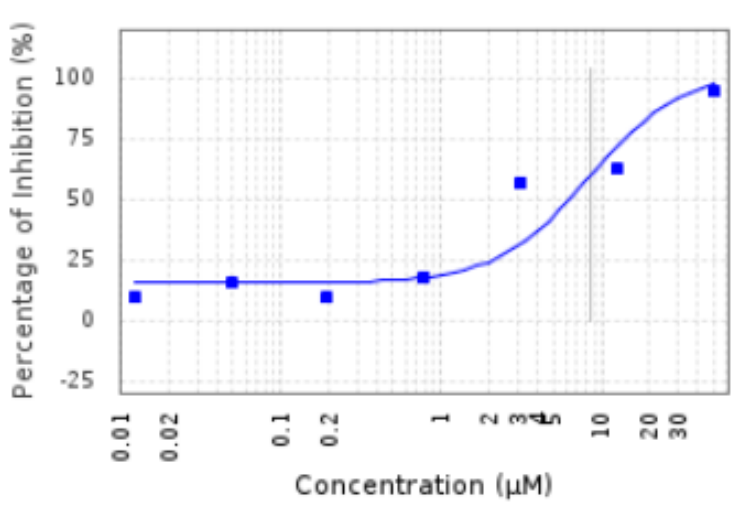

IC50: 8.4629 *(3.3368 - 21.464)* pass
slope=1.500 min=15.000 max=103.056 err=812.842 R^2=0.8792

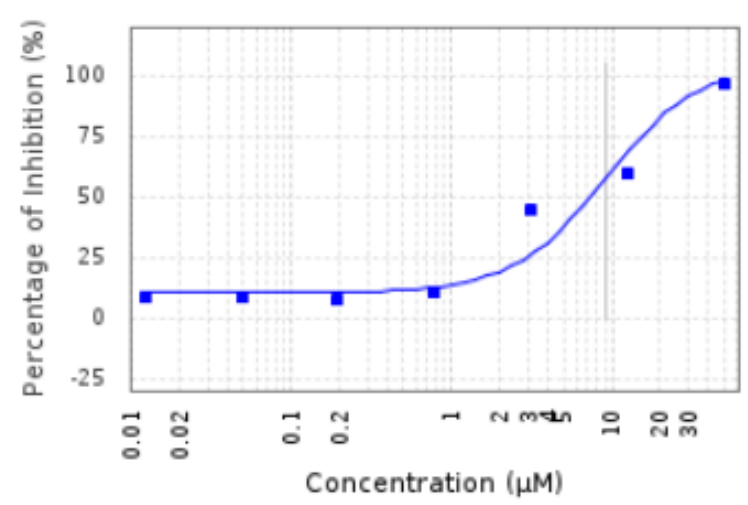

IC50: 9.155 *(4.7912 - 17.493)* pass
slope=1.500 min=10.229 max=104.845 err=434.237 R^2=0.9398

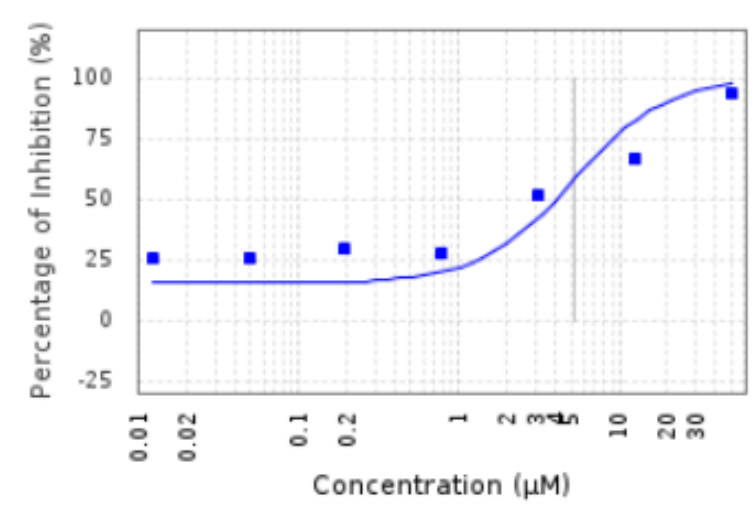

IC50: 5.1609 *(2.116 - 12.587)* pass
slope=1.500 min=15.000 max=100.000 err=804.204 R^2=0.8068

CCRF-CEM (IC50 2.0 µM)

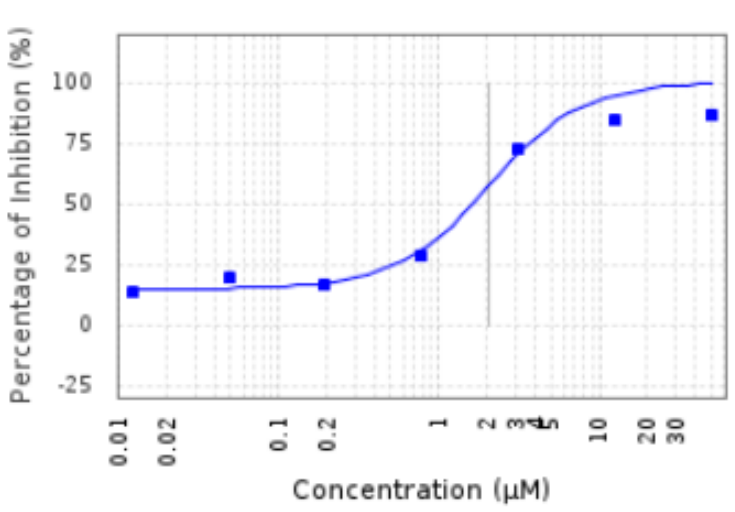

IC50: 2.0474 *(1.3065 - 3.2085)* pass
slope=1.500 min=14.677 max=100.000 err=294.479 R^2=0.9570

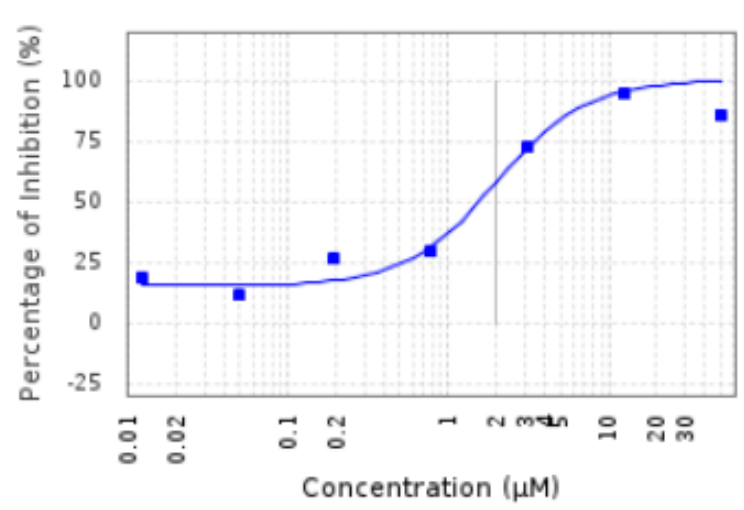

IC50: 1.9706 *(1.2673 - 3.0643)* pass
slope=1.541 min=15.000 max=100.000 err=294.035 R^2=0.9595

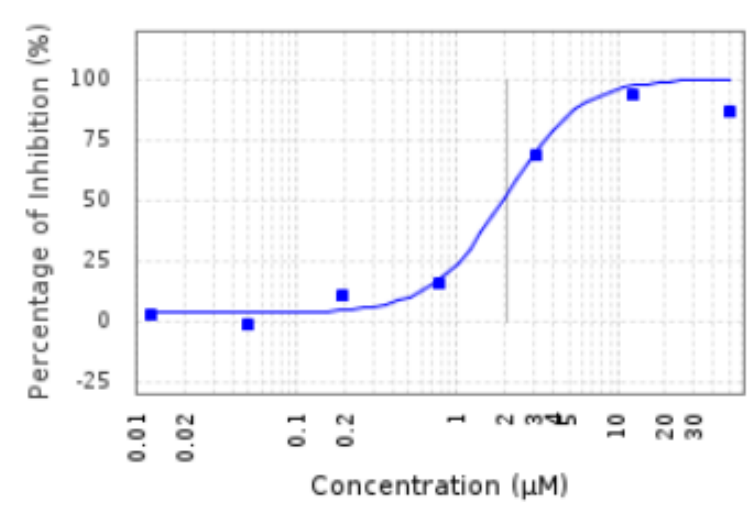

IC50: 2.0148 *(1.4645 - 2.7718)* pass
slope=1.882 min=3.120 max=100.000 err=262.414 R^2=0.9747

HCT116 (IC50 2.9 µM)

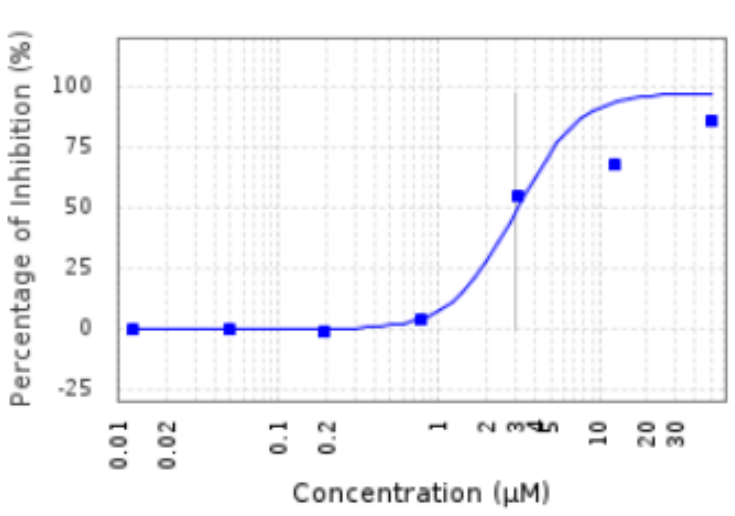

IC50: 3.0146 *(2.1447 - 4.2373)* pass
slope=2.200 min=-0.858 max=97.000 err=804.364 R^2=0.9070

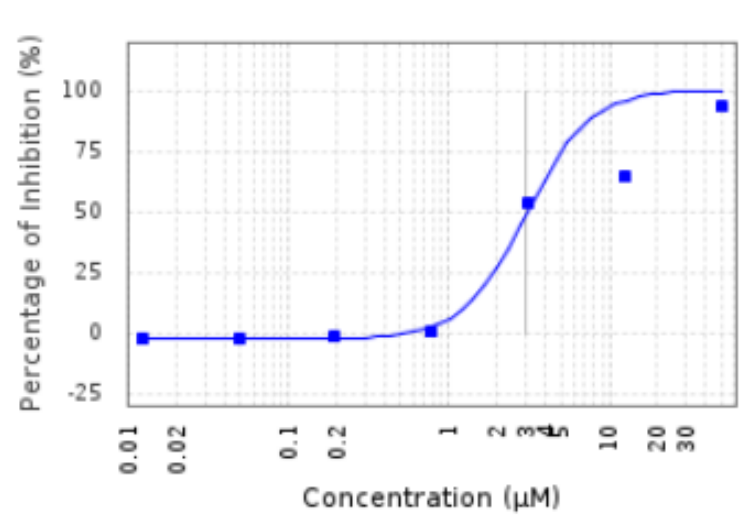

IC50: 3.0283 *(1.9702 - 4.6547)* pass
slope=2.200 min=-2.679 max=100.000 err=1019.345 R^2=0.8949

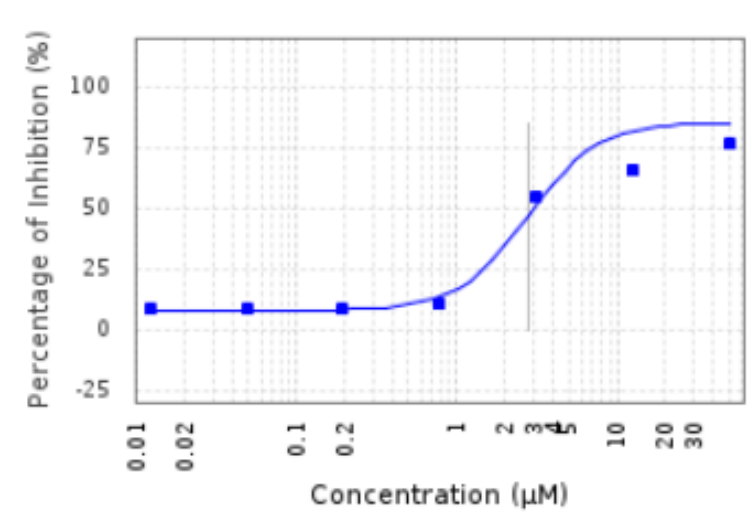

IC50: 2.7584 *(2.0208 - 3.7651)* pass
slope=2.000 min=7.621 max=85.000 err=348.133 R^2=0.9392

HCT116p53-/- (IC50 2.5 µM)

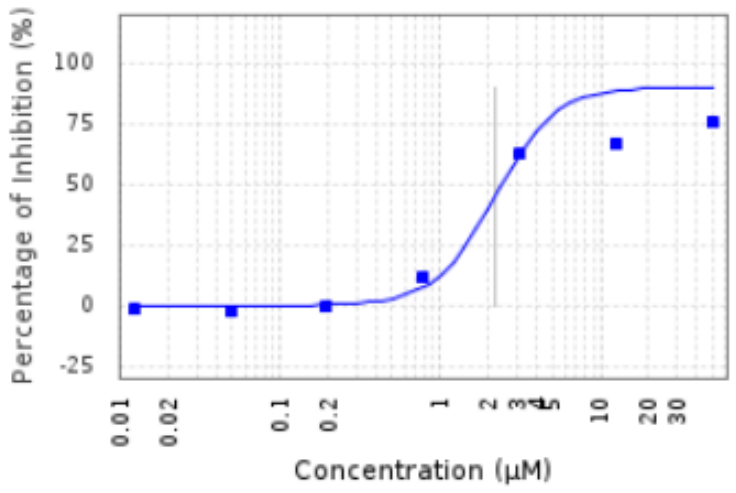

IC50: 2.2014 *(1.5262 - 3.1753)* pass
slope=2.300 min=-0.267 max=90.000 err=684.383 R^2=0.9123

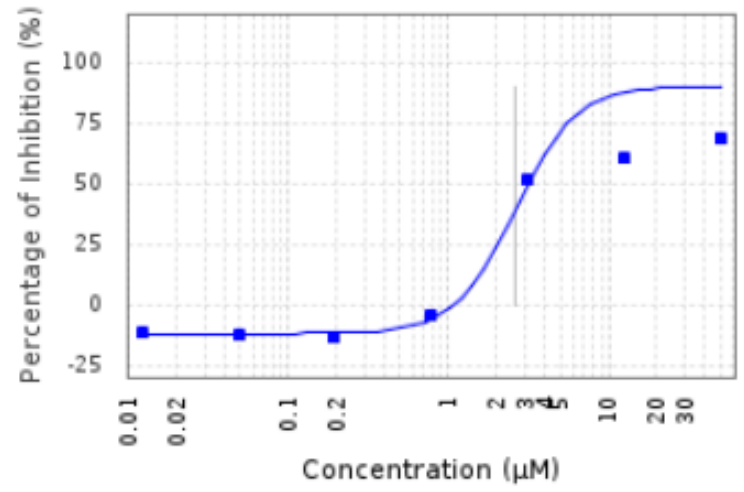

IC50: 2.6181 *(1.7684 - 3.876)* pass
slope=2.300 min=-12.103 max=90.000 err=1193.726 R^2=0.8645

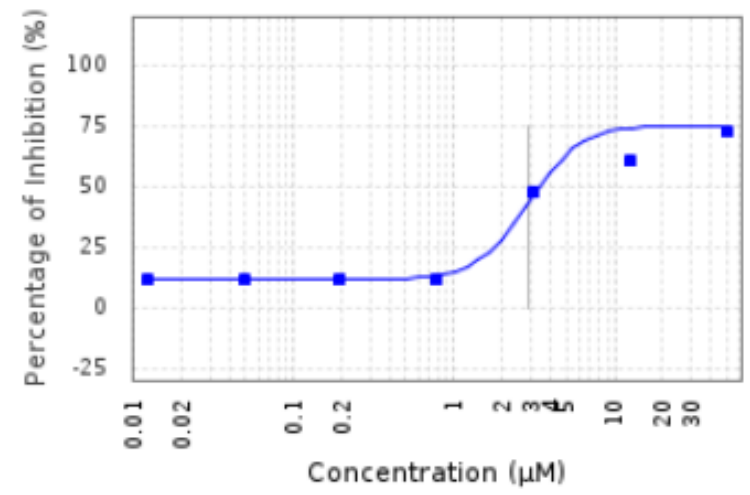

IC50: 2.9112 *(2.357 - 3.5957)* pass
slope=2.700 min=11.176 max=75.000 err=193.358 R^2=0.9553

K562 (IC50 1.3 µM)

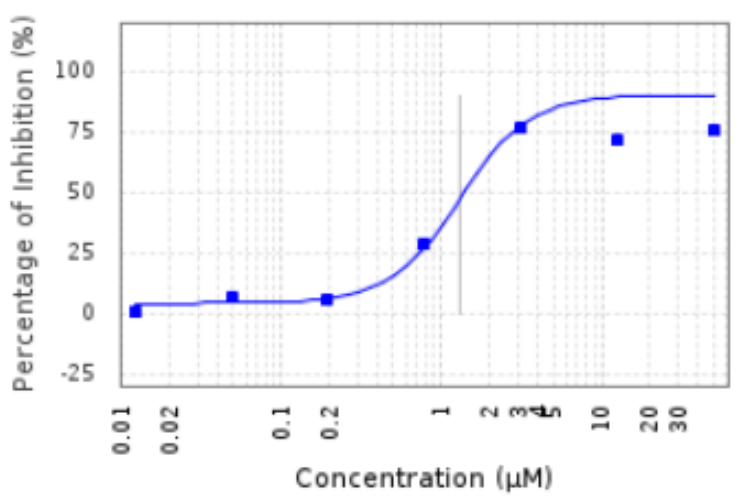

IC50: 1.2948 *(0.8666 - 1.9347)* pass
slope=2.000 min=3.944 max=90.000 err=540.986 R^2=0.9277

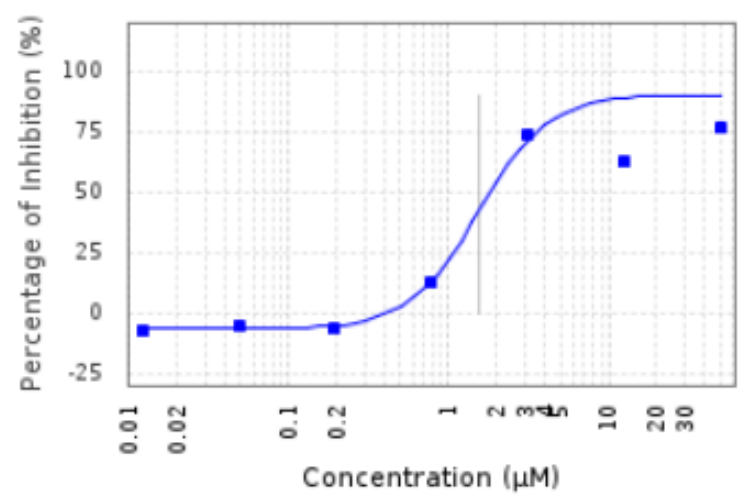

IC50: 1.5382 *(0.9707 - 2.4375)* pass
slope=2.000 min=-6.923 max=90.000 err=867.271 R^2=0.9078

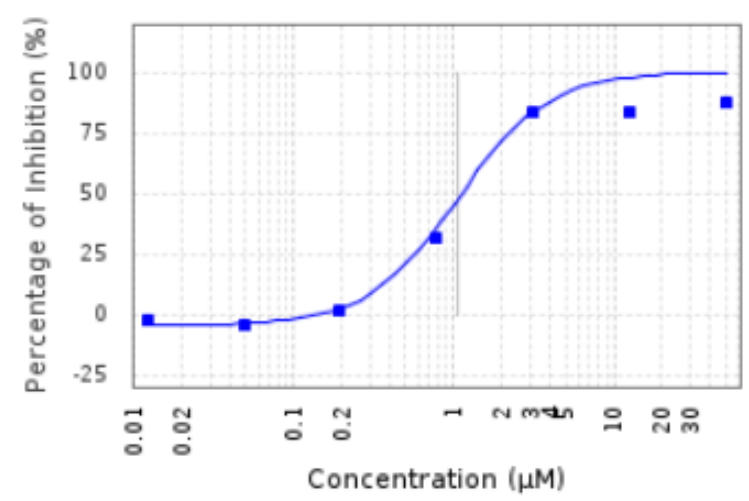

IC50: 1.0507 *(0.7150 - 1.5441)* pass
slope=1.545 min=-4.856 max=100.000 err=361.926 R^2=0.9682

U2OS (IC50 2.8 µM)

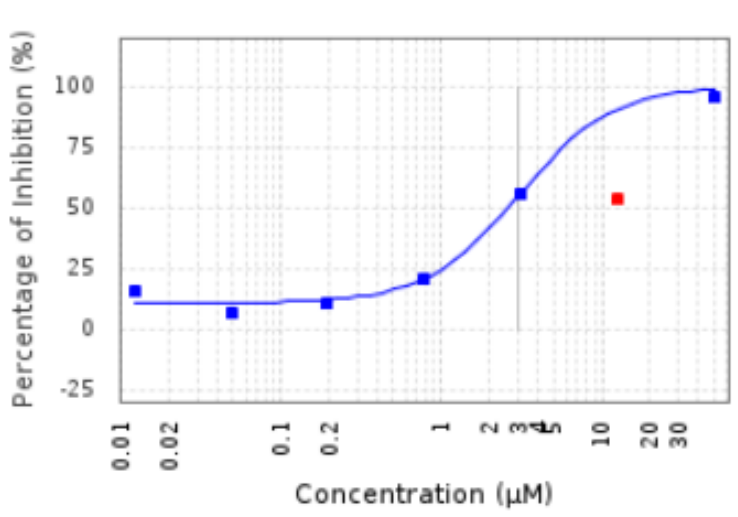

IC50: 3.0469 *(2.4574 - 3.7777)* pass
slope=1.500 min=10.424 max=100.000 err=52.293 R^2=0.9913

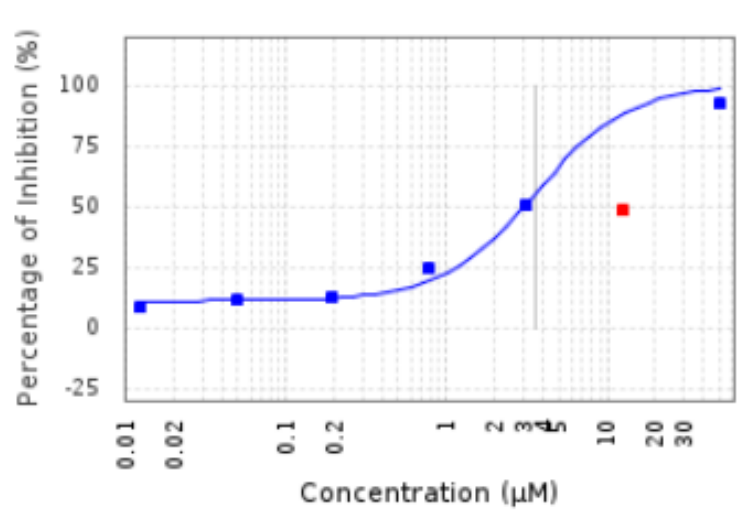

IC50: 3.5892 *(2.6854 - 4.7973)* pass
slope=1.500 min=10.934 max=100.000 err=70.884 R^2=0.9869

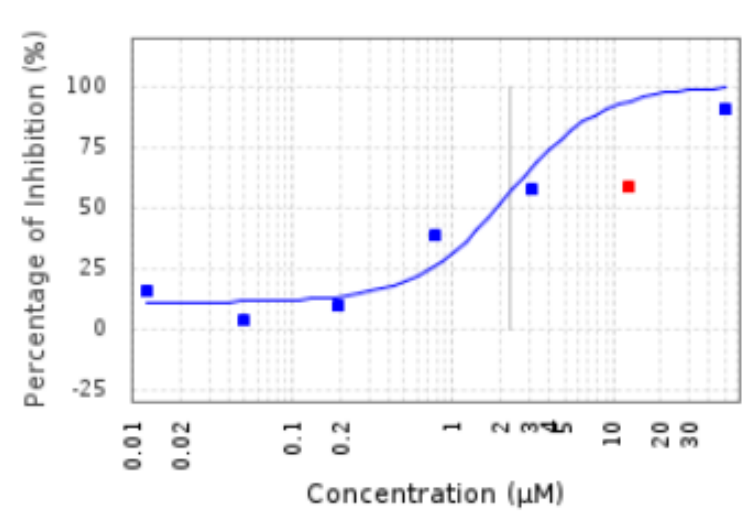

IC50: 2.2359 *(1.3063 - 3.827)* pass
slope=1.500 min=10.768 max=100.000 err=398.811 R^2=0.9296

LEM 241421 (5dd)

BJ (IC50 16 μM)

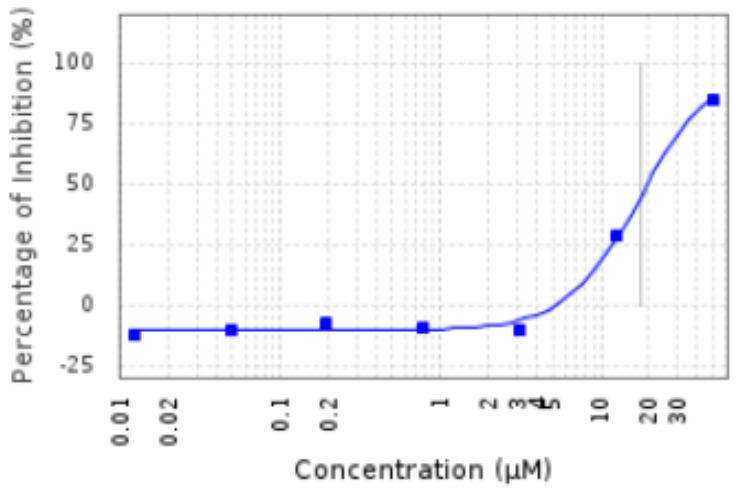

IC50: 17.714 *(12.795 - 24.524)* pass
slope=1.851 min=-10.683 max=100.000 err=29.266 R^2=0.9963

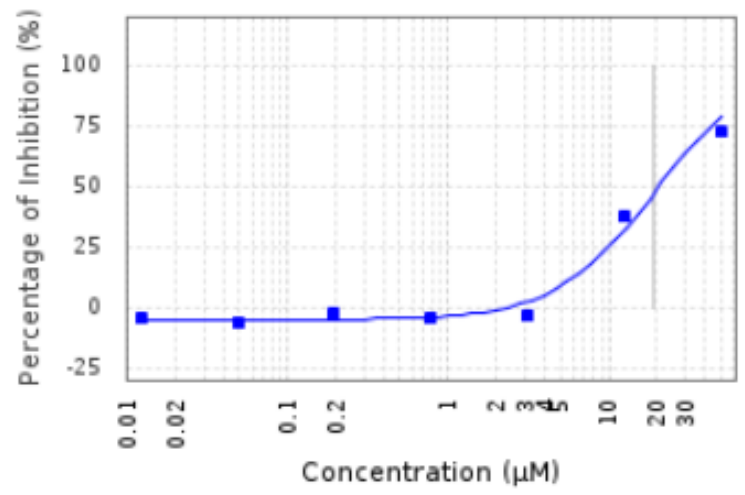

IC50: 19.295 *(13.909 - 26.765)* pass
slope=1.400 min=-5.344 max=100.000 err=101.768 R^2=0.9817

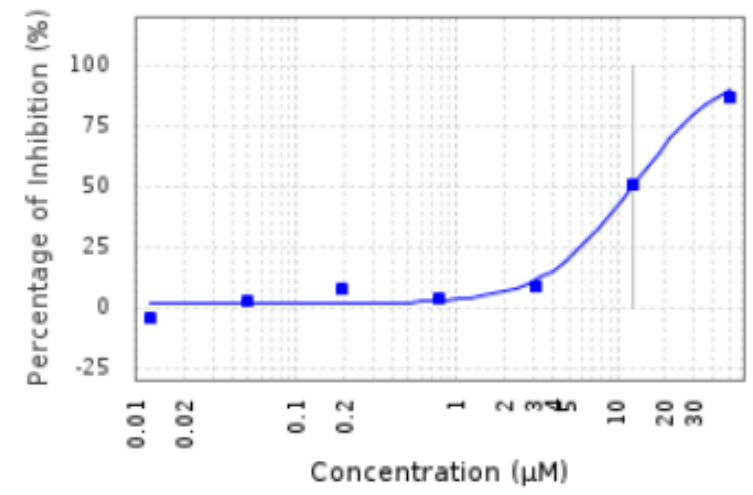

IC50: 12.723 *(9.1219 - 17.744)* pass
slope=1.556 min=1.211 max=100.000 err=84.324 R^2=0.9878

MRC5 (IC50 9.7 μM)

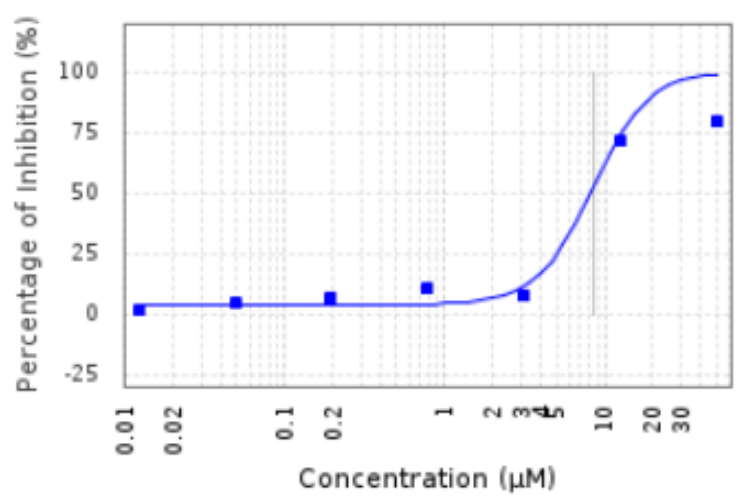

IC50: 8.4331 *(5.6272 - 12.638)* pass
slope=2.503 min=3.676 max=100.000 err=441.724 R^2=0.9372

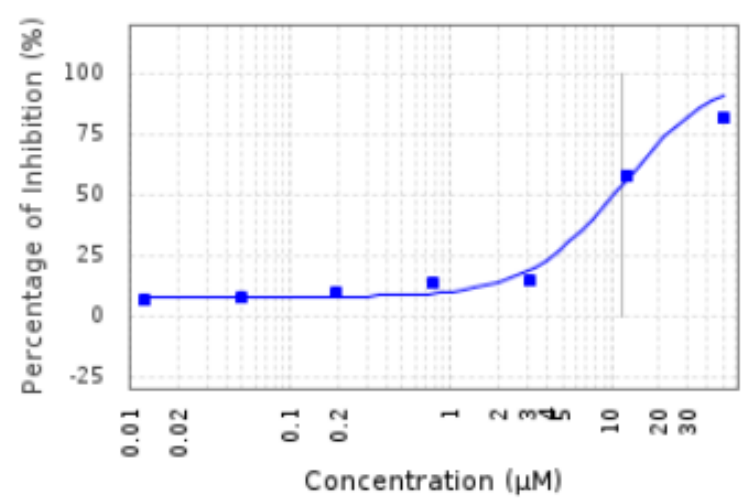

IC50: 11.718 *(7.7134 - 17.801)* pass
slope=1.500 min=7.558 max=100.000 err=124.108 R^2=0.9773

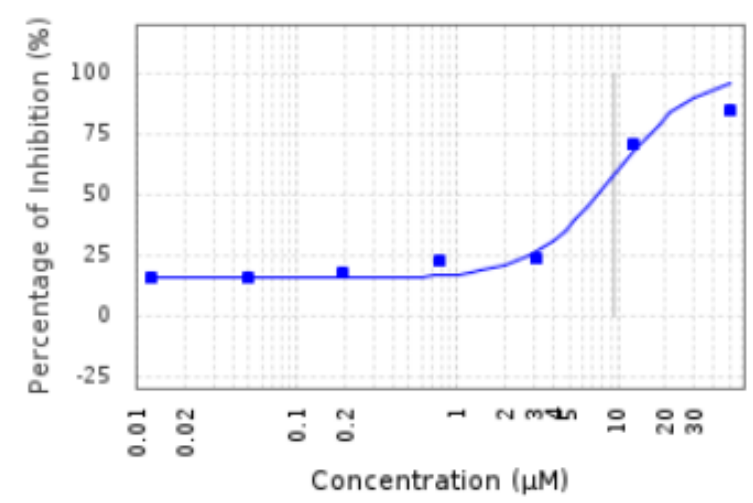

IC50: 9.4215 *(6.6886 - 13.271)* pass
slope=1.700 min=15.000 max=100.000 err=185.968 R^2=0.9633

A549 (IC50 26 μM)

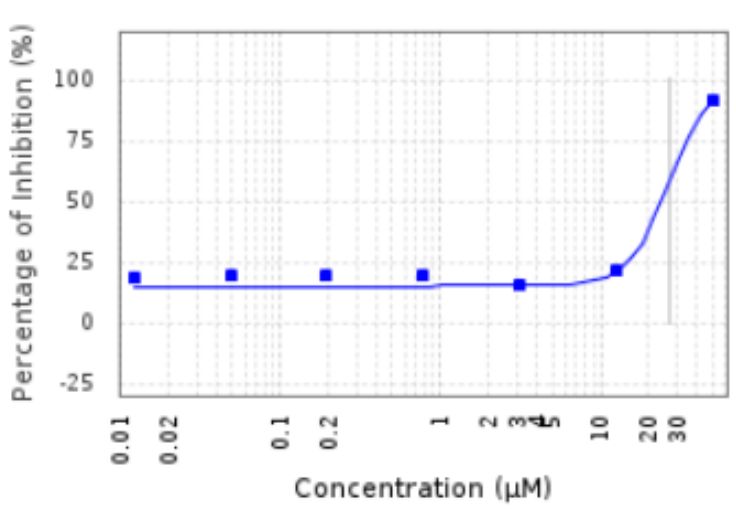

IC50: 26.854 *(9.95737E-9 - 7.2424E10)* pass
slope=3.377 min=15.000 max=100.832 err=65.567 R^2=0.9856

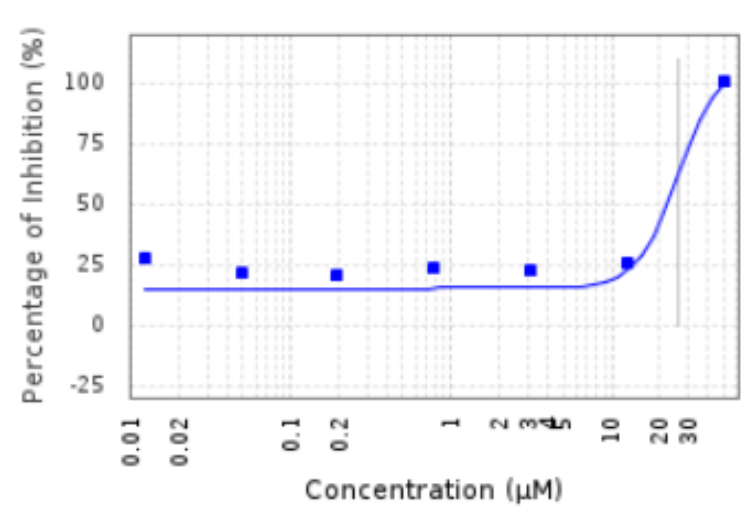

IC50: 26.352 *(1.79284E-14 - 3.87331E16)* pass
slope=3.276 min=15.000 max=110.000 err=352.177 R^2=0.9321

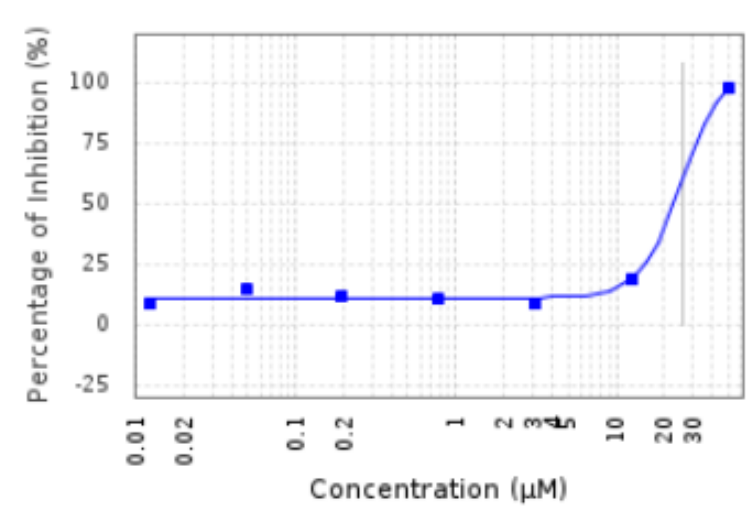

IC50: 25.858 *(22.046 - 30.33)* pass
slope=3.300 min=10.892 max=107.420 err=22.900 R^2=0.9964

CCRF-CEM (IC50 35 μM)

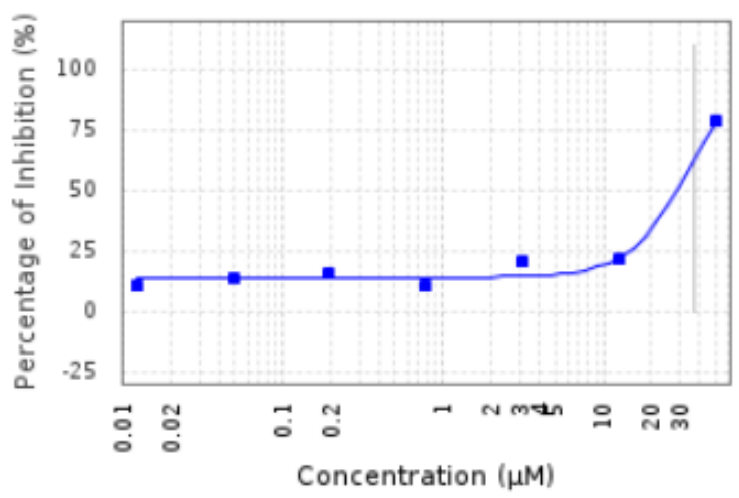

IC50: 36.532 (0.0021 - 636450) pass
slope=2.219 min=13.796 max=110.000 err=62.845 R^2=0.9821

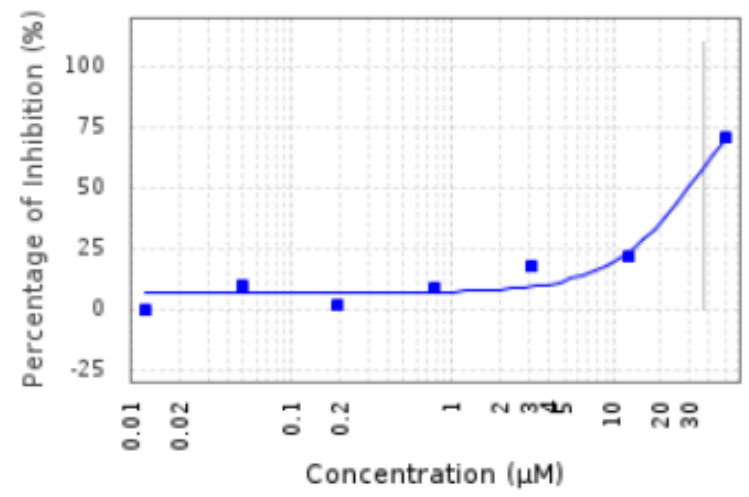

IC50: 37.134 (0.1588 - 8685) pass
slope=1.500 min=6.475 max=110.000 err=164.247 R^2=0.9528

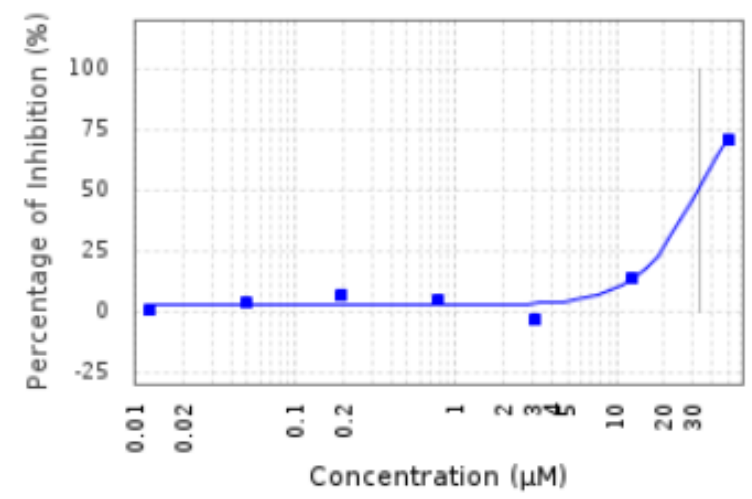

IC50: 33.433 (0.0431 - 25946) pass
slope=2.157 min=2.411 max=100.000 err=68.936 R^2=0.9827

HCT116 (IC50 34 µM)

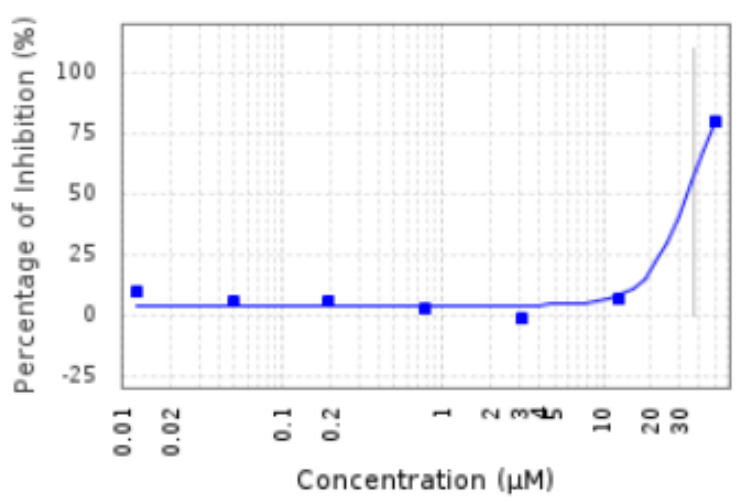

IC50: 36.941 (16.785 - 81.298) pass
slope=3.000 min=3.868 max=110.000 err=76.374 R^2=0.9845

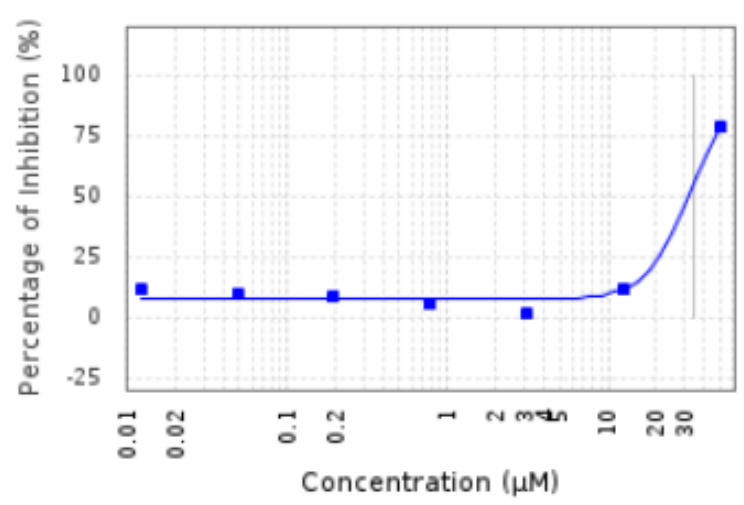

IC50: 33.88 (6.18043E-14 - 1.85725E16) pass
slope=3.064 min=7.316 max=100.000 err=60.627 R^2=0.9860

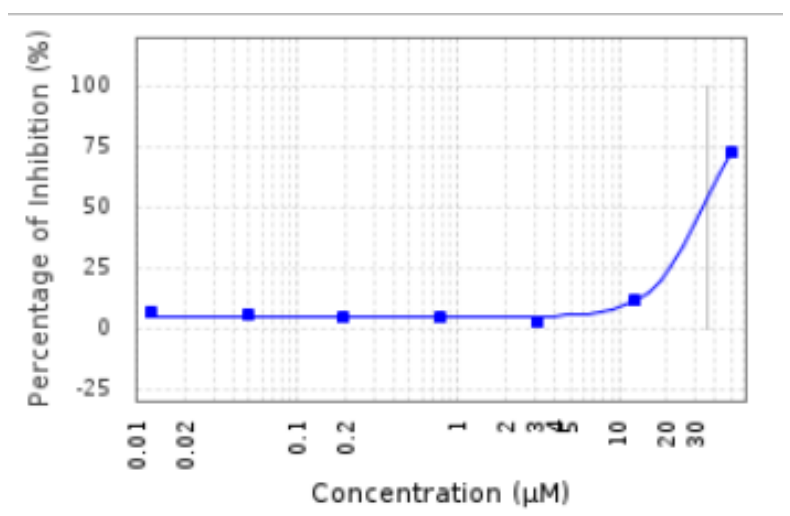

IC50: 35.054 (0.0706 - 17417) pass
slope=2.529 min=4.583 max=100.000 err=11.536 R^2=0.9970

HCT116p53-/- (IC50 17 µM)

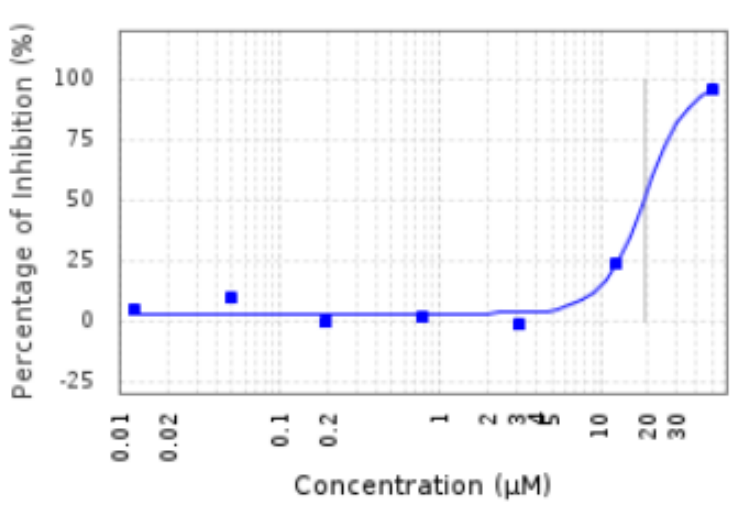

IC50: 19.128 (1.5117 - 242.02) pass
slope=3.136 min=2.865 max=100.000 err=79.265 R^2=0.9889

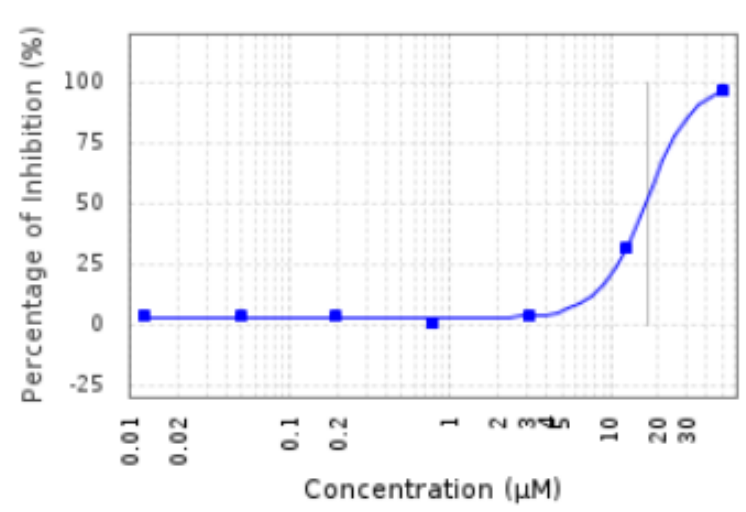

IC50: 16.874 (12.576 - 22.64) pass
slope=2.942 min=2.672 max=100.000 err=7.003 R^2=0.9991

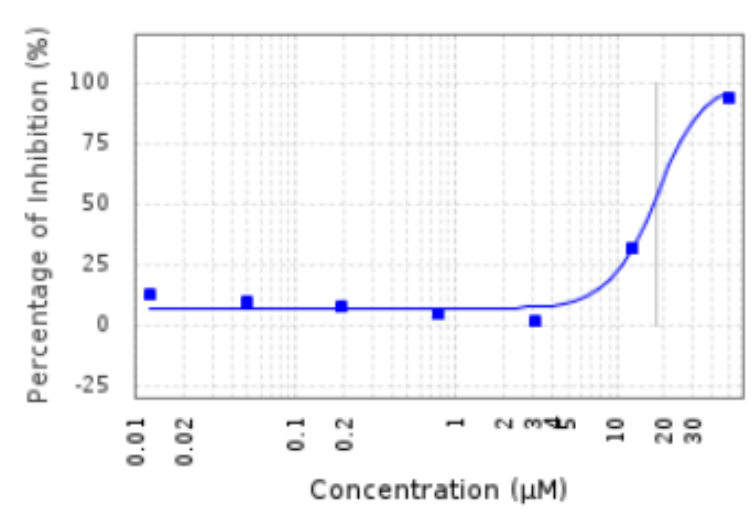

IC50: 17.771 (4.4031 - 71.721) pass
slope=2.945 min=6.732 max=100.000 err=79.266 R^2=0.9877

K562 (IC50 37 µM)

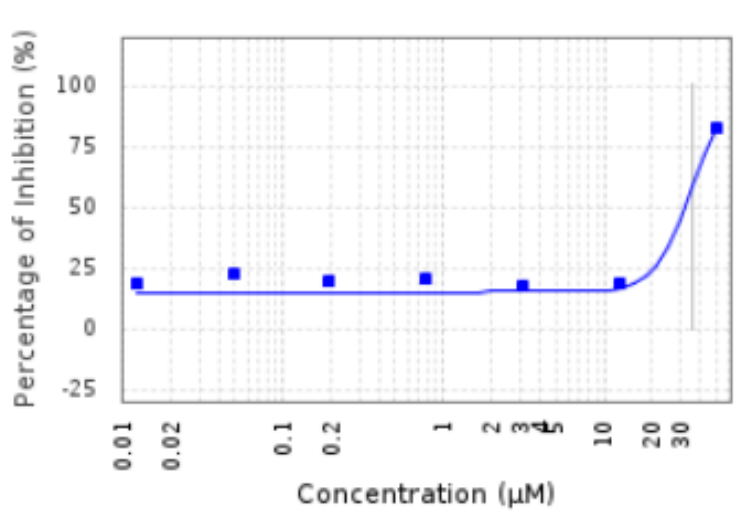

IC50: 35.401 (6.2176 - 201.57) pass
slope=3.800 min=15.000 max=100.280 err=126.110 R^2=0.9630

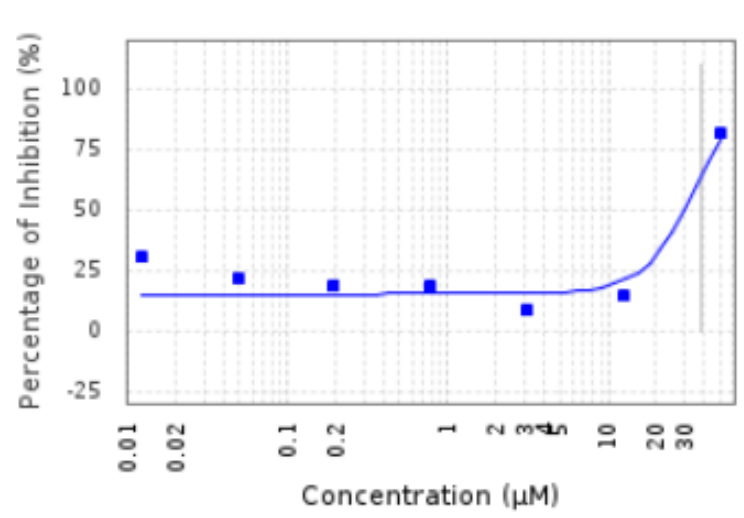

IC50: 37.767 (7.1226 - 200.26) pass
slope=2.500 min=15.000 max=110.000 err=391.116 R^2=0.8915

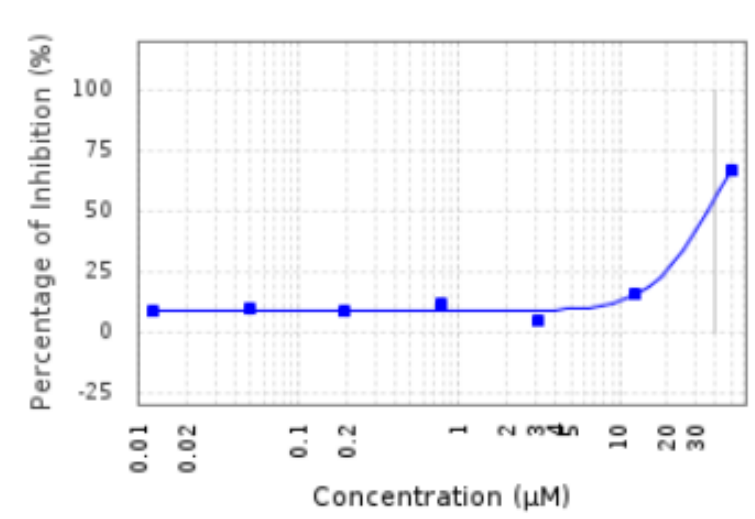

IC50: 38.919 (0.0067 - 227410) pass
slope=2.237 min=8.303 max=100.000 err=26.722 R^2=0.9907

U2OS (IC50 30 µM)

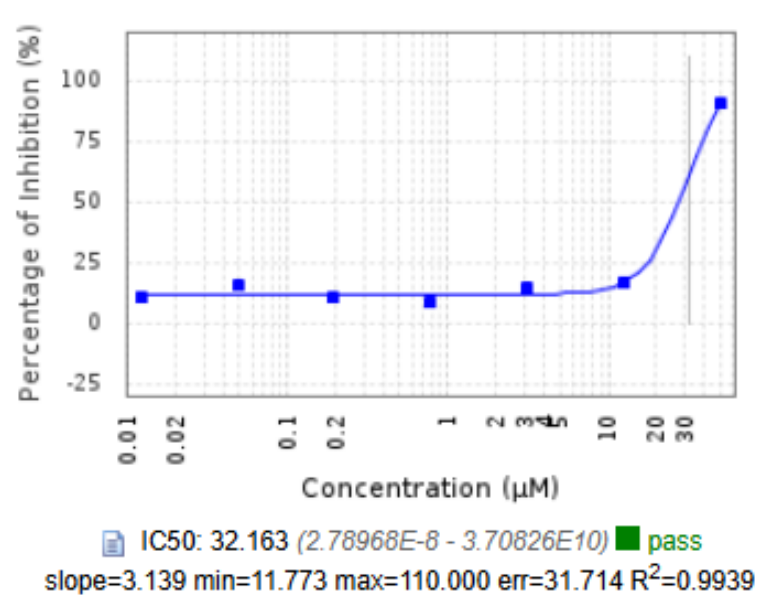

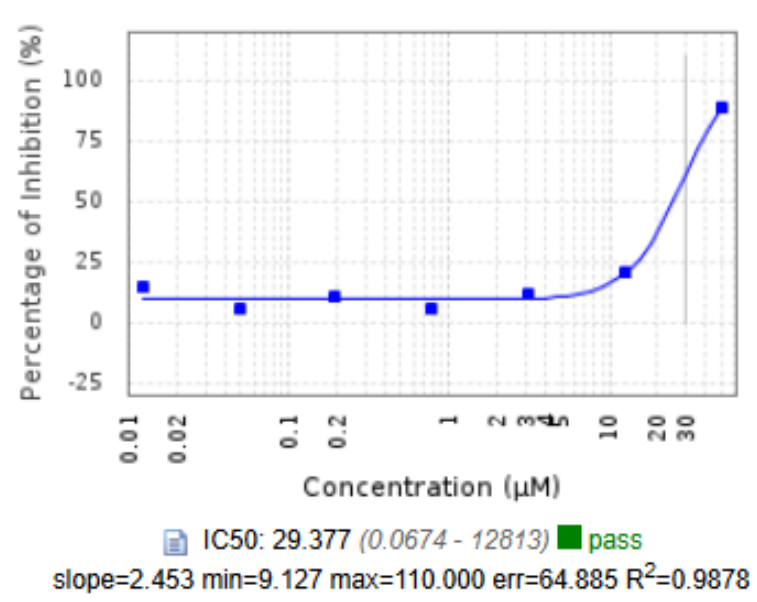

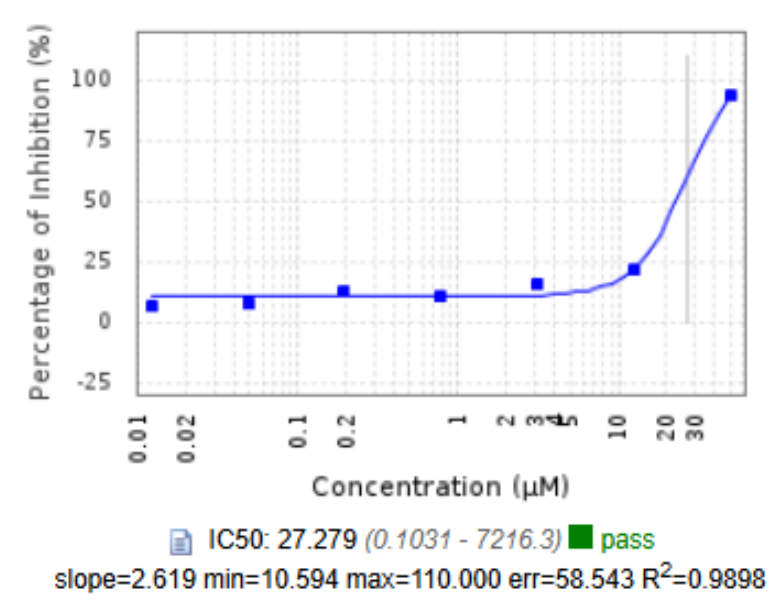

LEM 241417 (8aa)

BJ (IC50 > 50 µM)

Primary screen (50 µM)

plate	well	sample id	PI	pass	conc	inc		
1 (Plate 1)	C9	LEM00241417	24.973		50.0uM	+		
1 (Plate 1)	C10	LEM00241417	19.367		50.0uM	+		
1 (Plate 1)	C11	LEM00241417	18.772		50.0uM	+		

plate	well	sample id	PI	pass	conc	inc		
1 (Plate 1)	C9	LEM00241417	12.242		50.0uM	+		
1 (Plate 1)	C10	LEM00241417	23.442		50.0uM	+		
1 (Plate 1)	C11	LEM00241417	19.708		50.0uM	+		

MRC5 (IC50 > 50 µM)

Primary screen (50 µM)

plate	well	sample id	PI	pass	conc	inc		
1 (Plate 1)	C9	LEM00241417	14.7		50.0uM	+		
1 (Plate 1)	C10	LEM00241417	15.252		50.0uM	+		
1 (Plate 1)	C11	LEM00241417	12.902		50.0uM	+		

plate	well	sample id	PI	pass	conc	inc		
1 (Plate 1)	C9	LEM00241417	15.052		50.0uM	+		
1 (Plate 1)	C10	LEM00241417	12.561		50.0uM	+		
1 (Plate 1)	C11	LEM00241417	12.633		50.0uM	+		

A549 (IC50 > 50 µM)

Primary screen (50 µM)

plate	well	sample id	PI	pass	conc	inc		
1 (Plate 1)	C9	LEM00241417	21.895		50.0uM	+	✓	⊗
1 (Plate 1)	C10	LEM00241417	18.034		50.0uM	+		
1 (Plate 1)	C11	LEM00241417	13.208		50.0uM	+		

plate	well	sample id	PI	pass	conc	inc		
1 (Plate 1)	C9	LEM00241417	21.178		50.0uM	+	✓	⊗
1 (Plate 1)	C10	LEM00241417	17.905		50.0uM	+		
1 (Plate 1)	C11	LEM00241417	15.483		50.0uM	+		

CCRF-CEM (IC50 31 µM)

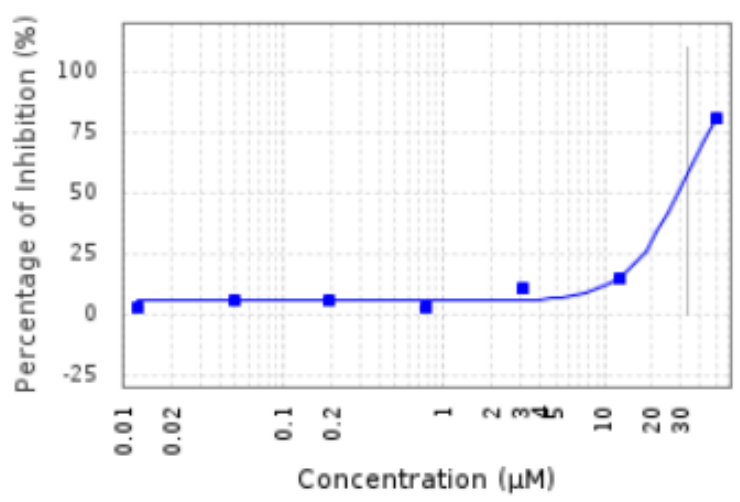

IC50: 33.172 *(0.1508 - 7298.7)* pass
slope=2.304 min=5.225 max=110.000 err=34.693 R^2=0.9928

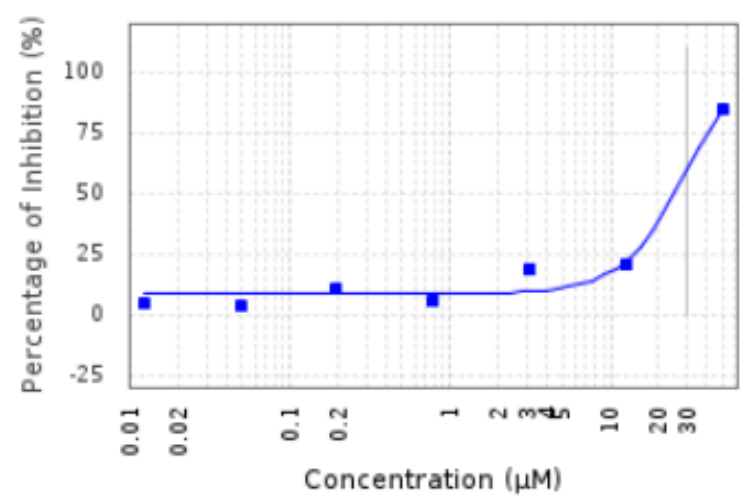

IC50: 29.985 *(0.0875 - 10273)* pass
slope=2.111 min=8.350 max=110.000 err=141.431 R^2=0.9715

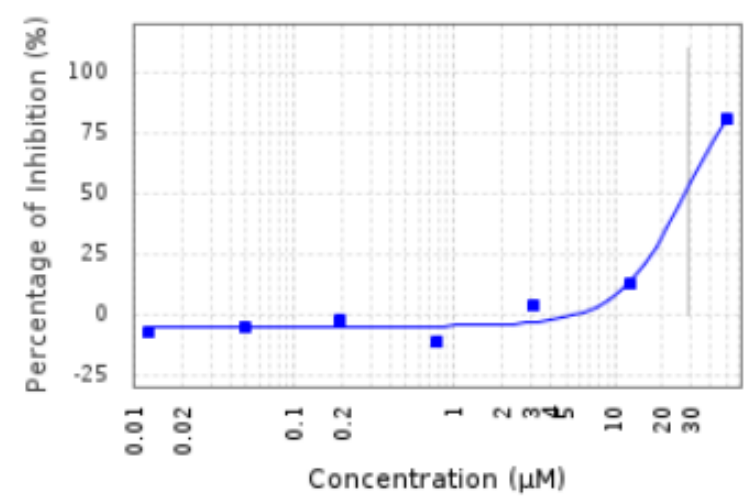

IC50: 29.058 *(1.0645 - 793.19)* pass
slope=1.949 min=-5.131 max=110.000 err=109.358 R^2=0.9824

HCT116 (IC50 > 50 µM)

Primary screen (50 µM)

plate	well	sample id	PI	pass	conc	inc		
1 (Plate 1)	C9	LEM00241417	10.845		50.0uM	+	✓	⊗
1 (Plate 1)	C10	LEM00241417	5.126		50.0uM	+		
1 (Plate 1)	C11	LEM00241417	1.7432		50.0uM	+		

plate	well	sample id	PI	pass	conc	inc		
1 (Plate 1)	C9	LEM00241417	10.131		50.0uM	+	✓	⊗
1 (Plate 1)	C10	LEM00241417	11.838		50.0uM	+		
1 (Plate 1)	C11	LEM00241417	5.6295		50.0uM	+		

HCT116p53-/- (IC50 > 50 µM)

Primary screen (50 µM)

plate	well	sample id	PI	pass	conc	inc		
1 (Plate 1)	C9	LEM00241417	7.5916		50.0uM	+	✓	⊗
1 (Plate 1)	C10	LEM00241417	16.323		50.0uM	+		
1 (Plate 1)	C11	LEM00241417	24.623		50.0uM	+		

plate	well	sample id	PI	pass	conc	inc		
1 (Plate 1)	C9	LEM00241417	11.385		50.0uM	+	✓	⊗
1 (Plate 1)	C10	LEM00241417	8.6248		50.0uM	+		
1 (Plate 1)	C11	LEM00241417	0.3432		50.0uM	+		

K562 (IC50 33 µM)

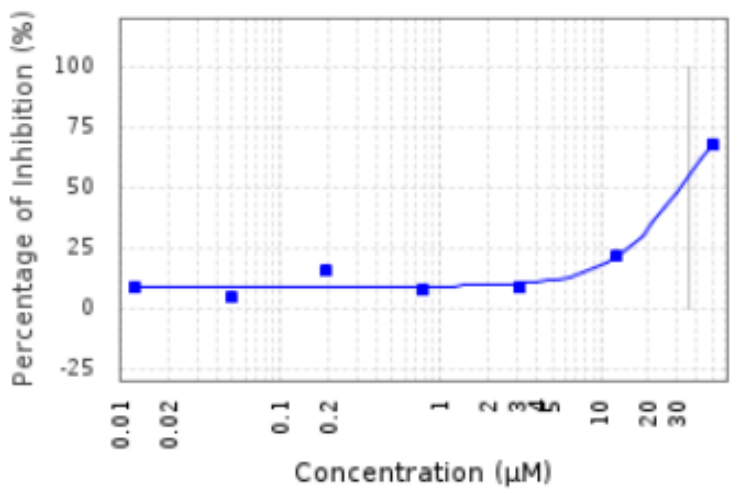

IC50: 35.457 (0.3161 - 3977.4) pass
slope=1.790 min=8.730 max=100.000 err=60.799 R^2=0.9795

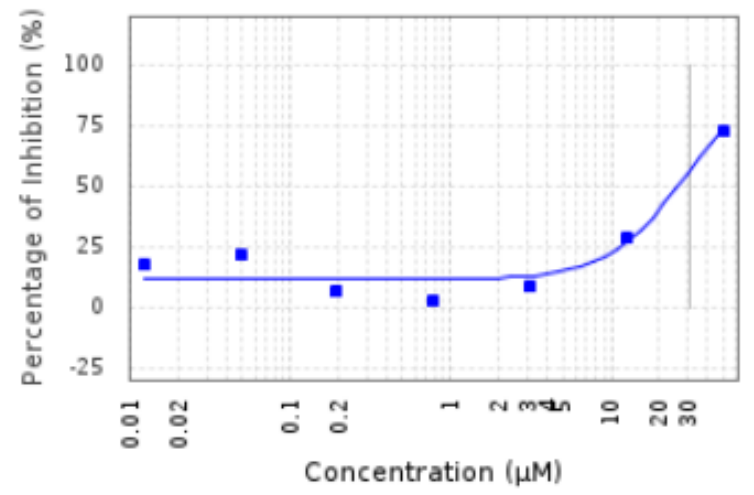

IC50: 30.832 (0.0543 - 17492) pass
slope=1.725 min=11.149 max=100.000 err=275.530 R^2=0.9192

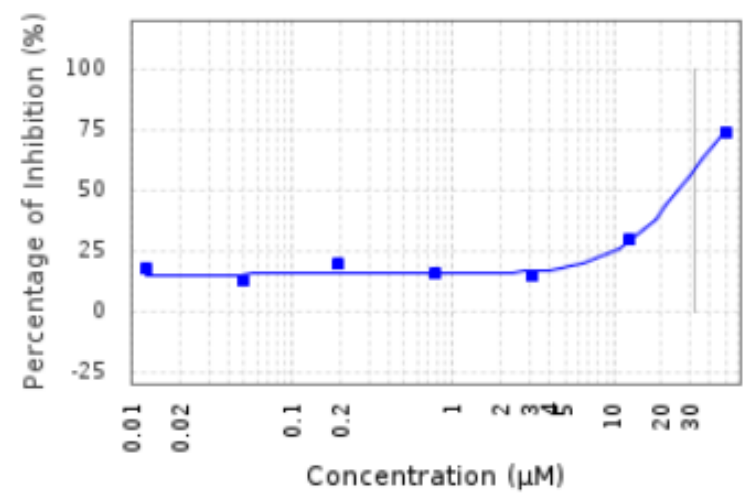

IC50: 31.514 (2.3902 - 415.5) pass
slope=1.790 min=15.000 max=100.000 err=31.790 R^2=0.9888

U2OS (IC50 > 50 µM)

Primary screen (50 µM)

plate	well	sample id	PI	pass	conc	inc	✓	⊗
1 (Plate 1)	C9	LEM00241417	35.39	●	50.0uM	+	✓	⊗
1 (Plate 1)	C10	LEM00241417	38.376	●	50.0uM	+		
1 (Plate 1)	C11	LEM00241417	39.371	●	50.0uM	+		

plate	well	sample id	PI	pass	conc	inc	✓	⊗
1 (Plate 1)	C9	LEM00241417	33.68	●	50.0uM	+	✓	⊗
1 (Plate 1)	C10	LEM00241417	31.733	●	50.0uM	+		
1 (Plate 1)	C11	LEM00241417	30.21	●	50.0uM	+		

LEM 241419 (8ac)

BJ (IC50 > 50 µM)

Primary screen (50 µM)

plate	well	sample id	PI	pass	conc	inc	✓	⊗
1 (Plate 1)	E9	LEM00241419	4.5019	●	50.0uM	+	✓	⊗
1 (Plate 1)	E10	LEM00241419	2.7181	●	50.0uM	+		
1 (Plate 1)	E11	LEM00241419	4.332	●	50.0uM	+		

plate	well	sample id	PI	pass	conc	inc	✓	⊗
1 (Plate 1)	E9	LEM00241419	2.909	●	50.0uM	+	✓	⊗
1 (Plate 1)	E10	LEM00241419	2.2302	●	50.0uM	+		
1 (Plate 1)	E11	LEM00241419	3.3332	●	50.0uM	+		

MRC5 (IC50 > 50 µM)

Primary screen (50 µM)

plate	well	sample id	PI	pass	conc	inc	✓	⊗
1 (Plate 1)	E9	LEM00241419	20.506	●	50.0uM	+	✓	⊗
1 (Plate 1)	E10	LEM00241419	21.957	●	50.0uM	+		
1 (Plate 1)	E11	LEM00241419	19.676	●	50.0uM	+		

plate	well	sample id	PI	pass	conc	inc	✓	⊗
1 (Plate 1)	E9	LEM00241419	16.903	●	50.0uM	+	✓	⊗
1 (Plate 1)	E10	LEM00241419	18.895	●	50.0uM	+		
1 (Plate 1)	E11	LEM00241419	14.483	●	50.0uM	+		

A549 (IC50 > 50 µM)

Primary screen (50 µM)

plate	well	sample id	PI	pass	conc	inc	✓	⊗
1 (Plate 1)	E9	LEM00241419	8.8644	●	50.0uM	+	✓	⊗
1 (Plate 1)	E10	LEM00241419	9.0712	●	50.0uM	+		
1 (Plate 1)	E11	LEM00241419	8.2439	●	50.0uM	+		

plate	well	sample id	PI	pass	conc	inc	✓	⊗
1 (Plate 1)	E9	LEM00241419	13.912	●	50.0uM	+	✓	⊗
1 (Plate 1)	E10	LEM00241419	13.126	●	50.0uM	+		
1 (Plate 1)	E11	LEM00241419	14.239	●	50.0uM	+		

CCRF-CEM (IC50 43 µM)

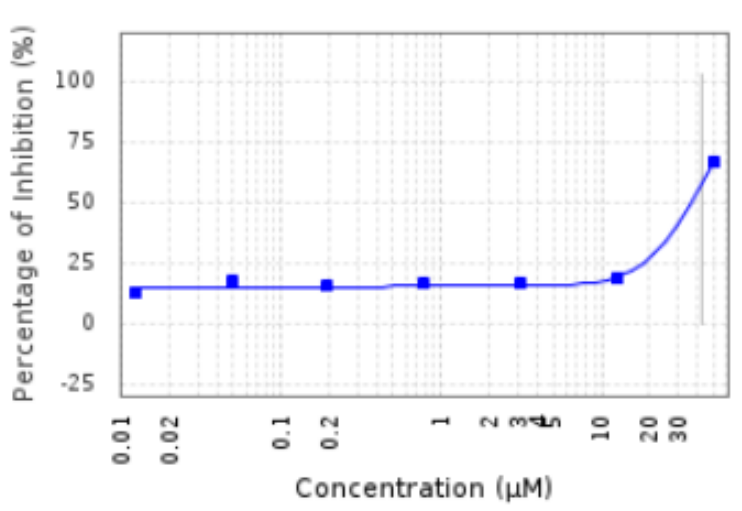

IC50: 42.991 (5.46735E-5 - 3.38048E7) pass
slope=2.478 min=15.000 max=102.201 err=10.937 R^2=0.9951

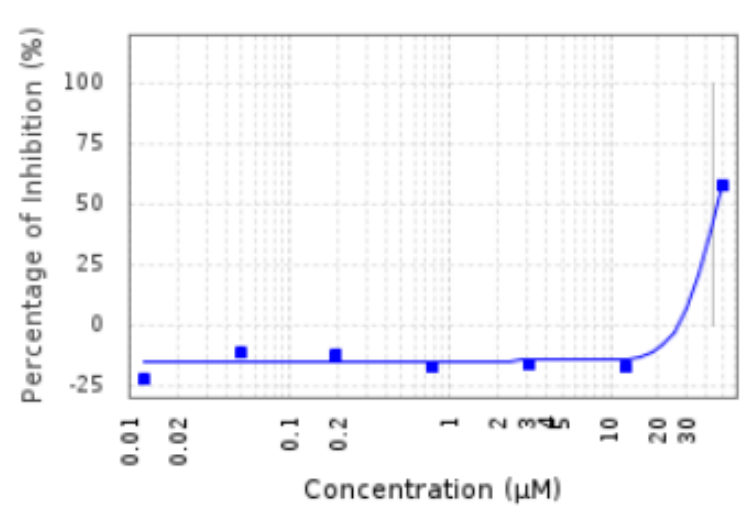

IC50: 43.563 (0 - ∞) pass
slope=4.015 min=-15.000 max=100.000 err=94.244 R^2=0.9802

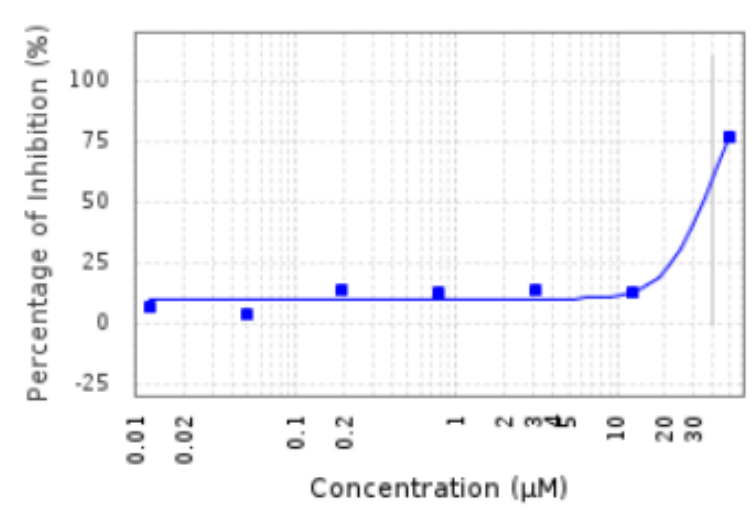

IC50: 39.555 (12.987 - 120.48) pass
slope=3.000 min=9.611 max=110.000 err=81.740 R^2=0.9790

HCT116 (IC50 > 50 µM)

Primary screen (50 µM)

plate	well	sample id	PI	pass	conc	inc	✓	⊗
1 (Plate 1)	E9	LEM00241419	3.5957	●	50.0uM	+	✓	⊗
1 (Plate 1)	E10	LEM00241419	3.9984	●	50.0uM	+		
1 (Plate 1)	E11	LEM00241419	4.3206	●	50.0uM	+		

plate	well	sample id	PI	pass	conc	inc	✓	⊗
1 (Plate 1)	E9	LEM00241419	1.5163	●	50.0uM	+	✓	⊗
1 (Plate 1)	E10	LEM00241419	1.2835	●	50.0uM	+		
1 (Plate 1)	E11	LEM00241419	3.0685	●	50.0uM	+		

HCT116p53-/- (IC50 > 50 µM)

Primary screen (50 µM)

plate	well	sample id	PI	pass	conc	inc	✓	⊗
1 (Plate 1)	C9	LEM00241417	7.5916	●	50.0uM	+	✓	⊗
1 (Plate 1)	C10	LEM00241417	16.323	●	50.0uM	+		
1 (Plate 1)	C11	LEM00241417	24.623	●	50.0uM	+		

plate	well	sample id	PI	pass	conc	inc	✓	⊗
1 (Plate 1)	E9	LEM00241419	2.2858	●	50.0uM	+	✓	⊗
1 (Plate 1)	E10	LEM00241419	0.7522	●	50.0uM	+		
1 (Plate 1)	E11	LEM00241419	-2.0083	●	50.0uM	+		

K562 (IC50 > 50 μM)

Primary screen (50 μM)

plate	well	sample id	PI	pass	conc	inc	✓	⊗
1 (Plate 1)	E9	LEM00241419	37.251	●	50.0uM	+	✓	⊗
1 (Plate 1)	E10	LEM00241419	38.892	●	50.0uM	+		
1 (Plate 1)	E11	LEM00241419	27.769	●	50.0uM	+		

plate	well	sample id	PI	pass	conc	inc	✓	⊗
1 (Plate 1)	E9	LEM00241419	17.907	●	50.0uM	+	✓	⊗
1 (Plate 1)	E10	LEM00241419	11.711	●	50.0uM	+		
1 (Plate 1)	E11	LEM00241419	12.153	●	50.0uM	+		

U2OS (IC50 > 50 μM)

Primary screen (50 μM)

plate	well	sample id	PI	pass	conc	inc	✓	⊗
1 (Plate 1)	E9	LEM00241419	13.856	●	50.0uM	+	✓	⊗
1 (Plate 1)	E10	LEM00241419	16.661	●	50.0uM	+		
1 (Plate 1)	E11	LEM00241419	13.675	●	50.0uM	+		

plate	well	sample id	PI	pass	conc	inc	✓	⊗
1 (Plate 1)	E9	LEM00241419	18.953	●	50.0uM	+	✓	⊗
1 (Plate 1)	E10	LEM00241419	16.922	●	50.0uM	+		
1 (Plate 1)	E11	LEM00241419	16.075	●	50.0uM	+		

LEM 241418 (11aa)

BJ (IC50 > 50 μM)

Primary screen (50 μM)

plate	well	sample id	PI	pass	conc	inc	✓	⊗
1 (Plate 1)	D9	LEM00241418	13.93	●	50.0uM	+	✓	⊗
1 (Plate 1)	D10	LEM00241418	15.969	●	50.0uM	+		
1 (Plate 1)	D11	LEM00241418	13.93	●	50.0uM	+		

plate	well	sample id	PI	pass	conc	inc	✓	⊗
1 (Plate 1)	D9	LEM00241418	17.757	●	50.0uM	+	✓	⊗
1 (Plate 1)	D10	LEM00241418	19.284	●	50.0uM	+		
1 (Plate 1)	D11	LEM00241418	16.569	●	50.0uM	+		

MRC5 (IC50 > 50 μM)

Primary screen (50 μM)

plate	well	sample id	PI	pass	conc	inc	✓	⊗
1 (Plate 1)	D9	LEM00241418	17.603		50.0uM	+	✓	⊗
1 (Plate 1)	D10	LEM00241418	16.98		50.0uM	+		
1 (Plate 1)	D11	LEM00241418	21.542		50.0uM	+		

plate	well	sample id	PI	pass	conc	inc	✓	⊗
1 (Plate 1)	D9	LEM00241418	12.917		50.0uM	+	✓	⊗
1 (Plate 1)	D10	LEM00241418	12.419		50.0uM	+		
1 (Plate 1)	D11	LEM00241418	11.494		50.0uM	+		

A549 (IC50 > 50 μM)

Primary screen (50 μM)

plate	well	sample id	PI	pass	conc	inc	✓	⊗
1 (Plate 1)	D9	LEM00241418	14.587		50.0uM	+	✓	⊗
1 (Plate 1)	D10	LEM00241418	11.967		50.0uM	+		
1 (Plate 1)	D11	LEM00241418	11.071		50.0uM	+		

plate	well	sample id	PI	pass	conc	inc	✓	⊗
1 (Plate 1)	D9	LEM00241418	16.727		50.0uM	+	✓	⊗
1 (Plate 1)	D10	LEM00241418	15.221		50.0uM	+		
1 (Plate 1)	D11	LEM00241418	16.399		50.0uM	+		

CCRF-CEM (IC50 39 μM)

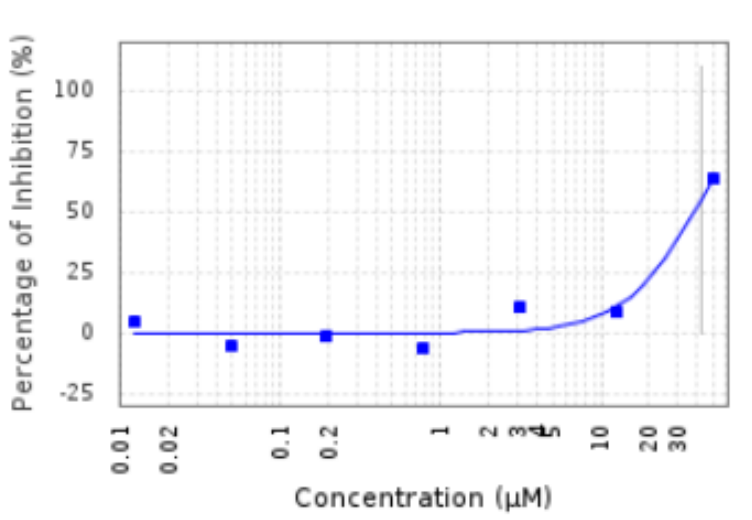

IC50: 42.188 (0.0002 - 9.36315E6) pass
slope=1.824 min=-0.216 max=110.000 err=192.019 R^2=0.9460

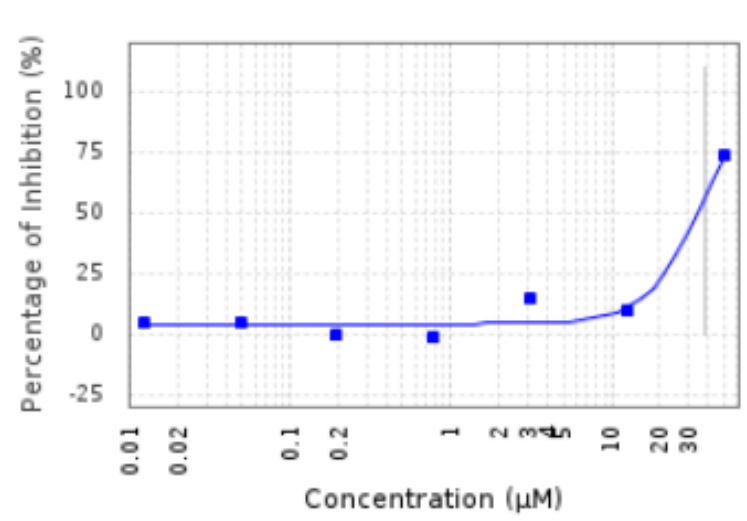

IC50: 38.599 (1.46932E-9 - 1.01398E12) pass
slope=2.403 min=3.963 max=110.000 err=157.843 R^2=0.9620

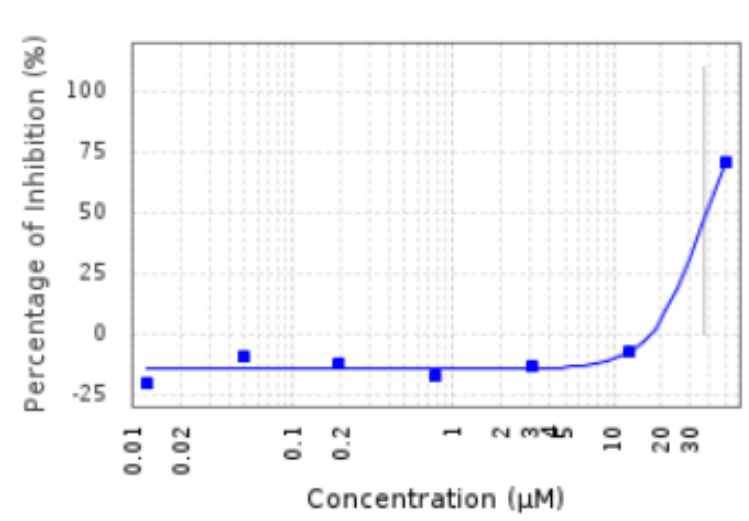

IC50: 37.258 (7.43471E-7 - 1.86713E9) pass
slope=2.598 min=-14.606 max=110.000 err=75.825 R^2=0.9876

HCT116 (IC50 > 50 μM)

Primary screen (50 μM)

plate	well	sample id	PI	pass	conc	inc	✓	⊗
1 (Plate 1)	D9	LEM00241418	17.207		50.0uM	+	✓	⊗
1 (Plate 1)	D10	LEM00241418	12.294		50.0uM	+		
1 (Plate 1)	D11	LEM00241418	13.1		50.0uM	+		

plate	well	sample id	PI	pass	conc	inc	✓	⊗
1 (Plate 1)	D9	LEM00241418	15.408		50.0uM	+	✓	⊗
1 (Plate 1)	D10	LEM00241418	11.14		50.0uM	+		
1 (Plate 1)	D11	LEM00241418	7.7249		50.0uM	+		

HCT116p53-/- (IC50 > 50 µM)

Primary screen (50 µM)

plate	well	sample id	PI	pass	conc	inc		
1 (Plate 1)	D9	LEM00241418	9.6397		50.0uM	+	✓	✗
1 (Plate 1)	D10	LEM00241418	7.1605		50.0uM	+		
1 (Plate 1)	D11	LEM00241418	10.61		50.0uM	+		

plate	well	sample id	PI	pass	conc	inc		
1 (Plate 1)	D9	LEM00241418	15.884		50.0uM	+	✓	✗
1 (Plate 1)	D10	LEM00241418	6.4778		50.0uM	+		
1 (Plate 1)	D11	LEM00241418	3.8195		50.0uM	+		

K562 (IC50 > 50 µM)

Primary screen (50 µM)

plate	well	sample id	PI	pass	conc	inc		
1 (Plate 1)	D9	LEM00241418	33.33		50.0uM	+	✓	✗
1 (Plate 1)	D10	LEM00241418	34.607		50.0uM	+		
1 (Plate 1)	D11	LEM00241418	35.701		50.0uM	+		

plate	well	sample id	PI	pass	conc	inc		
1 (Plate 1)	D9	LEM00241418	22.333		50.0uM	+	✓	✗
1 (Plate 1)	D10	LEM00241418	32.513		50.0uM	+		
1 (Plate 1)	D11	LEM00241418	12.485		50.0uM	+		

U2OS (IC50 44 µM)

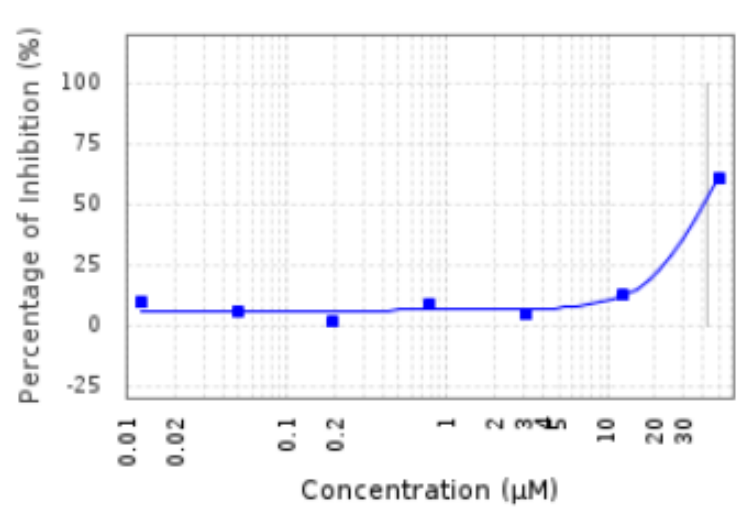

IC50: 42.75 (2.1296E-5 - 8.5816E7) pass
slope=2.200 min=5.996 max=100.000 err=45.786 R^2=0.9822

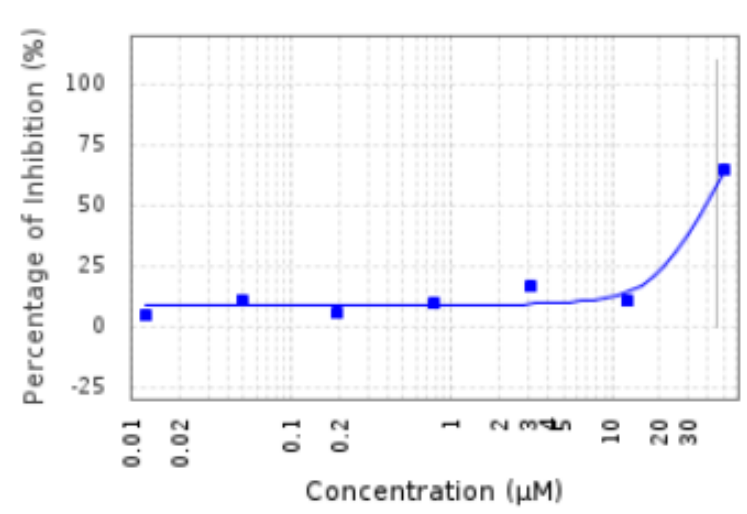

IC50: 46.148 (13.496 - 157.79) pass
slope=2.200 min=8.699 max=110.000 err=98.253 R^2=0.9632

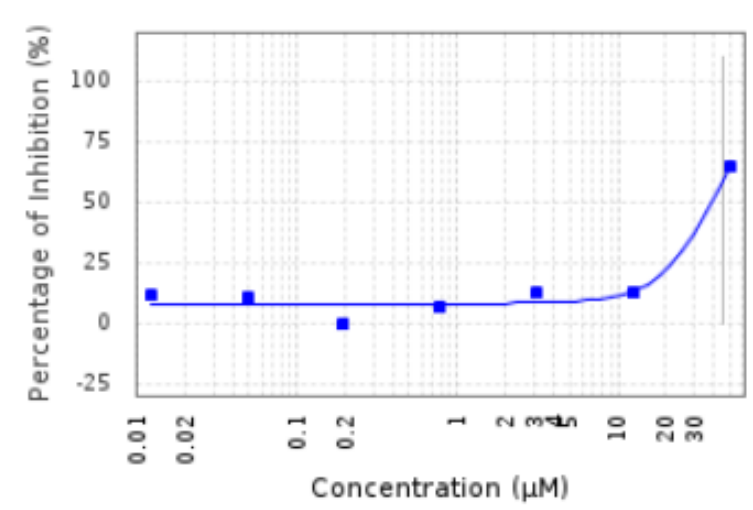

IC50: 45.405 (1.74646E-11 - 1.18045E14) pass
slope=2.273 min=7.880 max=110.000 err=101.167 R^2=0.9637

LEM 241420 (11ac)

BJ (IC50 > 50 µM)

Primary screen (50 µM)

plate	well	sample id	PI	pass	conc	inc		
1 (Plate 1)	F9	LEM00241420	22.17		50.0uM	+	✓	✗
1 (Plate 1)	F10	LEM00241420	27.096		50.0uM	+		
1 (Plate 1)	F11	LEM00241420	26.077		50.0uM	+		

plate	well	sample id	PI	pass	conc	inc		
1 (Plate 1)	F9	LEM00241420	20.133		50.0uM	+	✓	✗
1 (Plate 1)	F10	LEM00241420	20.811		50.0uM	+		
1 (Plate 1)	F11	LEM00241420	19.878		50.0uM	+		

MRC5 (IC50 > 50 µM)

Primary screen (50 µM)

plate	well	sample id	PI	pass	conc	inc
1 (Plate 1)	F9	LEM00241420	31.841		50.0uM	+
1 (Plate 1)	F10	LEM00241420	25.828		50.0uM	+
1 (Plate 1)	F11	LEM00241420	26.519		50.0uM	+

plate	well	sample id	PI	pass	conc	inc
1 (Plate 1)	F9	LEM00241420	25.301		50.0uM	+
1 (Plate 1)	F10	LEM00241420	27.507		50.0uM	+
1 (Plate 1)	F11	LEM00241420	24.945		50.0uM	+

A549 (IC50 > 50 µM)

Primary screen (50 µM)

plate	well	sample id	PI	pass	conc	inc
1 (Plate 1)	F9	LEM00241420	18.793		50.0uM	+
1 (Plate 1)	F10	LEM00241420	16.517		50.0uM	+
1 (Plate 1)	F11	LEM00241420	14.311		50.0uM	+

plate	well	sample id	PI	pass	conc	inc
1 (Plate 1)	F9	LEM00241420	20.131		50.0uM	+
1 (Plate 1)	F10	LEM00241420	18.167		50.0uM	+
1 (Plate 1)	F11	LEM00241420	20.72		50.0uM	+

CCRF-CEM (IC50 28 µM)

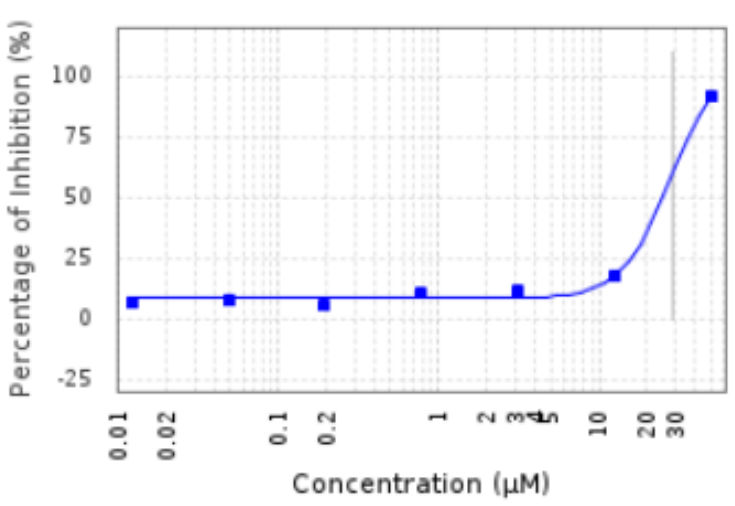

IC50: 28.69 (0.1766 - 4660) pass
slope=2.747 min=8.090 max=110.000 err=22.334 R^2=0.9962

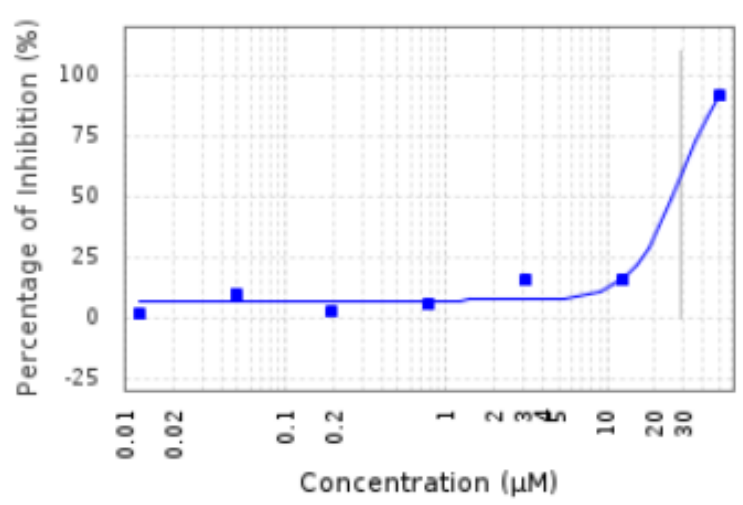

IC50: 29.178 (4.27481E-5 - 1.99152E7) pass
slope=2.773 min=6.983 max=110.000 err=125.842 R^2=0.9792

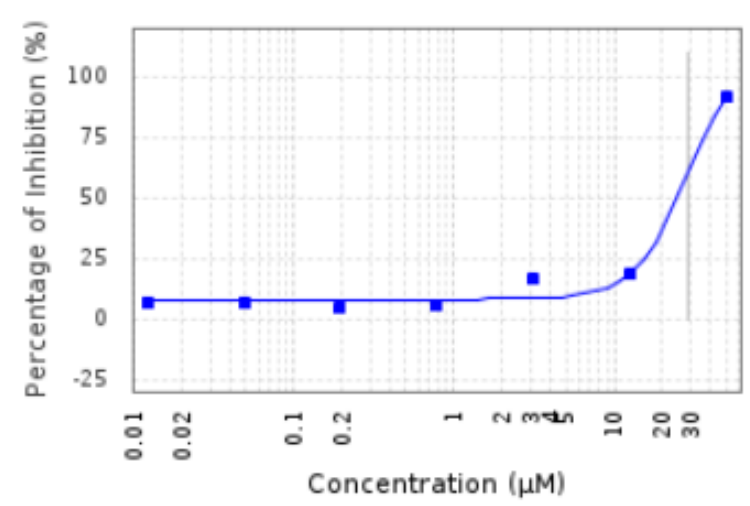

IC50: 28.381 (0.0142 - 56588) pass
slope=2.633 min=7.946 max=110.000 err=78.530 R^2=0.9867

HCT116 (IC50 > 50 µM)

Primary screen (50 µM)

plate	well	sample id	PI	pass	conc	inc
1 (Plate 1)	F9	LEM00241420	13.583		50.0uM	+
1 (Plate 1)	F10	LEM00241420	17.932		50.0uM	+
1 (Plate 1)	F11	LEM00241420	9.9586		50.0uM	+

plate	well	sample id	PI	pass	conc	inc
1 (Plate 1)	F9	LEM00241420	16.339		50.0uM	+
1 (Plate 1)	F10	LEM00241420	10.596		50.0uM	+
1 (Plate 1)	F11	LEM00241420	13.157		50.0uM	+

HCT116p53-/- (IC50 > 50 µM)

Primary screen (50 µM)

plate	well	sample id	PI	pass	conc	inc	✓	⊗
1 (Plate 1)	F9	LEM00241420	11.903	●	50.0uM	+	✓	⊗
1 (Plate 1)	F10	LEM00241420	11.257	●	50.0uM	+		
1 (Plate 1)	F11	LEM00241420	10.933	●	50.0uM	+		

plate	well	sample id	PI	pass	conc	inc	✓	⊗
1 (Plate 1)	F9	LEM00241420	16.395	●	50.0uM	+	✓	⊗
1 (Plate 1)	F10	LEM00241420	17.929	●	50.0uM	+		
1 (Plate 1)	F11	LEM00241420	13.635	●	50.0uM	+		

K562 (IC50 37 µM)

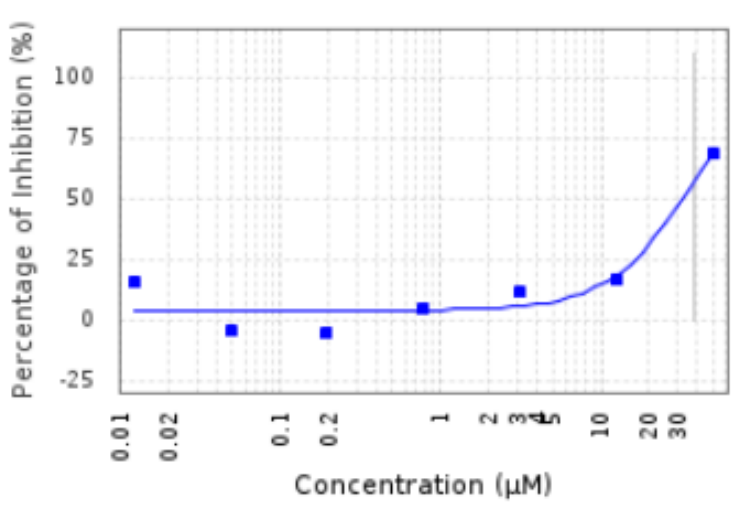

IC50: 38.319 (0.0025 - 576267) pass
slope=1.638 min=3.717 max=110.000 err=329.310 R^2=0.9133

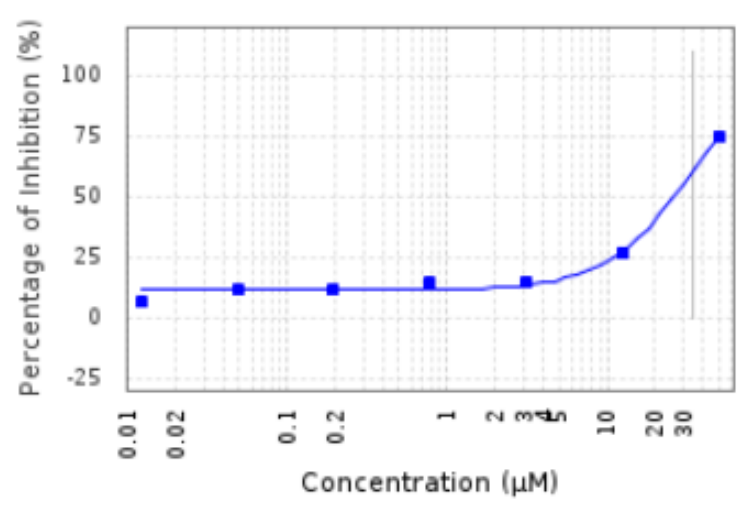

IC50: 34.646 (2.8838 - 416.23) pass
slope=1.597 min=11.161 max=110.000 err=36.100 R^2=0.9893

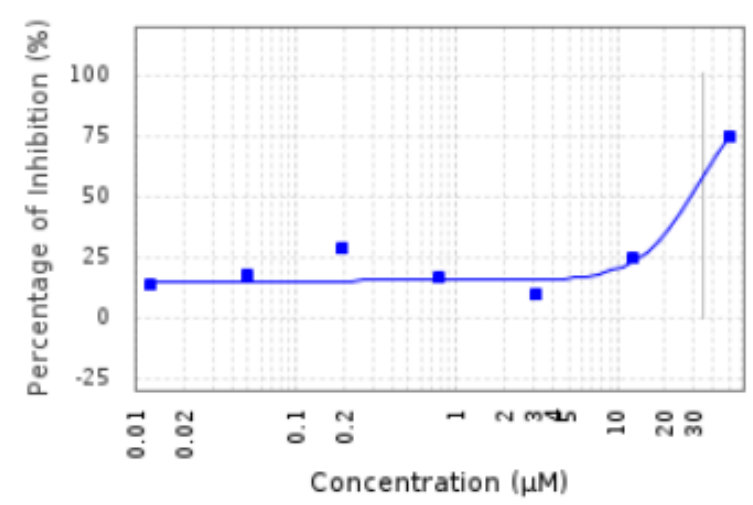

IC50: 34.517 (5.16468E-7 - 2.30685E9) pass
slope=2.249 min=15.000 max=100.889 err=232.084 R^2=0.9226

U2OS (IC50 39 µM)

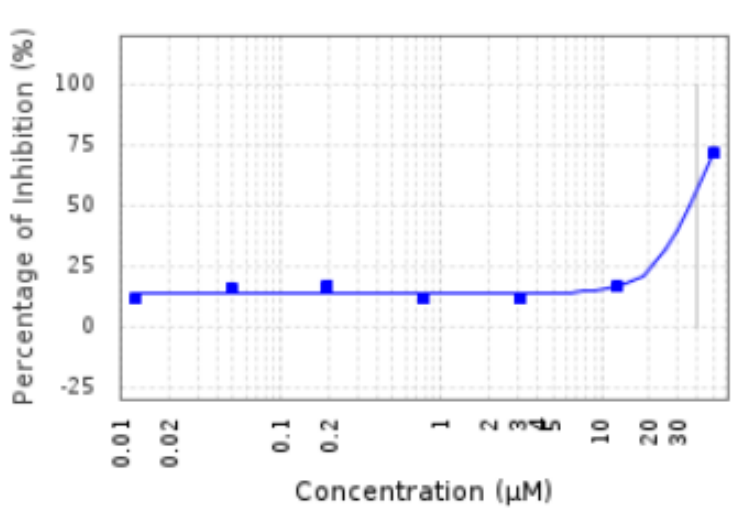

IC50: 39.752 (2.13455E-20 - 7.40297E22) pass
slope=3.019 min=13.548 max=100.000 err=29.527 R^2=0.9896

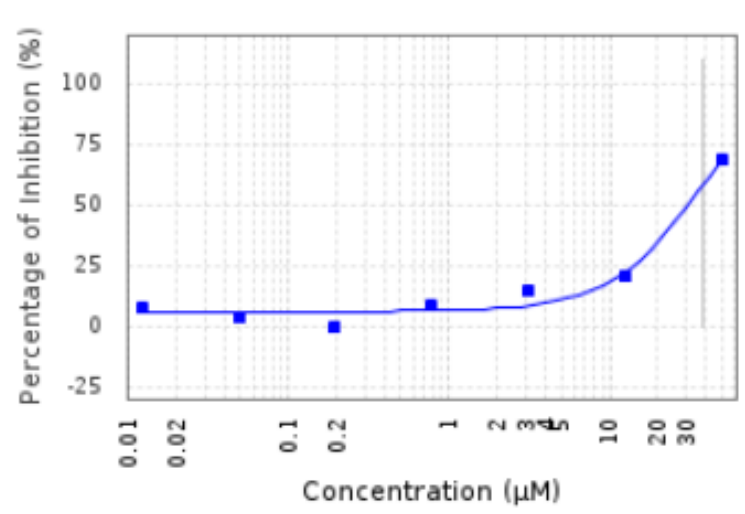

IC50: 38.297 (0.4903 - 2991.3) pass
slope=1.500 min=5.859 max=110.000 err=90.993 R^2=0.9726

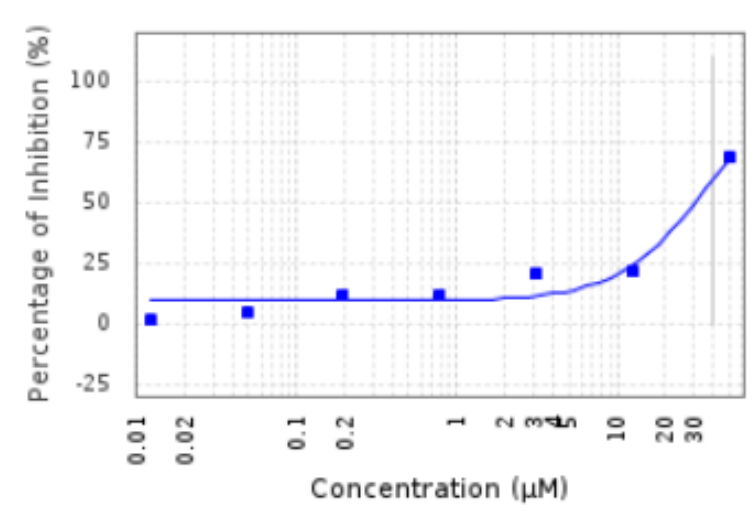

IC50: 40.002 (0.0243 - 65942) pass
slope=1.500 min=9.012 max=110.000 err=199.043 R^2=0.9355

S5. Copies of NMR spectra

NMR spectra of **3a**

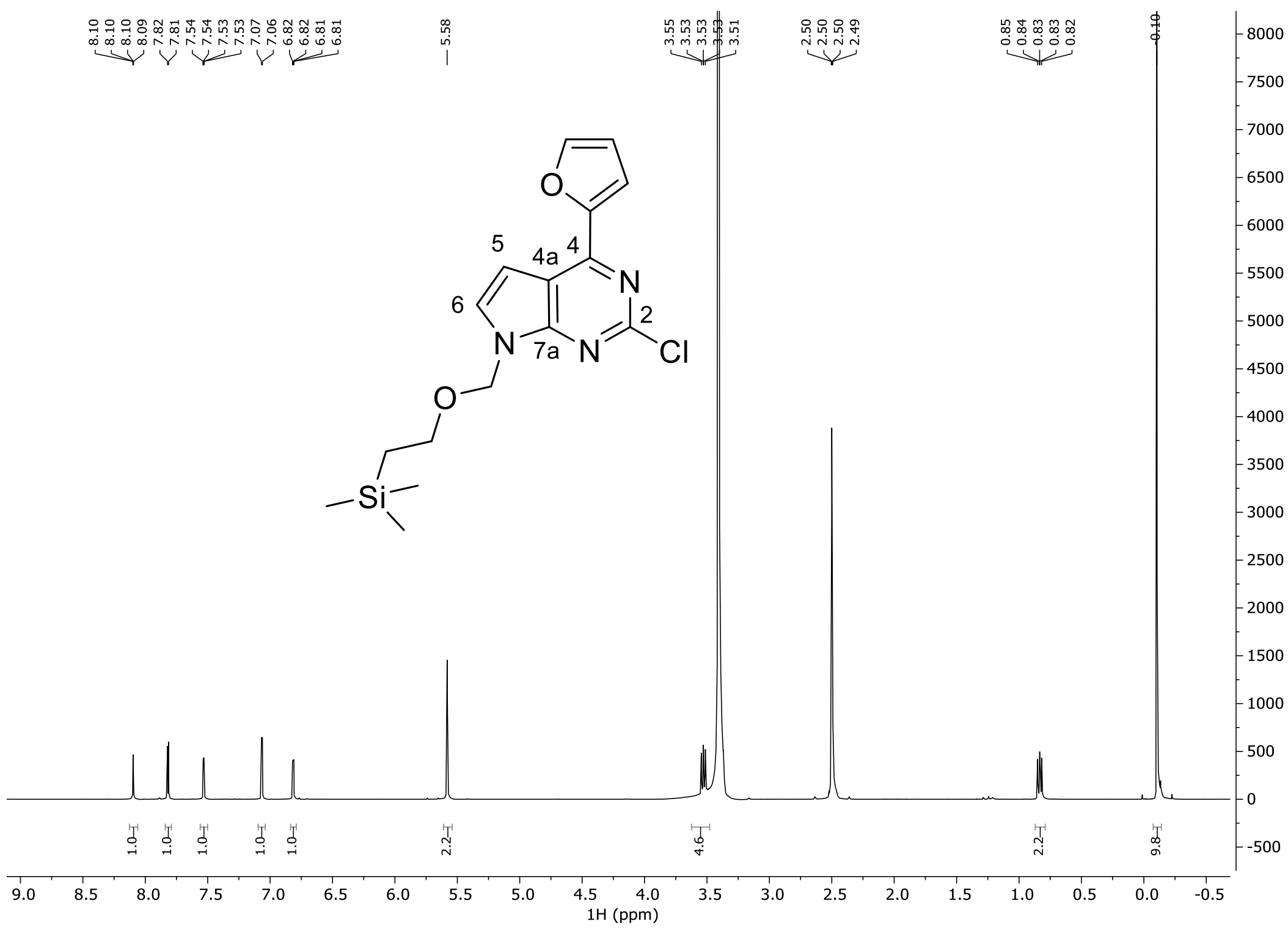

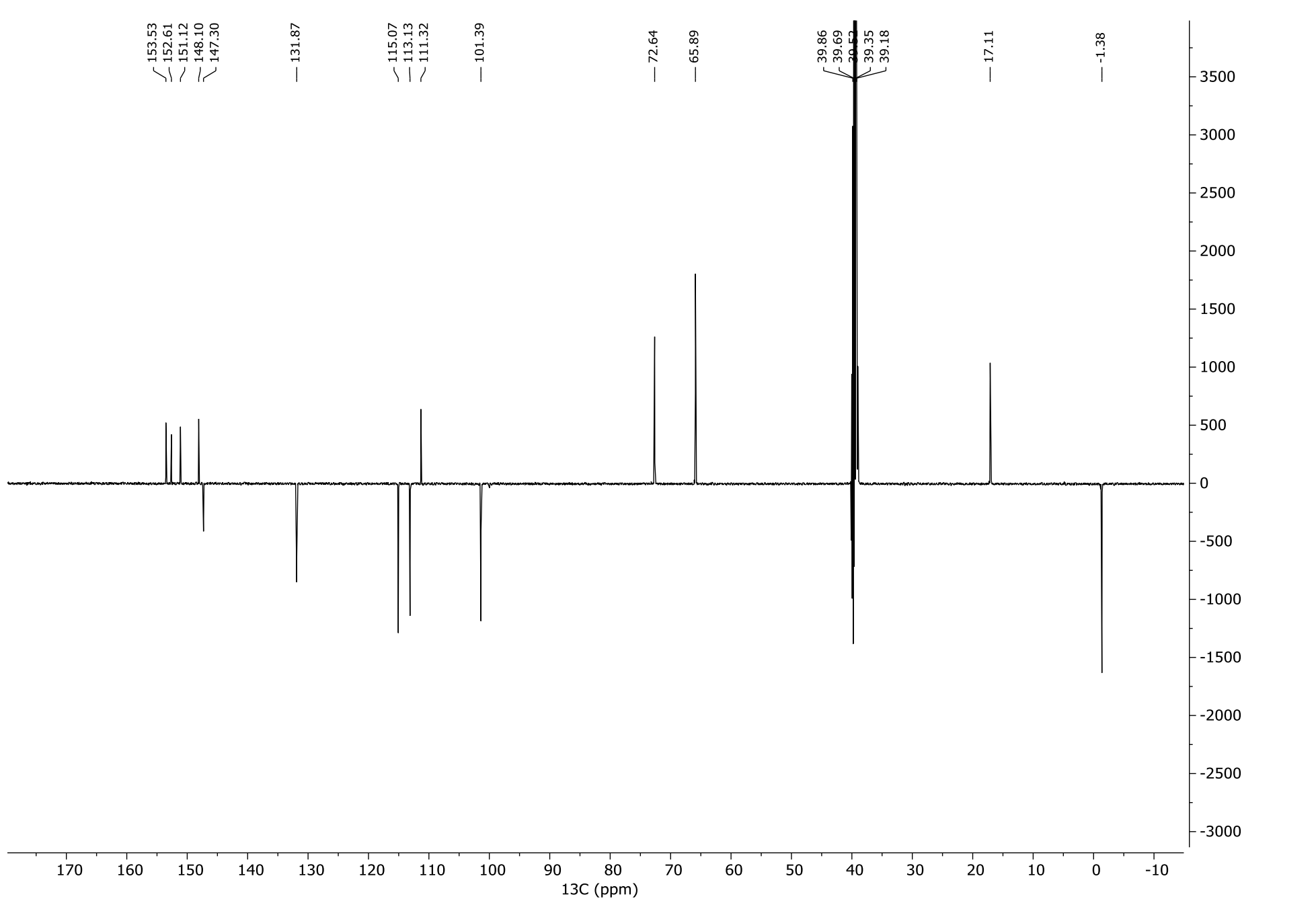

NMR spectra of **3b**

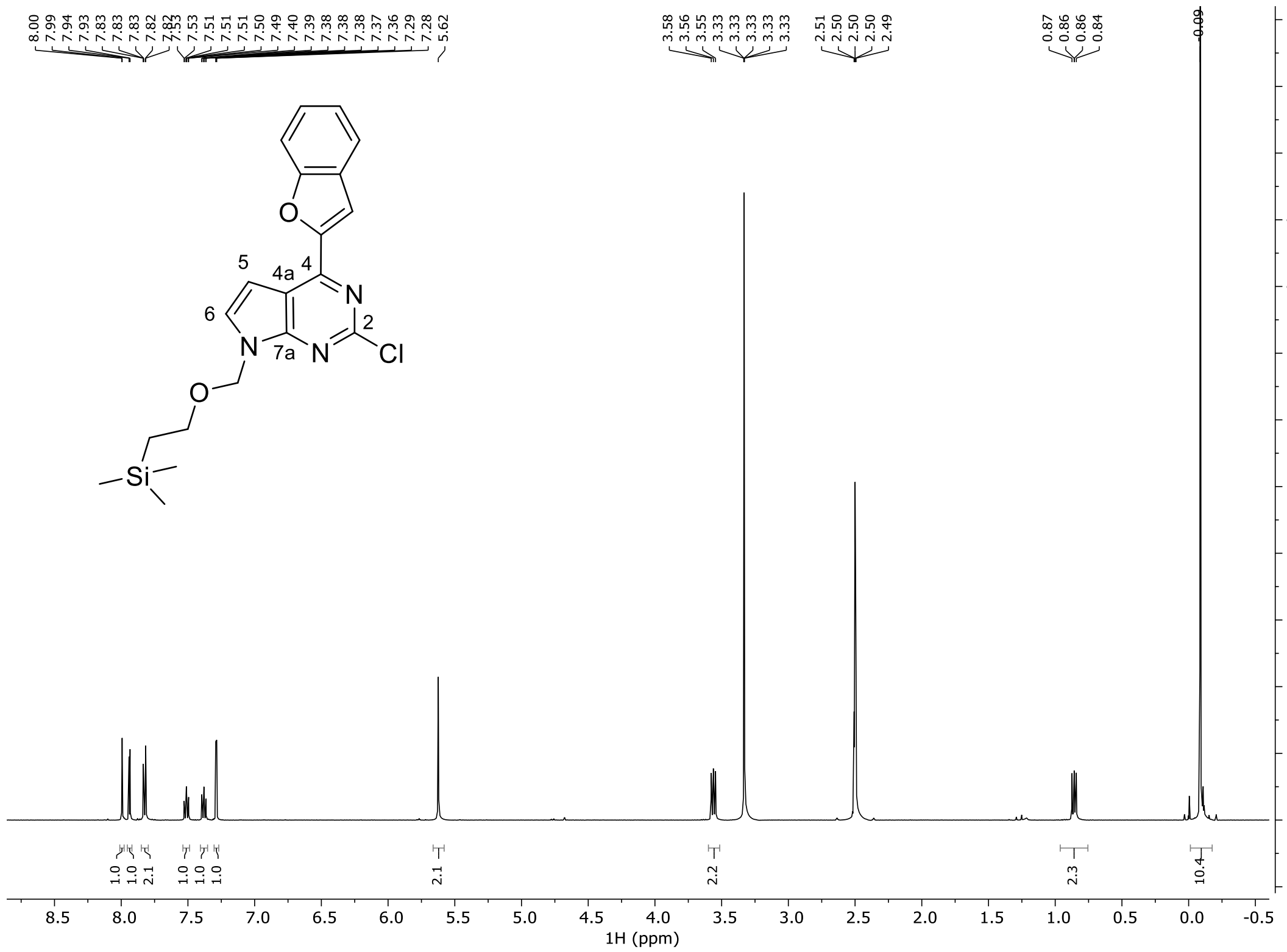

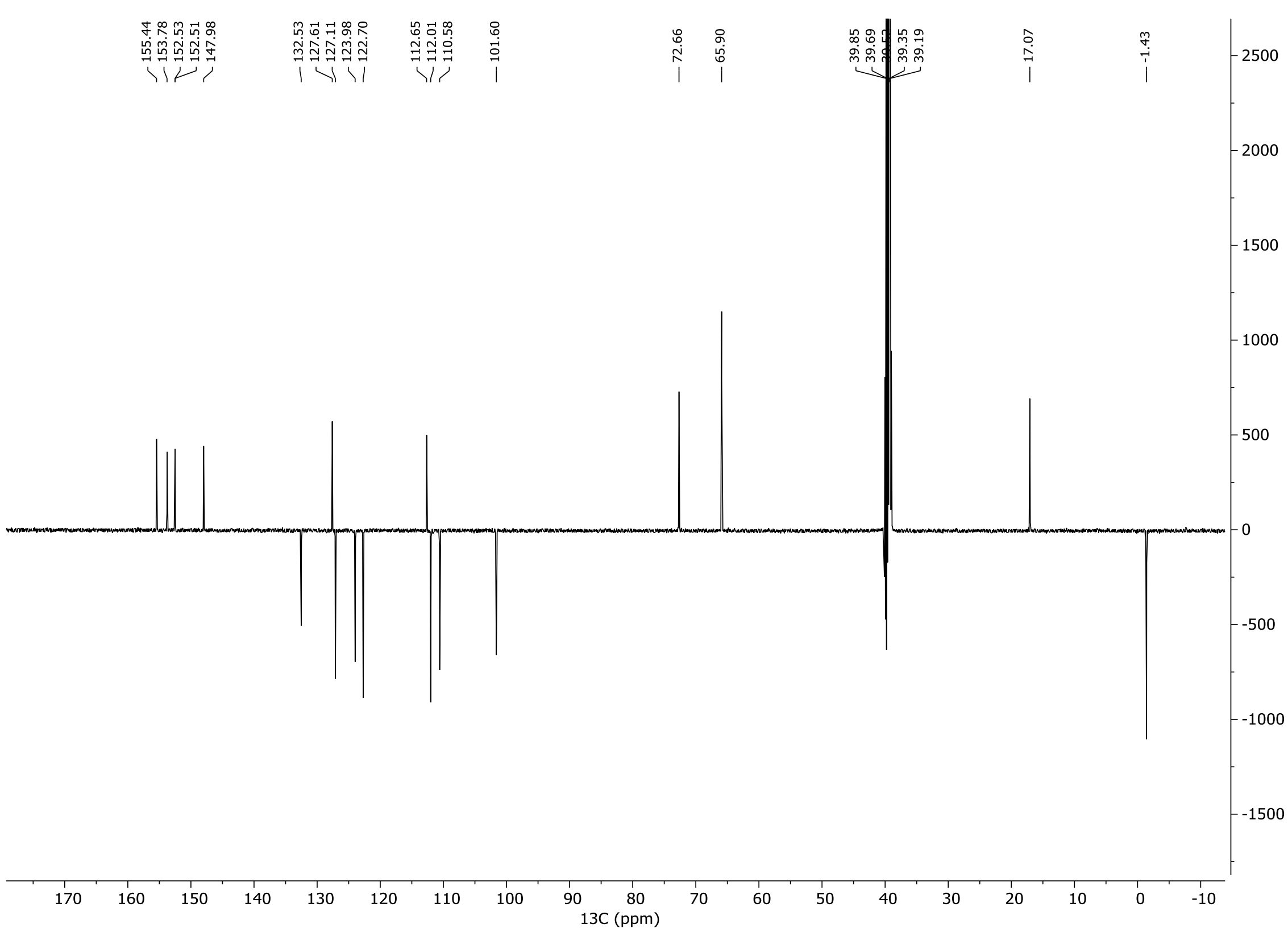

NMR spectra of **3c**

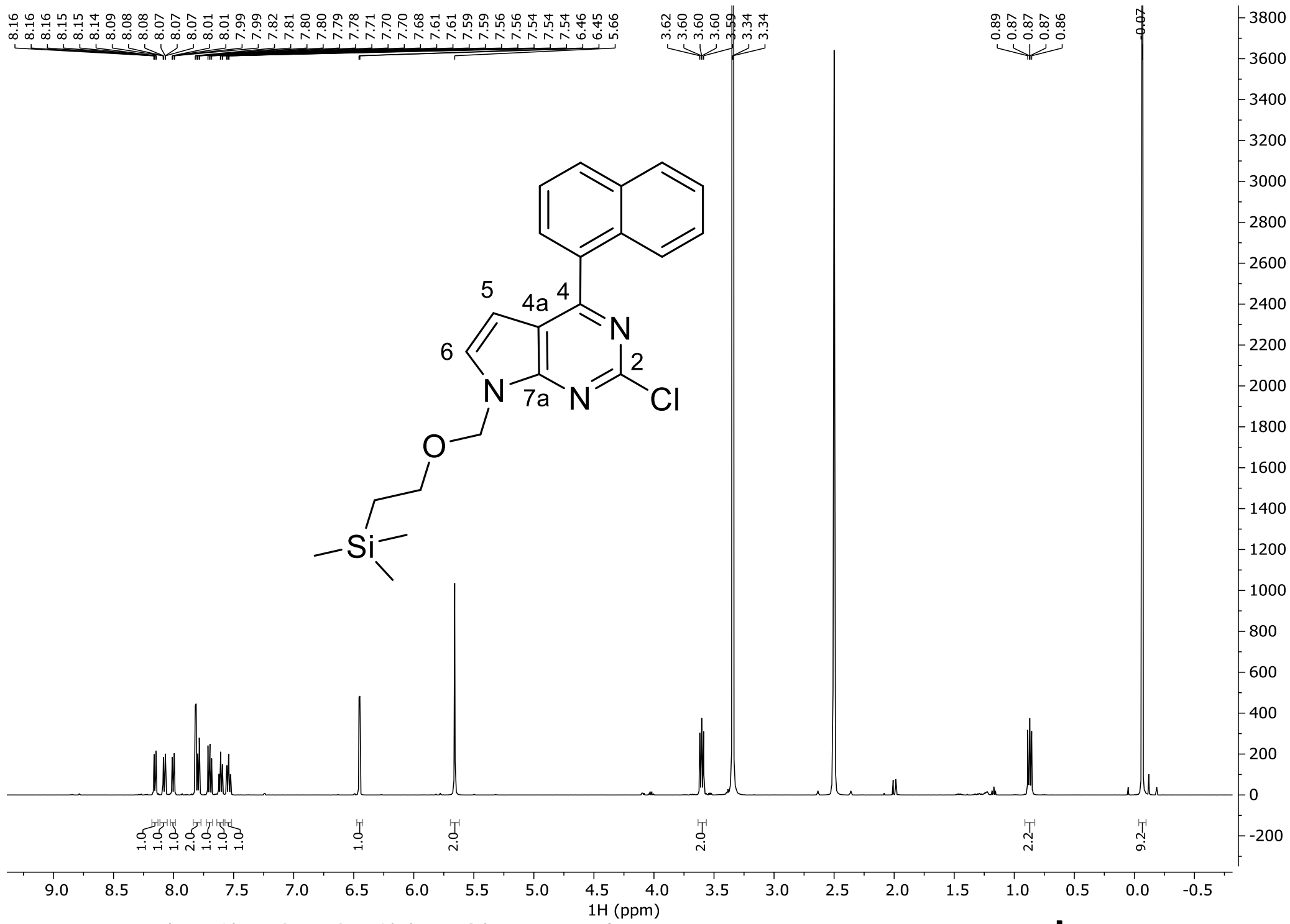

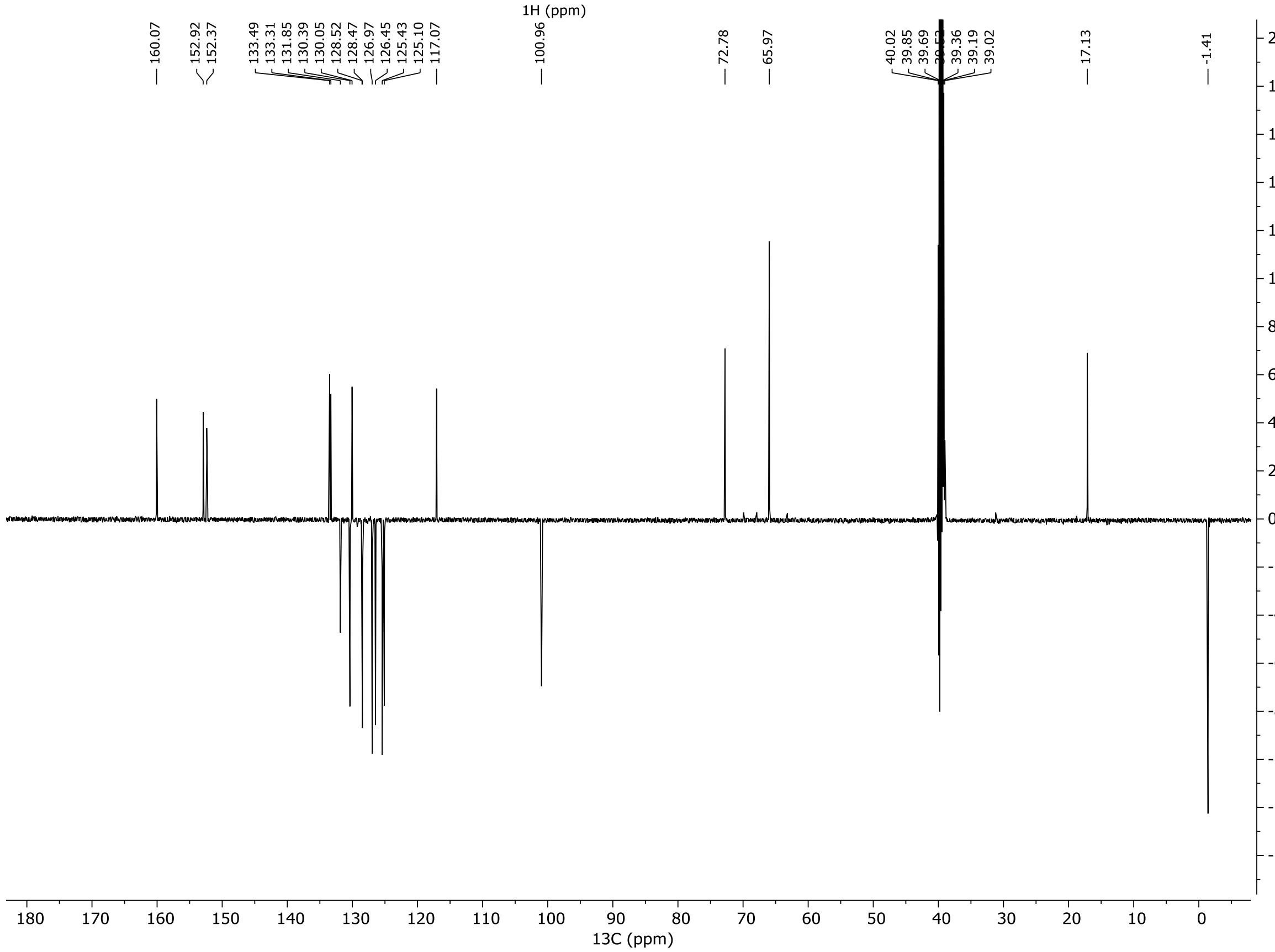

NMR spectra of **3d**

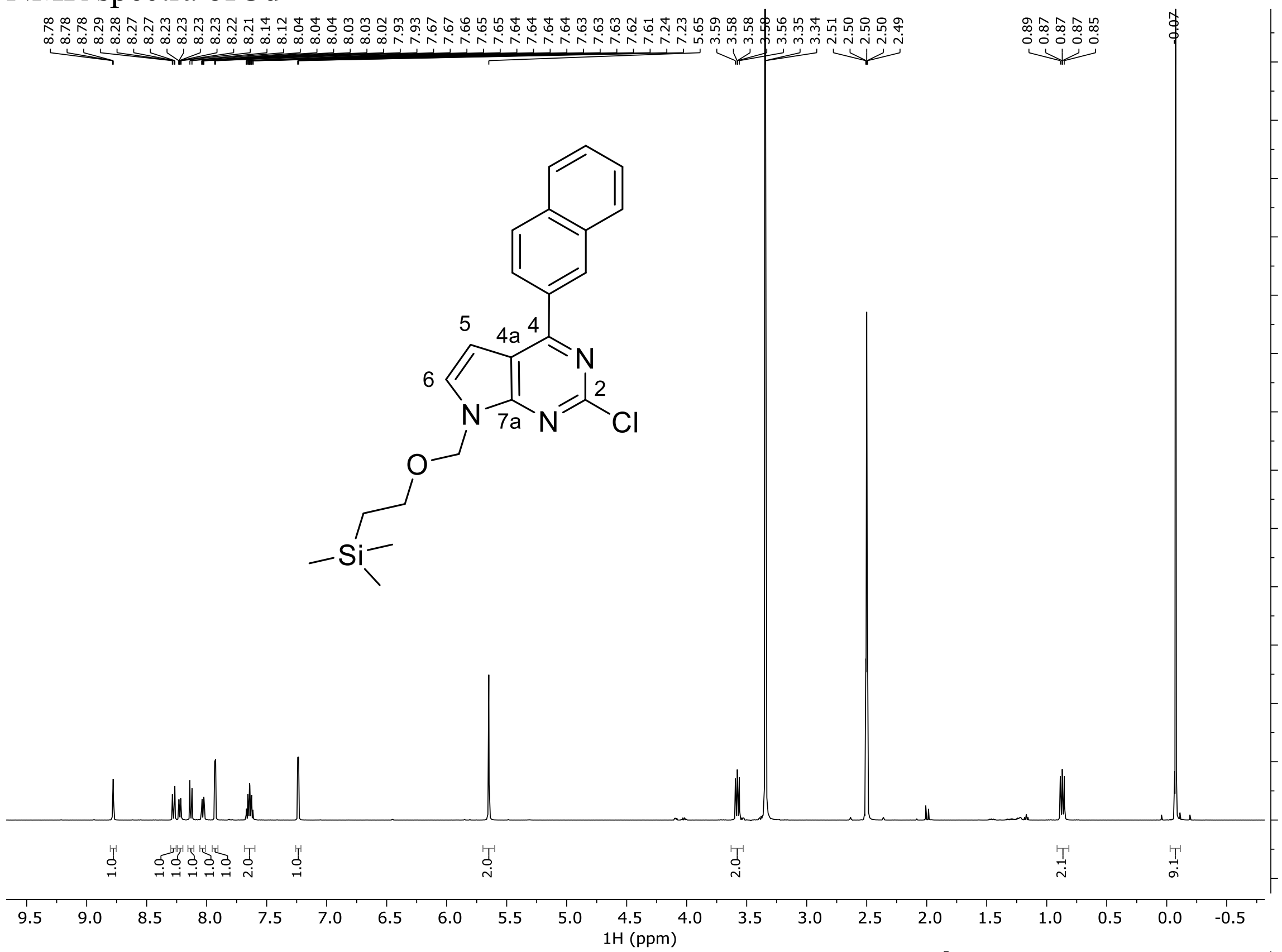

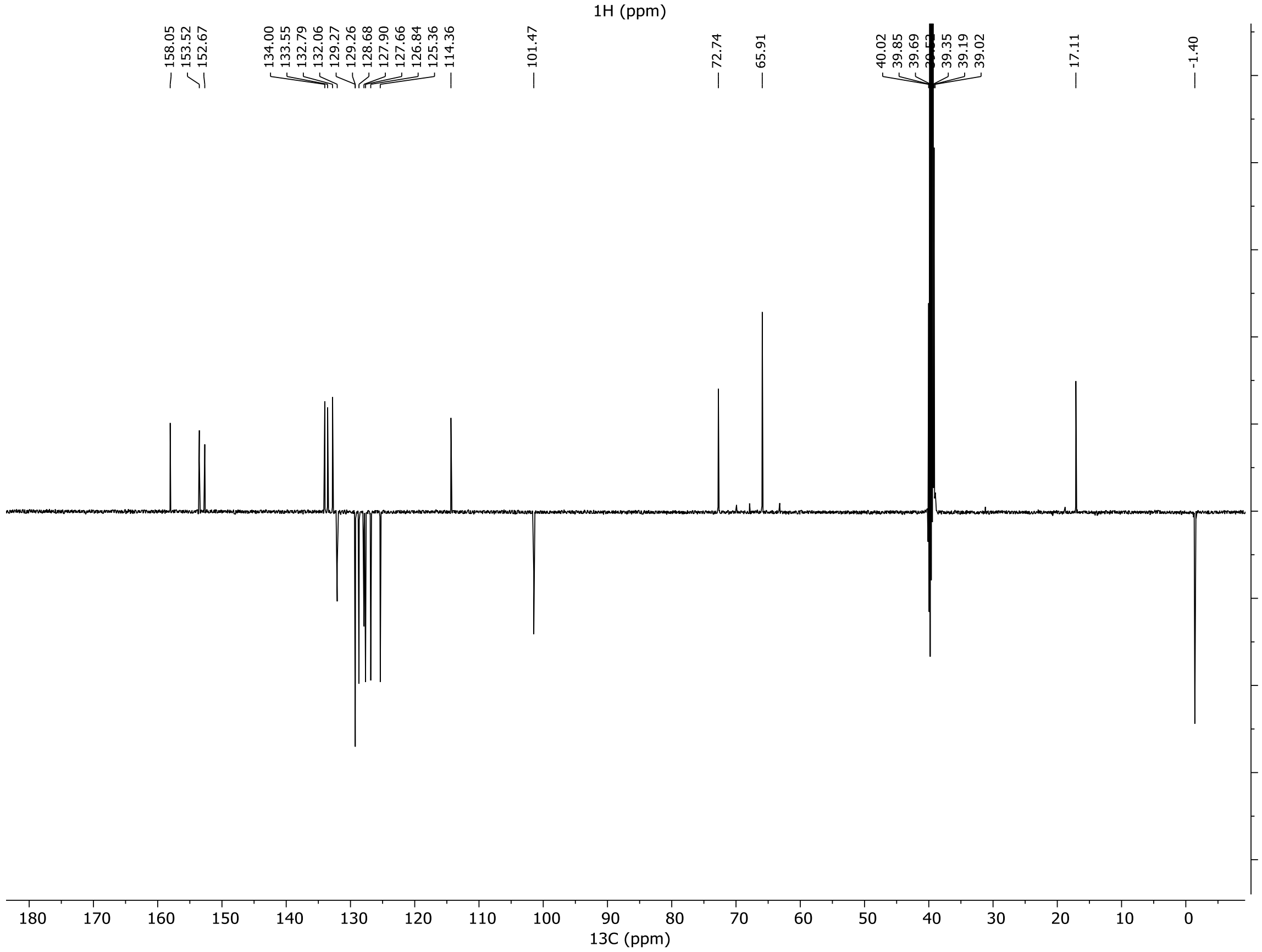

NMR spectra of **5aa**

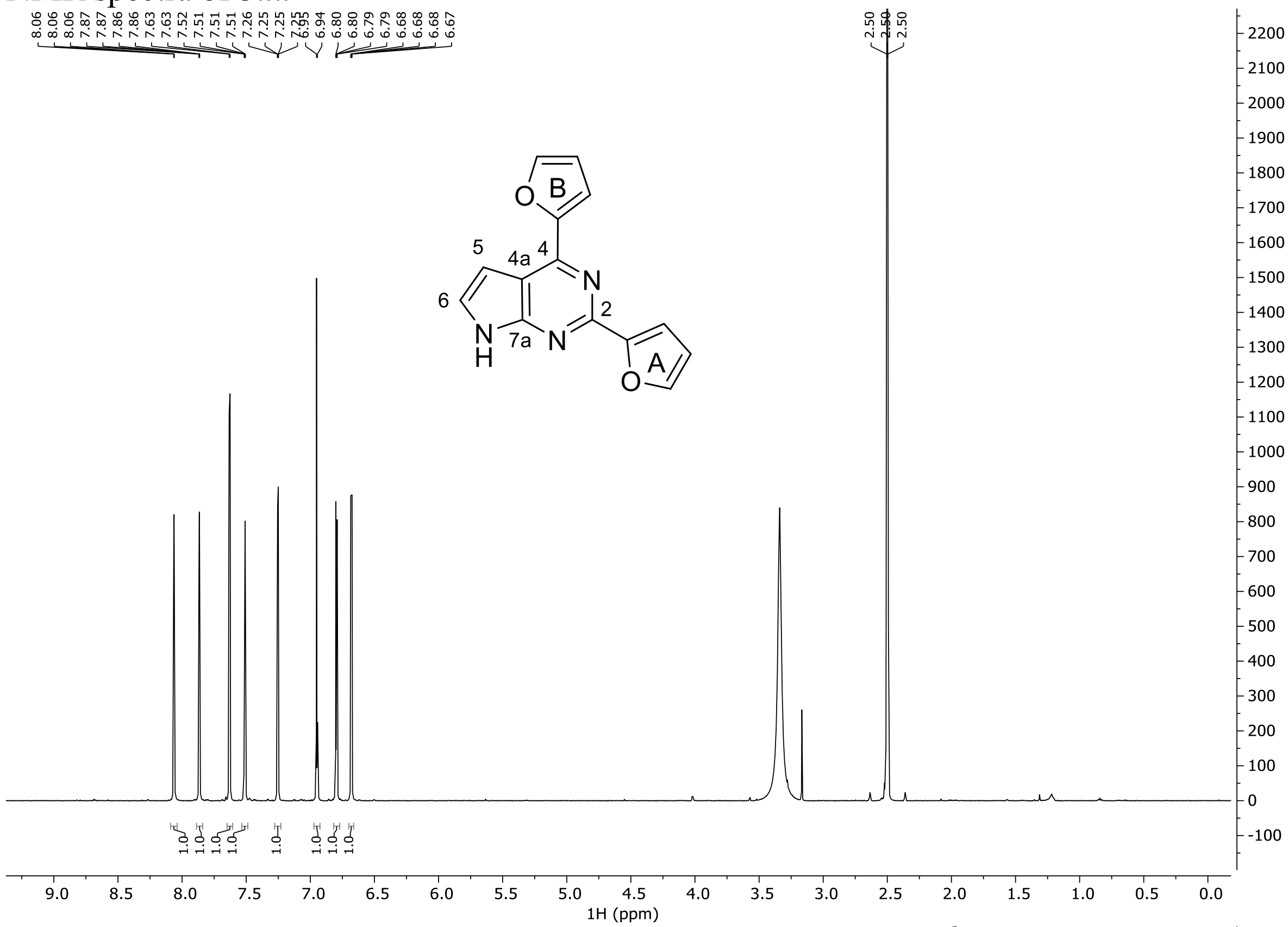

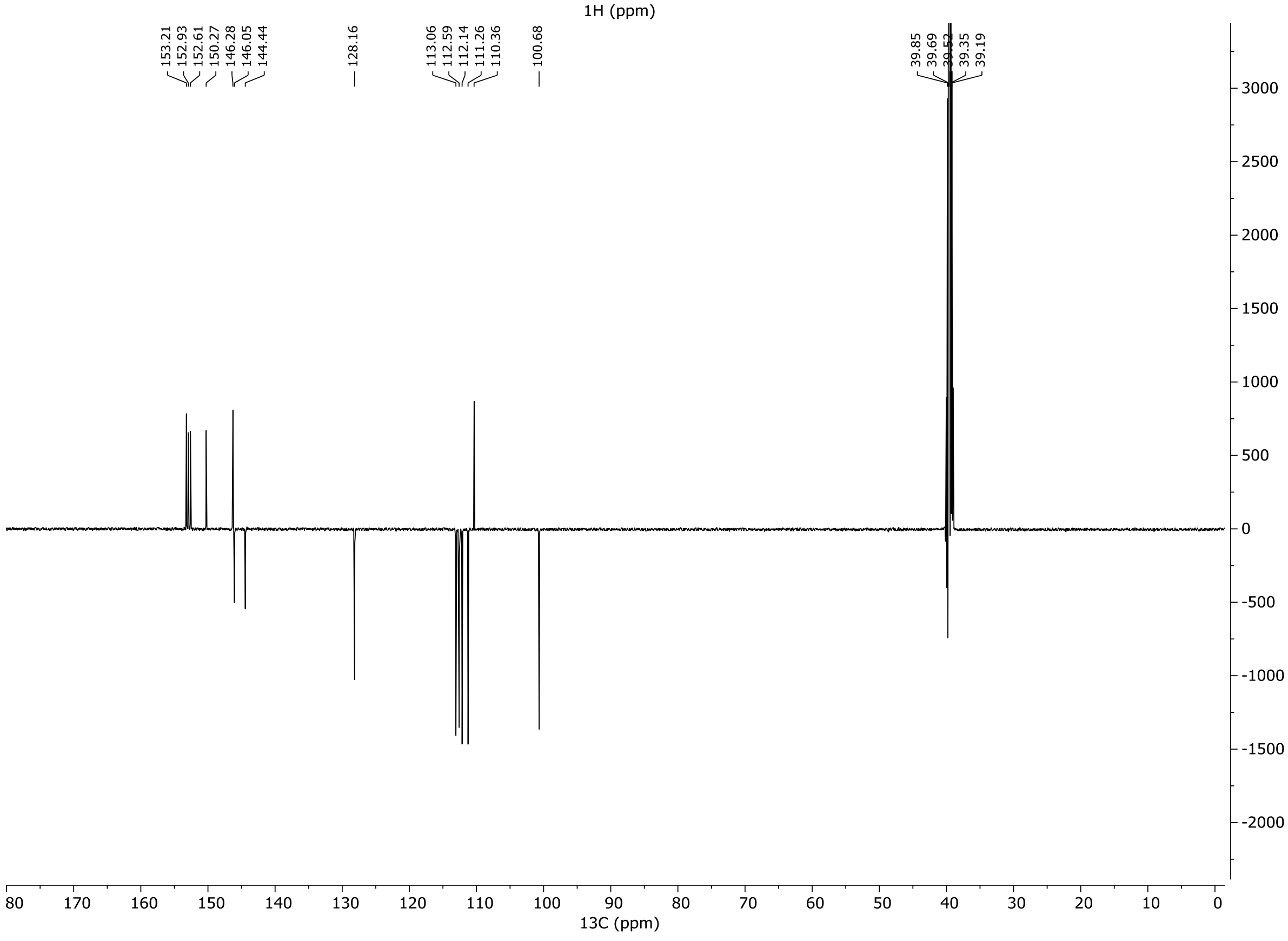

NMR spectra of **5ab**

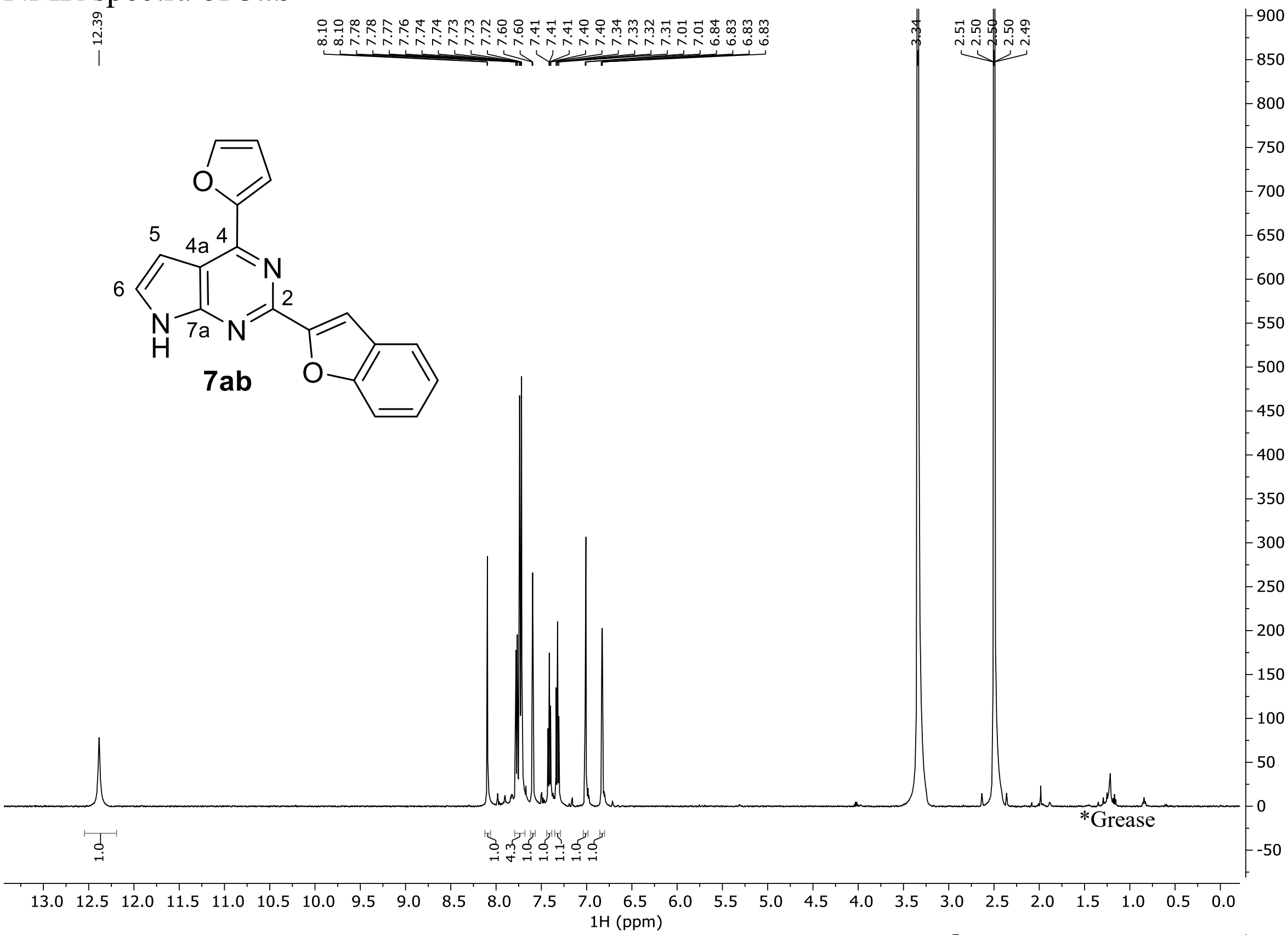

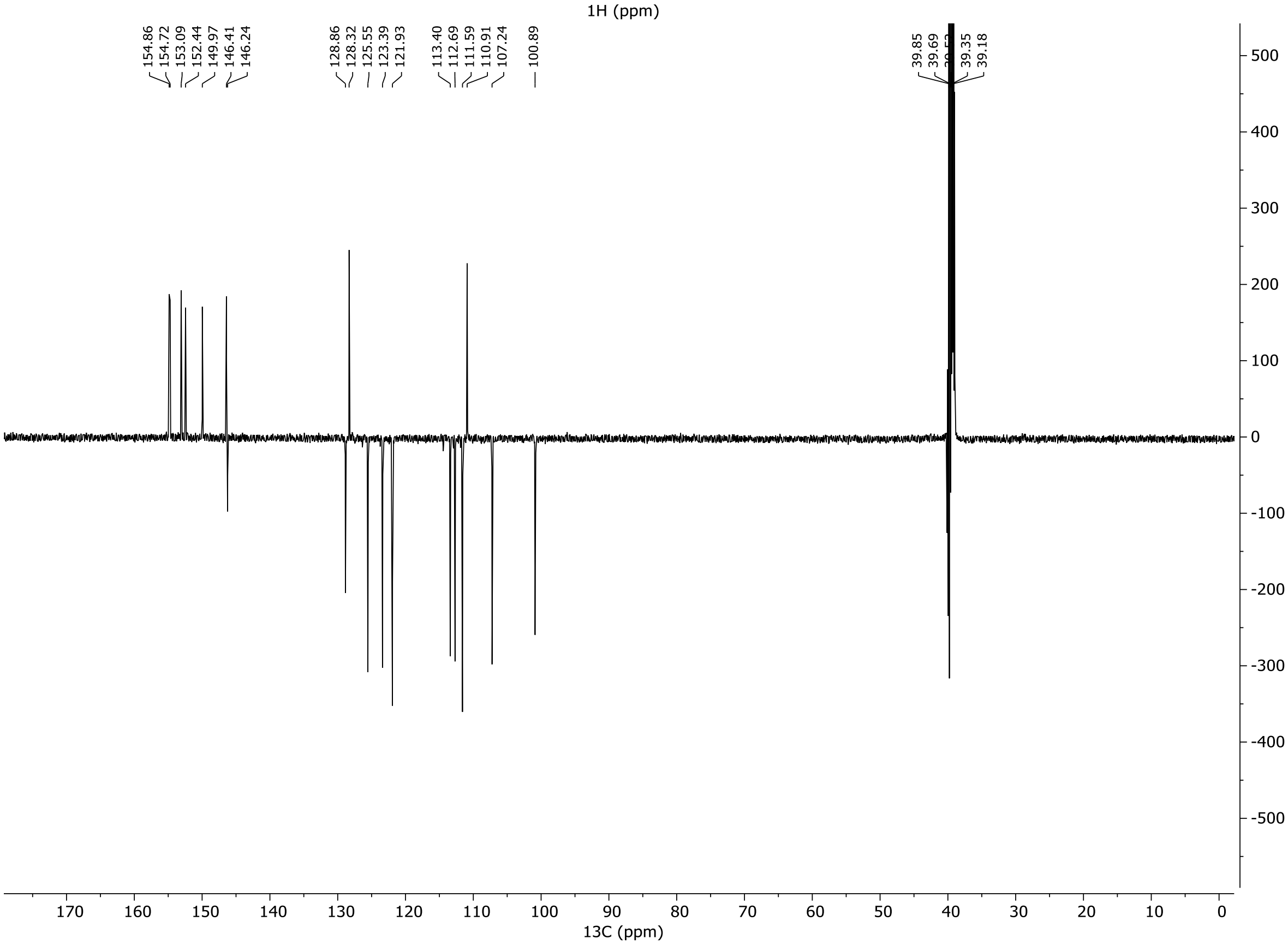

NMR spectra of **5ac**

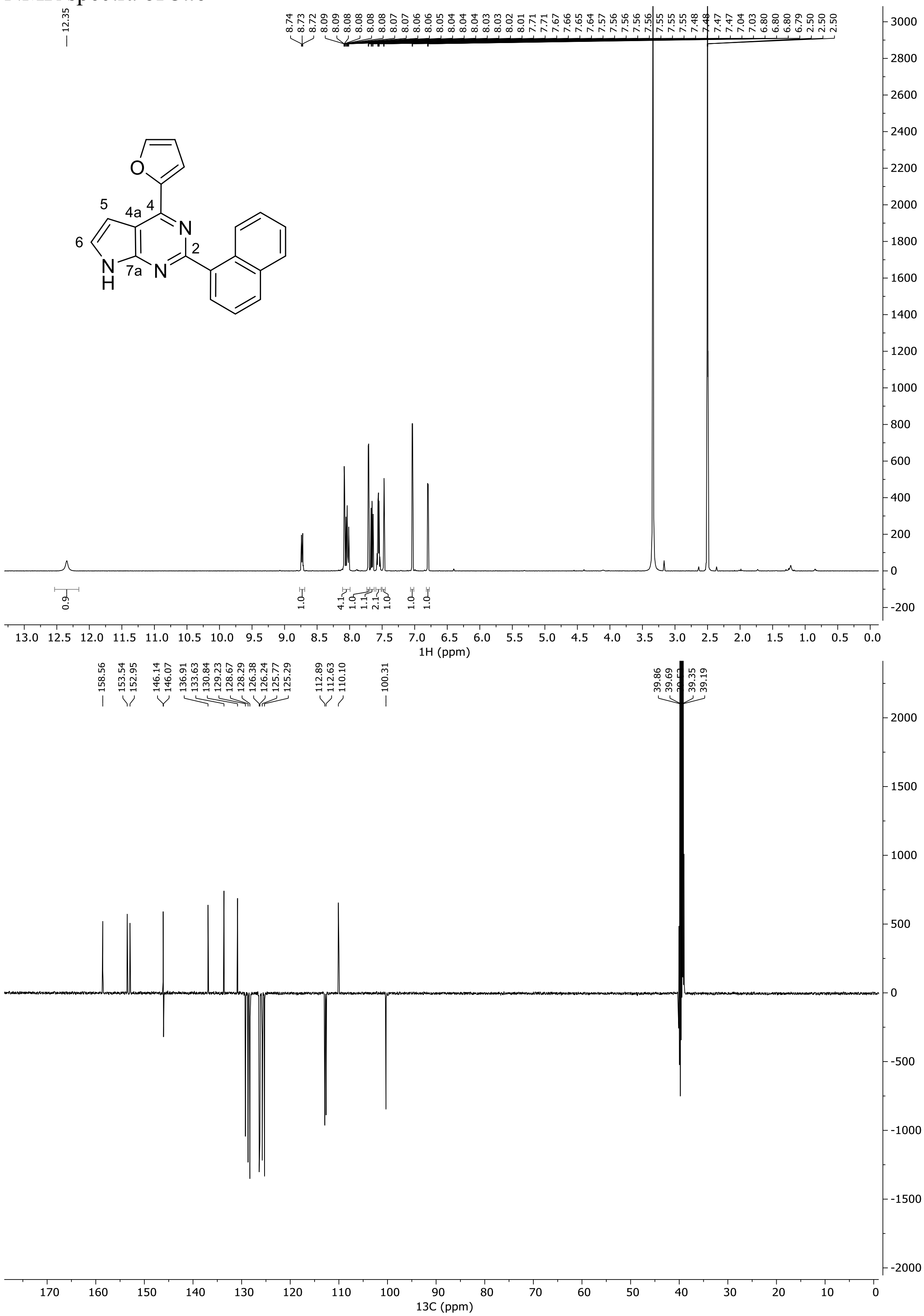

NMR spectra of **5ad**

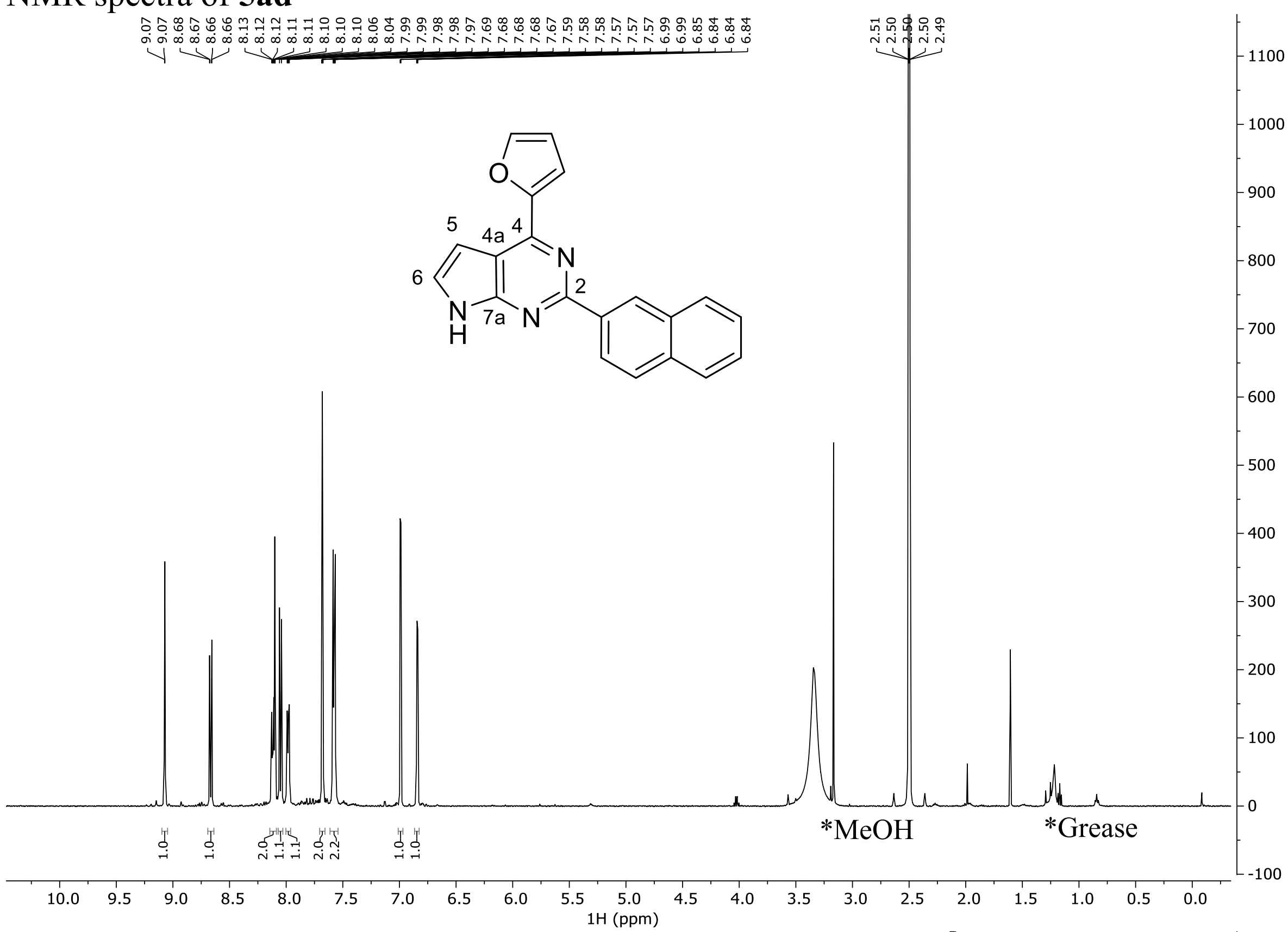

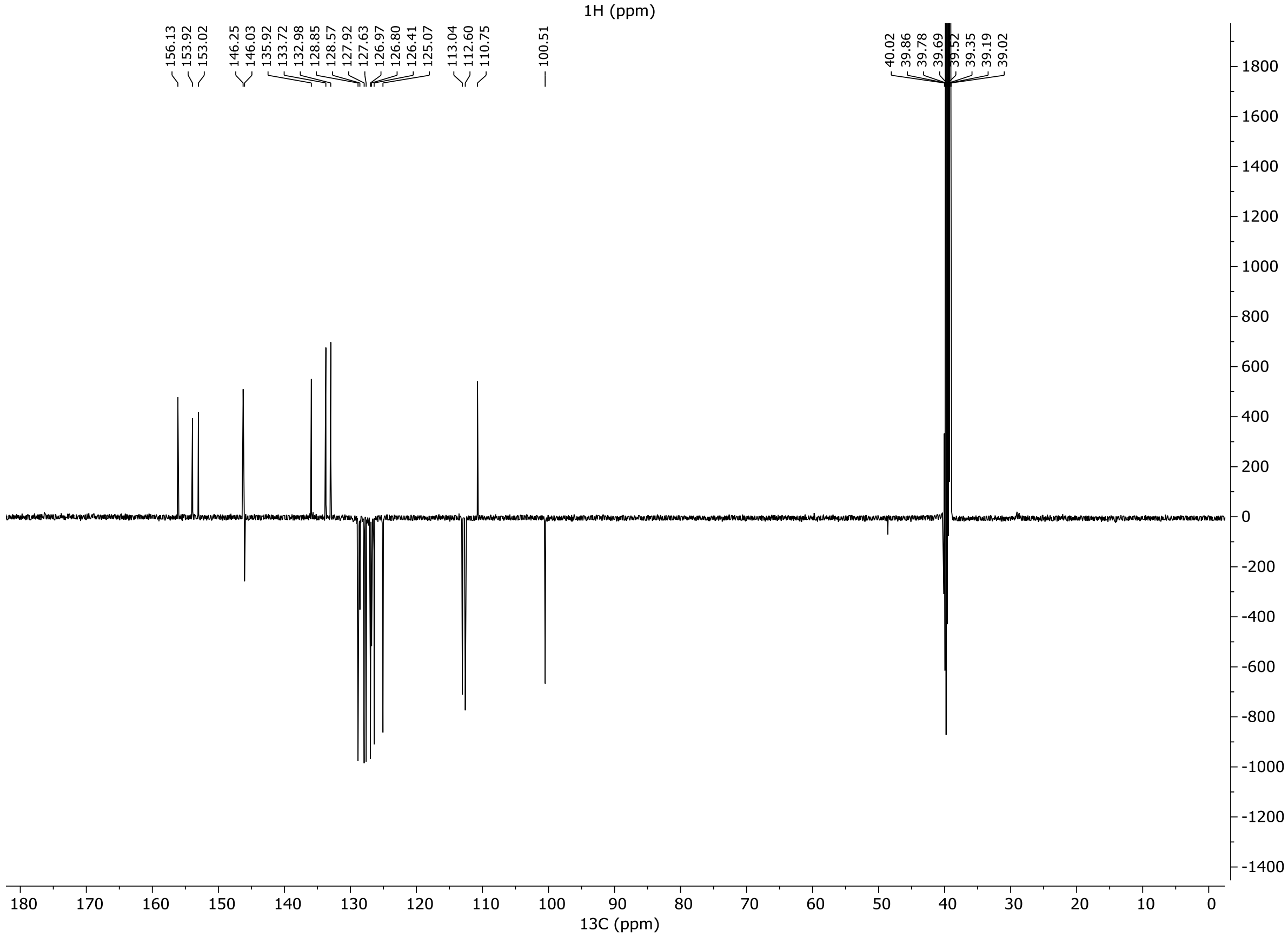

NMR spectra of **5ba**

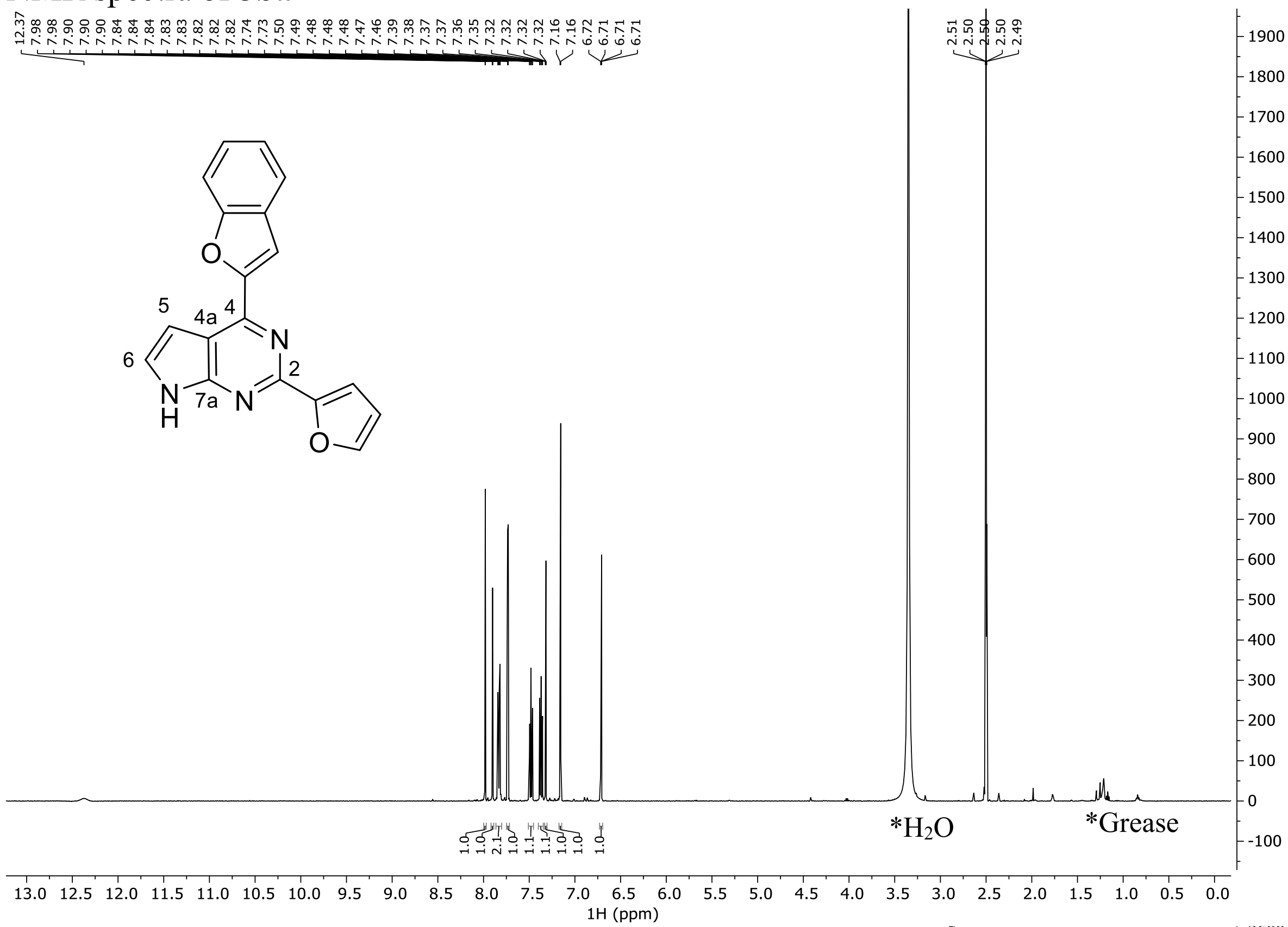

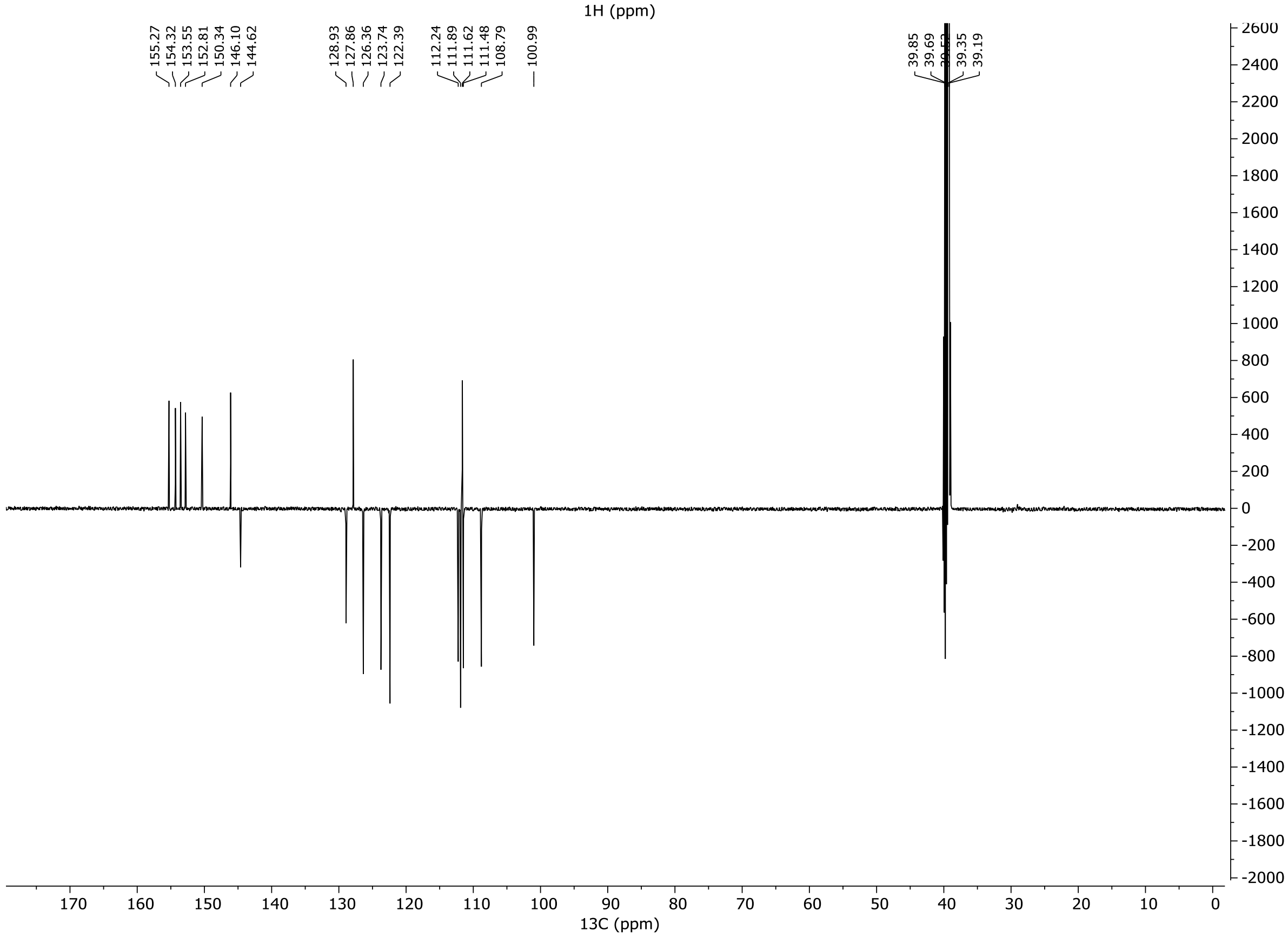

NMR spectra of **5bb**

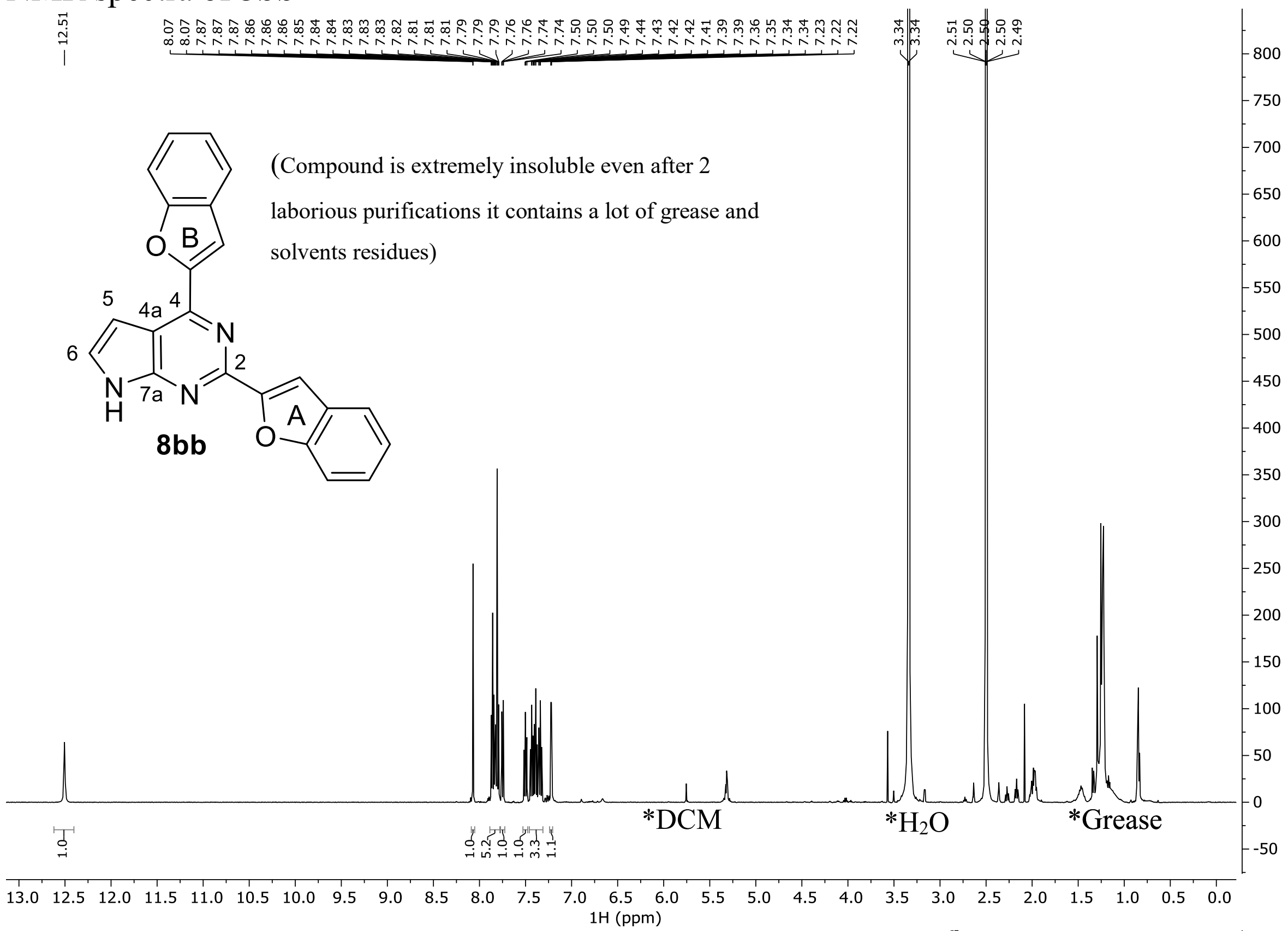

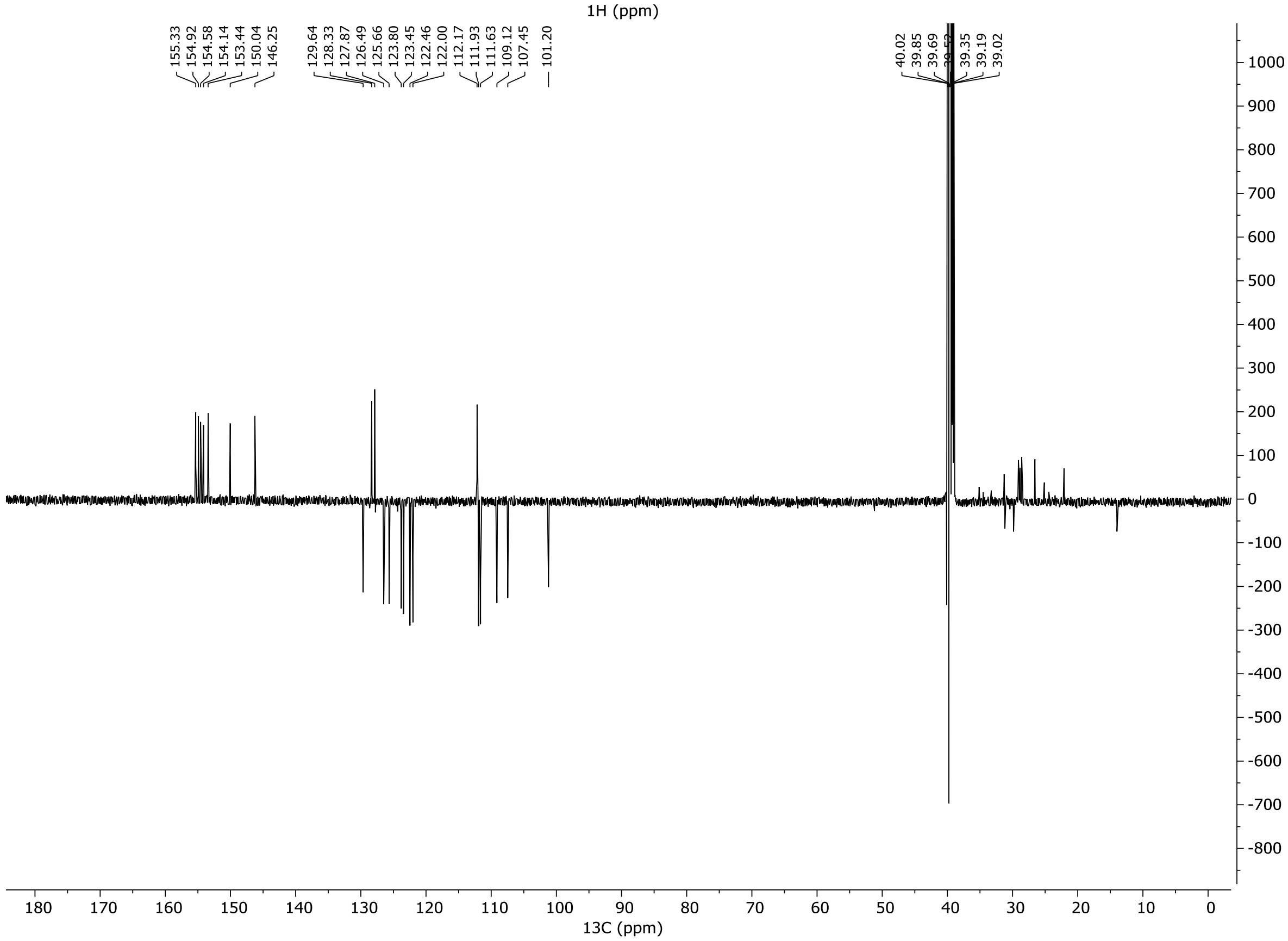

NMR spectra of **5bc**

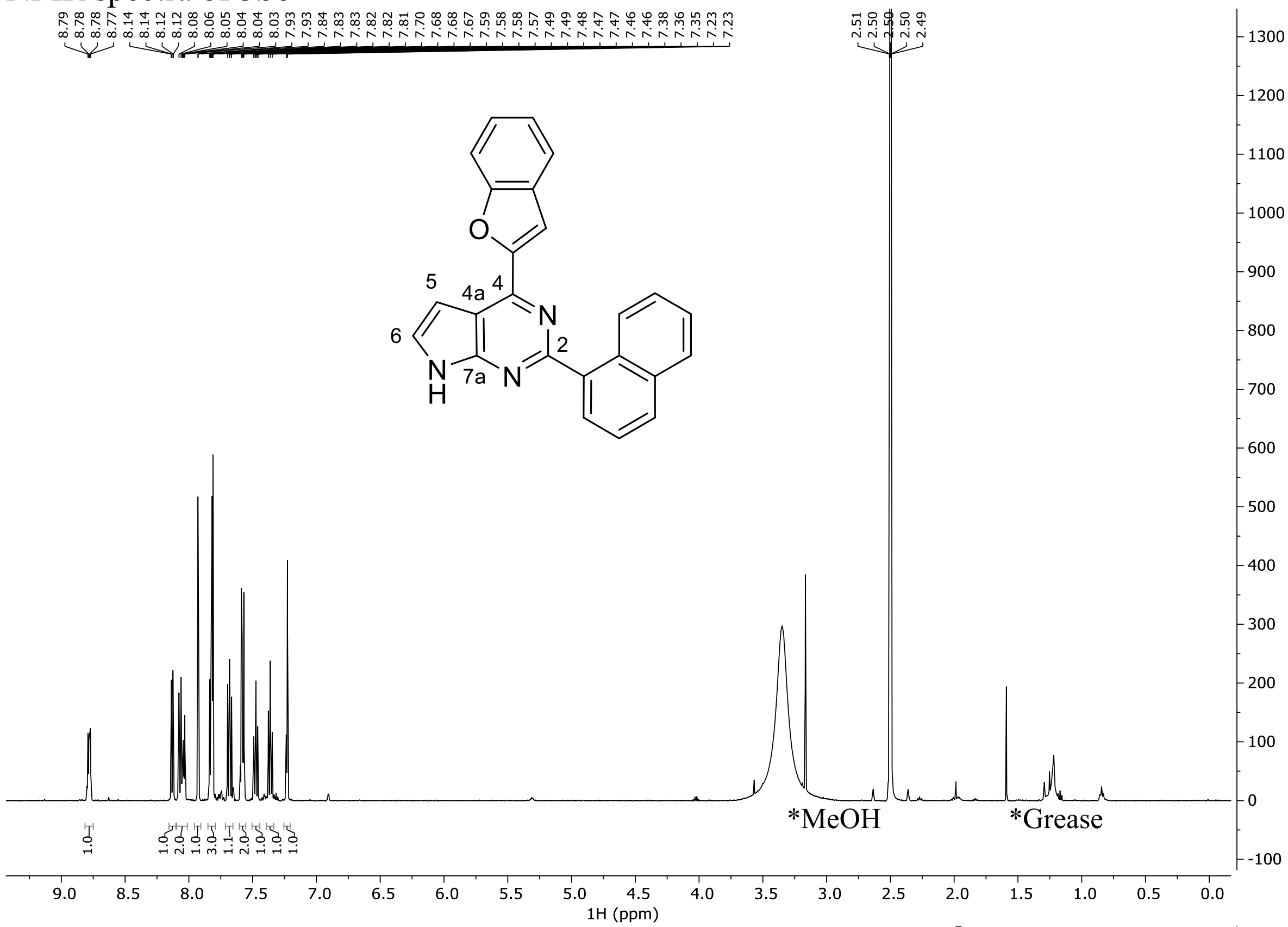

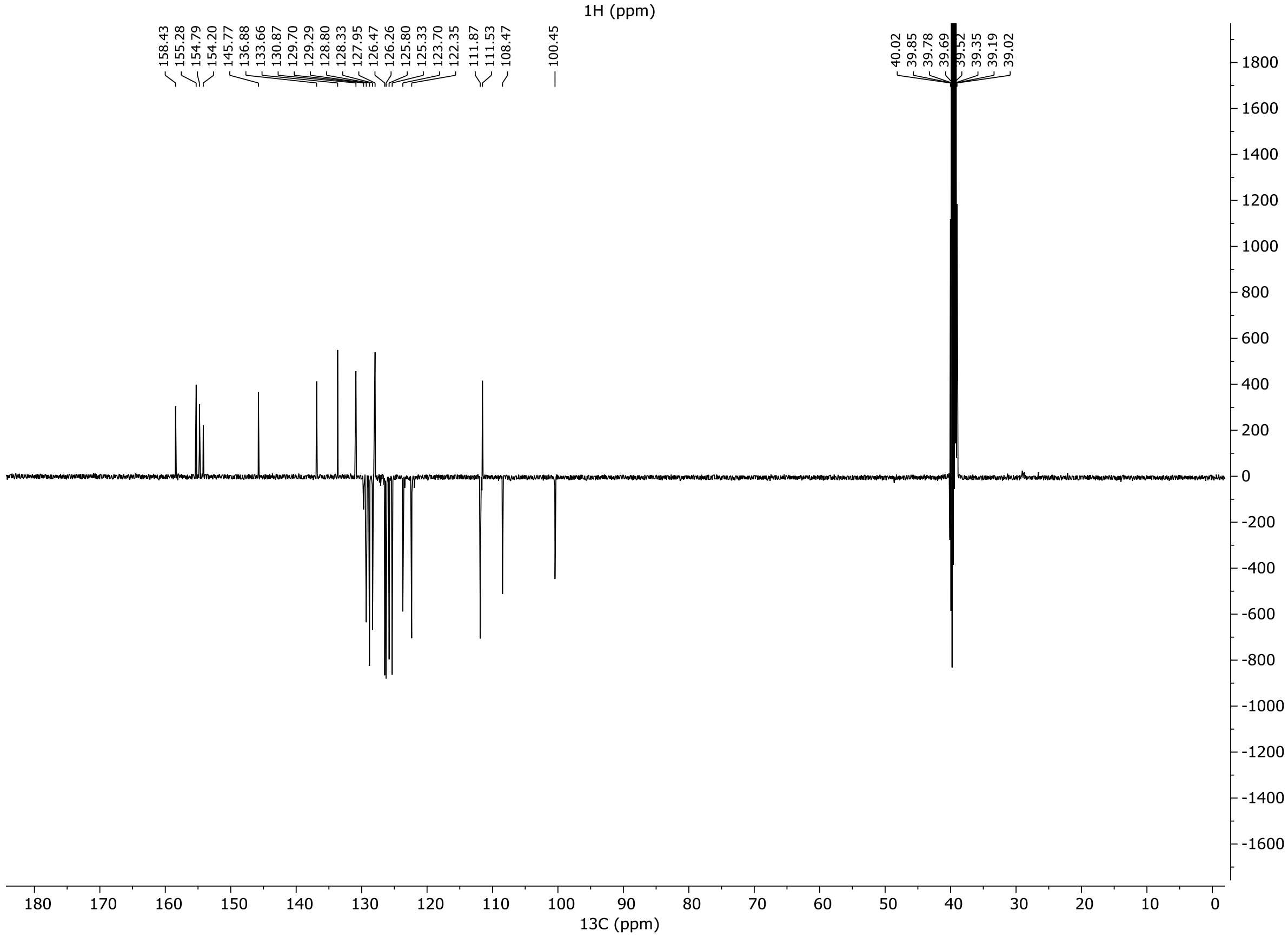

NMR spectra of **5bd**

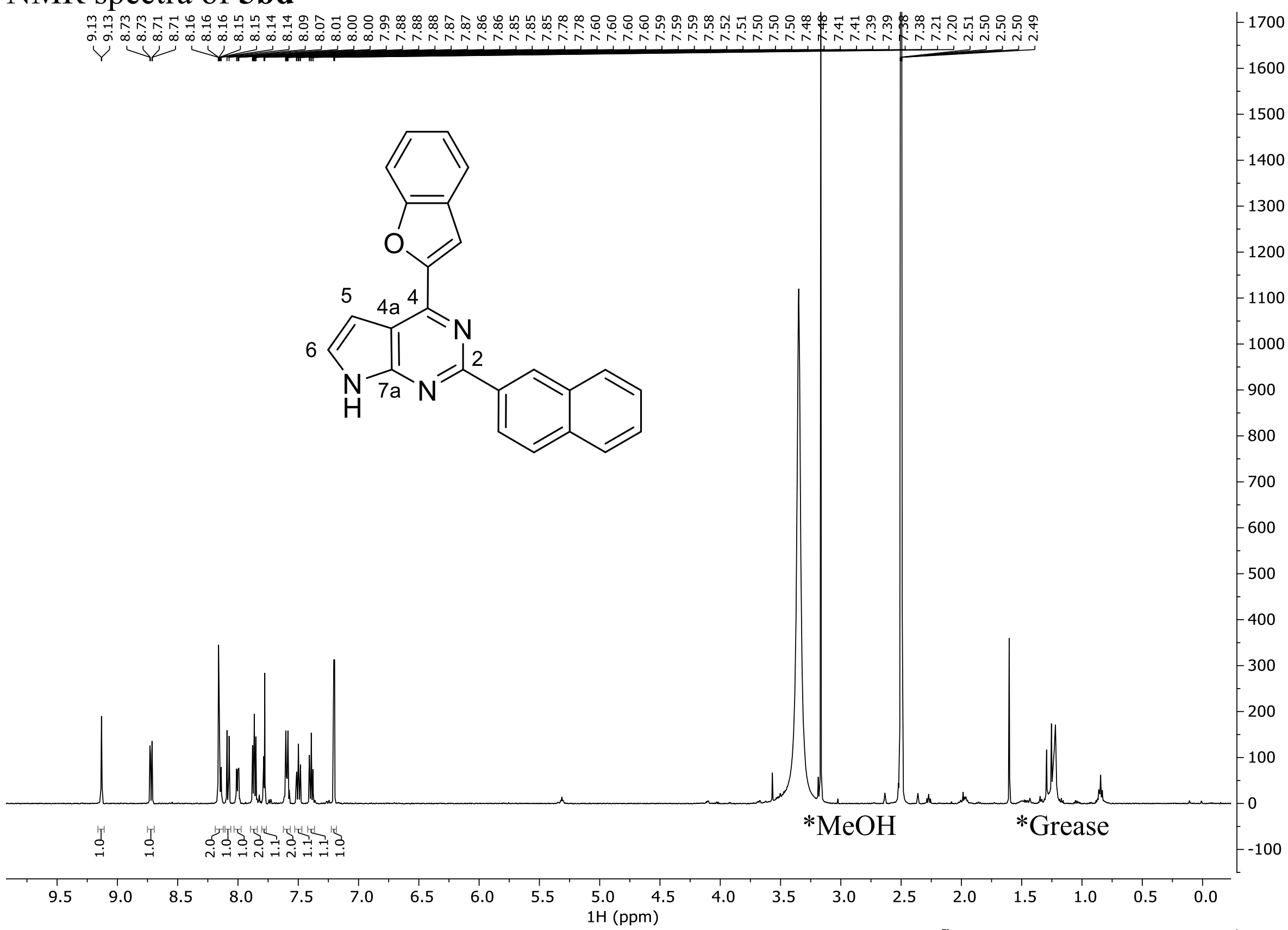

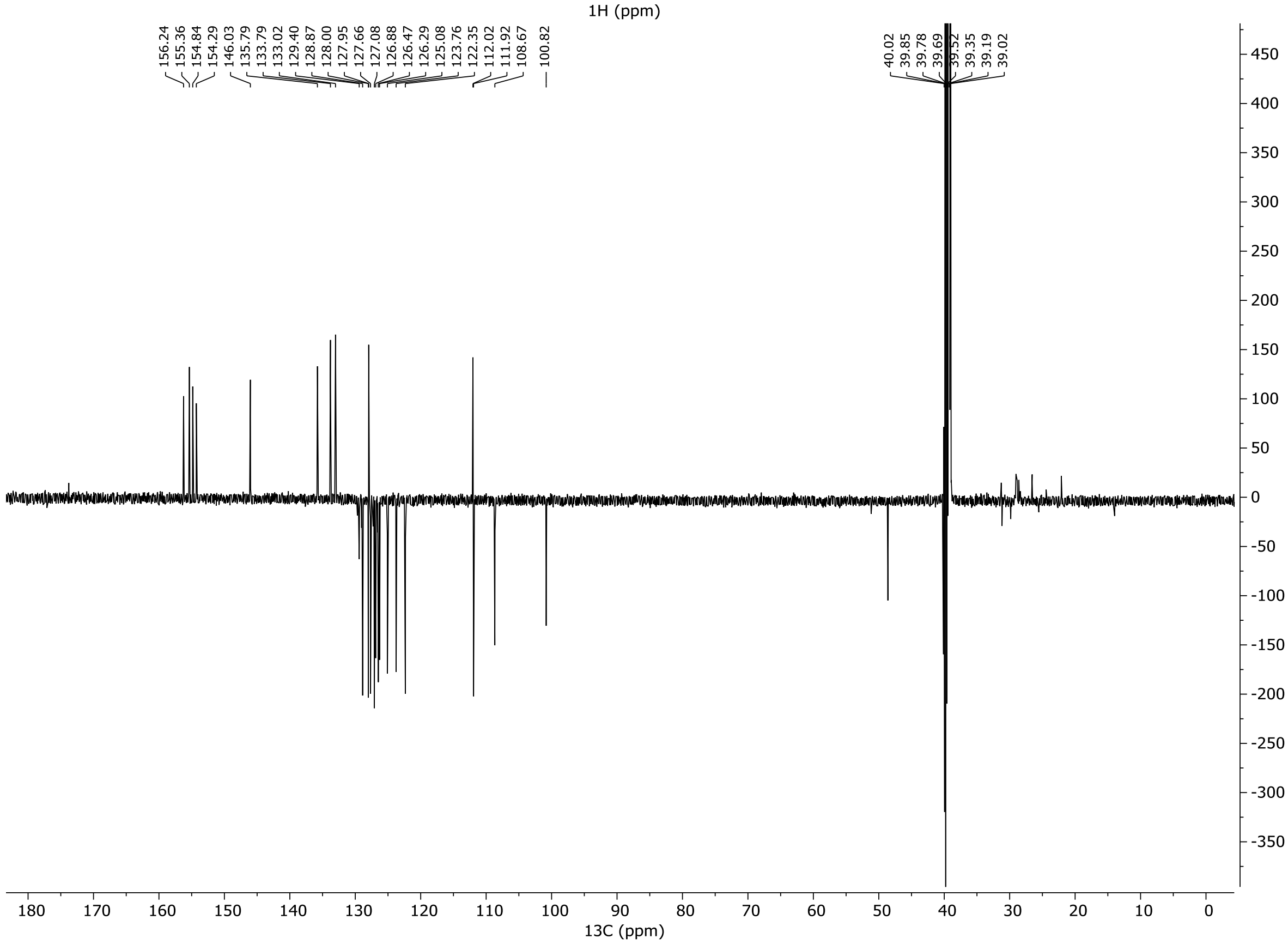

NMR spectra of **5ca**

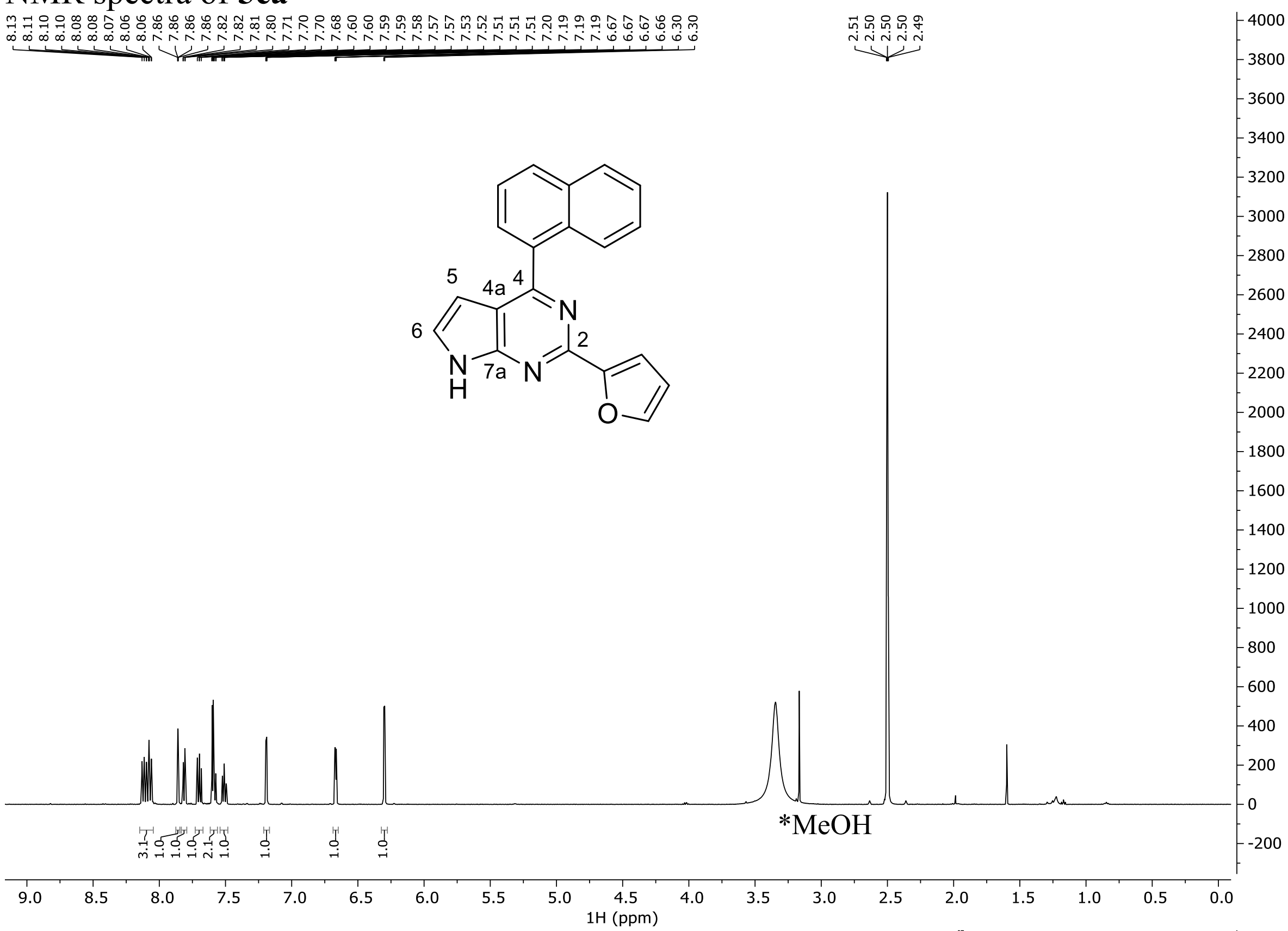

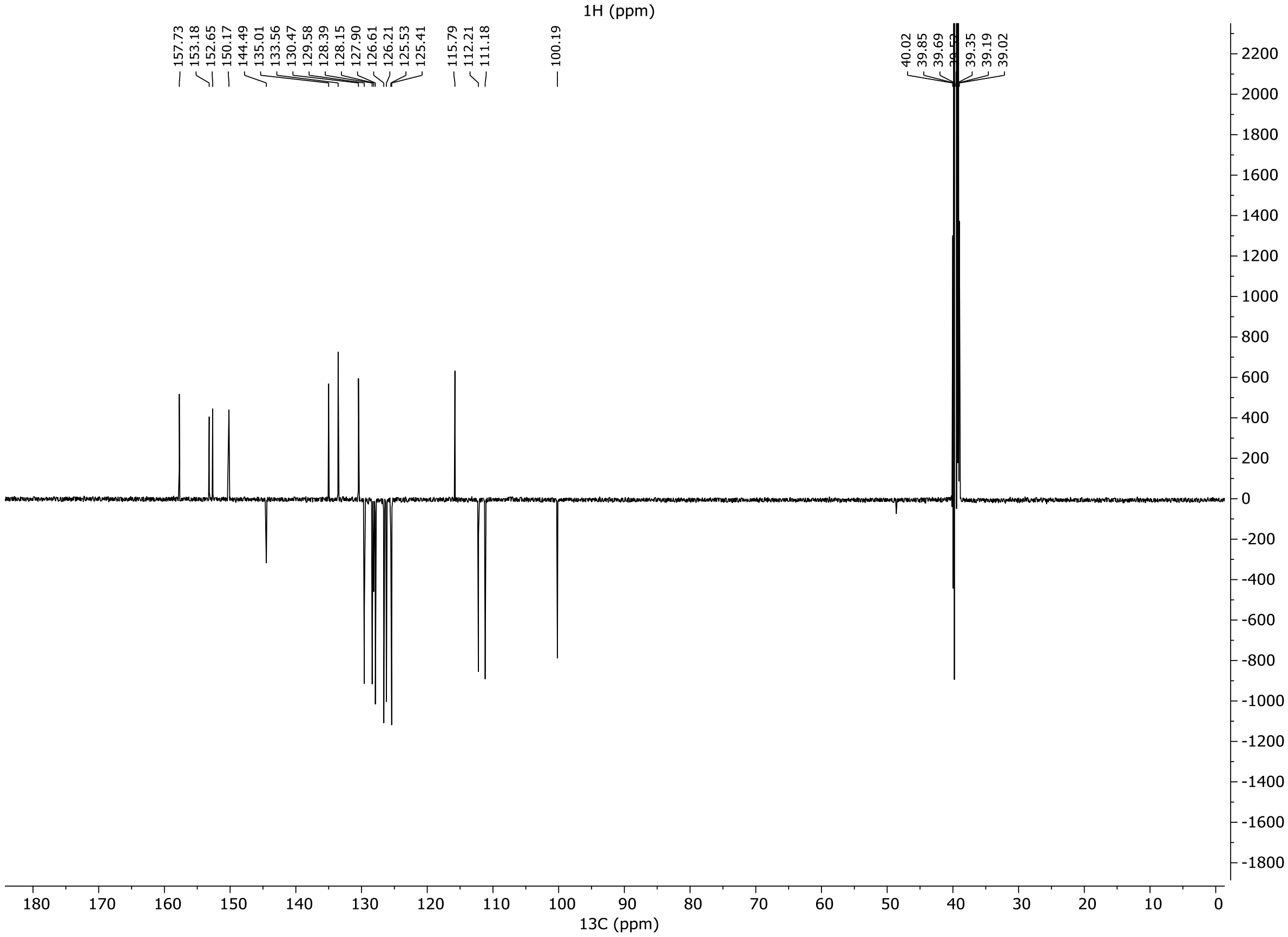

NMR spectra of **5cb**

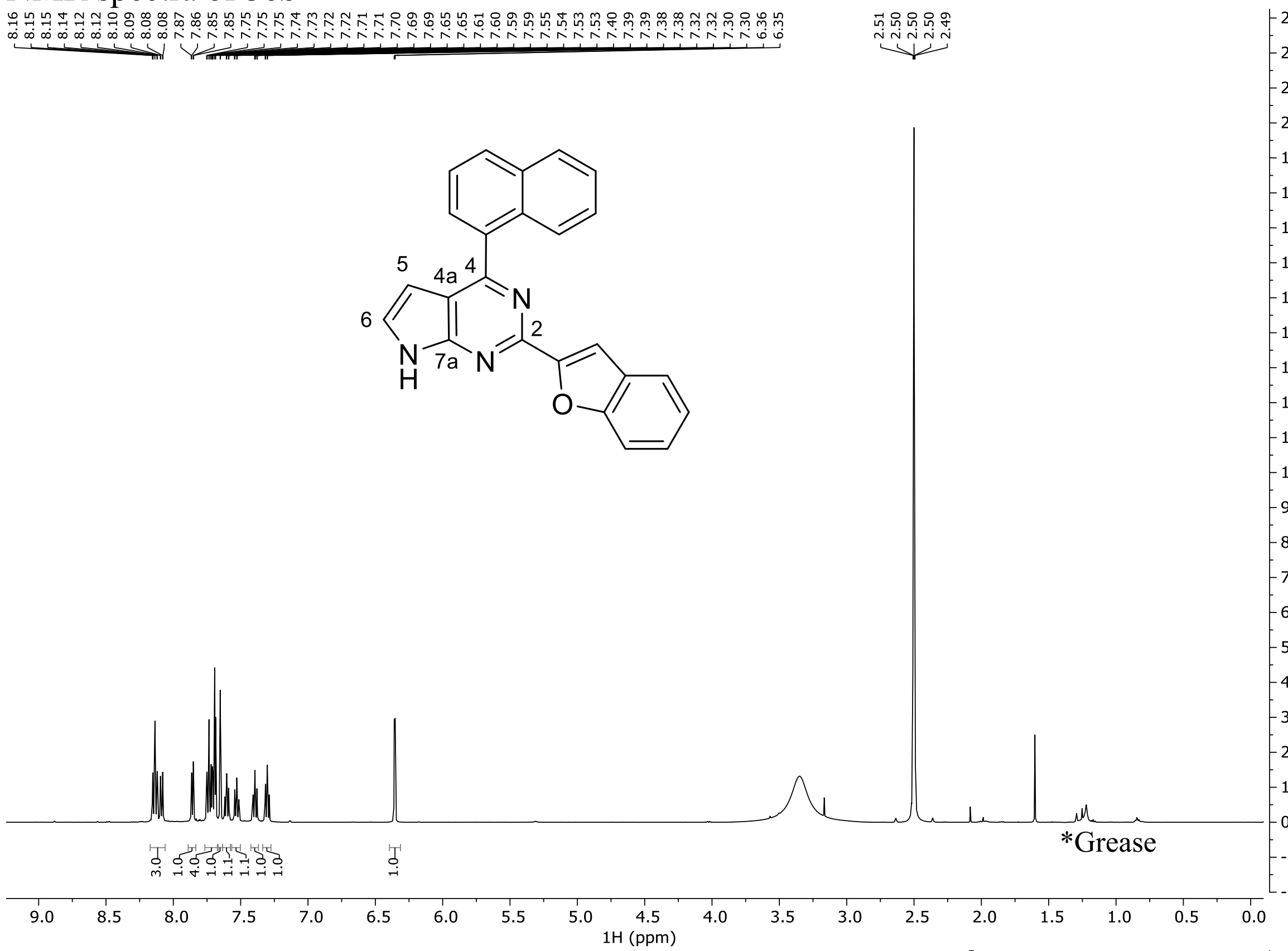

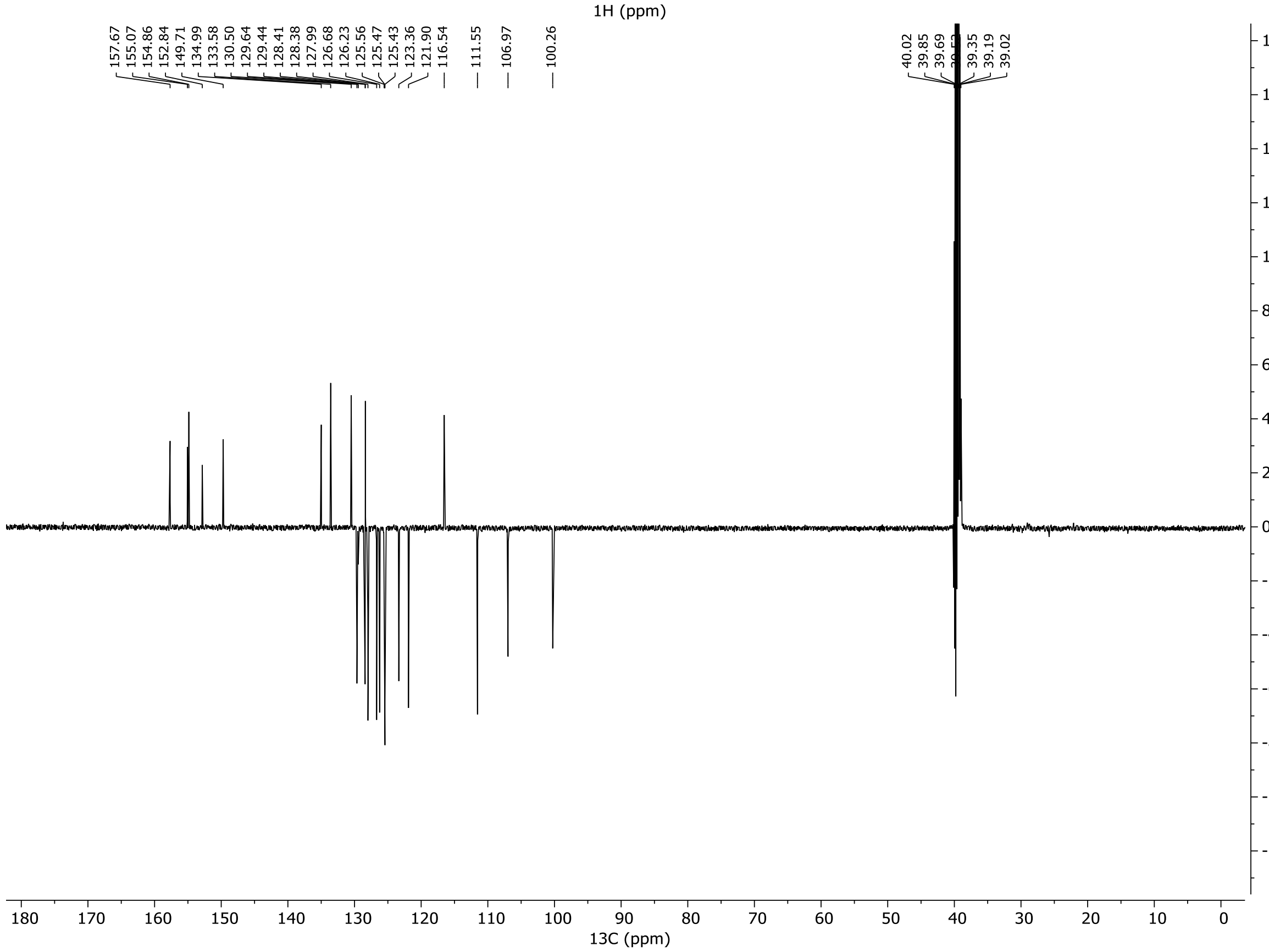

NMR spectra of **5cc**

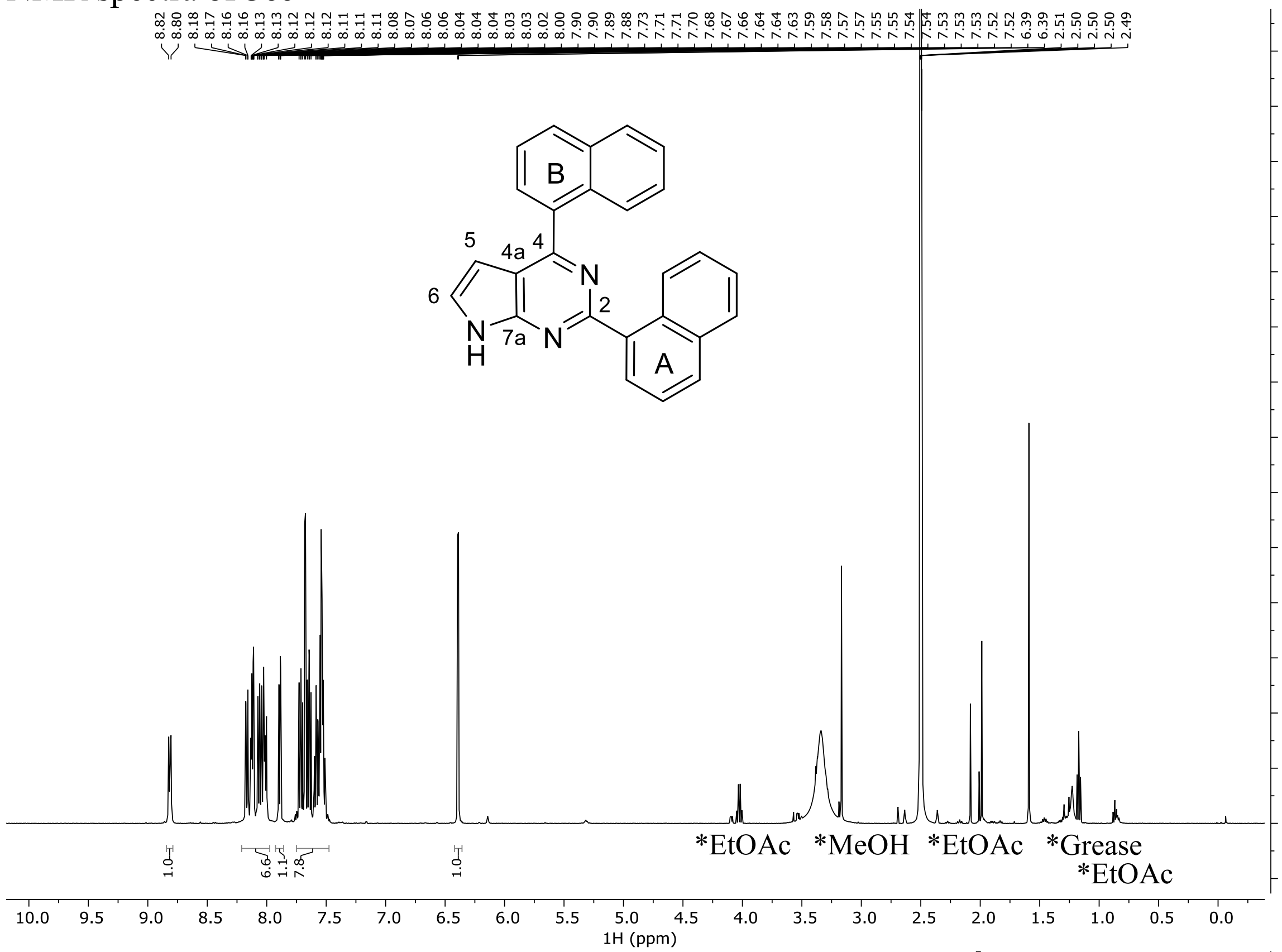

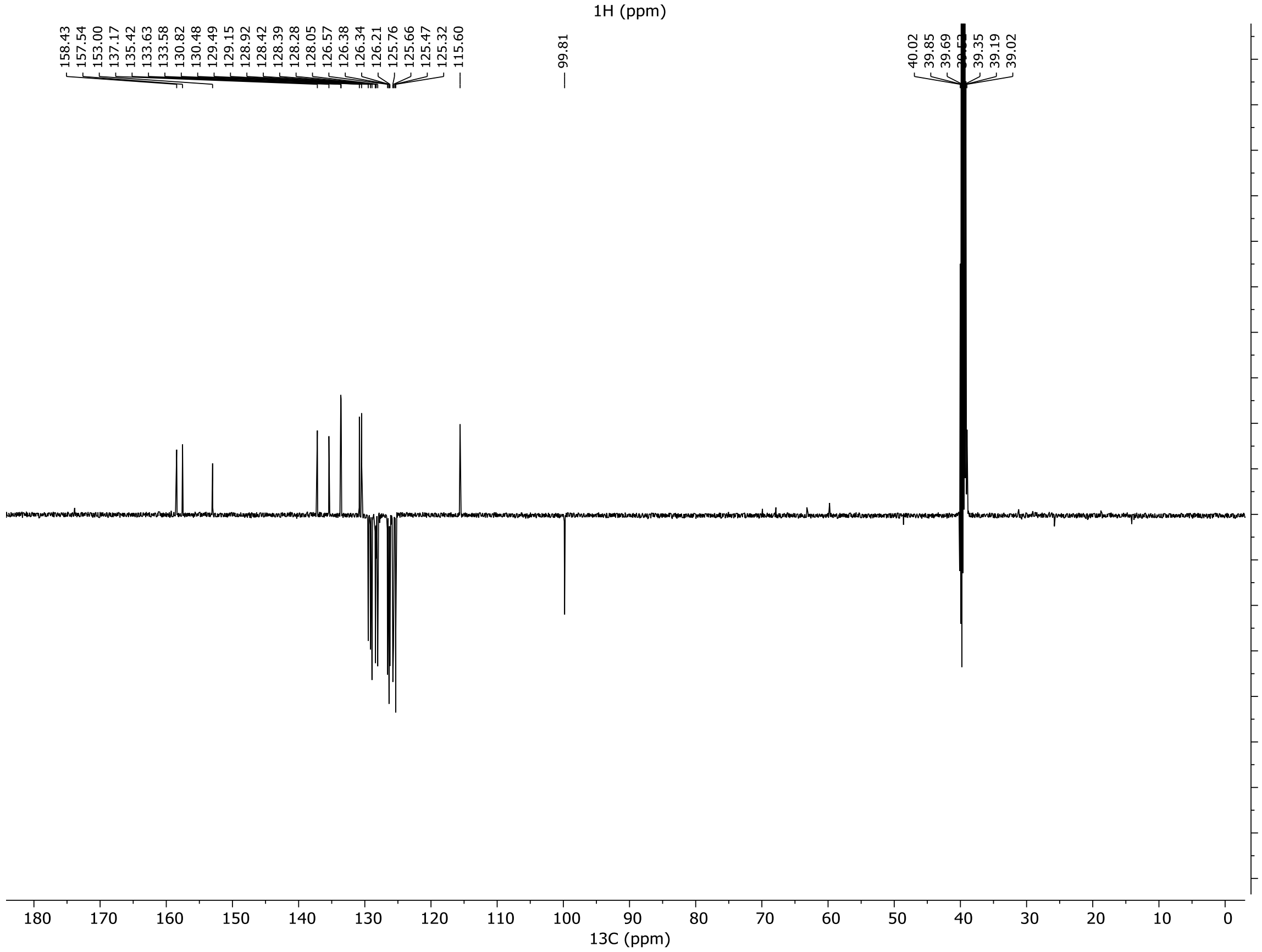

NMR spectra of **5cd**

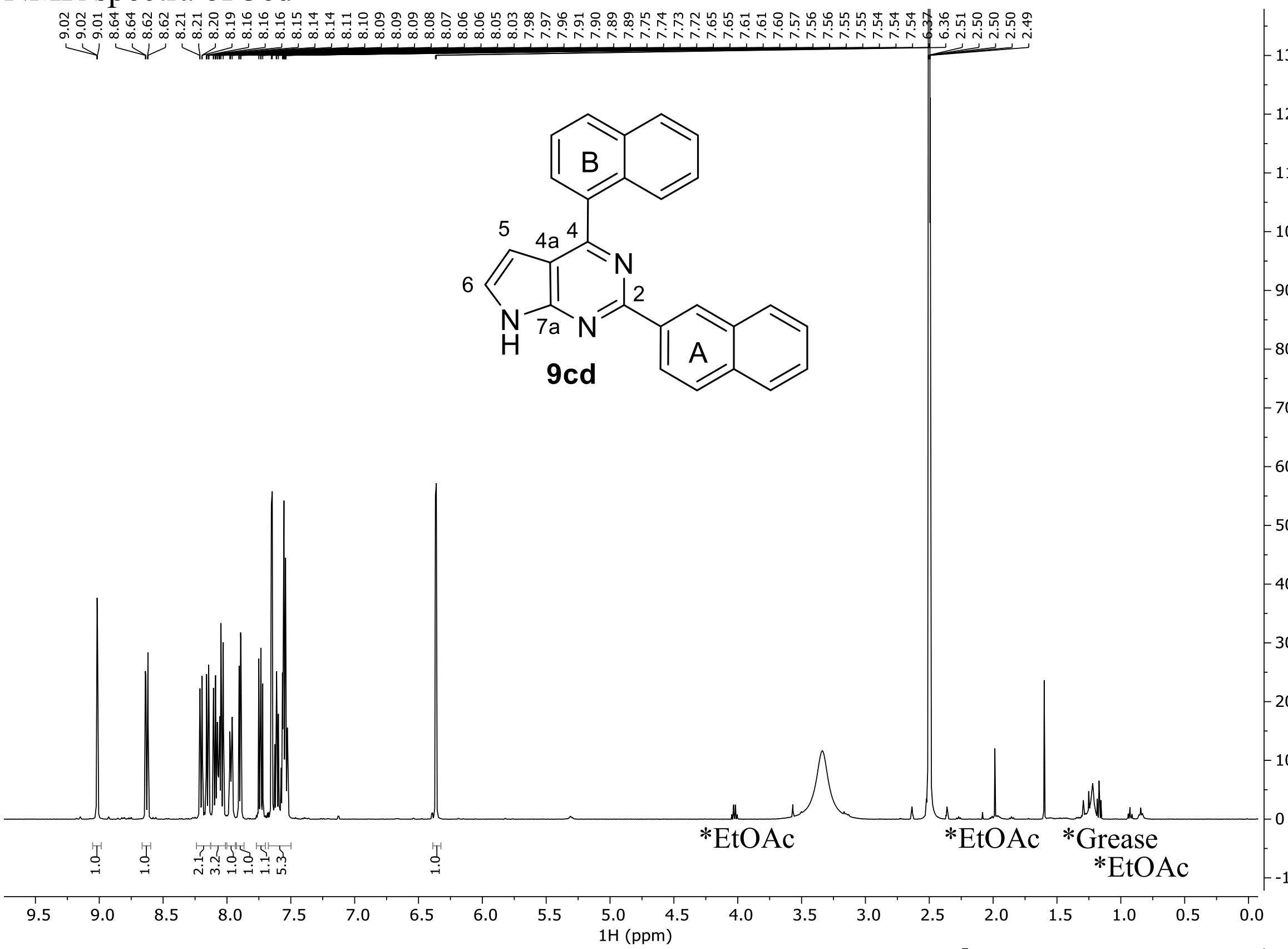

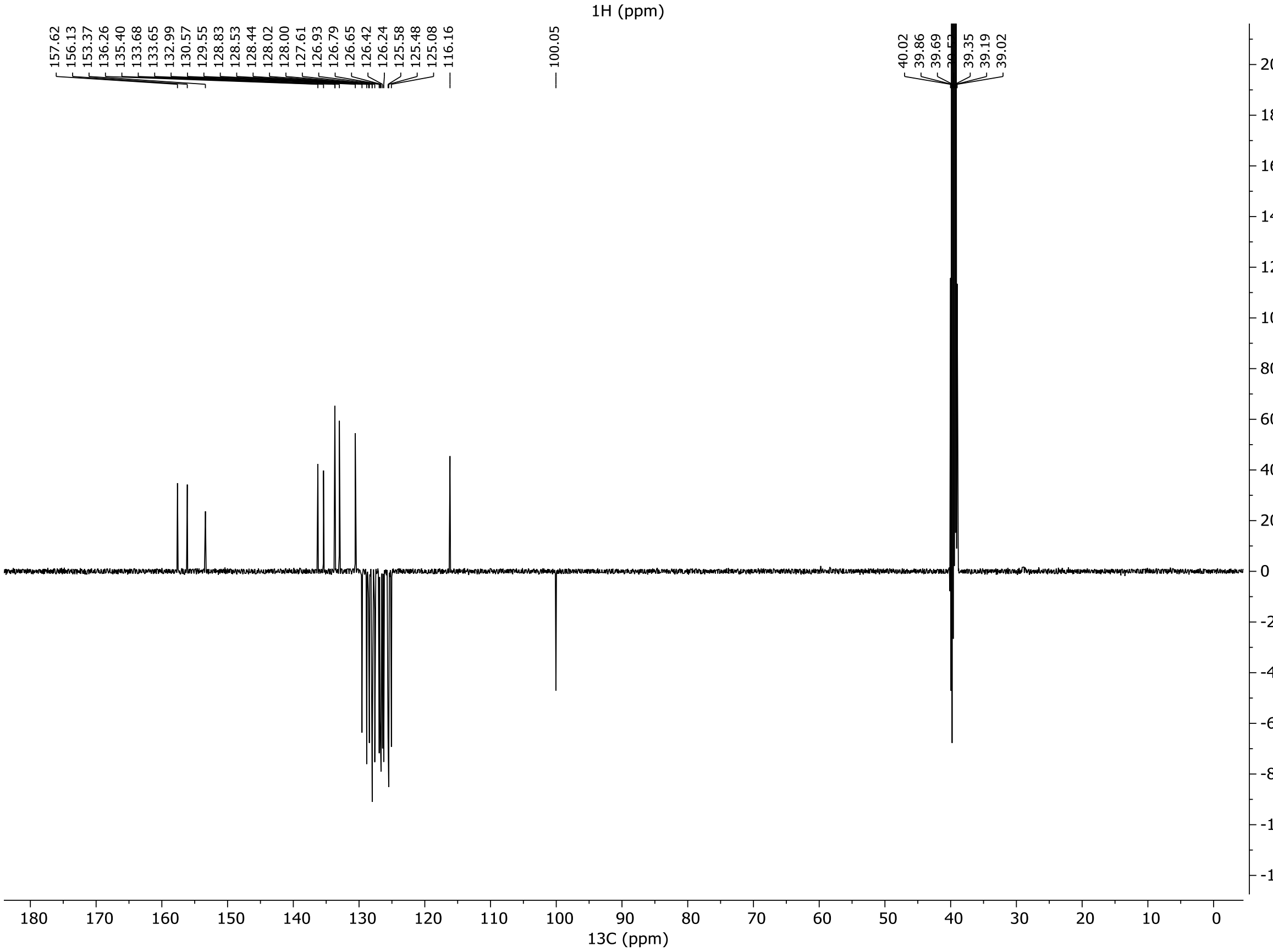

NMR spectra of **5da**

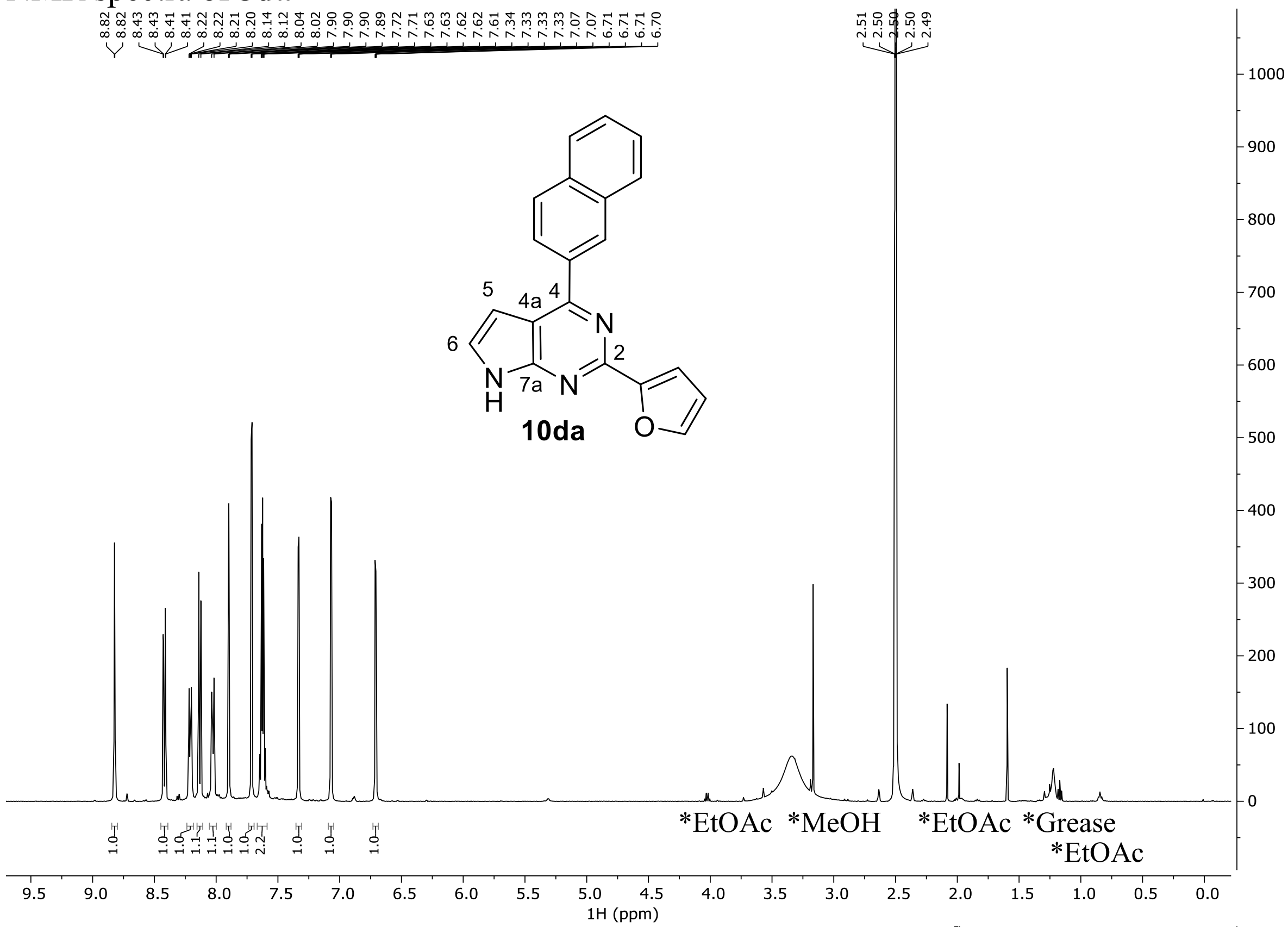

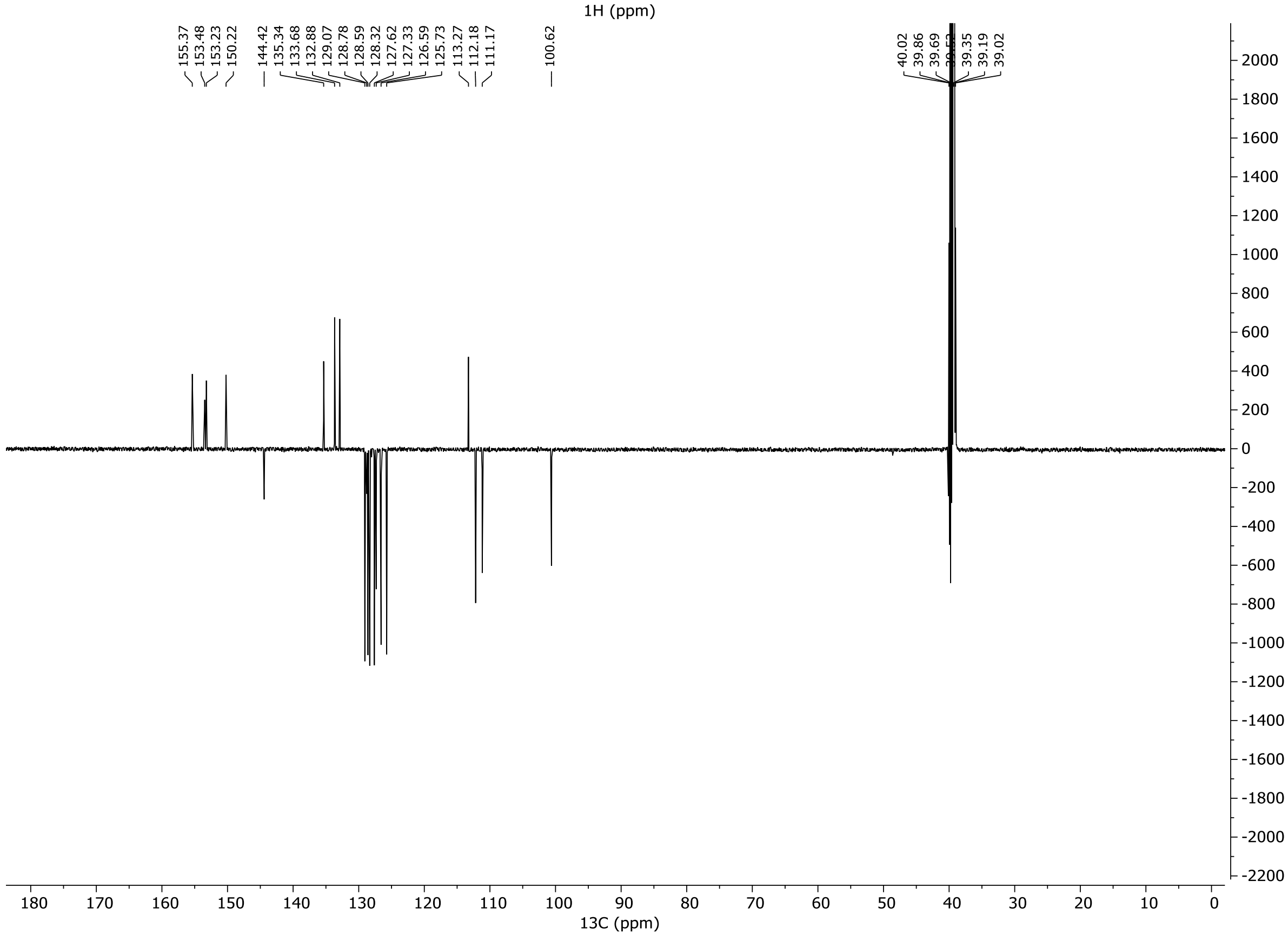

NMR spectra of **5db**

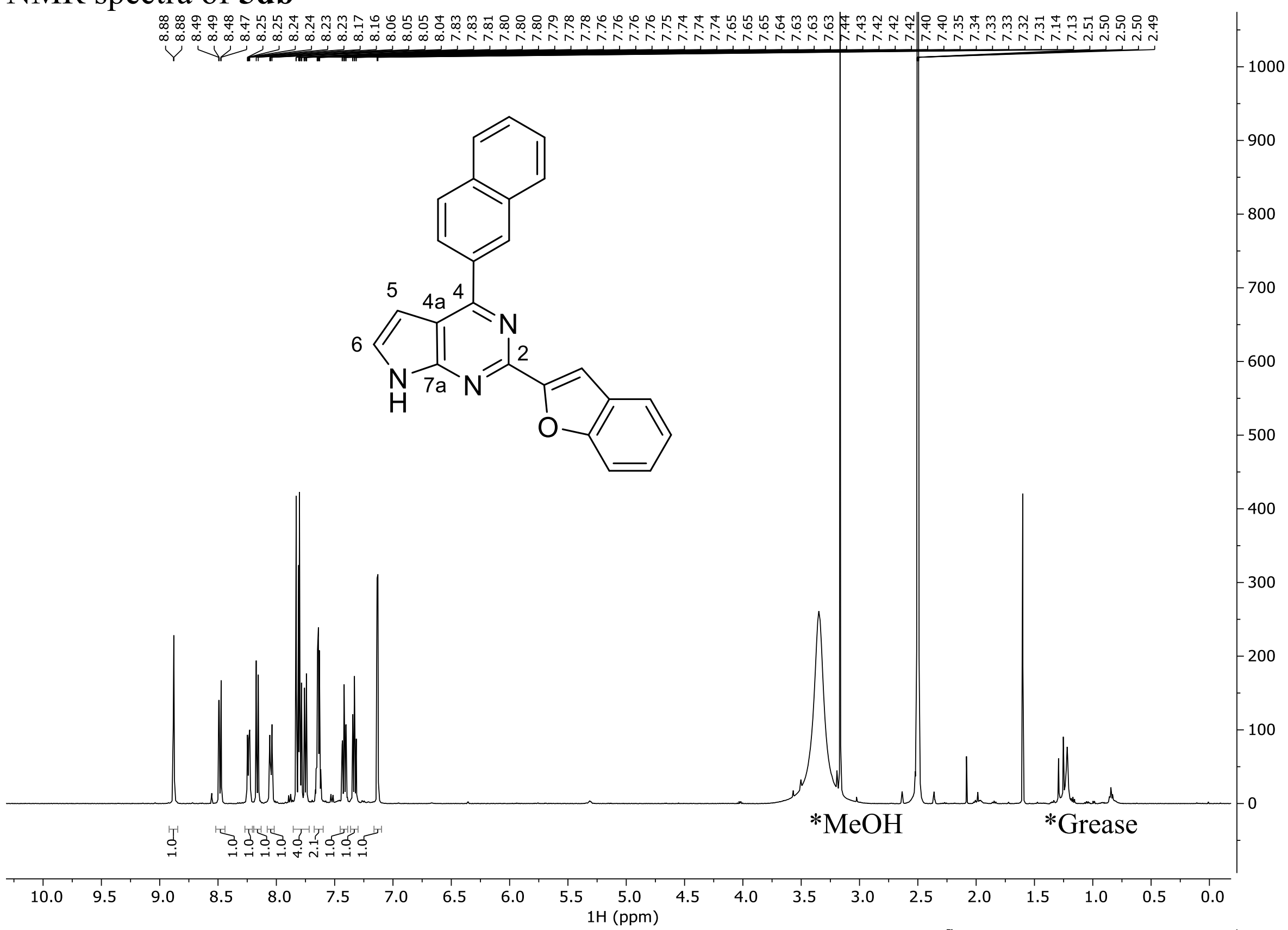

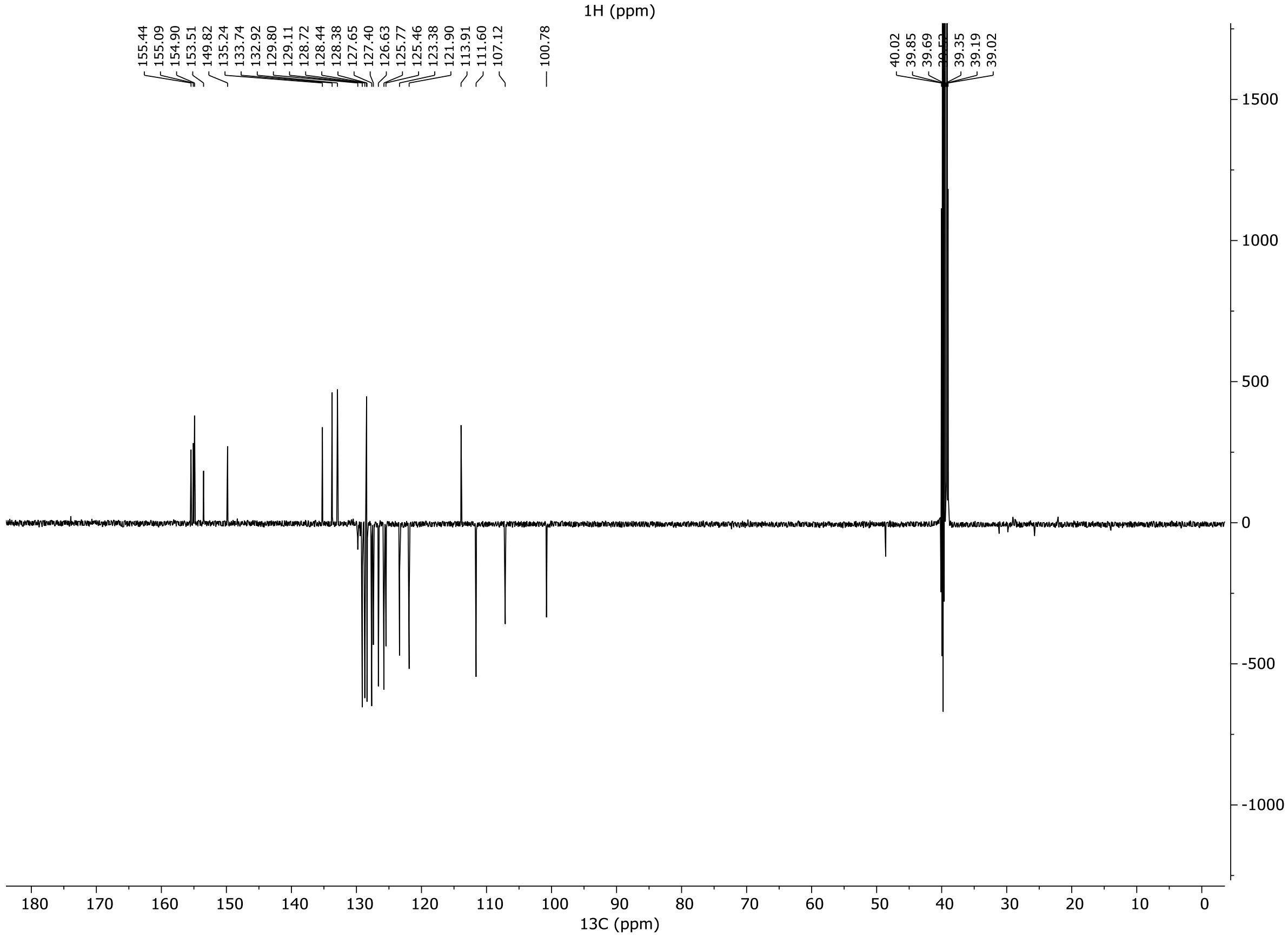

NMR spectra of **5dc**

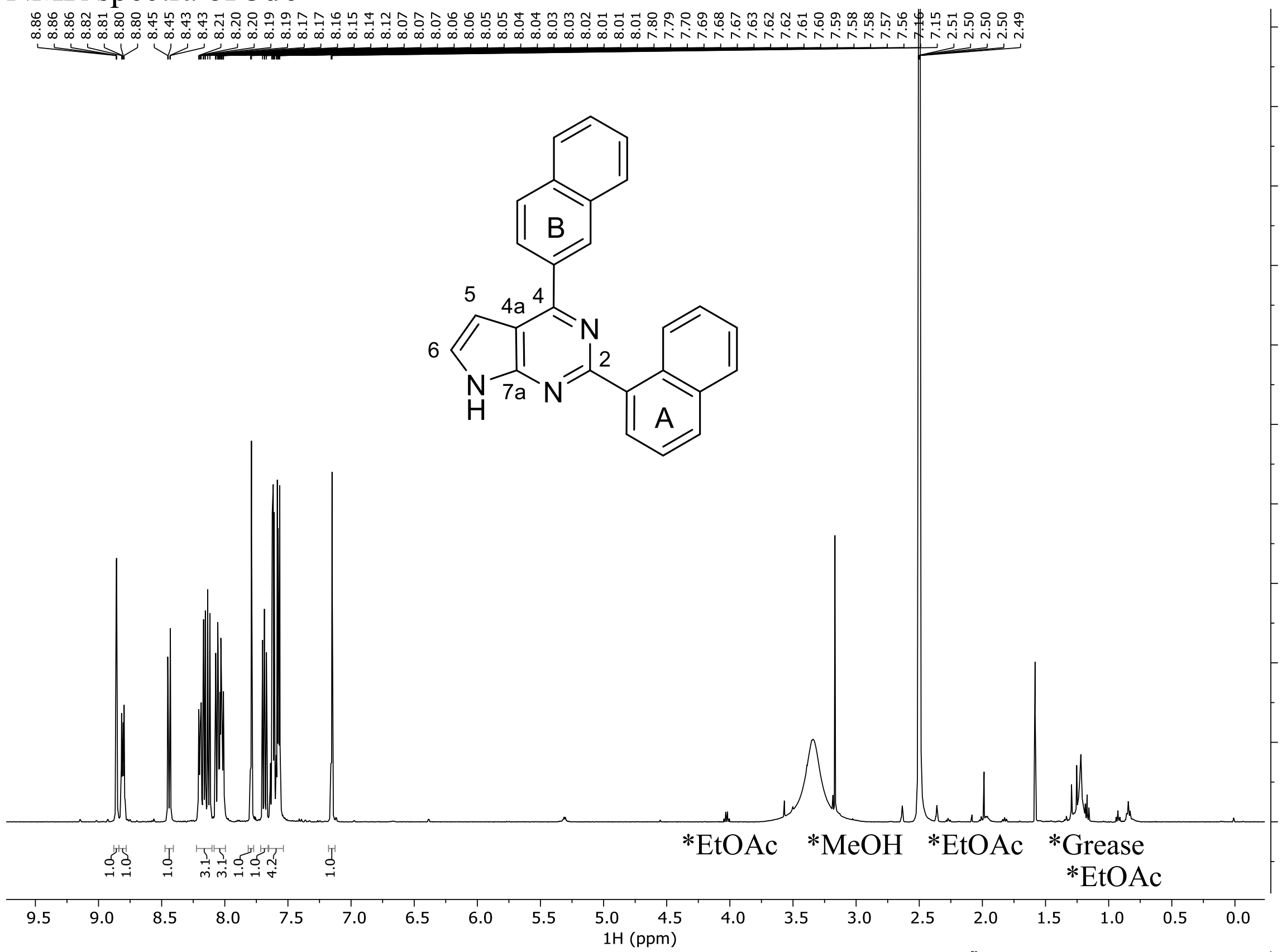

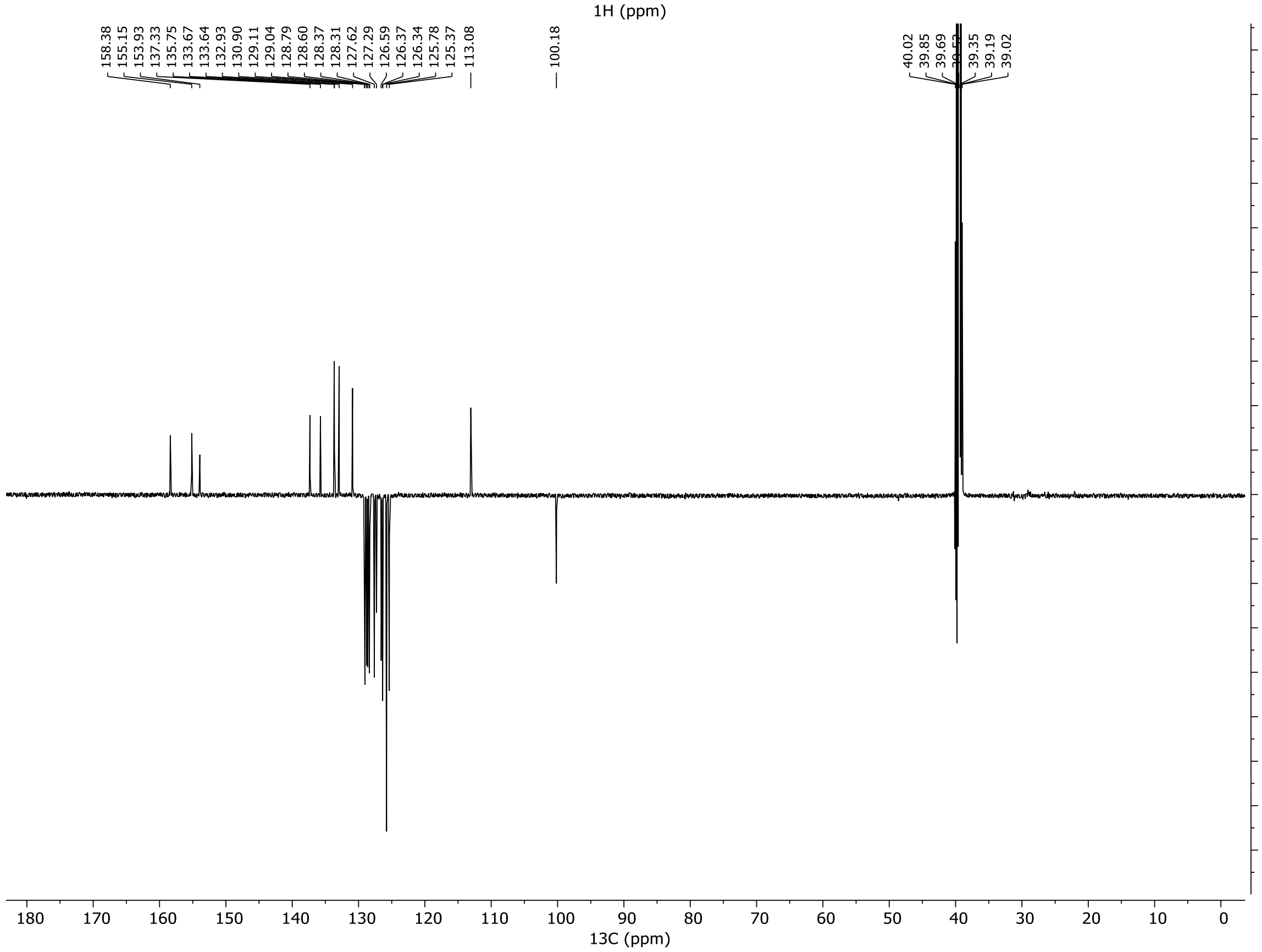

NMR spectra of **5dd**

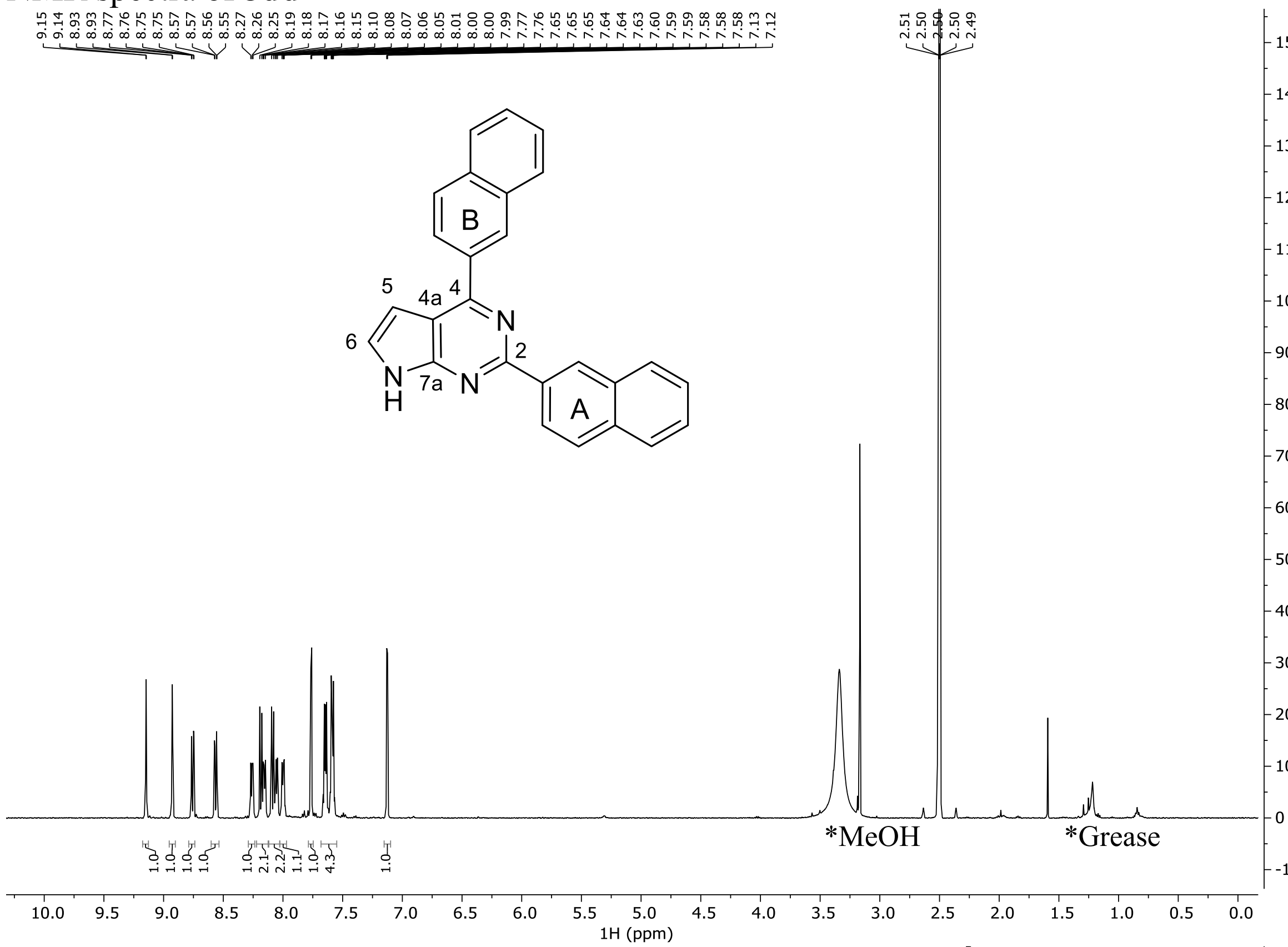

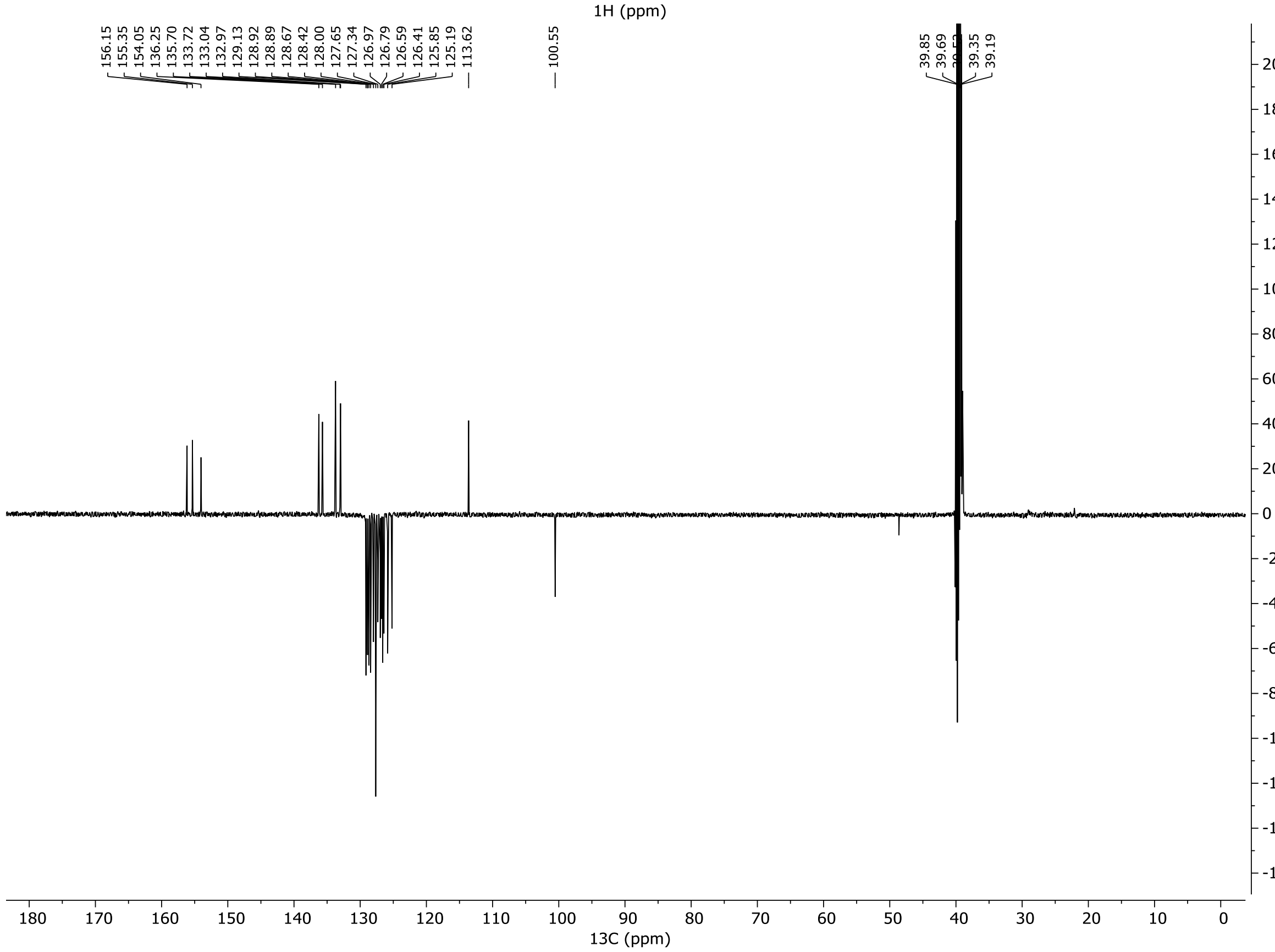

NMR spectra of **7a**

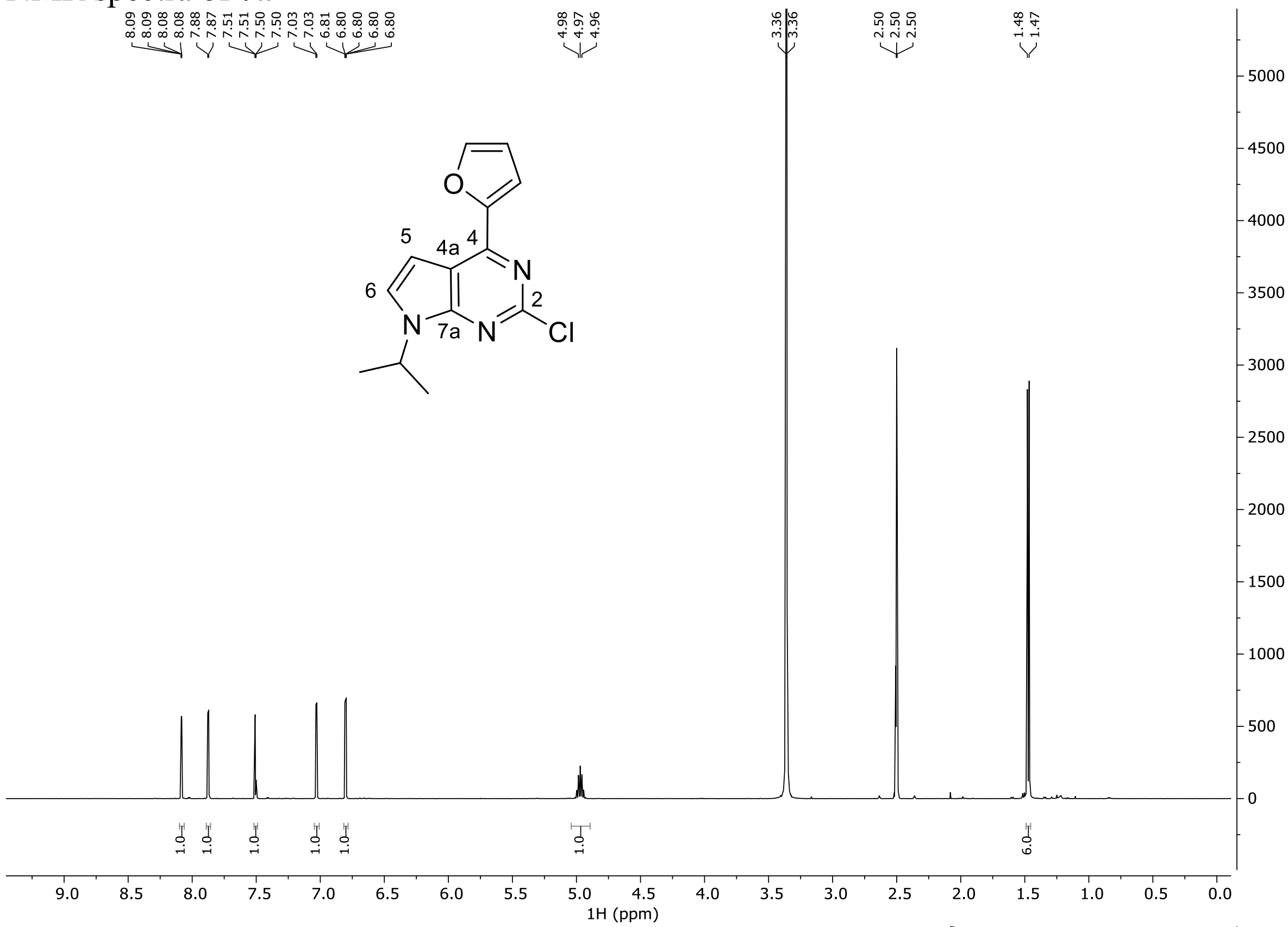

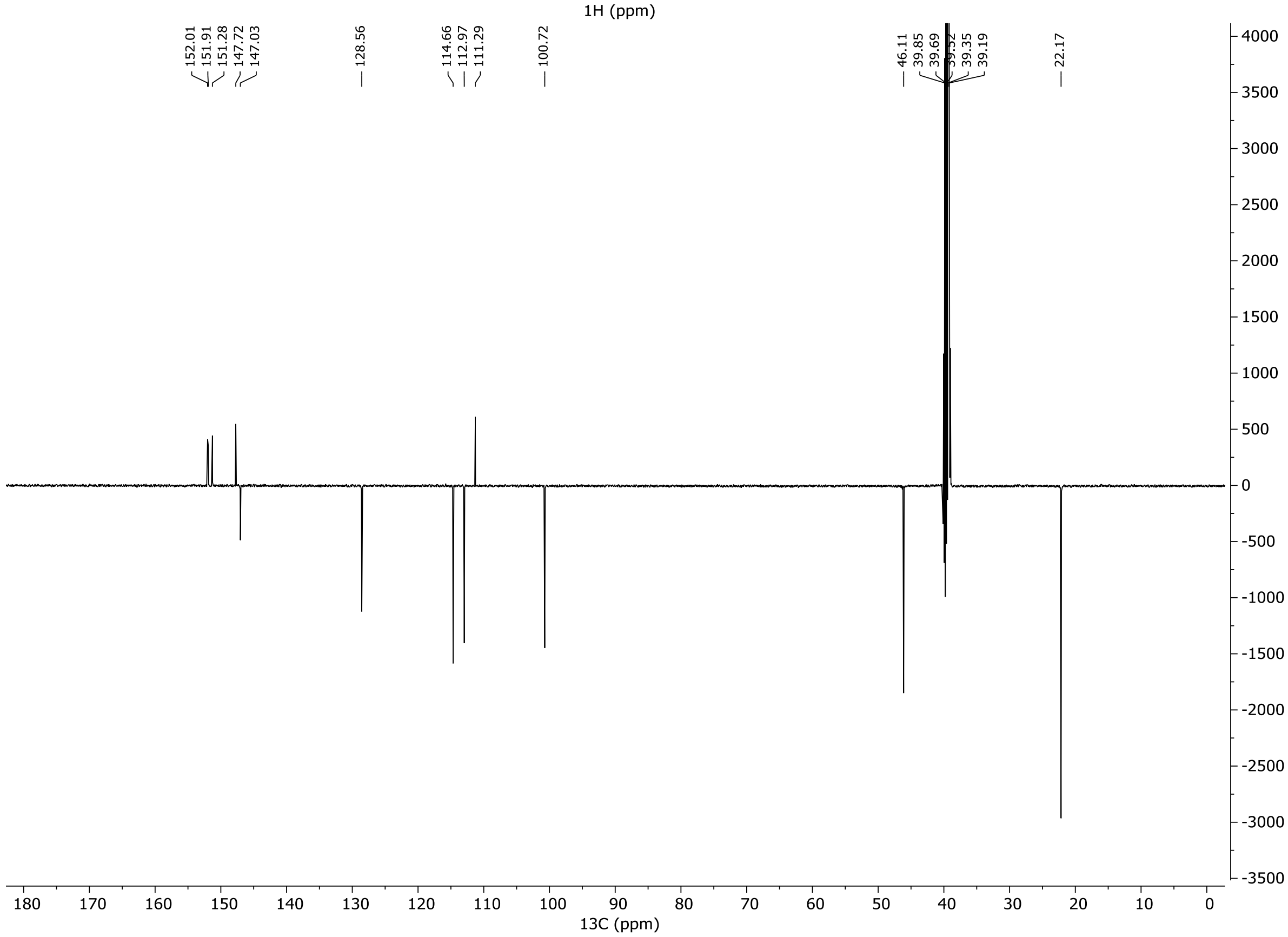

NMR spectra of **8aa**

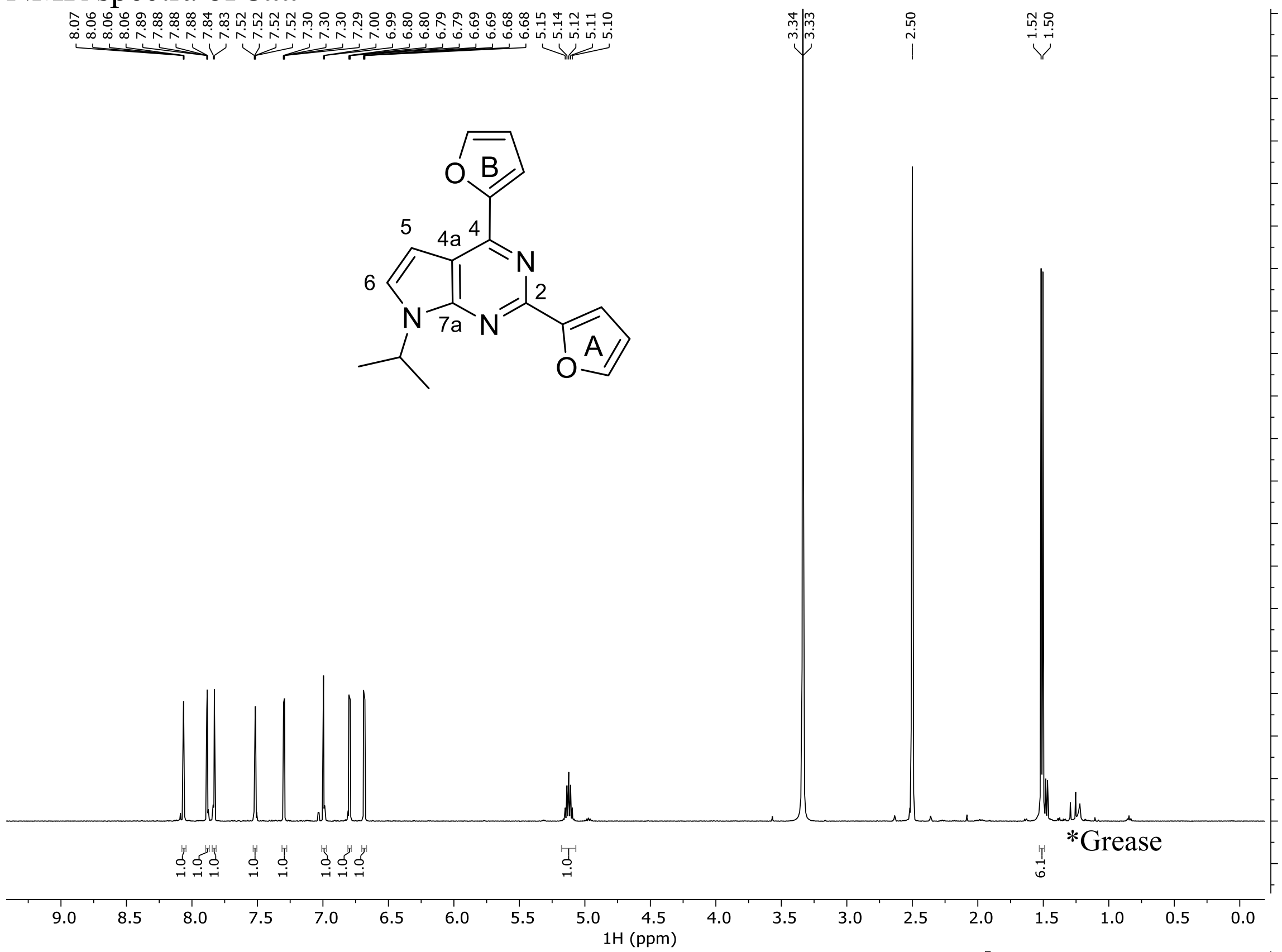

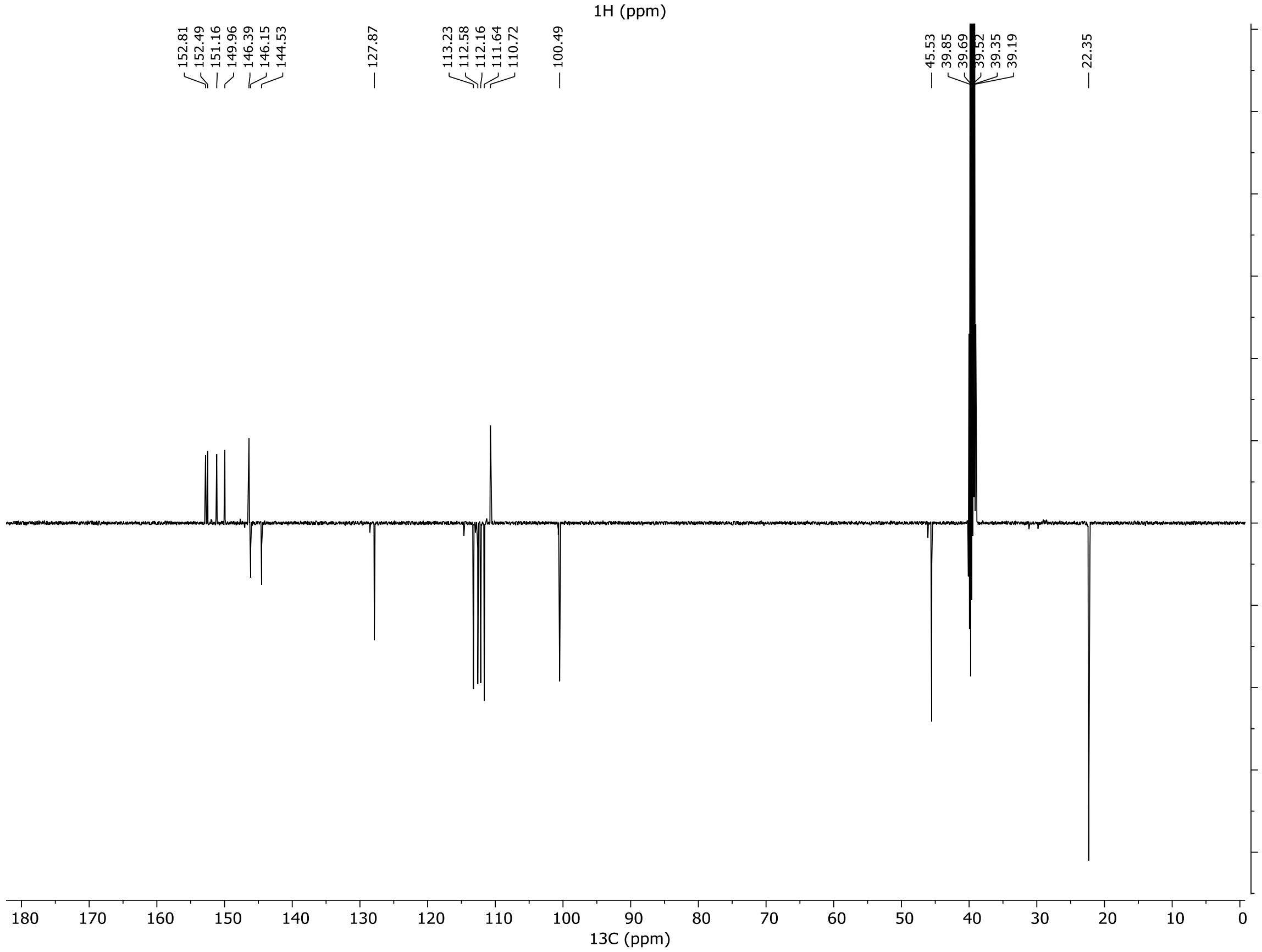

NMR spectra of **8ac**

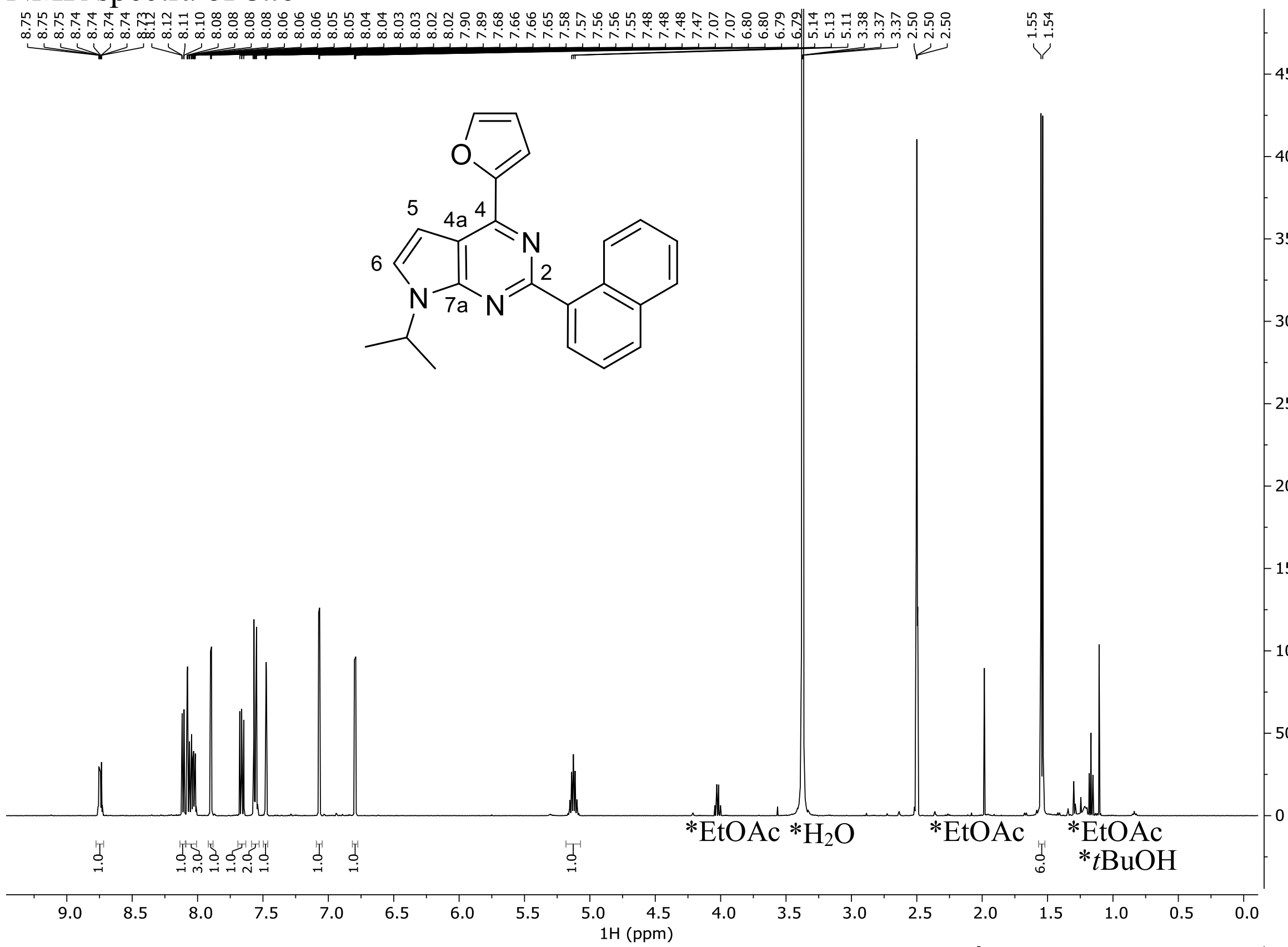

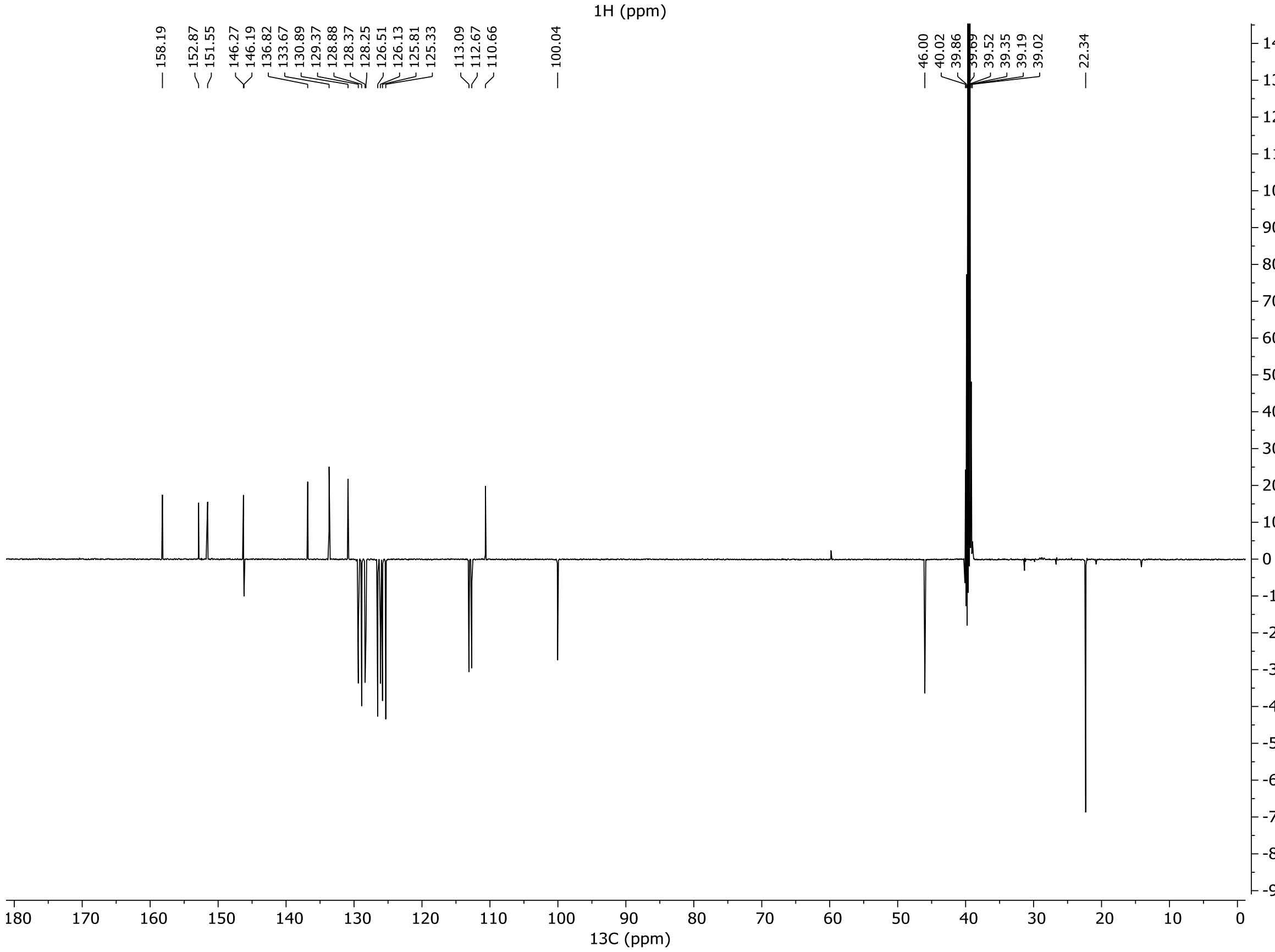

NMR spectra of **9**

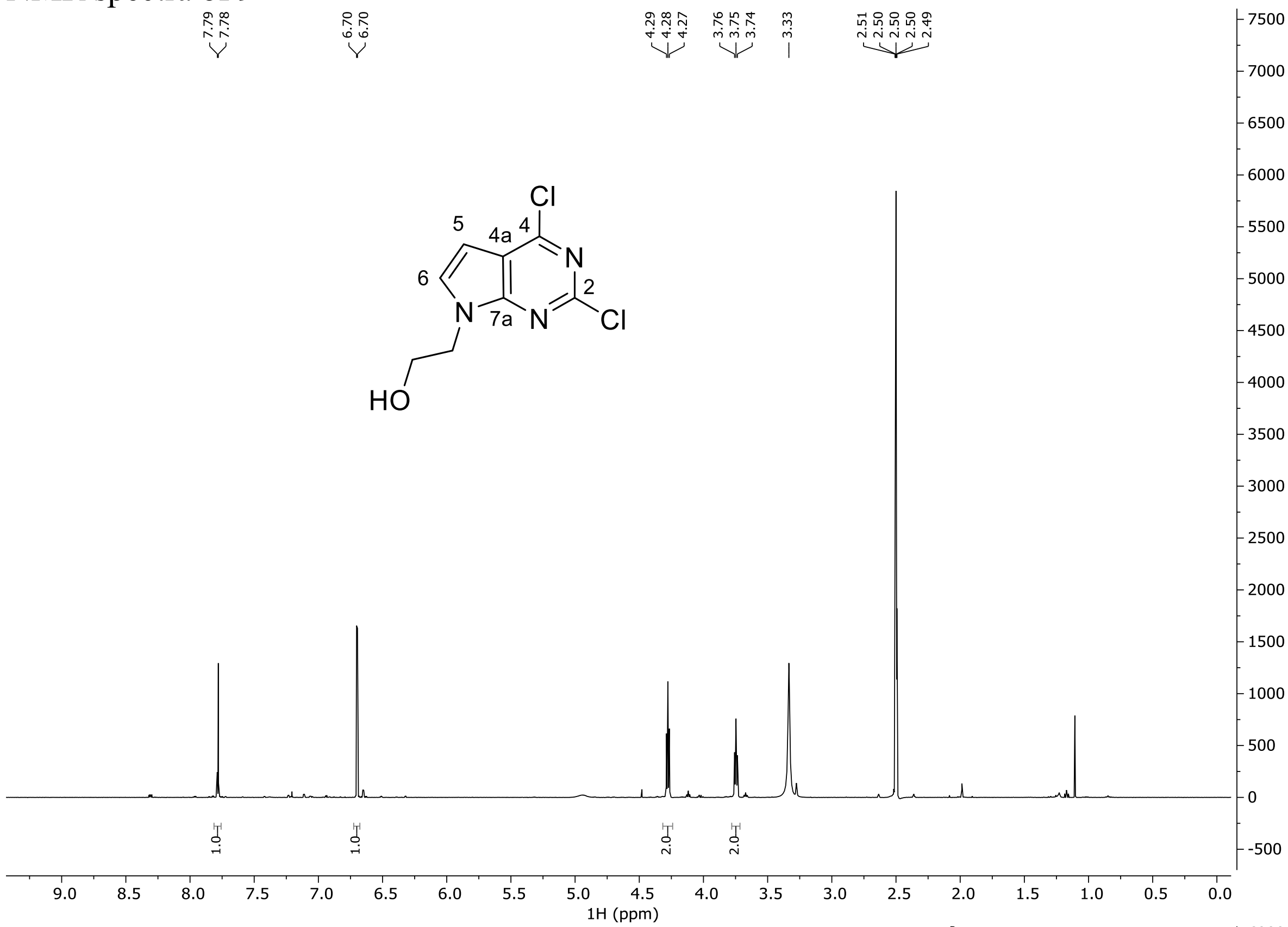

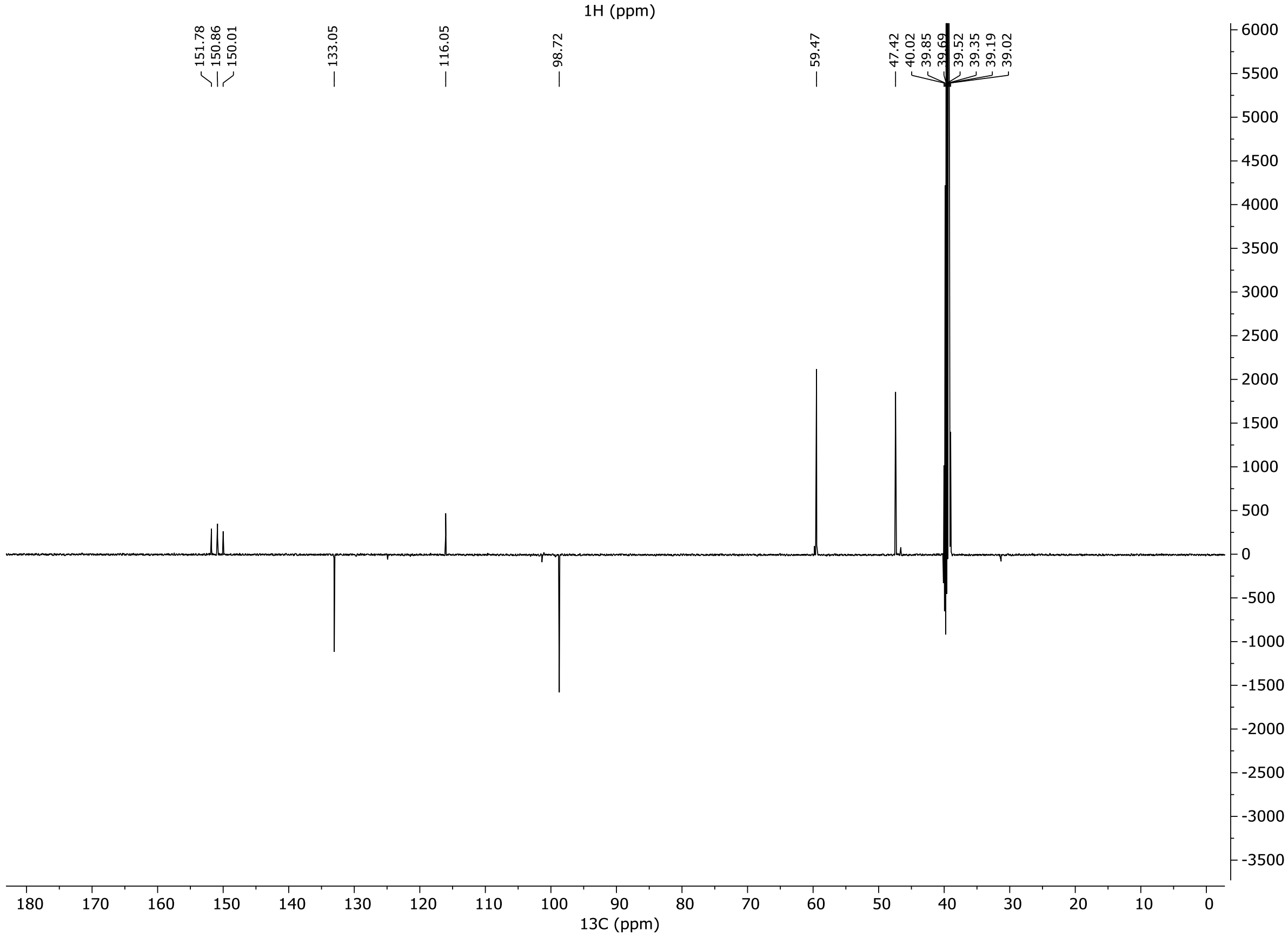

NMR spectra of **10a**

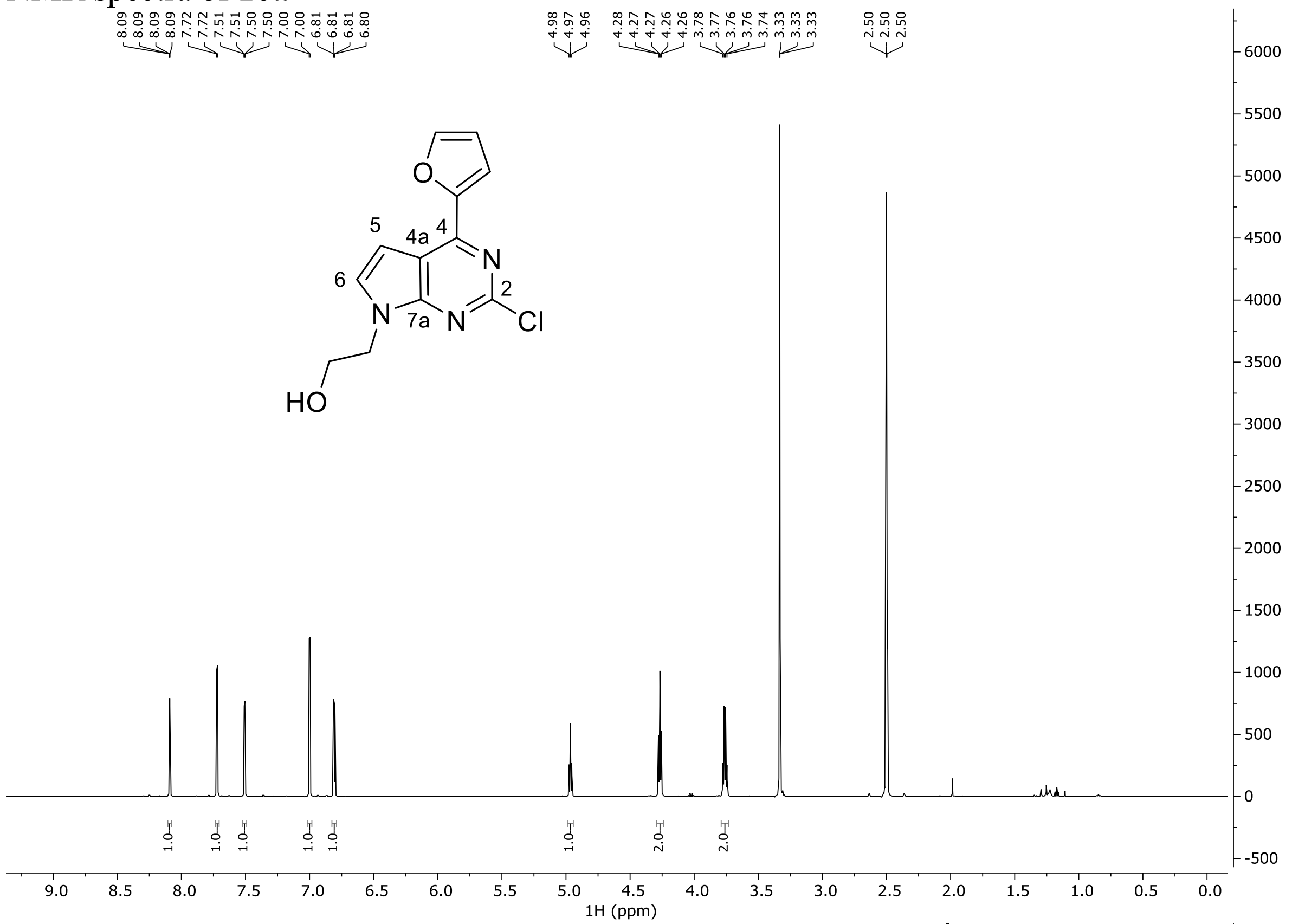

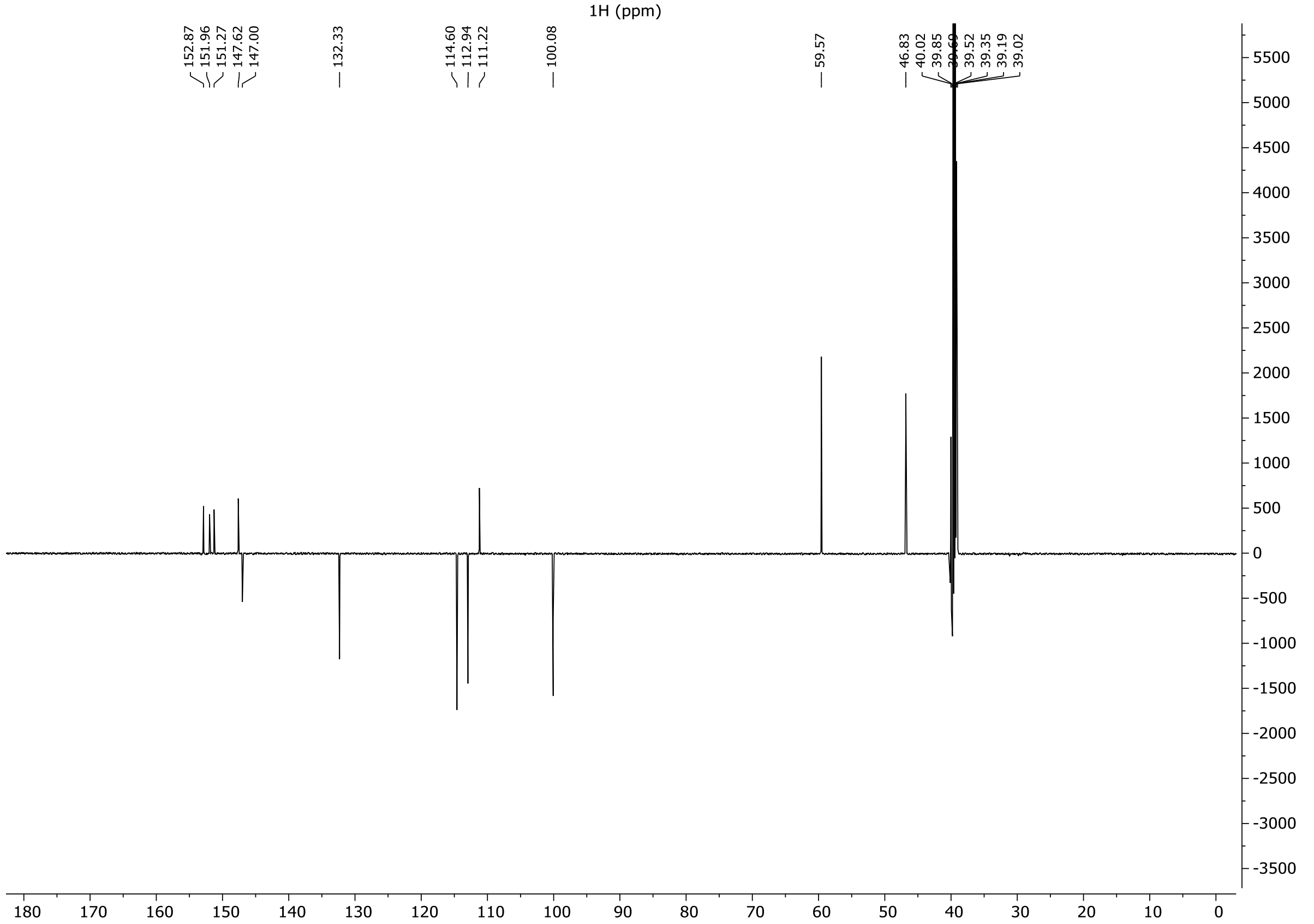

NMR spectra of **11aa**

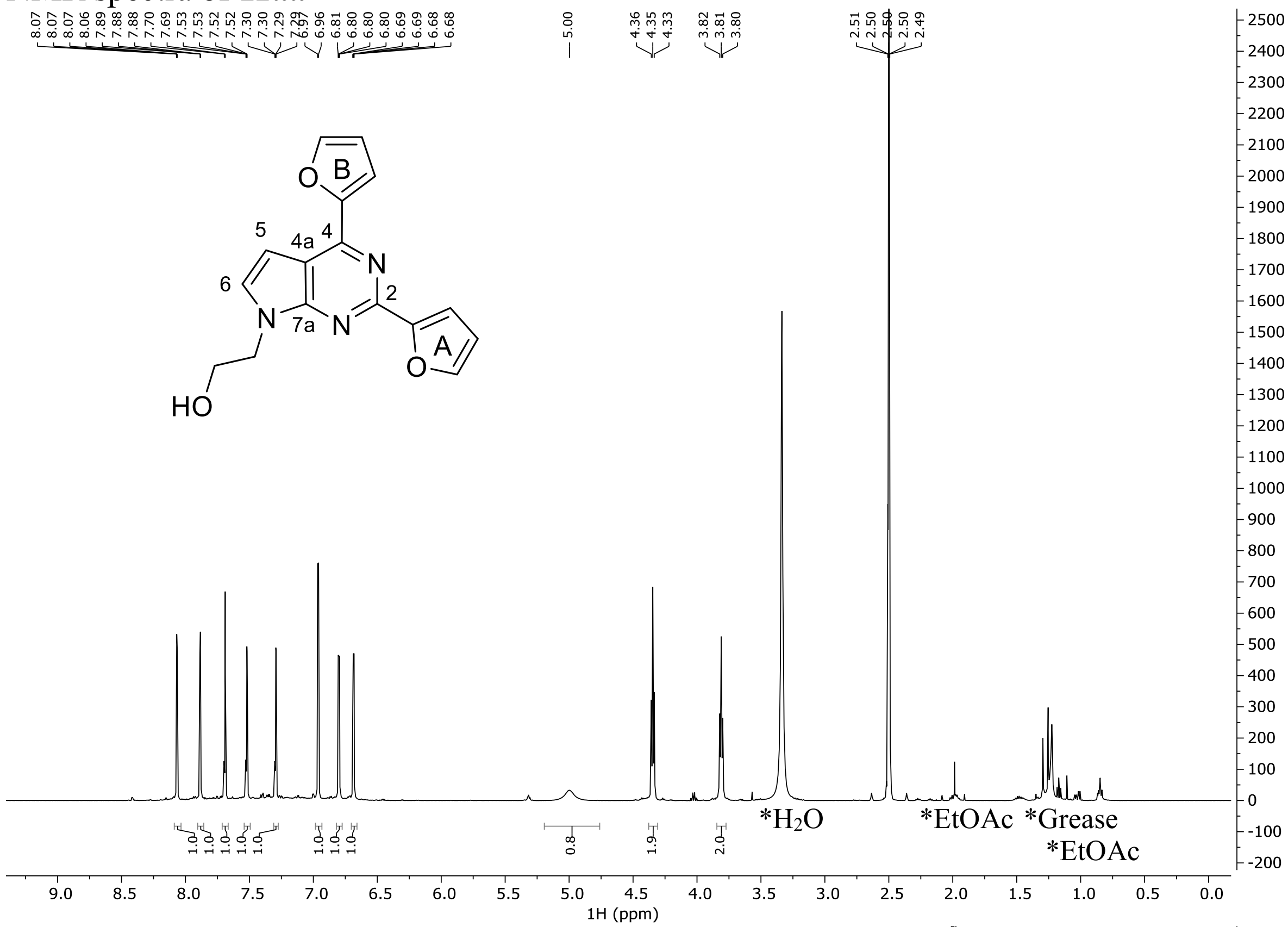

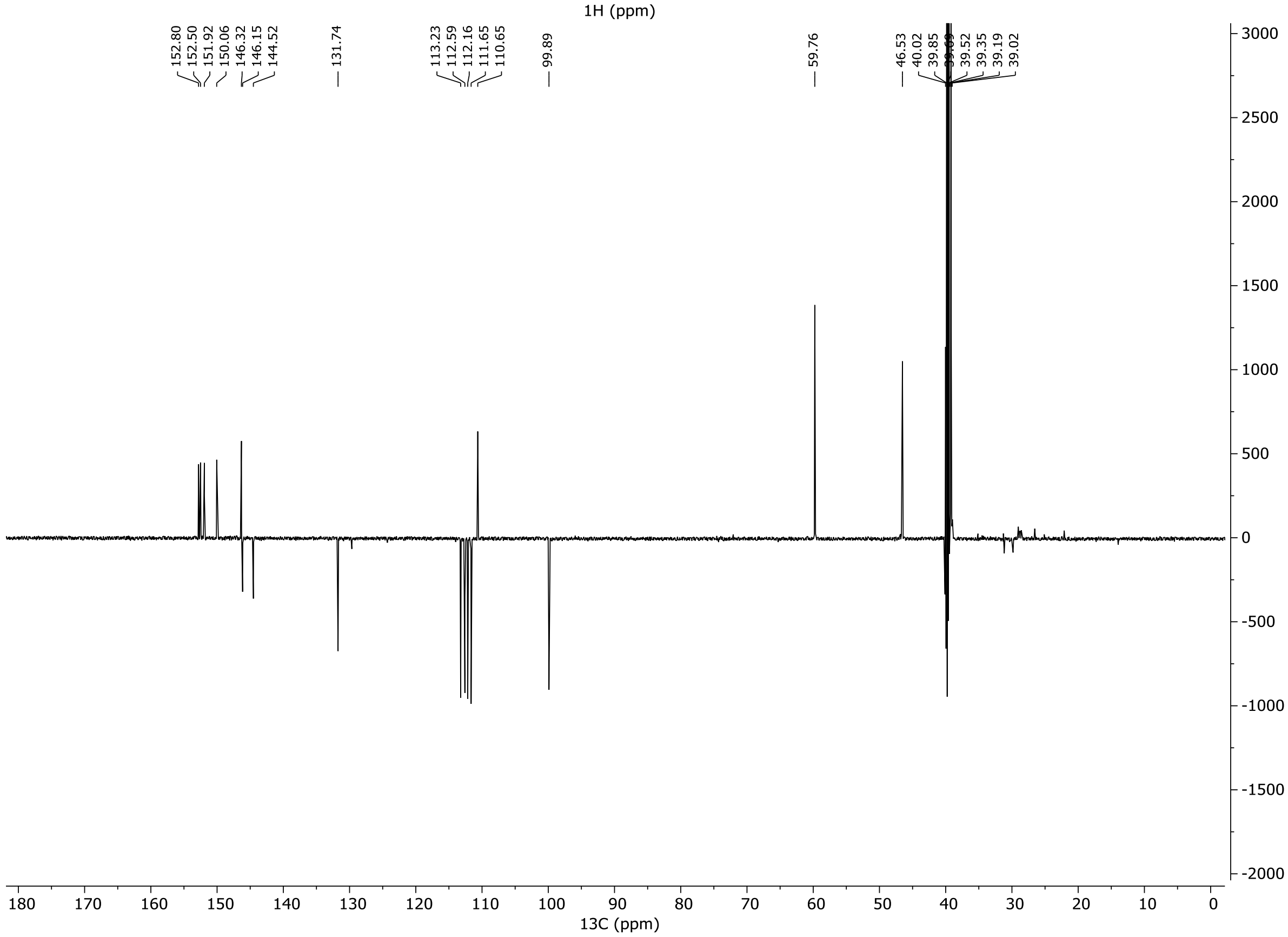

NMR spectra of **11ac**

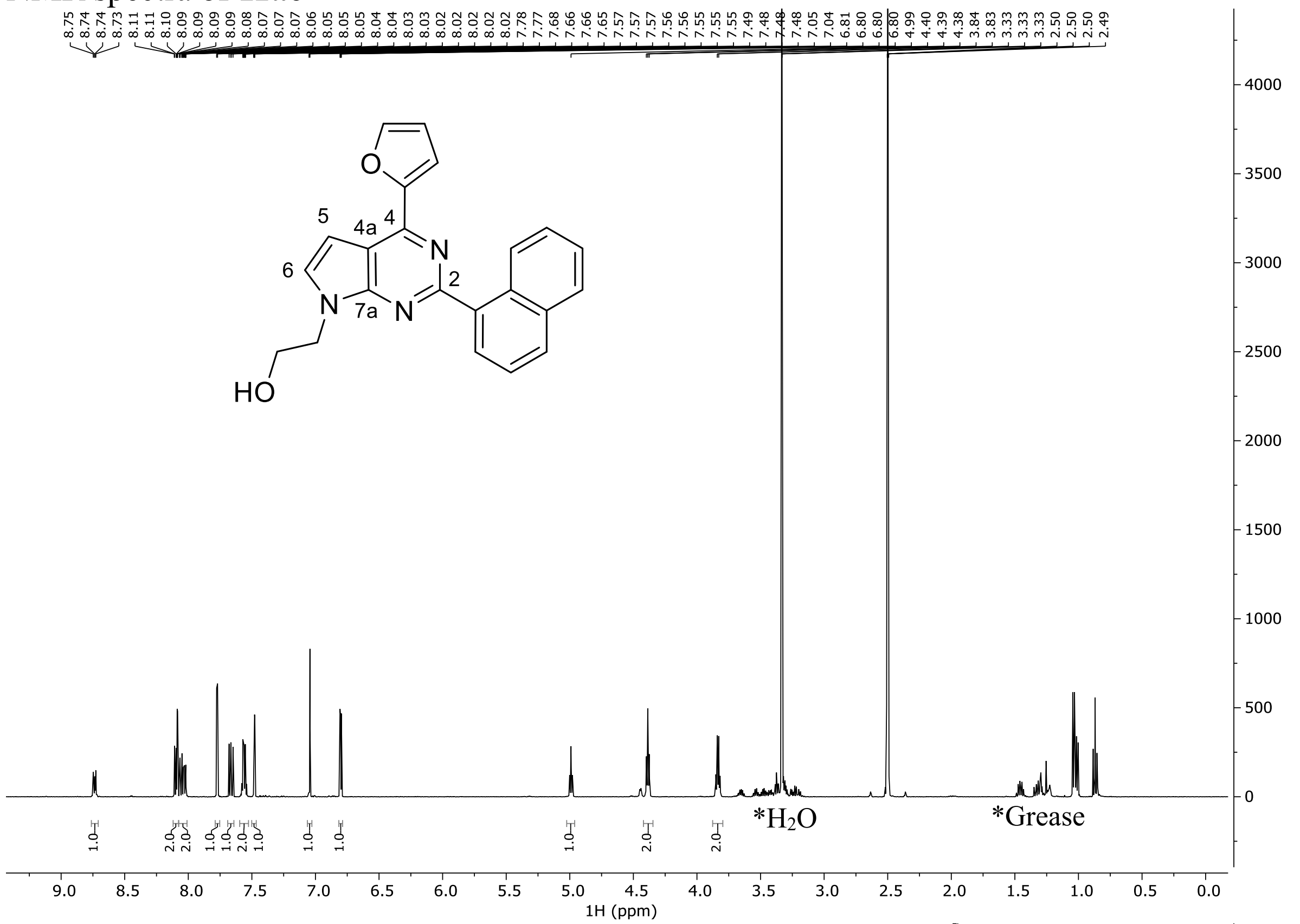

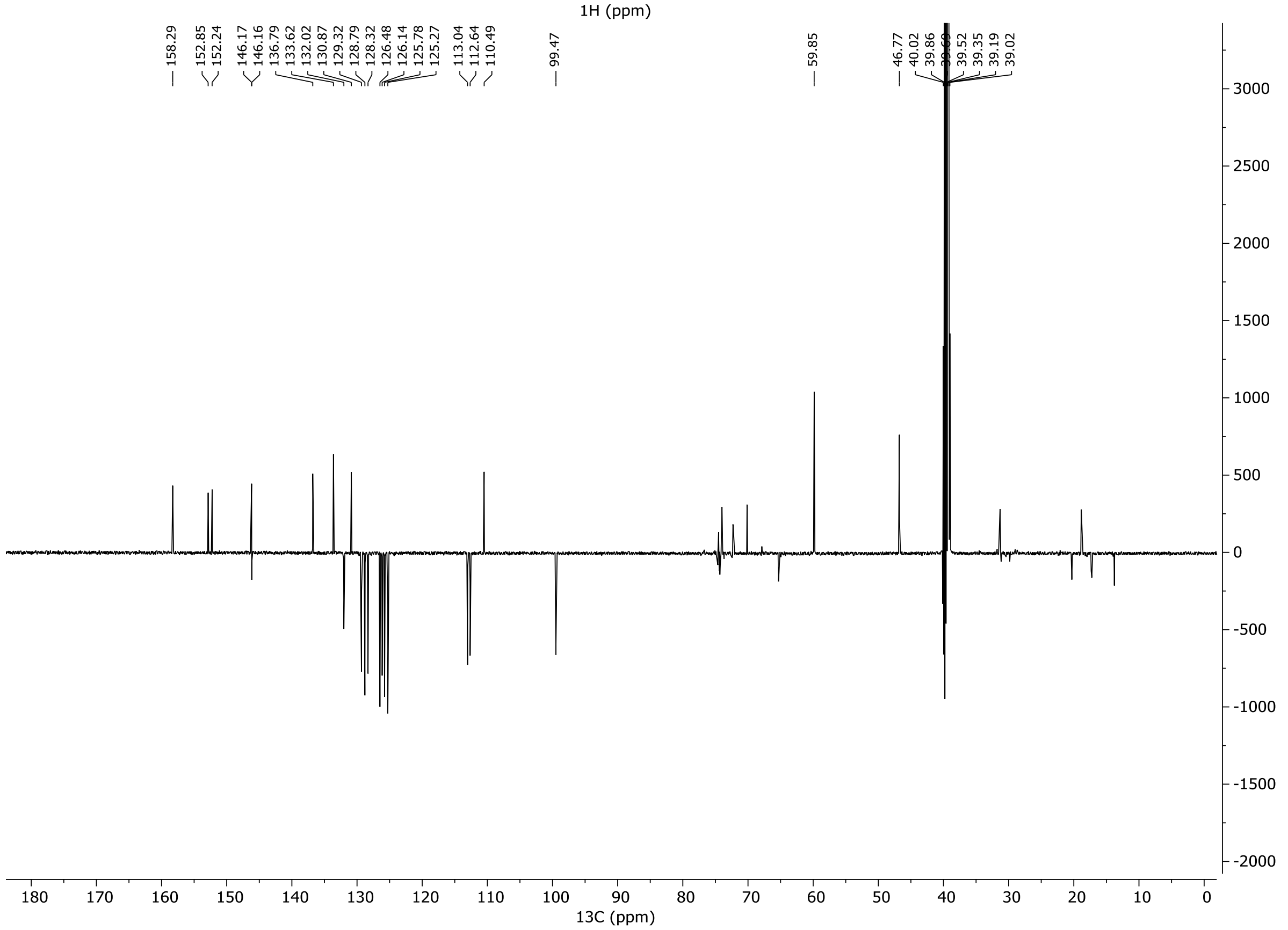

S4. HPLC Purity of Final Compounds

Comp	Method	t_r [min]	Purity [%]	Comp	Method	t_r [min]	Purity [%]
**5aa**	A	6.639	98.83	**5cc**	B	5.873	96.24
**5ab**	B	4.869	96.17	**5cd**	B	7.513	97.89
**5ac**	A	8.363	97.87	**5da**	B	5.890	97.03
**5ad**	B	6.367	97.64	**5db**	B	7.593	97.88
**5ba**	B	5.525	97.11	**5dc**	B	7.040	97.69
**5bb**	B	7.282	99.48	**5dd**	B	8.259	98.93
**5bc**	B	7.306	95.22	**8aa**	B	5.087	95.60
**5bd**	B	8.285	95.58	**8ac**	B	7.190	96.50
**5ca**	B	4.345	96.91	**11aa**	A	6.447	96.13
**5cb**	B	7.516	95.94	**11ac**	A	8.238	97.58

Mobile phases: H_2O + 0.1 % FA and MeOH + 0.1 % FA, Methods: A – 40 % to 100 % in 12.5 min; B – 60 % to 100 % in 12.5 min

S5. References

[1] J. R. Riggs, J. Elsner, D. Cashion, D. Robinson, L. Tehrani, M. Nagy, K. E. Fultz, R. Krishna Narla, X. Peng, T. Tran, A. Kulkarni, S. Bahmanyar, K. Condroski, B. Pagarigan, G. Fenalti, L. Lebrun, K. Leftheris, D. Zhu, J. F. Boylan, "Design and Optimization Leading to an Orally Active TTK Protein Kinase Inhibitor with Robust Single Agent Efficacy" *J. Med. Chem.*, **2019**, *62*, 4401–4410.

[2] E. Le Poul, S. Hisada, Y. Mizuguchi, V. J. Dupriez, E. Burgeon, M. Detheux, "Adaptation of aequorin functional assay to high throughput screening" *J. Biomol. Screen*. **2002**, *7*, 57–65.
